# Achieving Consensus for the Design and Delivery of an Online Intervention to Support Midwives in Work-Related Psychological Distress: Results From a Delphi Study

**DOI:** 10.2196/mental.5617

**Published:** 2016-07-12

**Authors:** Sally Pezaro, Wendy Clyne

**Affiliations:** ^1^ Centre for Technology Enabled Health Research Faculty of Health and Life Sciences Coventry University Coventry United Kingdom

**Keywords:** Delphi technique, Internet, intervention studies, midwifery, psychological, health workforce, self-help groups, stress

## Abstract

**Background:**

Some midwives are known to experience both professional and organizational sources of psychological distress, which can manifest as a result of the emotionally demanding midwifery work, and the traumatic work environments they endure. An online intervention may be one option midwives may engage with in pursuit of effective support. However, the priorities for the development of an online intervention to effectively support midwives in work-related psychological distress have yet to be explored.

**Objective:**

The aim of this study was to explore priorities in the development of an online intervention to support midwives in work-related psychological distress.

**Methods:**

A two-round online Delphi study was conducted. This study invited both qualitative and quantitative data from experts recruited via a scoping literature search and social media channels.

**Results:**

In total, 185 experts were invited to participate in this Delphi study. Of all participants invited to contribute, 35.7% (66/185) completed Round 1 and of those who participated in this first round, 67% (44/66) continued to complete Round 2. Out of 39 questions posed over two rounds, 18 statements (46%) achieved consensus, 21 (54%) did not. Participants were given the opportunity to write any additional comments as free text. In total, 1604 free text responses were collected and categorized into 2446 separate statements of opinion, creating a total of 442 themes. Overall, participants agreed that in order to effectively support midwives in work-related psychological distress, online interventions should make confidentiality and anonymity a high priority, along with 24-hour mobile access, effective moderation, an online discussion forum, and additional legal, educational, and therapeutic components. It was also agreed that midwives should be offered a simple user assessment to identify those people deemed to be at risk of either causing harm to others or experiencing harm themselves, and direct them to appropriate support.

**Conclusions:**

This study has identified priorities for the development of online interventions to effectively support midwives in work-related psychological distress. The impact of any future intervention of this type will be optimized by utilizing these findings in the development process.

## Introduction

Midwives can experience both occupational and organizational sources of psychological distress [1]. The well-being of health care professionals can be directly correlated with the safety and quality of patient care [2]. Therefore, in order to ensure high quality maternity care, psychological distress experienced by the midwifery profession will need to be met with appropriate and effective support. Although there is record of some support available for midwives, there currently appears to be a lack of online support available for midwives [3,4].

A recent review on maternity services has highlighted that midwives are more likely to report episodes of work-related stress than other health care professionals [5]. Yet there has been a reluctance to report episodes of unsafe practice or “impairment” due to a fear of adverse consequences [6]. Midwives are exposed to a variety of events which they perceive to be traumatic, and demonstrate a reluctance to seek support for fear of stigma and punitive responses when engaging with face-to-face support [7-9]. In line with other support provisions offered to physicians, midwives may also benefit from customized support, away from other health service users, among other midwives [10,11]. As such, midwives may be more likely to engage with an online intervention, which can facilitate the provision of confidentiality and anonymity and so encourage positive help-seeking behaviors and disclosure.

Generally, online interventions offer unique benefits such as greater accessibility, anonymity, convenience, and cost-effectiveness [12]. These benefits may appeal to midwives who often work long shifts during unsociable hours in an area of high litigation, where speaking openly about their ability to cope may prove to be challenging [13-15]. In light of the stigma surrounding nondisclosure in midwives requiring further support, and for those needing to disclose episodes of psychological distress or impairment, the provisions of confidentiality and anonymity may be essential for midwives to speak openly. In providing both anonymity and confidentiality within an online intervention, users will become unidentifiable and therefore cannot be held to account. This situation would result in a subsequent and inevitable amnesty. We refer to the concept of amnesty in this case as a period of forgiveness, where an episode of misconduct is pardoned for the purpose of enabling those in need of help to take a unique window of opportunity to seek help, where they may not otherwise have done so. With this in effect, immediate accountability will not be possible, and the immediate protection of the public may be unattainable. As it may be unfeasible for midwives to engage with face-to-face support and make open disclosures otherwise, it is vital that we consult with both midwives and others to explore the priorities for online interventions that support midwives in work-related psychological distress.

It is not currently known what should be prioritized in any online intervention, designed to effectively support midwives in work-related psychological distress. This paper reports the results of an online Delphi study designed to achieve consensus in the development of an online intervention to support midwives in work-related psychological distress.

The Delphi method was chosen due to its ability to stimulate anonymous discussion and erase any geographical distances between participants [16]. This online technique also protects the collaborative discussion from any one person dominating the conversation or governing the group’s thoughts and ideas [17].

## Methods

### Design

We conducted a two-round Delphi study between the 9^th^of September and the 30^th^of November 2015. Both rounds were completed online using Bristol Online Survey software and participants received feedback following both rounds electronically via blind carbon copied emails. Our study protocol has been published elsewhere [18]. The aim of this study was to achieve consensus in the design and delivery of an online intervention designed to support midwives in work-related psychological distress.

In total, 39 questions about what should be prioritized in the development of an online intervention for midwives were posed to eligible participants over two rounds. Questions were posed as statements for the expert panel to respond to, and were chosen in response to a scoping review of the academic and grey literature, and the lived experience of working within maternity services. This literature review was broad in scope, and included a combination of search terms relating to midwives, work-related psychological distress and online support interventions. A snowballing of the literature then led the research team to identify further themes of relevance [19]. Final themes were categorized within the online survey as; ethical inclusions, inclusions of therapeutic support and intervention design and practical inclusions.

Consensus was defined as a minimum of 60% of panelists responding within two adjacent points on the 7-point rating scale. This scale was anchored at “Not a priority” and “Essential priority”. Any item could reach consensus at any point within the scale, whether at the higher or lower end of the scale. The presence of consensus in this study was specified in advance of data collection.

Ethical approval for this study has been granted by Coventry University Ethics Committee (project reference ID P35069).

### Recruitment

Participant recruitment for panelists began in the September of 2015. Key papers which related to the subjects of midwifery work, psychological distress, online interventions and interventions designed to support mental well-being already known by the research team were screened for potential subject experts. A snowballing of the literature led the research team to scan reference lists and identify further key papers of relevance [19]. The authors of these papers were then invited to participate in the study.

### Inclusion Criteria

Participants were eligible to participate if they possessed all or some of the following practical knowledge in either: midwifery, midwifery education, research, therapies, health care services, staff experience or patient experience. Participants were also eligible if they had been listed as an author in at least one academic paper relevant to midwifery, psychological trauma, psychology, psychiatry or health care services. No exclusion criterion was applied.

### Online Recruitment Strategy

A social media recruitment drive was also conducted, in line with the study protocol [18]. Our strategy aimed to reach a range of midwifery professionals, those with a knowledge of psychology and psychological trauma, those with a background in psychiatry and/or practitioner health, patient and staff groups, those with a knowledge of risk, quality and safety in the health services and experts in the field of online interventions. In total, 185 people were invited to participate in the study. Some were contacted directly via email by the research team, and others contacted the research team expressing their interest in participation. All potential participants were invited to visit the research recruitment blog page, which detailed the study protocol and inclusion criteria [20].

During the study the research recruitment blog page was accessed 422 times. This blog page was also shared on Facebook 59 times, Linkedin 3 times, and Twitter 47 times. Additionally, the blog page was shared to a further 236 unidentified websites by its readership. The destination of a further 77 shares via social media remain unknown. An overview of social media engagement and the recruitment process are detailed within Multimedia Appendix 1.

Although participants remained anonymous throughout this study, some participants were keen to disclose their specific expert status to the research team. The team did not seek to verify the eligibility of each participant, and they simply consented to having the relevant expertise. The majority of participants who disclosed their expert status were either clinical and/or academic midwives. Other participants included psychiatrists, psychologists, health care, policy and midwifery leaders, and academic experts in the field of post-traumatic stress disorder (PTSD), secondary trauma and psychological distress. Some experts also disclosed their country of origin as the United Kingdom, the United States of America, Australia, Nigeria, Israel, and Oman. However, the locations of each individual participant are unknown.

### Round 1

Round 1 comprised a list of 20 statements relevant to the design and delivery of an online intervention to support midwives in work-related psychological distress. Participants were asked to choose a number that best represented their response to each statement with a 7-point Likert response scale. Two questions were given for each statement: “Why did you choose this rating of priority?”, followed by: “Do you have any additional comments you would like to share?” Space for free text responses was provided after each question.

### Round 2

All panelists received feedback on the panels’ responses to Round 1. The participant report delivered to participants following Round 1 can be found in Multimedia Appendix 2. Statements that did not achieve consensus in Round 1 were returned to participants in Round 2. In addition, 10 new statements were included in Round 2 on the basis of participant comments in Round 1 that were not reflected by the content of an existing statement. All statements are presented initially within Multimedia Appendix 2 and Multimedia Appendix 3.

All panelists who had participated in Round 2 were sent a summary of the outcome of Round 2, which can be found in Multimedia Appendix 3. Open text responses were coded by the primary researcher and then assigned to emergent themes in a succession of refinements. The themes and categorizations of statements were then revised and refined following an inspection and reflective discussion with the second researcher. The authorship of statements remained unknown to the researchers throughout.

## Results

### Consensus

**Figure 1:**
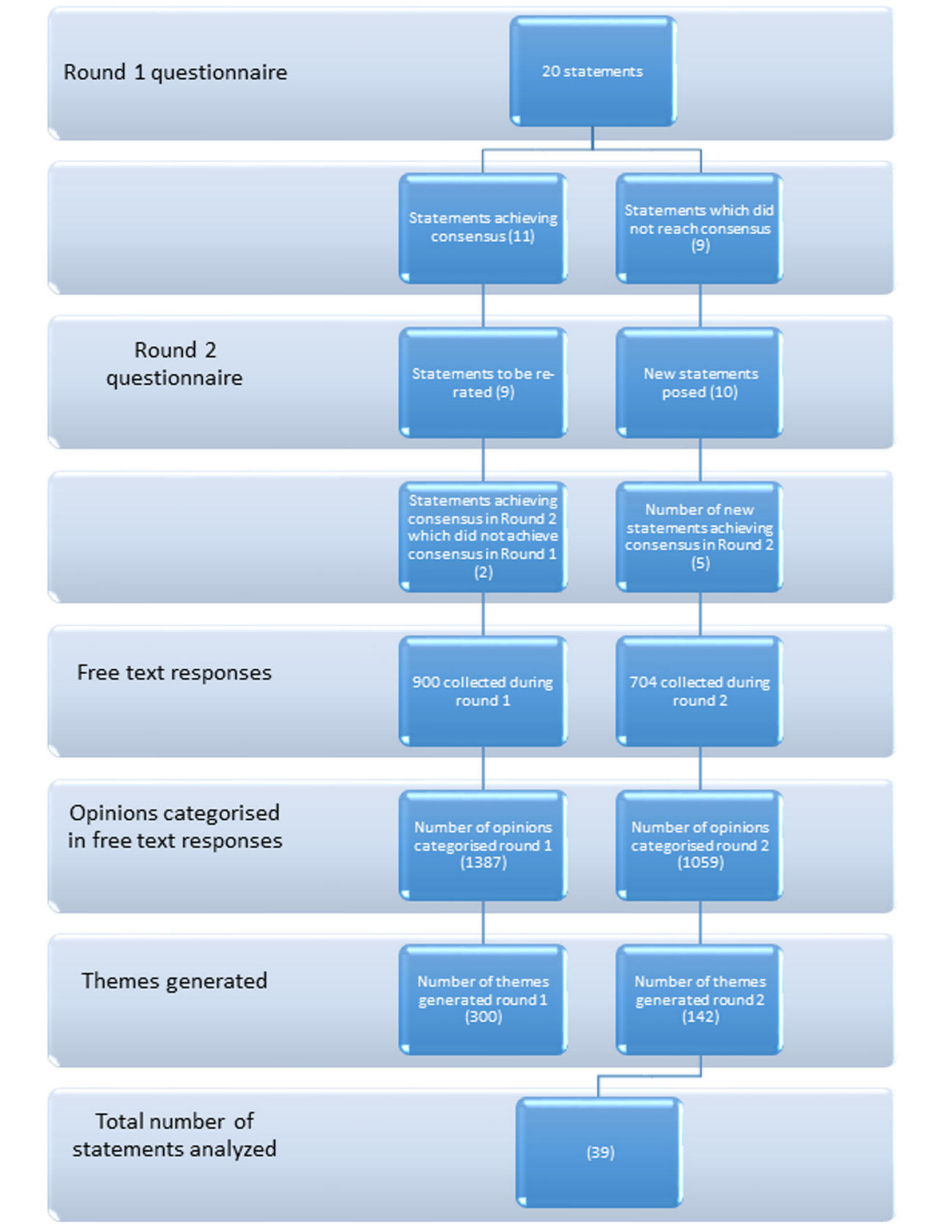
Number of Round 1 and 2 opinions, themes and statements achieving consensus.

Numerical data is reported in line with the outputs generated by the Bristol online survey software. Of people who were invited to participate in the study 35.7% (66/185) completed Round 1, and 67% (44/66) of those who contributed to Round 1 completed Round 2. Of the 20 statements posed during Round 1, 11 statements achieved consensus and 9 did not. Of the 19 questions posed within Round 2, 7 statements achieved consensus and 12 did not, giving a total of 18 consensus statements from the 30 statements posed to panelists. In total, 1604 free text responses were collected and categorized into 2446 separate statements. One free text response was removed in order to maintain confidentiality. An overview of results is presented in Figure 1. A detailed summary of the results for Rounds 1 and 2 are presented in Tables 1 and 2 respectively.

**Table 1:** Detailed summary of numeric results for Round 1.

Statement		Consensus achieved	% of consensus	Minimum score	Maximum score
**Ethical inclusions**									
	Confidentiality for all platform users and service users in all matters of discussion	Yes (high/essential priority)	90.90%	Not a priority/low priority 0/66 (0%)	Essential priority 54/66 (82%)
	Anonymity for all platform users and service users in all matters of discussion	Yes (high priority)	84.90%	Not a priority/low priority 0/66 (0%)	Essential priority 39/66 (59%)
	Amnesty for all platform users in that they will not be referred to any law enforcement agencies, their employer or regulatory body for either disciplinary or investigative proceedings in any case	No	N/A	Low/somewhat a priority 3/66 (5%)	Essential priority 22/66 (33%)
	Prompting platform users automatically to remind them of their responsibilities to their professional codes of conduct.	No	N/A	Somewhat a priority 0/66 (0%)	Essential priority 18/66 (27%)
	Prompting platform users automatically to seek help, by signposting them to appropriate support	Yes (high/essential priority)	78.80%	Not a priority/low priority/somewhat a priority 0/66 (0%)	Essential priority 31/66 (47%)
**Inclusions of Therapeutic Support**									
	The inclusion of Web-based videos, multimedia resources, and tutorials which explore topics around psychological distress	Yes (moderate/high priority)	68.20%	Not a priority/low priority/somewhat a priority 1/66 (2%)	High priority 27/66 (41%)
	The inclusion of informative multimedia designed to assist midwives to recognize the signs and symptoms of psychological distress	Yes (high/essential priority)	71.30%	Somewhat a priority 0/66 (0%)	High priority 26/66 (39%)
	The inclusion of multimedia resources which disseminate self-care techniques	Yes (high/essential priority)	74.20%	Low priority 0/66 (0%)	High Priority 29/66 (44%)
	The inclusion of multimedia resources which disseminate relaxation techniques	Yes (moderate/high priority)	65.10%	Not a priority/low priority/somewhat a priority 1/66 (2%)	Moderate priority 23/66 (35%)
	The inclusion of mindfulness tutorials and multimedia resources	Yes (moderate/high priority)	66.70%	Low priority 0/66 (0%)	High priority 27/66 (41%)
	The inclusion of Cognitive Behavioral Therapy (CBT) tutorials and multimedia resources	Yes (moderate/high priority)	60.60%	Somewhat a priority 0/66 (0%)	Moderate Priority 22/66 (33%)
	The inclusion of information designed to inform midwives where they can access alternative help and support	Yes (high/essential priority)	86.40%	Not a priority/low priority/somewhat a priority 0/66 (0%)	Essential priority 31/66 (47%)
	The inclusion of information designed to inform midwives as to where they can access legal help and advice	No	N/A	Not a priority/low priority/somewhat a priority 1/66 (2%)	Essential Priority 24/66 (36%)
	Giving platform users the ability to share extended personal experiences for other platform users to read	No	N/A	Not a priority 1/66 (2%)	Moderate priority 17/66 (26%)
	The inclusion of a Web-based peer-to-peer discussion chat room	No	N/A	Somewhat a priority 2/66 (3%)	High Priority 20/66 (30%)
	Giving platform users the ability to communicate any work or home-based subjects of distress	No	N/A	Somewhat a priority 1/66 (2%)	Moderate priority/high priority 16/66 (24%)
**Intervention design and practical inclusions**									
	An interface which does not resemble NHS, employer or other generic health care platforms	No	N/A	Low priority/somewhat a priority 2/66 (3%)	Essential priority 18/66 (27%)
	A simple, anonymized email log-in procedure which allows for continued contact and reminders which may prompt further platform usage	No	N/A	Low priority 1/66 (2%)	Moderate priority 20/66 (30%)
	An automated moderating system where “key words” would automatically initiate a moderated response	No	N/A	Not a priority/low priority 3/66 (5%)	Neutral 21/66 (32%)
	Mobile device compatibility for platform users	Yes (high/essential priority)	71.20%	Low priority/somewhat a priority 0 (0%)	Essential priority 27/66 (41%)

**Table 2:** Detailed summary of numeric results for Round 2.

Statement		Consensus achieved	% of consensus	Minimum score	Maximum score
**Ethical inclusions**					
	Amnesty for all platform users in that they will not be referred to any law enforcement agencies, their employer or regulatory body for either disciplinary or investigative proceedings in any case	No	N/A	Not a priority 2/44 (5%)	High priority 9/44 (21%)
	Prompting platform users automatically to remind them of their responsibilities to their professional codes of conduct	No	N/A	Somewhat a priority 2/44 (5%)	High priority 9/44 (21%)
**Inclusions of therapeutic support**					
	The inclusion of information designed to inform midwives as to where they can access legal help and advice	Yes (high/essential Priority)	65.90%	Not a priority 0/44 (0%)	High priority 17/44 (39%)
	Giving platform users the ability to share extended personal experiences for other platform users to read	No	N/A	Not a priority 0/44 (0%)	High priority 11/44 (25%)
	The inclusion of a Web-based peer-to-peer discussion chat room	Yes (moderate/high priority)	63.60%	Not a priority 1/44 (2%)	Moderate priority 15/44 (34%)
	Giving platform users the ability to communicate any work or home-based subjects of distress	No	N/A	Not a priority 1/44 (2%)	Moderate/essential priority 11/44 (25%)
**Intervention design and practical inclusions**					
	An interface which does not resemble NHS, employer or other generic health care platforms	No	N/A	Not a priority 1/44 (2%)	Essential priority 13/44 (30%)
	A simple, anonymized email log-in procedure which allows for continued contact and reminders which may prompt further platform usage	No	N/A	Not a priority/low Priority 0/44 (0%)	High priority 14/44 (32%)
	An automated moderating system where “key words” would automatically initiate a moderated response	No	N/A	Low priority 2/44 (5%)	Neutral 13/44 (30%)
**New items for consideration**					
	An interface which resembles and works in a similar way to current popular and fast pace social media channels (eg, Facebook)	No	N/A	Not a priority 0/44 (0%)	Neutral 12/44 (27%)
	The inclusion of midwives from around the world	No	N/A	Not a priority 3/44 (7%)	Moderate priority 11/44 (25%)
	Proactive moderation (ie, users are able to block unwanted content and online postings are “pre-approved”)	Yes (high/essential priority)	61.40%	Not a priority 1/44 (2%)	High priority 15/44 (34%)
	Reactive moderation (ie, users are able to report inappropriate content to a system moderator for removal)	Yes (high/essential priority)	70.50%	Not a priority 1/44 (2%)	High priority 16/44 (36%)
	24/7 availability of the platform	Yes (high/essential priority)	84.10%	Not a priority/low priority 0/44 (0%)	Essential priority 25/44 (57%)
	The implementation of an initial simple user assessment using a psychological distress scale to prompt the user to access the most suitable support available	Yes (moderate/high priority)	70.40%	Not a priority/somewhat priority 1/44 (2%)	High priority 25/44 (39%)
	The gathering of anonymized data and concerns from users, only with explicit permission, so that trends and concerns may be highlighted at a national level.	No	N/A	Not/low/somewhat a priority 2/44 (5%)	Essential priority 15/44 (34%)
	Access for a midwife's friends and family members	No	N/A	Essential priority 0/44 (0%)	Not a priority 17/44 (39%)
	The follow up and identification of those at risk	Yes (high/essential priority)	63.70%	Low/somewhat a priority 1/44 (2%)	Essential priority 16/44 (36%)
	The provision of a general statement about professional codes of conduct and the need for users to keep in mind their responsibilities in relation to them	No	N/A	Not a priority 1/44 (2%)	Essential priority 12/44 (27%)

### Thematic Analysis

#### Themes

Full details of the number of statements recorded in each theme are given in Multimedia Appendix 4. Below we describe the thematic analysis, presented by statement type. More detailed reports of the thematic analysis of Round 1 and 2 can be found in Multimedia Appendix 5 and Multimedia Appendix 6, respectively.

#### Ethical Inclusions

Confidentiality and anonymity were both considered to be an essential priority, with one participant describing how “some midwives would be fearful of people finding out they were finding it difficult to cope and would therefore seek anonymity to feel safe to access support” and another revealing how “anonymity would enable honesty and a true space to unburden” as “a confidential forum allows discussion to take place without feeling judged”. However, the corollary to confidentiality and anonymity, amnesty, is a source of tension, both within some participants who are ambivalent about amnesty and between participants with different perspectives.

Panelists remained largely conflicted in opinion about the provision of amnesty. Consequently, consensus was not achieved for the statement regarding amnesty in either Round 1 or Round 2. One comment illustrates this conflict well: “amnesty is an ethical issue, particularly relating to criminal matters; however, without it midwives may not feel able to disclose their concerns causing distress”. Polarized views were also apparent, as one comment suggests that “people are not going to be fully revealing if they believe they will suffer as a result!” and another participant expressed concern that this statement “almost suggests that there may be grounds for this route to be considered”. Finally, one participant commented that “unless amnesty is assured confidentiality/anonymity won't be maintained”.

Opinion remained divided throughout both rounds of questioning about whether an online intervention designed to support midwives should remind users of their professional codes of conduct. Similarly, experts did not agree about whether the provision of a general statement about professional codes of conduct and the need for users to keep in mind their responsibilities in relation to them should be prioritized or not. Although participants expressed a loyalty to their professional codes of conduct, they also conveyed concerns about whether this may deter midwives from speaking openly and/or seeking help. There was also some concern that reminders about codes of conduct may be seen as condescending. Experts were unable to agree upon whether this would inhibit the functionality of effective support or should be provided to reinforce the professional responsibilities of the midwife.

In terms of opening the online intervention up to global midwifery populations, many experts highlighted the challenges in relating to the various cultural and contextual differences across the globe. However, many acknowledged the need for midwifery support all over the world. Equally, when panelists were asked to consider whether an online intervention designed to support midwives in work-related psychological distress should prioritize access for a midwife's friends and family members, a consensus of opinion could not be reached. In this case, experts highlighted that midwives may lose their anonymity if friends and family members were permitted access to the intervention. Many open text responses expressed the need to prioritize access to the intervention for midwives only. One in particular summarizes that “while family and friends provide important support, the needs of the midwife should remain paramount.”

Experts expressed a need to prioritize the implementation of an initial simple user assessment using a psychological distress scale to prompt the user to access the most suitable support available. This was largely “as individuals may not realize that they are in psychological distress “or “don't recognize the signs and symptoms of stress, PTSD, depression or anxiety”. However, many remained unsure about what may trigger a response, how the user may be prompted, and what support may then be offered. Additionally, experts stated that midwives may feel uncomfortable with this level of screening. This point was also one of the reasons given by panelists reluctant to prioritize the gathering of anonymized data and concerns from users, even with explicit permission. Where many experts saw the benefits of capturing national trends, with one comment summarizing that it may be “critical that trends are identified and strategies developed to address those trends at a national level”, others were wary that if this was the case, midwives may be reluctant to engage.

Experts agreed that the intervention should prioritize the follow-up and identification of those at risk. However, there were requests to clarify the definition of what may classify someone as being “at risk”. Some panel members suggested that “if suicidal behavior is conveyed through the postings” or if there is “talk of harming someone”, those individuals may be identified as being “at risk”. Yet many open text responses illuminated the difficulties in following up anonymous users. Some experts were also unsure about how this particular component may be facilitated. Additionally, others purported that this should not be the responsibility or purpose of this particular online platform.

The expert panel concurred that midwives using the platform should be automatically prompted to seek help, by signposting them to appropriate support. However, some panelists questioned how this may be organized, what types of support may be on offer, and whether or not this provision may encourage users to pathologize normal reactions to certain types of events.

#### Therapeutic Support

In terms of the nature of the support within an online intervention to support midwives, the expert panel agreed that priorities include Web-based videos, multimedia resources and tutorials which explore psychological distress and assist midwives to recognize the signs and symptoms of psychological distress. One comment which illustrates a widely held belief was that “midwives often feel guilty for catching up on sleep, having time out watching TV, gently exercising with friends etc.” As such, it was also agreed that an online intervention should prioritize resources which disseminate self-care techniques and Cognitive Behavioral Therapy (CBT) tutorials through a range of online media sources. Largely, it was inferred that this online intervention should establish itself as a “one stop shop”.

Expert participants also agreed that midwives in distress should be offered information designed to inform them where they can access alternative help and support. The most frequent reason given for this was the need for provision of choice. Equally, there was consensus that an online intervention should prioritize the inclusion of information to inform midwives about where they can access legal help and advice. During Round 1, participants noted that midwives could already find this information from trade unions such as the Royal College of Midwives (RCM), and may be further distressed by the thought of needing legal assistance. Yet one comment in particular highlighted the notion that “we live in a litigious and unforgiving world”. However, during Round 2, experts noted that midwives may need a wider range of legal information available to them in order to be prepared should a need arise. One comment illustrated this by reiterating that “any help and advice is welcome”.

When expert panelists were asked whether an online intervention to support midwives should prioritize giving users the ability to share extended personal experiences for other platform users to read, no consensus of opinion was reached. Open text responses gravitated towards concerns relating to breaches in confidentiality, risk of misuse, and the need for active moderation. However, a number of responses highlighted the potential cathartic and therapeutic benefits of both reading and writing personal experiences, providing opportunities for reflection, sharing, learning, and fellow feeling with others.

Although experts did not agree to prioritize the inclusion of a Web-based peer-to-peer discussion chat room during Round 1, within Round 2 this item became a moderate to high priority inclusion. While many experts expressed a need for the appropriate moderation of an online chat room, the benefits of peer-based discussion were highlighted as a key component of support. One comment summarizes these thoughts by stating that “sharing experiences and getting feedback from peers who have experienced similar situations is very helpful”. More significantly, it was also highlighted that this chat room “would require high volume site traffic to be viable and sustainable”. When asked about topics of discussion within the chat room, experts did not reach a consensus as to whether the chat room should give users the ability to communicate any work or home-based subjects of distress. However, these two subjects were seen as being intertwined.

#### Intervention Design and Practical Inclusions

Regarding the aesthetics of the online intervention, opinions remained divided about whether the intervention should resemble any National Health Service (NHS), employer or other generic health care platforms. Although the panel acknowledged that the intervention should look trusted, professional and official, they were also wary that should the intervention resemble an official health care organization, midwives may feel unable to speak openly. One particular comment defines opinion in that “any resemblance to NHS etc.… could deter people from using the platform”, however, this same panelist also felt that the intervention “needs to resemble a clean professional image”. Additionally, panelists remained divided in opinion and wary of an anonymized email log-in procedure which allows for continued contact and reminders which may prompt further platform usage. Although experts favored the use of anonymity in log-in procedures, some felt that prompting use may cause further distress.

In terms of accessibility and ease of use, experts agreed that making the intervention available to midwives in work-related psychological distress 24 hours a day and via mobile access should be made high to essential priorities. However, experts did not agree upon whether an online intervention to support midwives in work-related psychological distress should prioritize an interface which resembles and works in a similar way to current popular and fast-paced social media channels (eg, Facebook). In this case, many free text responses alluded to the fact that Facebook and other social media channels are perceived as risky to use by midwives. Nevertheless, many other comments suggested that emulating the familiarity of a known platform may promote an inherent ability for midwives to engage with the intervention more sinuously. Ultimately, one particular comment summarizes that “ease of use and familiarity for most users will encourage engagement”.

The importance of effective moderation remained a recurrent theme throughout this study. Experts agreed that both proactive moderation (ie, users are able to block unwanted content and online postings are “pre-approved”) and reactive moderation (ie, users are able to report inappropriate content to a system moderator for removal) should be made high to essential priorities. One comment in particular highlights one recurring theme in that “the platform needs to be regulated to avoid inappropriate posts and language”.

Other interventions of this nature have employed an automated moderating system where “key words” would automatically initiate a moderated response. However, this group of experts remained divided about whether this should be prioritized in an online intervention to support midwives. Many panelists cited the importance of regulation; however, some were unsure about how this particular provision may work in the real world. Additionally, fears were raised that this provision may make the intervention seem impersonal. Overall, it was the principal judgment of this group that, easy 24-hour mobile access and “an easy log-in and easy to use interface couldn't be more essential”.

### Summary of Results

Out of 39 questions posed over two rounds, 18 statements (46%) achieved consensus, 21 (54%) did not. Provisions that were endorsed tended to favor those which enabled knowledge acquisition, ease of use, ongoing support, skill development, and human interaction. The highest priority scores were given to the provisions of anonymity (84.9%) and confidentiality (90.9%). For those items which achieved consensus, the lowest priority scores were given to the provisions of CBT resources (60.6%) and proactive moderation (61.4%). Overall, the expert panel agreed that each statement should be made at least a moderate priority.

Overall, open text responses demonstrated both interest and enthusiasm for the development of an online intervention to support midwives in work-related psychological distress. However, some provisions were favored over others, and in some cases, when invited to engage in moral decision making participants were polarized and conflicted in opinion.

## Discussion

### Principal Findings

This Delphi study has extracted the priorities, associated underlying beliefs and opinions of a panel of experts regarding the delivery of an online intervention to support midwives in work-related psychological distress. The expert panel in this case identified 18 statements to be prioritized by those seeking to design and deliver an online intervention to support midwives. This is the first study of this type to identify these matters of salience. Additionally, the thematic analysis of free text responses offered by the panel illuminates the ethical, moral, and practical challenges involved in the design and delivery of an effective online intervention to support midwives.

Overall, the recurring themes explored by this study were the reluctance of midwives to speak openly and/or seek help for the fear of retribution, the need for both anonymity and confidentiality at all times, ease of use, effective moderation and the necessity to help and support midwives in work-related psychological distress. Challenges remain in complex ethical, legal, and moral decision making in facilitating effective online support provision for midwives in distress.

### Interpretation of Findings

Interestingly, based on quantitative and qualitative responses, participants in this study do not readily differentiate between confidentiality and anonymity in this particular context. Their reasons or justifications for the requirement to have both confidentiality and anonymity are generally very similar. Ethicists and intervention developers may differentiate between these two concepts but this group does not. There is no meaningful difference between confidentiality and anonymity for this stakeholder group, which largely comprises the potential end users of the online resource.

When both confidentiality and anonymity are in place, their corollary, amnesty becomes apparent. Many of the expert panel members cited that midwives would not speak openly for the fear of stigma and retribution. Indeed, these findings have been verified within other studies where midwives reported stigma, and a perceived punitive response to face-to-face discussions concerning work-related traumas [6,7,9,21]. As such, many of the expert panel members saw amnesty as an essential provision in supporting midwives to seek help. Other panel members were opposed to the provision of amnesty, either because they feared that this would be in direct conflict with moral or professional duties and obligations, or because they favored immediate accountability for the direct protection of the public and patients. A number of panel members recognized both sides of this argument, and were therefore unable to decide their position in this case. This moral conflict is reflected in the many confidential health practitioner services that exist for doctors in distress [22-24]. In these cases, the public recognize the value in offering impaired physicians’ identity protection for the purpose of remediation, yet they also call for open reporting where risks to patients and the public are identified within the public sphere.

The primary concern for those who are ambivalent or who are opposed to amnesty was the risk of harm to third parties by midwives; both preventing future harm and accountability for harm that has already occurred. Satisfying this concern will be essential for the acceptance of an online resource for midwives experiencing psychological distress. One element of negotiation may be to encourage those in distress to self-disclose episodes of impairment with the support of the online community. This idea is supported by one free text response which purports that “ideally an online platform should encourage the professional themselves to take action if appropriate”. This outcome could result in more midwives coming forward in help-seeking, for the benefit of maternity services as a whole.

It is clear that this expert group feels that a range of multimedia resources in relation to help-seeking, diagnostic criteria, therapeutic, and practical inclusions should be prioritized in the development of an online intervention to support midwives. Future developments should consider becoming a “one stop shop” for midwives in relation to this finding. Going further, it may be prudent to develop online interventions with the functionality to incorporate a range of midwifery populations, global health care workforces, and other groups of clinical professionals as a prospective future growth model evolves. This concept is also supported by an expert response, suggesting that “in developing this platform for a specific group of midwives, a future goal may be to adapt it for other specific groups once this project is functioning and any difficulties have been eliminated”.

In developing an effective online intervention to support midwives in work-related psychological distress, the practicalities of galvanizing a large user base, evolving a robust system of moderation and rousing the support of professional and regulatory bodies will be vital in securing its sustainability. Gaining the trust of midwives in distress and engaging them in using a safe online intervention may enable this one solution to flourish and improve the health of midwives, which crucially may increase protection for the public, secure the long term health of midwives, and increase safety for maternity services. This study will be integral to the development process of any online intervention designed to support midwives, as the application of this data to the development process optimizes the likelihood of accomplishing an efficacious intervention overall.

### Strengths and Limitations

The research team invited experts in the subject areas of both e-mental health and m-health via the academic emails provided in recently published research papers to participate within this study. We also invited midwives, psychologists, psychiatrists, other physicians, and academic experts to take part. While this Delphi study has harnessed the opinions of a diverse group of experts on a practice-related problem, we are unable to verify the expert status of all participants due to the provision of participant anonymity. Therefore, some fields of expertise may not have been reflected in the data.

Although we acknowledge that the decision to allow respondents to be completely anonymous in a Delphi study is an unusual one, we feared that participants would feel unable to be completely open and honest without the provision of anonymity in place. As such, this course of action has undoubtedly impacted upon the confirmation of the participants’ expertise, especially as the expertise of participants was not confirmed by the research team, leaving participants merely to consent to having the relevant expertise.

Additionally, and unlike many Delphi studies, the feedback provided after each round did not include each participant’s own previous response. This was again due to the provision of anonymity afforded to participants. Therefore, Participants were unable to compare their own response to the groups’ response. We also note that there has been a significant participant dropout rate between the two rounds. Therefore, the change in item endorsement may have been influenced by the different participants that remained in the study. This is a limitation of this study, but one that is not possible to explore.

Though our response rates may be deemed relatively low (35.7% and 67% respectively), these response rates are similar to those found in other Delphi studies [25,26]. Additionally, the Delphi technique relies on the opinions of those recruited, yet its methodology requires empirical measures to determine consensus. Therefore, the presence of consensus in this study has been determined empirically and was specified in advance of data collection.

Our literature searches to both identify salient themes and recruit expert panel members were broad. As such, our searches may have failed to identify some key papers of relevance and potential expert panel members. Our search terms were led by a process of snowballing, where the research team responded to emerging themes and findings [19]. We recognize that these searches may not have captured all of the key literature relating to the characteristics which may be salient in supporting midwives online.

### Conclusions

This paper has reported the results of a two-round Delphi study to achieve consensus about the key features of online interventions to support midwives in work-related psychological distress. This study provides an account of some key priorities for the development of such interventions; although some practical, ethical, and moral challenges remain unresolved.

In pursuit of excellence in maternity services, future research has the opportunity to explore the provision that might best support midwives in psychological distress. Future studies could use this information to turn the vision of online support for midwives in distress into practice.

## Appendix

Multimedia Appendix 1 Social media engagement and recruitment summary.

Multimedia Appendix 2 Participant report – Round 1.

Multimedia Appendix 3 Participant report – Round 2.

Multimedia Appendix 4 Results of thematic analysis.

Multimedia Appendix 5 Analysis report – Round 1.

Multimedia Appendix 6 Analysis report – Round 2.

## Abbreviations

CBT: Cognitive Behavioral Therapy
NHS: National Health Service
PTSD: Post-Traumatic Stress Disorder
RCM: Royal College of Midwives
