# Supplementary figures and images for: Achieving Consensus for the Design and Delivery of an Online Intervention to Support Midwives in Work-Related Psychological Distress: Results From a Delphi Study
Source: JMIR Ment Health. 2016 Jul 12;3(3):e32. doi: 10.2196/mental.5617 (PMC4961877; doi:10.2196/mental.5617)

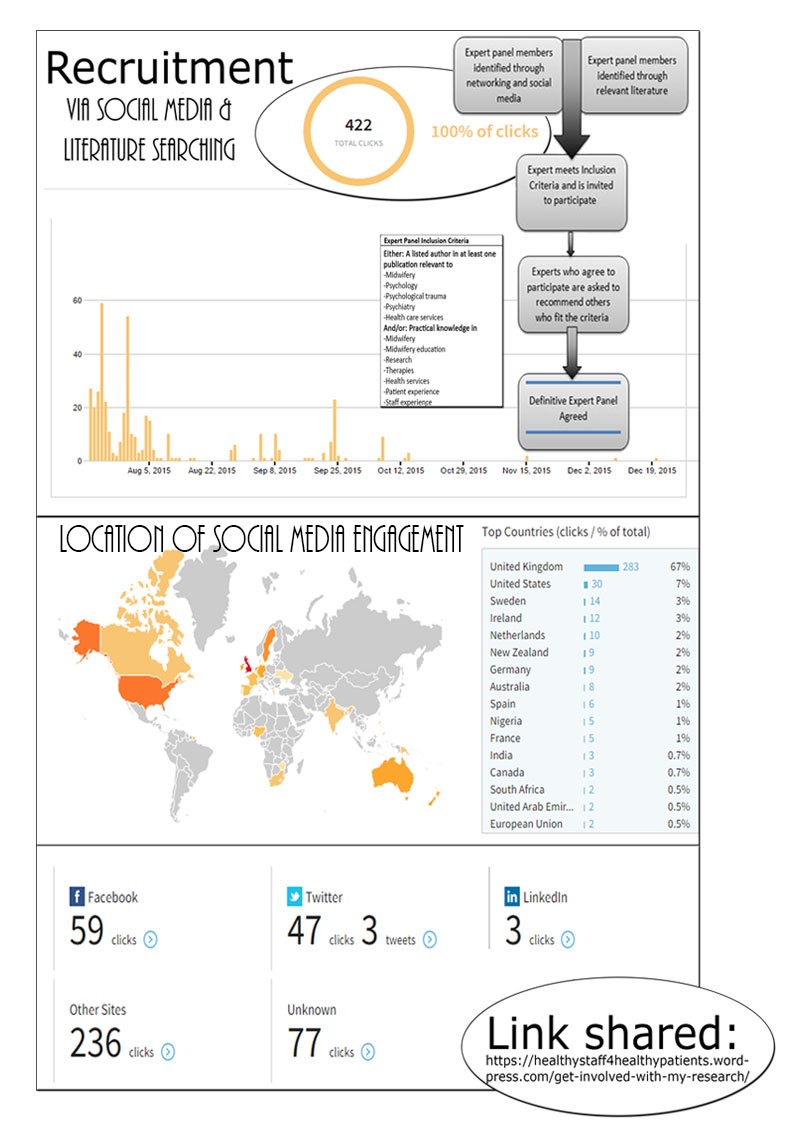

Supplement: Multimedia Appendix 1 [file mental_v3i3e32_app1.jpg]
