# Supplementary material for: Achieving Consensus for the Design and Delivery of an Online Intervention to Support Midwives in Work-Related Psychological Distress: Results From a Delphi Study
Source: JMIR Ment Health. 2016 Jul 12;3(3):e32. doi: 10.2196/mental.5617 (PMC4961877; doi:10.2196/mental.5617)
Supplement: Multimedia Appendix 2 [file mental_v3i3e32_app2.pdf]

Delphi Study First Round Participant Report: Delphi Study to achieve Consensus in the Development of an Online Intervention Designed to Effectively Support Midwives in Work related Psychological Distress.

Round One

Thankyou from the research team

66 of you completed this first round of questioning designed to achieve consensus in the development of an online intervention designed to support midwives in work-related psychological distress. Thankyou! Your responses were informative, useful and very valuable to this study.

Overview of Round One

A total of 185 people were invited to participate in this Delphi Study. 66 (35.7%) of those participants invited completed the first round. Panellists were asked to assign a priority rating to 20 statements using a 7 point likert type scale. Prior to receiving your responses we set a criteria for consensus. As such, a consensus of opinion was considered to be reached when 60% or more of the responses fell within 2 adjacent response points on the 7-point scale.

example	Not a priority	Low priority	Somewhat a priority	Neutral	Moderate priority	High priority	Essential priority
An interface which does not resemble NHS, employer or other generic healthcare platforms	☐	☐	☐	☐	☐	☐	☐

Summary of Round One

The research team assessed whether each item had achieved a consensus of opinion or not. The team then went on to allocate each free text response with one or more themes of significance. Of the 20 questions posed, 11 lines of enquiry achieved consensus and 9 did not. A total of 900 free text responses were provided by the panel which were then categorised into themes by two of the research team. Table one summarises the results from round one of this Delphi study.

Table 1: Results Summary for Round One Questionnaire

	**Question: An online intervention designed to support midwives in work-related psychological distress should prioritise:**	**Consensus achieved**	**% of Consensus**	**Mean Score**	**Comments submitted in free text**	**Statements Categorised**	**Themes Generated**
1.	Confidentiality for all platform users and service users in all matters of discussion	Yes (High/ Essential Priority)	90.9%	6.61	58	85	17
2.	Anonymity for all platform users and service users in all matters of discussion	Yes (High Priority)	84.9%	6.27	51	88	22
3.	Amnesty for all platform users in that they will not be referred to any law enforcement agencies, their employer or regulatory body for either disciplinary or investigative proceedings in any case.	No	N/A	5.08	59	136	19
4.	Prompting platform users automatically to remind them of their responsibilities to their professional codes of conduct.	No	N/A	4.91	63	81	12
5.	prompting platform users automatically to seek help, by signposting them to appropriate support	Yes (High priority/ Essential Priority)	78.8%	6.15	48	79	17
6.	the inclusion of web based videos, multimedia resources and tutorials	Yes (Moderate priority/ High	68.2%	5.41	51	81	11

	which explore topics around psychological distress	priority)					
7.	the inclusion of informative multimedia designed to assist midwives to recognise the signs and symptoms of psychological distress	Yes (High priority/ Essential priority)	71.3%	5.85	50	80	18
8.	The inclusion of multimedia resources which disseminate self-care techniques	Yes (High priority/ Essential priority)	74.2%	5.89	45	67	16
9.	The inclusion of multimedia resources which disseminate relaxation techniques	Yes (Moderate priority/ High priority)	65.1%	5.41	41	55	21
10	The inclusion of mindfulness tutorials and multimedia resources	Yes (Moderate priority /High priority)	66.7%	5.38	45	59	22
11	The inclusion of Cognitive behavioural Therapy (CBT) tutorials and multimedia resources	Yes (Moderate priority/ High priority)	60.6%	5.12	42	62	25
12	The inclusion of information designed to inform midwives where they can access alternative help and support	Yes (High priority/ Essential priority)	86.4%	6.32	30	41	12
13	The inclusion of information designed to inform midwives as to where they can access legal help and advice	No	N/A	5.7	32	40	9
14	Giving platform users the ability to share extended personal experiences for other platform users to read	No	N/A	5.02	49	87	12
15	The inclusion of a web based peer to peer discussion chat room	No	N/A	4.79	48	78	18

16	Giving platform users the ability to communicate any work or home based subjects of distress	No	N/A	5.12	38	50	10
17	An interface which does not resemble NHS, employer or other generic healthcare platforms	No	N/A	5.24	38	50	13
18	A simple, anonymised email login procedure which allows for continued contact and reminders which may prompt further platform usage	No	N/A	5.33	32	53	6
19	An automated moderating system where 'key words' would automatically initiate a moderated response	No	N/A	4.74	44	67	12
20	Mobile device compatibility for platform users	Yes (High Priority/ Essential Priority)	71.2%	5.97	36	48	8
					Total= 900	Total = 1387	Total =300

Table two summarises the responses given during round one of this Delphi study. Please note that percentages have been rounded toward the nearest whole number.

Table 2: Summary of round 1 responses

Topic of enquiry	Themes generated	Number of times referenced	%	Total number of statements categorised
Confidentiality for all platform users and service users in all matters of discussion	Confidentiality – Required for open and honest disclosure	24	28%	85
	Midwives - Fear retribution	6	7%	
	Confidentiality – Decided by user	5	6%	
	Confidentiality – for third parties	9	11%	
	Midwives - May need further support/ intervention	2	2%	
	Midwives – Feel shame if not managing	2	2%	
	Confidentiality – Needed to avoid public identification	3	4%	
	Confidentiality – Not possible online	2	2%	
	Confidentiality - Needed to protect the reputation of the profession	2	2%	

	Confidentiality – Context dependent	4	5%	
	Confidentiality – Essential criterion for provision of support	10	12%	
	Midwives – Need to feel safe	10	12%	
	Midwives – Have little existing provision	1	1%	
	Professional – Legal/Regulatory obligations	1	1%	
	Midwives – Fear consequences	1	1%	
	Confidentiality – High priority	2	2%	
	Midwives – Need reassurance	1	1%	

**Topic of enquiry**	**Themes generated**	**Number of times referenced**	**%**	**Total number of statements categorised**
Anonymity for all platform users and service users in all matters of discussion	Anonymity – Required for open and honest disclosure	20	23%	88
	Anonymity – May prevent further intervention	3	3%	
	Anonymity – Needed for support	10	11%	
	Anonymity - Decided by user	7	8%	
	Feeling safe/safety - Required	4	5%	
	Midwives – Fear retribution	6	7%	
	Midwives – Support is highest priority	4	5%	
	Anonymity – Not possible online	3	3%	
	Professional – Legal/Regulatory obligations	4	5%	
	Anonymity – for third parties	5	6%	
	Midwives – Need assurances	1	1%	
	Anonymity is synonymous with confidentiality	2	2%	
	Midwives – Feel shame if not managing	2	2%	
	Anonymity – Use of pseudonyms	2	2%	
	Anonymity - could be misused/cause distress	4	5%	
	Practicalities – Additional support may be required	2	2%	
	Anonymity – Requires policy	1	1%	
	Practicalities – Legal obligations over raising concerns	2	2%	
	Practicalities – User verification	1	1%	
	Midwives – Fearful of disclosure	1	1%	
	Anonymity – Needed to seek support	1	1%	
	Anonymity – Needed to feel safe	2	2%	
	Anonymity – Unsure of relevance	1	1%	

**Topic of enquiry**	**Themes generated**	**Number of times referenced**	**%**	**Total number of statements categorised**
Amnesty for all platform users in that they will not be referred to any law enforcement agencies, their employer or regulatory body for either disciplinary or investigative proceedings in any case.	Amnesty – Important/Helpful	19	14%	136
	Amnesty - Conflicted in opinion	13	10%	
	Amnesty - Cannot be supported	14	10%	
	Amnesty – Legal and ethical obligations – duty of care	18	13%	
	Amnesty – Required to facilitate support	13	10%	
	Amnesty – Automatic if confidentiality/anonymity is afforded	2	1%	
	Midwives – Fear speaking openly/retribution	15	11%	
	Practicalities – Intervention may be required	10	7%	
	Amnesty – may not be possible	9	7%	
	Midwives – Have little existing provision	1	1%	
	Midwives – Need support	1	1%	
	Midwives – Should self-report	4	3%	
	Intervention – Disclaimers may be required	1	1%	
	Practicalities – Further intervention by management required	2	1%	
	Amnesty – Required for recovery	5	4%	
	Midwife – Needs support	1	1%	
	Amnesty – May cause distress to others	3	2%	
	Intervention – Consider emulating the principles of	1	1%	

	comparable interventions.			
	Amnesty – Conflicted in opinion	3	2%	
	Intervention – Warnings may be required	1	1%	

Topic of enquiry	Themes generated	Number of times referenced	%	Total number of statements categorised
Prompting platform users automatically to remind them of their responsibilities to their professional codes of conduct.	Prompting - May be harmful	8	10%	81
	Midwives – Will already be aware	11	14%	
	Prompting – Should be done sensitively	6	7%	
	Prompting – Helpful inclusion	18	22%	
	Prompting – Unhelpful inclusion	14	17%	
	Professional codes – adherence a professional responsibility	14	17%	
	Prompting – Need unclear	3	4%	
	Conflicted opinion	2	2%	
	Prompts – Not supportive	2	2%	
	Prompts – adherence to code a pre-condition of use	1	1%	
	Prompts – Sensitivity needed	1	1%	
	Codes of conduct – important to highlight	1	1%	

Topic of enquiry	Themes generated	Number of times referenced	%	Total number of statements categorised
Prompting platform users automatically to seek help, by signposting them to appropriate support	Signposting to support – A useful inclusion	36	46%	79
	Prompts - unsuitable	2	3%	
	Practicalities – Dependent on the nature of support	4	5%	
	Practicalities – Needs a personalised tailored response	1	1%	
	Intervention – if evidence-based	1	1%	
	Safety is important	3	4%	
	Conflicted opinion	4	5%	
	Signposting to support – Clarity on method required	3	4%	
	Signposting to support – Help seeking may be low	9	11%	
	Signposting to support – Support must be high quality	2	3%	
	Midwives – In control of their own help seeking behaviours	3	4%	
	Signposting to support – A helpful inclusion	4	5%	
	Signposting to support - Could lead to users pathologising symptoms	1	1%	
	Signposting to support – intervention itself is sufficient	2	3%	
	Prompting - Consider alternative delivery	1	1%	
	Consider using third sector groups and organisations	1	1%	
	Automatic signposting - Clarity on method required	2	3%	

Topic of enquiry	Themes generated	Number of times referenced	%	Total number of statements categorised
the inclusion of web based videos, multimedia resources and tutorials which explore topics around psychological distress	Multimedia tutorials – Helpful inclusion	40	49%	81
	Multimedia resources – Unhelpful inclusion	6	7%	
	Multimedia – Variety in content presentation useful	18	22%	
	Multimedia resources – Evidence based/high quality resources required	3	4%	
	Multimedia resources – Conflicted opinion	4	5%	
	Multimedia resources – benefit dependent upon the nature of distress	1	1%	
	Midwives – Greater need for alternative support	3	4%	
	Multimedia resources – Benefit dependent upon the nature of resource	3	4%	
	Midwives – Feel like failures	1	1%	

	Midwives – Material needs to be matched to user needs	1	1%	
	Usability - depends upon the content	1	1%	
**Topic of enquiry**	**Themes generated**	**Number of times referenced**	**%**	**Total number of statements categorised**
The inclusion of informative multimedia designed to assist midwives to recognise the signs and symptoms of psychological distress	Informative Multimedia – Helpful inclusion	40	50%	80
	Informative Multimedia – Unhelpful inclusion	3	4%	
	Multimedia – Need a variety of resources	2	3%	
	Conflicted – Depends upon objectives/content	7	9%	
	Multimedia - Resources must be high quality/evidence based	1	1%	
	Multimedia - Needs to be unique	1	1%	
	Multimedia - Not required	2	3%	
	Midwives – Do not always recognise own distress	13	16%	
	Informative Multimedia - could lead to inappropriate self-diagnosis	3	4%	
	Midwives – Support is important	1	1%	
	Midwives – Help Seeking is important	2	3%	
	Organisational – Distress is a normal response to organisational issues	1	1%	
	Informative Multimedia – resource should be clear and simple.	1	1%	
	Informative multimedia – To be used in initial engagement	1	1%	
	Informative media – Requires a variety of options	1	1%	
	Midwives – Do not always recognise own distress	1	1%	
**Topic of enquiry**	**Themes generated**	**Number of times referenced**	**%**	**Total number of statements categorised**
The inclusion of multimedia resources which disseminate self-care techniques	Multimedia self-help resources – Helpful inclusion	30	45%	67
	Multimedia self-help resources – Unhelpful inclusion	1	1%	
	Need a variety of resources	6	9%	
	Multimedia self-help resources – Needs to be useful	3	4%	
	Midwives - do not prioritise self-care	5	7%	
	Midwives – additional support may be needed	6	9%	
	Multimedia self-help resources - ease of use important	3	4%	
	Neutral	2	3%	
	Midwives – Need support and understanding	2	3%	
	Midwives – Do not always recognise own distress	2	3%	
	Resource – Must be multiple options available	2	3%	
	Midwives – Need assessment	1	1%	
	Midwives – Must be accountable	1	1%	
	Midwives – Provision of coaching	1	1%	
	Midwives - Meaning of self-care unclear	1	1%	
	Resources - Peer support is useful	1	1%	
**Topic of enquiry**	**Themes generated**	**Number of times referenced**	**%**	**Total number of statements categorised**
The inclusion of multimedia resources which disseminate relaxation techniques	Relaxation techniques - A helpful inclusion	18	33%	55
	Relaxation techniques - An unhelpful inclusion	8	15%	
	Resources – Need a variety of options	4	7%	
	Relaxation is a self-care technique	2	4%	
	Resources - Must be easy to use	2	4%	
	Organisational - distress can have organisational cause	1	2%	
	Resources – Could emulate comparable alternatives	1	2%	
	Resources must be safe to use	1	2%	
	Midwives – shortage of support and understanding	1	2%	
	Outside pressures – May inhibit use	2	4%	

Topic of enquiry	Themes generated	Number of times referenced	%	Total number of statements categorised
	Relaxation techniques– benefit dependent on technique used	1	2%	
	Midwives – often feel guilty	2	4%	
	Need to generate viral content	1	2%	
	Midwives – May need additional support	1	2%	
	Relaxation techniques – May convey the wrong message	1	2%	
	Resources - could/should explain theory behind relaxation	1	2%	
	Midwives – Can apply their own knowledge	1	2%	
	Resources - Need to simple and comprehensive	1	2%	
	Relaxation - Limited evidence base	1	2%	
	Relaxation techniques – Requires a range of options	4	7%	
	Techniques - Consider mindfulness	1	2%	
**Topic of enquiry**	**Themes generated**	**Number of times referenced**	**%**	**Total number of statements categorised**
The inclusion of mindfulness tutorials and multimedia resources	Mindfulness - A helpful inclusion	26	44%	59
	Mindfulness - An unhelpful inclusion	2	3%	
	Resource – Need a variety of options available	3	5%	
	Mindfulness – Neutral opinion	2	3%	
	Mindfulness – Degree of evidence	2	3%	
	Mindfulness – Meaning unclear	3	5%	
	Midwives - should know this technique already	1	2%	
	Midwives – Face stigma	1	2%	
	Midwives - May not want face to face support	1	2%	
	Resources - need to be simple and safe to use	1	2%	
	Mindfulness - Midwives may be sceptical	2	3%	
	Mindfulness – Conflicted opinion	2	3%	
	Midwives – other pressures may inhibit use	1	2%	
	Midwives – Do not always recognise own distress	2	3%	
	Relaxation - synonymous with mindfulness	1	2%	
	Effectiveness - dependent on the degree of distress	1	2%	
	Mindfulness - A supportive professional friend would be better	1	2%	
	Resources - May send unwanted messages	1	2%	
	Resources – Must offer a variety of options	3	5%	
	Midwives – Must be risk assessed	1	2%	
	Midwives – Must be encouraged to seek professional help	1	2%	
	Resources – Must be accessible	1	2%	
**Topic of enquiry**	**Themes generated**	**Number of times referenced**	**%**	**Total number of statements categorised**
The inclusion of Cognitive behavioural Therapy (CBT) tutorials and multimedia resources	CBT tutorials - A helpful inclusion	18	29%	62
	CBT tutorials – An unhelpful inclusion	3	5%	
	Resources - Need a variety of options to suit all	2	3%	
	Intervention - Users may need additional support	9	15%	
	CBT tutorials - reduced evidence base	1	2%	
	CBT tutorials – Unclear meaning	2	3%	
	CBT tutorials - Needs to be easy and safe to use	1	2%	
	Resources - too many interventions may weaken the effect	2	3%	
	Midwives – May not access other CBT support	2	3%	
	Midwives – Need safety to disclose	2	3%	
	Midwives – face stigma	2	3%	
	Midwives – other pressures may inhibit use	1	2%	
	Midwives – May not be convinced of positive effect	1	2%	
	Effectiveness – Dependent on evidence and context	2	3%	
	Midwives – may need a targeted intervention	1	2%	
	Resources - Need to offer as many options as possible	1	2%	

	Midwives – May be impractical	1	2%	
	CBT tutorials - need to be professional and simple to use	1	2%	
	Resources - Consider Dialectical Behavioural Therapy (DBT)	2	3%	
	(CBT) tutorials - Question evidence base	1	2%	
	Midwives – Need risk assessment	1	2%	
	Midwives – Need encouragement to seek help	1	2%	
	Therapies - Evidence base instils confidence	2	3%	
	CBT – Unfamiliar with the therapy	1	2%	
	EMDR – Works well	2	3%	
**Topic of enquiry**	**Themes generated**	**Number of times referenced**	**%**	**Total number of statements categorised**
The inclusion of information designed to inform midwives where they can access alternative help and support	Signposted to help and support – A helpful inclusion	18	44%	41
	Help and support – Need a variety of options available	7	17%	
	Help and support – Must be evidence based	2	5%	
	Help and support – Face to face support preferable	3	7%	
	Alternative help and support – Unclear meaning	1	2%	
	Help and support - few resources actually available	1	2%	
	Therapies – Must be real and local	1	2%	
	Therapies – Too many = Confusion	1	2%	
	Midwives – Impaired functioning when distressed	2	5%	
	Therapies - EFT (Emotional Freedom Technique) can be useful	2	5%	
	Therapies – Suggest peer group debriefing	2	5%	
	Therapies – Suggest links to local occupational Health resources	1	2%	
**Topic of enquiry**	**Themes generated**	**Number of times referenced**	**%**	**Total number of statements categorised**
The inclusion of information designed to inform midwives as to where they can access legal help and advice	Legal help and advice - A helpful inclusion	24	60%	40
	Legal help and advice - An unhelpful inclusion	4	10%	
	Legal help and advice – conflicted opinion	3	8%	
	Legal help and advice - Unnecessary	4	10%	
	Legal help and advice – Question evidence base for this	1	3%	
	Legal help and advice - Few resources available	1	3%	
	Legal help and advice - Not a priority	1	3%	
	Legal Help and advice – Varies globally	1	3%	
	Legal help and advice - Consider providing personal legal advice	1	3%	
**Topic of enquiry**	**Themes generated**	**Number of times referenced**	**%**	**Total number of statements categorised**
Giving platform users the ability to share extended personal experiences for other platform users to read	extended personal experiences = A helpful inclusion	52	60%	87
	extended personal experiences – An unhelpful inclusion	5	6%	
	extended personal experiences - Must be optional	1	1%	
	extended personal experiences – conflicted opinion	6	7%	
	extended personal experiences – Requires moderation	7	8%	
	extended personal experiences = Must protect confidentiality	7	8%	
	extended personal experiences – Could be misused	2	2%	
	extended personal experiences - ethically problematic	1	1%	
	Midwives – if conducted within professional codes	1	1%	
	extended personal experiences - Requires anonymity	1	1%	
	extended personal experiences = Must protect anonymity	3	3%	
	extended personal experiences = Must remain professional	1	1%	

Topic of enquiry	Themes generated	Number of times referenced	%	Total number of statements categorised
The inclusion of a web based peer to peer discussion chat room	Peer to peer discussion - A helpful inclusion	31	40%	78
	Peer to peer discussion - An unhelpful inclusion	9	12%	
	Peer to peer discussion - Needs moderation	9	12%	
	Peer to peer discussion - Could risk confidentiality/anonymity	3	4%	
	Peer to peer discussion - Risk of unethical use	6	8%	
	Peer to peer chatroom - May not be used	2	3%	
	Peer to peer chatroom - Requires high volume site traffic	1	1%	
	Peer to peer chatroom - May require trained supporters	1	1%	
	Effectiveness – Depends upon help seeking behaviour	1	1%	
	Peer to peer chatroom - Requires rules and standards	1	1%	
	Professional – Legal/Regulatory obligations	1	1%	
	Midwives - May need local chat rooms	1	1%	
	Peer to peer discussion - Should be an optional choice	1	1%	
	Peer to peer discussion - Requires moderation	6	8%	
	Peer to peer discussion - Risk of misuse	1	1%	
	Peer to peer discussion - May risk anonymity/confidentiality	2	3%	
	Peer to peer discussion - Requires guidance	1	1%	
	Peer to peer discussion - May be local variations	1	1%	
**Topic of enquiry**	**Themes generated**	**Number of times referenced**	**%**	**Total number of statements categorised**
Giving platform users the ability to communicate any work or home based subjects of distress	Discussions re: work or home based subjects of distress – A helpful inclusion	13	26%	50
	Discussions re: work or home based subjects of distress - intertwined	13	26%	
	Discussions – unhelpful inclusion	10	20%	
	Discussions - Should be kept separate	4	8%	
	Discussions - uncontrollable	1	2%	
	Discussions – May risk anonymity/confidentiality	1	2%	
	Discussions – Require moderation	3	6%	
	Discussions – Require support	1	2%	
	Priority – Depends upon the context	3	6%	
	Discussions re: work or home based subjects of distress – chaotic	1	2%	
**Topic of enquiry**	**Themes generated**	**Number of times referenced**	**%**	**Total number of statements categorised**
An interface which does not resemble NHS, employer or other generic healthcare platforms	Resemblance – Should be authority neutral	28	56%	50
	Resemblance – Should be authority based	3	6%	
	Resemblance – Not important	3	6%	
	Resemblance - Variants on a global scale	1	2%	
	This would not matter if the intervention was clearly independent.	1	2%	
	Priority – user friendliness	5	10%	
	Question – relevance unclear	2	4%	
	Question – Cannot answer	1	2%	
	Midwives – Fearful of detection	2	4%	
	Intervention – Needs support of authorities	1	2%	
	Prioritise – visually safe space	1	2%	
	Intervention –confidentiality and anonymity important	1	2%	
	Intervention - Consider analysing feedback	1	2%	
**Topic of enquiry**	**Themes generated**	**Number of**	**%**	**Total**

		times referenced		number of statements categorised
A simple, anonymised email login procedure which allows for continued contact and reminders which may prompt further platform usage	Anonymised email login procedure - A helpful inclusion	19	36%	53
	Anonymised email login procedure - An unhelpful inclusion	13	25%	
	Anonymised email login procedure – must be optional	1	2%	
	Priorities – A user-friendly intervention	9	17%	
	Prompting – A helpful inclusion	4	8%	
	Anonymised email login procedure – Unsure of alternatives	1	2%	
	Midwives – May require alternative support	1	2%	
	Prompting – An unhelpful inclusion	4	8%	
	Confidentiality must be upheld	1	2%	

**Topic of enquiry**	**Themes generated**	**Number of times referenced**	**%**	**Total number of statements categorised**
An automated moderating system where ‘key words’ would automatically initiate a moderated response	‘key words’ initiating a moderated response – A helpful inclusion	15	22%	67
	‘key words’ initiating a moderated response – an unhelpful inclusion	13	19%	
	‘key words’ initiating a moderated response – Moderation is required	6	9%	
	‘key words’ initiating a moderated response – conflicted	5	7%	
	‘key words’ initiating a moderated response –must be supportive in nature	1	1%	
	‘key words’ initiating a moderated response – May not be adequate	8	12%	
	‘key words’ initiating a moderated response – Confusing	1	1%	
	Midwives – Need to be risk assessed	2	3%	
	Question – Need to know more	11	16%	
	Midwives – Must be protected from suicide	3	4%	
	‘key words’ initiating a moderated response – Must be sophisticated	1	1%	
	‘key words’ initiating a moderated response – Moderation = high maintenance	1	1%	

**Topic of enquiry**	**Themes generated**	**Number of times referenced**	**%**	**Total number of statements categorised**
Mobile device compatibility for platform users	Mobile device compatibility – High priority	35	73%	48
	Mobile device compatibility - Unhelpful	1	2%	
	Mobile device compatibility – Must work	6	13%	
	mobile device compatibility – Neutrality important	1	2%	
	mobile device compatibility – Must be secure	1	2%	
	Midwives – Require support	2	4%	
	Midwives – may not seek alternative support	1	2%	
	Intervention - Risky	1	2%	

Ethical Inclusions

Participants reached consensus and expressed a strong need to permit confidentiality within an online intervention. This was largely due to the opinion that midwives would not feel able to engage with or speak openly within an online intervention designed to support them without confidentiality. There was also a concern that midwives would not feel able to speak openly for fear of retribution or 'recriminations', either by their employer, regulator or by the public should the provision of confidentiality not be in place. Some users felt that the level of individual confidentiality should be left to the user to decide

There was concern about how anonymity may be achieved within an online intervention designed to support midwives. This was partnered with concerns around the ethical and legal responsibilities of both platform users and developers in providing reactive interventions to 'at risk' midwives. Additionally, concerns were raised about the obligations a user or developer may have in relation to the disclosure of dangerous practice, safeguarding issues, or discrimination which may put the reputation of the midwifery profession at risk.

Many respondents stated that people generally may feel more able to reveal personal information if they cannot be recognised. This provision of anonymity was therefore considered to have a therapeutic advantage. However concerns arose for some about the need to act upon potential disclosures of dangerous practice, which the provision of anonymity would prevent.

Some suggestions were made as to how anonymity could be achieved. For example, through the use of pseudonyms. Others suggested that users should be able to be 'traced' in exceptional circumstances for the protection of the service user and the public. Overall, the

consensus to this enquiry was that anonymity and confidentiality would be a high priority in the development of an online intervention designed to support midwives.

Participants were conflicted over whether or not amnesty should be permitted within an online intervention designed to support midwives. For some, it was clear that an amnesty would be therapeutic and provide midwives with an opportunity to speak openly, others had concerns regarding legal and ethical requirements to refer midwives who may be in breach of their professional responsibilities.

41% of 81 statements provided in relation to reminding users of their professional responsibilities in relation to codes of conduct, referred to the need the importance of adhering to the code and reporting breaches of codes of conduct. The importance of the midwife adhering to their professional responsibilities and codes of conduct remained a majority theme throughout.

Inclusions of Therapeutic Support

It was agreed that an online intervention designed to support midwives in work-related psychological distress should prioritise the inclusion of informative multimedia to help midwives recognise their own signs and symptoms of psychological distress, as 50% of statements in this category agreed that these would be a useful inclusion. 49% of subject specific statements agreed that multimedia designed to explore topics around psychological distress would also be a helpful inclusion. 3.32% of all generated statements referred to a need to offer a variety of options to accommodate all user types.

1% of statements in relation to self-care techniques and 3% of statements in relation to cognitive behavioural therapy detailed how those participants were unfamiliar with these particular therapies. On the topic of mindfulness, 5% of statements submitted that those

participants were not familiar with mindfulness as an approach. 44% of other statements given in regards to the provision of mindfulness, stated that it would be a high priority for inclusion. In regards to other types of therapeutic inclusions, 45% of subject specific statements agreed that the dissemination of self-care techniques would be a helpful inclusion, 33% of subject specific statements agreed that relaxation techniques would be a helpful inclusion, and 29% of subject specific statements agreed that Cognitive Behavioural Therapy would be a helpful inclusion. A few participants suggested other therapeutic approaches that could be considered, including Eye Movement Desensitization and Reprocessing (EMDR), Emotional Freedom Technique (EFT) and Dialectical Behaviour Therapy (DBT)

In relation to providing legal advice and information, there was concern for some that “the mere suggestion that they might want to access legal support may add further stress to midwives who hadn’t previously considered that option”. There were also some references to the fact that this information may already be freely available through unions or governing bodies.

Many statements categorised referred to the concept of fear in relation to speaking openly, retribution, bullying, complaints, litigation and investigations with one comment suggesting that “complaints, investigations and litigation are some of midwives biggest fears”.

60% of subject specific responses in relation to the sharing of extended personal experiences agreed that this provision would be a helpful inclusion. One comment reported that “Writing and sharing is a very powerful and helpful tool in treating psychological distress”. Another stipulated how experience "lived" has so much to give to the "reader". One other expert noted how “It is important to be able to share stories to help

with others supporting and to help the person involved feel heard and not alone and also that often just verbalising issues albeit online is a huge step to help process it and prevent people internalising it and making it worse”. However, the majority of alternate statements referred to concerns in relation to potential breaches of ethics, professional codes and confidentiality.

Many participants felt that discussions around work-based stresses and personal stresses were inextricably linked, as 26% of subject specific responses referred to this standpoint. 26% of subject specific statements agreed that the ability to speak about both sources of distress at both work and home would be a helpful inclusion. Yet many subject specific responses (20%) believed that this would be an unhelpful inclusion. Others referred to a need to keep these discussions either focussed upon work-related stress or separate.

In relation to the provision of a web based peer to peer discussion chat room, a consensus of opinion was not reached. 40% of subject specific statements agreed that a web based peer to peer discussion chat room would be a helpful inclusion, yet the majority of related statements cited concerns in relation to the risks of breaching confidentiality and the risk of unprofessional behaviour. One statement explained how the participant had been “empowered by the exchanges of knowledge and experience” within alternative online support groups, however, the need for online moderation remained a highlighted theme.

Intervention Design and Practical Inclusions

Participants were asked whether the online intervention should prioritise an interface which does not resemble NHS, employer or other generic healthcare platform. Some participants were unsure as to why this may be important, as 10% of subject specific statements reflected. Others stated that midwives may feel safer in speaking out if they felt that the

intervention was somehow separated from authority, with 56% of subject specific responses agreeing that having a platform which remained unrelated to authority would be helpful. One participant noted however that the intervention “Needs to be supported by the NHS and look professional”.

A large number of free text responses noted the importance of making sure that the intervention was “easy to use and visually appealing”. Some also noted the need for the platform to be user friendly and easy to access.

In relation to the provision of an anonymised email login procedure, 36% of subject specific statements agreed that this would be a helpful inclusion. However, the panel became divided in opinion on whether the platform should prompt further usage via email, with 8% of statements referring to this as a helpful inclusion and 8% of statements regarding this as being an unhelpful inclusion.

In relation to the prioritisation of an automated moderating system where ‘key words’ would automatically initiate a moderated response, 16% of subject specific statements highlighted that panel members were unclear as to what this may mean. The issue we are referring to here is a safety measure which may allow the online platform to provide a message about National Suicide Prevention services if keyword searches suggest suicidal feelings. This is a concept described by Luxton and colleagues (Luxton, June, & Fairall, 2012).

Example Comment: I am having **suicidal thoughts** today.

Example automated response: As you have been discussing **Suicidal thoughts,** we would like to let you know that help is available at Lifeline on 131 114 or online. Alternatively you can call the Suicide Call Back Service on 1300 659 467.

Panel members remained divided on this issue, as 22% of subject specific statements indicated that an automated moderating system would be a helpful inclusion, and 19% of statements indicated that this would be an unhelpful inclusion.

Lastly, 73% of subject specific statements agreed that an online intervention designed to support midwives in work-related psychological distress should prioritise mobile device compatibility for platform users. Other statements in relation to this emphasised a general need to promote security and usability. One comment noted that some midwives may be averse to mobile platforms, overall, the panel expressed a consensus of opinion that mobile device compatibility should be a priority in the development of an online intervention designed to support midwives in work-related psychological distress.

Next Steps

We would like to thank all panel members for their responses, ideas and opinions. We hope that all 66 panel members will now move on to complete the second round of this Delphi study, so that further consensus may be reached.

We invite you to participate in round 2, as you consider the responses from your fellow panel members to round 1.

The questions which did not reach a consensus of opinion in round 1, along with some new questions based on your comments in round 1, will be presented to all panellists in round 2. Once again, there are no right or wrong answers. It is your opinion we are interested in. We look forward to receiving your responses, and thank you once again for all the time and trouble you have taken with this project.

References

Luxton, D. D., June, J. D., & Fairall, J. M. (2012). Social media and suicide: A public health perspective. *American Journal of Public Health*, *102*(S2), S195-S200.
