# Supplementary material for: Achieving Consensus for the Design and Delivery of an Online Intervention to Support Midwives in Work-Related Psychological Distress: Results From a Delphi Study
Source: JMIR Ment Health. 2016 Jul 12;3(3):e32. doi: 10.2196/mental.5617 (PMC4961877; doi:10.2196/mental.5617)
Supplement: Multimedia Appendix 3 [file mental_v3i3e32_app3.pdf]

Delphi Study second round Participant Report: Delphi Study to achieve Consensus in the Development of an Online Intervention Designed to Effectively Support Midwives in Work related Psychological Distress.

Round Two

Thankyou from the research team

44 of you completed this second round of questioning designed to achieve consensus in the development of an online intervention designed to support midwives in work-related psychological distress. Thankyou! Your responses were informative, useful and very valuable to this project.

Overview of Round Two

A total of 185 people were invited to participate in this Delphi Study. 66 participants completed the first round of questioning. These 66 participants were then asked to complete a second round of questioning. 44 (66.6%) of those invited to participate completed this second round of questioning. Panellists were asked to assign a priority rating to 20 statements during the first round of questioning and 19 statements during the second round of questioning using a 7 point likert type scale. 11 items achieved a consensus of opinion during round one, 9 did not. These 9 items were returned to the panel during this second round of questioning. 10 new questions were also brought before the panel during this second round of questioning. Prior to receiving responses, we set some criteria for consensus. As such, consensus of opinion was considered to be reached when 60% or more of the responses fell within 2 adjacent response points on the 7-point scale.

example	Not a priority	Low priority	Somewhat a priority	Neutral	Moderate priority	High priority	Essential priority
An interface which does not resemble NHS, employer or other generic healthcare platforms	☐	☐	☐	☐	☐	☐	☐

Summary of Round Two

The research team assessed whether each item had achieved a consensus of opinion or not. The team then went on to allocate each free text response to one or more themes. A total of 19 statements were put forward to the panel. Of the 19 questions posed within this second round, 7 statements achieved consensus and 12 did not. A total of 704 free text responses were provided by the panel. These were categorised into 1059 separate statements and allocated to a theme by two members of the research team. Table one summarises the results from round two of this Delphi study.

Table 1: Results Summary for Round Two Questionnaire

	**Question:** **An online intervention designed to support midwives in work-related psychological distress should prioritise:**	**Consensus achieved**	**% of Consensus**	**Mean Score**	**Comments submitted in free text**	**Statements Categorised**	**Themes Generated**
1.	Amnesty for all platform users in that they will not be referred to any law enforcement agencies, their employer or regulatory body for either disciplinary or investigative proceedings in any case.	No	N/A	4.61	58	103	14
2.	Prompting platform users automatically to remind them of their responsibilities to their professional codes of conduct.	No	N/A	4.18	49	72	12
3.	The inclusion of information designed to inform midwives as to where they can access legal help and advice.	Yes (High/Essential Priority)	65.9%	5.57	37	53	7
4.	Giving platform users the ability to share extended personal experiences for other platform users to read	No	N/A	4.75	47	71	7
5.	The inclusion of a web based peer to peer discussion chat room	Yes (Moderate/High priority)	63.6%	4.86	43	73	7
6.	Giving platform users the ability to communicate any work or home based subjects of distress	No	N/A	5.18	39	54	10
7.	An interface which does not resemble NHS, employer or other generic healthcare platforms	No	N/A	5.23	35	53	10

8.	A simple, anonymised email login procedure which allows for continued contact and reminders which may prompt further platform usage	No	N/A	5.34	32	47	10
9.	An automated moderating system where 'key words' would automatically initiate a moderated response	No	N/A	4.48	34	51	9
10	An interface which resembles and works in a similar way to current popular and fast pace social media channels: e.g. Facebook	No	N/A	4.82	38	54	9
11	The inclusion of midwives from around the world	No	N/A	4.48	41	57	5
12	Proactive moderation (i.e, users are able to block unwanted content and online postings are 'pre-approved')	Yes (High/Essential priority)	61.4%	5.5	36	58	6
13	Reactive moderation (i.e., users are able to report inappropriate content to a system moderator for removal)	Yes (High/Essential priority)	70.5%	5.68	25	36	3
14	24/7 availability of the platform	Yes (High/Essential priority)	84.1%	6.3	33	41	4
15	The implementation of an initial simple user assessment using a psychological distress scale to prompt the user to access the most suitable support available	Yes (Moderate /High priority)	70.4%	5.39	31	45	6
16	The gathering of anonymised data and concerns from users, only with explicit permission, so that trends and concerns	No	N/A	5.39	33	50	6

	may be highlighted at a national level.						
17	Access for a midwife's friends and family members	No	N/A	2.68	33	47	6
18	The following up and identification of those at risk	Yes (High/Essential priority)	63.7%	5.59	31	55	5
19	The provision of a general statement about professional codes of conduct and the need for users to keep in mind their responsibilities in relation to them	No	N/A	5.07	29	39	6
					**Total= 704**	**Total = 1059**	**Total =142**

Table two summarises the responses given during round two of this Delphi study. Please note that percentages have been rounded toward the nearest whole number.

Table 2: Summary of round Two responses

Topic of enquiry	Theme	Number of times categorised	%	Total number of statements categorised
Amnesty for all platform users in that they will not be referred to any law enforcement agencies, their employer or regulatory body for either disciplinary or investigative proceedings in any case.	Amnesty – Required for open and honest disclosure	27	26%	103
	Midwives - Fear retribution	11	11%	
	Amnesty – Cannot be given in any circumstances	1	1%	
	Amnesty - Cannot be given in all cases	25	24%	
	Amnesty - Required for change/Help seeking	9	9%	
	Amnesty - Conflicted in opinion	15	15%	
	Amnesty - required for privacy	1	1%	
	Midwives - Have little support	2	2%	
	Amnesty - A helpful inclusion	3	3%	
	Intervention - Requires disclaimer policies	2	2%	
	Amnesty - Difficult to moderate	1	1%	
	Amnesty - An unhelpful inclusion	1	1%	
	Amnesty can enable resolution of situations	4	4%	
	Question - Meaning unclear	1	1%	

**Topic of Enquiry**	**Theme**	**Number of times categorised**	**%**	**Total number of statements categorised**
Prompting platform users automatically to remind them of their responsibilities to their professional codes of conduct.	Prompting professional codes - A helpful inclusion	21	29%	72
	Prompting professional codes - An unhelpful inclusion	17	24%	
	Prompting professional codes - Ethically essential	9	13%	
	Midwives - Already aware of codes - Not required	6	8%	
	Prompting professional codes - Not the purpose of the intervention	3	4%	
	Midwives - Duty of care should be priority	2	3%	
	Codes - Inadequate	1	1%	
	Prompting professional codes - Alternative approach required	6	8%	
	Midwives - Need support	1	1%	
	Midwives - Should remain professional even in distress	3	4%	
	Midwives - If needing reminders, should not be working	2	3%	
	Prompting professional codes - Requires sensitivity	1	1%	
**Topic of enquiry**	**Theme**	**Number of times categorised**	**%**	**Total number of statements categorised**
Information designed to inform midwives as to where they can access legal help and advice.	Information, legal help and advice - A helpful inclusion	32	60%	53
	Information, legal help and advice - An unhelpful inclusion	4	8%	
	Intervention - A range of options should be made available	5	9%	
	Question - Meaning unclear	2	4%	
	Midwives - Fear retribution	1	2%	
	Midwives - Need support	6	11%	
	Information, legal help and advice - Can be found elsewhere	2	4%	
	Midwives - Blame themselves	1	2%	
**Topic of enquiry**	**Theme**	**Number of times categorised**	**%**	**Total number of statements categorised**
Giving platform users the ability to share extended personal experiences for other platform users to read	Sharing extended personal experiences - A helpful inclusion	41	58%	71
	Sharing extended personal experiences- An unhelpful inclusion	6	8%	
	Sharing extended personal experiences - Moderation required	11	15%	
	Sharing extended personal experiences - Should be optional	6	8%	
	Sharing extended personal experiences - Undecided	2	3%	
	Sharing extended personal experiences - Risky	3	4%	
	Sharing extended personal experiences - effect may be context dependant	2	3%	
**Topic of enquiry**	**Theme**	**Number of times categorised**	**%**	**Total number of statements categorised**
The inclusion of a web based peer to peer discussion chat room	Discussion chat room - A helpful inclusion	37	51%	73
	Discussion chat room- An unhelpful inclusion	11	15%	
	Discussion chat room - Moderation required	10	14%	
	Discussion chat room - More information required	6	8%	
	Discussion chat room - Risky	7	10%	
	Discussion chat room- Challenging to facilitate	1	1%	
	Discussion chat room - Consider additional features	1	1%	

Topic of enquiry	Theme	Number of times categorised	%	Total number of statements categorised
Giving platform users the ability to communicate any work or home based subjects of distress	Communicating any work or home based subjects of distress - A helpful inclusion	15	28%	54
	Communicating any work or home based subjects of distress - An unhelpful inclusion	9	17%	
	Communicating any work or home based subjects of distress -Inevitable	7	13%	
	Communicating any work or home based subjects of distress - Both subjects intertwined	16	30%	
	Communicating any work or home based subjects of distress - Risk of breaching confidentiality	1	2%	
	Communicating any work or home based subjects of distress - Requires moderation	2	4%	
	Communicating any work or home based subjects of distress - Undecided	1	2%	
	Communicating any work or home based subjects of distress - Difficult to engage whilst at work	1	2%	
	Communicating any work or home based subjects of distress - discussions need to remain workplace based	1	2%	
	Communicating any work or home based subjects of distress - Consider links to outside agencies	1	2%	
**Topic of enquiry**	**Theme**	**Number of times categorised**	**%**	**Total number of statements categorised**
An interface which does not resemble NHS, employer or other generic healthcare platforms	Online intervention interface - Should not resemble NHS, employer or other generic healthcare platforms	21	40%	53
	Midwives - Fear bringing the profession into disrepute	1	2%	
	Online intervention interface - Should prioritise usability	6	11%	
	Online intervention interface - Should look professional	8	15%	
	Conflicted opinion	3	6%	
	Midwives - May not engage if they fear organisational involvement	11	21%	
	Intervention - Should be a safe haven	1	2%	
	Intervention - Must appear to be for midwives only	1	2%	
	Online intervention interface - Options should be researched	1	2%	
**Topic of enquiry**	**Theme**	**Number of times categorised**	**%**	**Total number of statements categorised**
A simple, anonymised email login procedure which allows for continued contact and reminders which may prompt further platform usage	Anonymised email login procedure - A helpful inclusion	19	40%	47
	Anonymised email login procedure - An unhelpful inclusion	4	9%	
	Anonymised email login procedure - Anonymity may not be possible	2	4%	
	Undecided	2	4%	
	Anonymised email login procedure - Can be used to intervene	3	6%	
	Anonymity - Essential	2	4%	
	Anonymised email login procedure - Ease of use a priority	8	17%	
	Anonymised email login procedure - Security a priority	2	4%	
	Anonymised email login procedure - Should be optional	2	4%	
	Anonymised email login procedure - Risky	3	6%	
**Topic of enquiry**	**Theme**	**Number of times**	**%**	**Total number of**

		categorised		statements categorised
An automated moderating system where 'key words' would automatically initiate a moderated response	An automated moderating system - A helpful inclusion	16	31%	51
	An automated moderating system - An unhelpful inclusion	9	18%	
	An automated moderating system - Meaning unclear	8	16%	
	Undecided	5	10%	
	Moderation - Should be a priority	3	6%	
	Intervention - Must be a safe space	1	2%	
	An automated moderating system - Must be appropriate	6	12%	
	Moderation - Should be a human response	2	4%	
	An automated moderating system - Should allow users to flag concerns	1	2%	
**Topic of enquiry**	**Theme**	**Number of times categorised**	**%**	**Total number of statements categorised**
An interface which resembles and works in a similar way to current popular and fast pace social media channels: e.g. Facebook	An interface which resembles and works in a similar way to current popular and fast pace social media channels - Helpful	20	37%	54
	An interface which resembles and works in a similar way to current popular and fast pace social media channels - unhelpful	15	28%	
	Usability should be the priority	12	22%	
	An interface which resembles and works in a similar way to current popular and fast pace social media channels - Undecided	6	11%	
	Question - Misunderstood	1	2%	
**Topic of enquiry**	**Theme**	**Number of times categorised**	**%**	**Total number of statements categorised**
The inclusion of midwives from around the world	The inclusion of midwives from around the world - Helpful	24	42%	57
	The inclusion of midwives from around the world - unhelpful	14	25%	
	The inclusion of midwives from around the world - Undecided	6	11%	
	The inclusion of midwives from around the world - Challenging to facilitate	11	19%	
	The inclusion of midwives from around the world - Could be made fit for purpose	2	4%	
**Topic of enquiry**	**Theme**	**Number of times categorised**	**%**	**Total number of statements categorised**
Proactive moderation (i.e., users are able to block unwanted content and online postings are 'pre-approved')	Proactive moderation - Helpful	24	41%	58
	Proactive moderation - Unhelpful	11	19%	
	Proactive moderation - Must be tailored to suit context	11	19%	
	Proactive moderation - Meaning unclear	2	3%	
	Moderation - essential	9	16%	
	Midwives - Able to self-moderate	1	2%	
**Topic of enquiry**	**Theme**	**Number of times categorised**	**%**	**Total number of statements categorised**
Reactive moderation (i.e., users are able to report	Reactive moderation - Helpful	24	67%	36
	Reactive moderation - Unhelpful	5	14%	
	Reactive moderation - Design challenges	7	19%	

inappropriate content to a system moderator for removal)				
**Topic of enquiry**	**Theme**	**Number of times categorised**	**%**	**Total number of statements categorised**
24/7 availability of the platform	24/7 availability - Helpful	37	90%	41
	24/7 availability - Undecided	1	2%	
	Midwives - Need confidentiality	1	2%	
	Midwives - Have no time	2	5%	
**Topic of enquiry**	**Theme**	**Number of times categorised**	**%**	**Total number of statements categorised**
The implementation of an initial simple user assessment using a psychological distress scale to prompt the user to access the most suitable support available	Simple user assessment - Helpful	25	56%	45
	Simple user assessment - unhelpful	2	4%	
	Simple user assessment - Context for use required	11	24%	
	Simple user assessment - Undecided	3	7%	
	Simple user assessment - Should be optional	1	2%	
	Simple user assessment - Context for use required	3	7%	
**Topic of enquiry**	**Theme**	**Number of times categorised**	**%**	**Total number of statements categorised**
The gathering of anonymised data and concerns from users, only with explicit permission, so that trends and concerns may be highlighted at a national level.	The gathering of anonymised data and concerns - Helpful	32	64%	50
	The gathering of anonymised data and concerns - Unhelpful	11	22%	
	Midwives - Require anonymity	2	4%	
	The gathering of anonymised data and concerns - Undecided	1	2%	
	The gathering of anonymised data and concerns - Requires ethical consideration	3	6%	
	Midwives - Require confidentiality	1	2%	
**Topic of enquiry**	**Theme**	**Number of times categorised**	**%**	**Total number of statements categorised**
Access for a midwife's friends and family members	Access for a midwife's friends and family - Helpful	4	9%	47
	Access for a midwife's friends and family - Unhelpful	17	36%	
	Access for a midwife's friends and family - Undecided	7	15%	
	Access for a midwife's friends and family - Ethical considerations must be recognised	10	21%	
	Access for a midwife's friends and family - Could require a separate, designated area	6	13%	
	Access for a midwife's friends and family - Need more information	3	6%	
**Topic of enquiry**	**Theme**	**Number of times categorised**	**%**	**Total number of statements categorised**

The following up and identification of those at risk	The following up and identification of those at risk - Helpful	23	42%	55
	The following up and identification of those at risk - Unhelpful	9	16%	
	The following up and identification of those at risk - Ethical considerations must be recognised	14	25%	
	The following up and identification of those at risk - Undecided	6	11%	
	The following up and identification of those at risk - Beyond the scope of this project	3	5%	
**Topic of enquiry**	**Theme**	**Number of times categorised**	**%**	**Total number of statements categorised**
The provision of a general statement about professional codes of conduct and the need for users to keep in mind their	A general statement about professional codes of conduct - Helpful	15	38%	39
	A general statement about professional codes of conduct - Unhelpful	8	21%	
	A general statement about professional codes of conduct - Must be applicable	2	5%	
	Midwives - Already aware of codes	4	10%	
	Online community - Should develop its own codes of conduct	2	5%	
	Achieving consensus - Frustrated by survey questions	5	13%	
	A general statement about professional codes of conduct - Requires sensitivity	3	8%	

Items which did not achieve a consensus of opinion within the first round of questioning

The first 9 statements put forward within this second round of questioning were statements returned to the panel which failed to reach a consensus in Round One. The purpose of this was to allow participants to consider these questions once more, in light of the group responses put forward following the first round of questioning. Participants were encouraged to deliberate upon the group response prior to approaching the second round of questioning. 2 of these 9 statements achieved consensus.

Participants agreed that the inclusion of information designed to inform midwives as to where they can access legal help and advice should be a high/essential priority. Consensus was 65.9%. 60% of free text responses alluded to this as a helpful inclusion for some, however, others suggested that this may be unhelpful, and suggested that midwives could access this information elsewhere.

Participants also agreed that the inclusion of a web based peer to peer discussion chat room should be a moderate to high priority. Consensus was 63.6%. The majority of participants suggested that this would be a helpful inclusion, however, many expressed concerns over the risks of speaking openly within an online forum, and expressed the need for appropriate moderation.

Seven lines of enquiry re-presented to the panel within this second round of questioning failed to achieve a consensus of opinion. Free text responses given within these items offered the research team key insights and ideas to explore. Some participants remained reluctant to the idea of providing amnesty within an online platform designed to assist midwives in distress. Here, although 26% of participant statements acknowledged that midwives may require an amnesty in order to speak openly and seek help, others expressed a moral discomfort in permitting this.

The panel also remained conflicted upon the subject of prompting platform users automatically to remind them of their responsibilities to their professional codes of conduct, as many felt that this would be a helpful inclusion, others remained cautious about escalating fear and distress for midwives seeking help. 24% of panellist comments indicated that this would be an unhelpful inclusion, whilst alternatively, 29% of comments indicated that this inclusion would be a helpful inclusion. Upon the subject of giving platform users the ability to share extended personal experiences for other platform users to read, 58% of panellist statements agreed that this would be a useful inclusion for some. However, other panel members remained concerned that this may give rise to breaches in confidentiality, and advocated a need for strict moderation. Similar concerns and comments also arose as

the panel were asked about giving platform users the ability to communicate any work or home based subjects of distress.

Regarding the visual branding of the platform and any association with the NHS, employers or other generic healthcare platforms, some panellists reported that the platform “needs to feel legitimate” in order to enable midwives to feel confident in speaking openly. 40% of panellist statements felt that an online intervention designed to support midwives in work-related psychological distress should not resemble NHS, employer or other generic healthcare platforms, and many feared that midwives would be reluctant to speak out if they felt that they were being observed by an employer. Similarly, panellists expressed a fear that midwives may fear being “Identified” through the use of a simple, anonymised email login procedure which allows for continued contact and reminders. However, 40% of participant statements indicated that an anonymised email login procedure would be a helpful inclusion. Lastly, panellists were asked for their opinions about an automated moderating system where ‘key words’ would automatically initiate a moderated response. 31% of participant statements agreed that this type of moderation would be helpful, though some remained confused as to how this intervention may work in practice.

New items for consideration

10 new questions were put forward before the panel. These questions were either generated by the data collected during round one, or requested by the expert panel. 5 out of these 10 new questions achieved a consensus of opinion.

Participants were asked to give their opinions about whether an interface which resembles and works in a similar way to current popular and fast pace social media channels: e.g. Facebook should be prioritised. Although participants generally recognised a need for

platform users to enjoy an ease of use, they expressed a reluctance to approve the use of Facebook within the midwifery profession. Overall, 28% of participant statements indicated that this interface would be unhelpful, and 37% of participant statements indicated that this interface would be helpful. Participants were then asked whether the platform should be open for midwives from around the world to use. Although many participants were open to this, others expressed a need to primarily interact with a smaller cohort of local midwives in order to be able to be able to identify with a comparable group of midwives. It was suggested that a broader user group could become a future goal for the platform to accomplish.

Throughout both rounds of this Delphi study, many expert panellists have expressed a need to prioritise appropriate moderation for this online intervention designed to support midwives in work-related psychological distress. Therefore, panellists were asked to offer their opinions in relation to both proactive moderation (i.e., users are able to block unwanted content and online postings are 'pre-approved') and reactive moderation (i.e., users are able to report inappropriate content to a system moderator for removal). Although neither of these options achieved a consensus in opinion, the research team were able to explore key themes and ideas in relation to potential moderation techniques. Overwhelmingly, participants agreed that moderation should remain an essential priority. However, panellists were somewhat conflicted about whether this moderation should be proactive or reactive. Whilst both varieties of moderation were deemed applicable, 61.4% of participant statements indicated that proactive moderation would be helpful and 70.5% of participant statements indicated that reactive moderation would be helpful. It was acknowledged that users may not want to wait for content to be ‘pre-approved’, yet concerns remained over any potential exposure to harmful content. Alternatively, many

expressed that an ability to alert moderators to inappropriate content and an individual ability to block access to unwanted content may be sufficient in assuring appropriate moderation.

Overwhelmingly, 90% of participant comments agreed that the platform should be available to users 24/7. It was also agreed that the implementation of an initial simple user assessment using a psychological distress scale to prompt the user to access the most suitable support available should be prioritised, as 56% of participant comments suggested that this would be a helpful inclusion. However, panellists were unsure how this may be achieved or which user assessment tool may be appropriate. Additionally, some comments suggested that this component may be too intrusive for platform users. When asked about the gathering of anonymised data and concerns from users, with explicit permission, so that trends and concerns may be highlighted at a national level, 40% of panel member comments expressed that there would be value in highlighting trends and concerns. However, ethical considerations were raised and some were concerned that data collection may dissuade some platform users from candour. This concern was also cited as one reason that there should be no access for a midwife's friends and family members. However, no firm consensus could be reached upon this issue.

Panel members agreed that the following up and identification of those at risk was a high/essential priority (63.7%), though many identified difficulties in doing so. It was also suggested that this may be too intrusive for platform users wishing to remain anonymous. Lastly, panel members were asked whether the provision of a general statement about professional codes of conduct and the need for users to keep in mind their responsibilities in relation to them should be a priority. Although 38% agreed that this inclusion would be

helpful, 21% were concerned that this inclusion may be unhelpful and cause further distress. Some suggestions were made that the platform should evolve its own codes of conduct as a separate community of support.

Next Steps

We would like to thank all panel members for their responses, ideas and opinions kindly offered within this Delphi study. Your input to this study has been invaluable, and the research team now have a much deeper understanding of what should be prioritised in the development of an online intervention designed to support midwives in work-related psychological distress.

The research team now plans to publish the results of this study within a peer reviewed journal. Once this has been achieved, you will be sent a copy of any articles published. Should you wish to opt out of this and/or decline any future correspondence, please contact the research team accordingly.

The research team would like to thank you once again for all the time and trouble you have taken with this project.
