# Supplementary material for: Achieving Consensus for the Design and Delivery of an Online Intervention to Support Midwives in Work-Related Psychological Distress: Results From a Delphi Study
Source: JMIR Ment Health. 2016 Jul 12;3(3):e32. doi: 10.2196/mental.5617 (PMC4961877; doi:10.2196/mental.5617)
Supplement: Multimedia Appendix 4 [file mental_v3i3e32_app4.pdf]

| Item | Round | Theme | Count | % |
|---|---|---|---|---|
| An online intervention designed to support midwives in work-related psychological distress should prioritize confidentiality for all platform users and service users in all matters of discussion. | 1 - Ethical inclusions | Confidentiality – Required for open and honest disclosure | 23 | 27% |
| | | Confidentiality – Essential criterion for provision of support | 10 | 12% |
| | | Midwives – Need to feel safe | 10 | 12% |
| | | Confidentiality – for third parties | 9 | 11% |
| | | Midwives - Fear retribution | 6 | 7% |
| | | Confidentiality – Decided by user | 5 | 6% |
| | | Confidentiality – Context dependent | 4 | 5% |
| | | Confidentiality – Needed to avoid public identification | 3 | 4% |
| | | Confidentiality – High priority | 2 | 2% |
| | | Midwives - May need further support/ intervention | 2 | 2% |
| | | Midwives – Feel shame if not managing | 2 | 2% |
| | | Confidentiality - Needed to protect the reputation of the profession | 2 | 2% |
| | | Professional – Legal/Regulatory obligations | 1 | 1% |
| | | Midwives – Fear consequences | 1 | 1% |
| | | Confidentiality – Not possible online | 1 | 1% |
| | | Midwives – Need reassurance | 1 | 1% |
| | | Confidentiality - required to promote disclosure | 1 | 1% |
| | | Confidentiality – Not possible online | 1 | 1% |
| | | Midwives – Have little existing provision | 1 | 1% |
| | | **Total** | **85** | |
| **Item** | **Round** | **Theme** | **Count** | **%** |
| An online intervention designed to support midwives in work-related psychological distress should prioritise anonymity for all platform users and service users in all matters of discussion. | 1 - Ethical inclusions | Anonymity – Required for open and honest disclosure | 18 | 21% |
| | | Anonymity – Needed for support | 10 | 11% |
| | | Anonymity - Decided by user | 6 | 7% |
| | | Midwives – Fear retribution | 6 | 7% |
| | | Anonymity – for third parties | 5 | 6% |
| | | Anonymity - could be misused/cause distress | 4 | 5% |
| | | Feeling safe/safety - Required | 4 | 5% |
| | | Professional – Legal/Regulatory obligations | 4 | 5% |
| | | Anonymity – May prevent further intervention | 3 | 3% |
| | | Midwives – Support is highest priority | 3 | 3% |
| | | Anonymity – Not possible online | 3 | 3% |
| | | Anonymity – Use of pseudonyms | 2 | 2% |

| | | | | |
|---|---|---|---|---|
| | | Anonymity - Required for open disclosure | 2 | 2% |
| | | Practicalities – Additional support may be required | 2 | 2% |
| | | Practicalities – Legal obligations over raising concerns | 2 | 2% |
| | | Anonymity – Needed to feel safe | 2 | 2% |
| | | Anonymity is synonymous with confidentiality | 2 | 2% |
| | | Anonymity – Optionality required | 1 | 1% |
| | | Anonymity – Requires policy | 1 | 1% |
| | | Practicalities – User verification | 1 | 1% |
| | | Midwives – Fearful of disclosure | 1 | 1% |
| | | Anonymity – Needed to seek support | 1 | 1% |
| | | Midwives – Support is a high priority | 1 | 1% |
| | | Anonymity – Unsure of relevance | 1 | 1% |
| | | Midwives – Need assurances | 1 | 1% |
| | | Midwives – Feel shame if not managing | 1 | 1% |
| | | **Total** | **87** | |
| **Item** | **Round** | **Theme** | **Count** | **%** |
| An online intervention designed to support midwives in work-related psychological distress should prioritise amnesty for all platform users in that they will not be referred to any law enforcement agencies, their employer or regulatory body for either disciplinary or investigative proceedings in any case. | 1 - Ethical inclusions | Amnesty – Important/Helpful | 19 | 14% |
| | | Amnesty – Legal and ethical obligations – duty of care | 18 | 13% |
| | | Midwives – Fear speaking openly/retribution | 15 | 11% |
| | | Amnesty - Cannot be supported | 14 | 10% |
| | | Amnesty - Conflicted in opinion | 13 | 10% |
| | | Amnesty – Required to facilitate support | 13 | 10% |
| | | Practicalities – Intervention may be required | 10 | 7% |
| | | Practicalities – There is a duty to report concerns | 5 | 4% |
| | | Amnesty – Required for recovery | 5 | 4% |
| | | Amnesty – may not be possible | 4 | 3% |
| | | Amnesty – May cause distress to others | 3 | 2% |
| | | Amnesty – Conflicted in opinion | 3 | 2% |
| | | Practicalities – Further intervention by management required | 2 | 1% |
| | | Midwives – Ideally should self-report concerns | 2 | 1% |
| | | Amnesty – Automatic if confidentiality/anonymity is afforded | 2 | 1% |
| | | Intervention – Disclaimers may be required | 1 | 1% |
| | | Midwife – Needs support | 1 | 1% |
| | | It would be preferable if the individual were encouraged to self- | 1 | 1% |

| | | | | |
|---|---|---|---|---|
| | | report. | | |
| | | Intervention – Consider emulating the principles of comparable interventions. | 1 | 1% |
| | | Intervention – Warnings may be required | 1 | 1% |
| | | Midwives – Have little existing provision | 1 | 1% |
| | | Midwives – Need support | 1 | 1% |
| | | Midwives – Should self-report | 1 | 1% |
| | | **Total** | **136** | |
| **Item** | **Round** | **Theme** | **Count** | **%** |
| An online intervention designed to support midwives in work-related psychological distress should prioritise prompting platform users automatically to remind them of their responsibilities to their professional codes of conduct. | 1 - Ethical inclusions | Prompting – Helpful inclusion | 18 | 22% |
| | | Prompting – Unhelpful inclusion | 14 | 17% |
| | | Professional codes – adherence a professional responsibility | 14 | 17% |
| | | Midwives – Will already be aware | 11 | 14% |
| | | Prompting - May be harmful | 8 | 10% |
| | | Prompting – Should be done sensitively | 6 | 7% |
| | | Prompting – Need unclear | 3 | 4% |
| | | Conflicted opinion | 2 | 2% |
| | | Prompts – Not supportive | 2 | 2% |
| | | Prompts – adherence to code a pre-condition of use | 1 | 1% |
| | | Prompts – Sensitivity needed | 1 | 1% |
| | | Codes of conduct – important to highlight | 1 | 1% |
| | | **Total** | **81** | |
| **Item** | **Round** | **Theme** | **Count** | **%** |
| An online intervention designed to support midwives in work-related psychological distress should prioritise prompting platform users automatically to seek help, by signposting them to appropriate support | 1 - Ethical inclusions | Signposting to support – A useful inclusion | 36 | 46% |
| | | Signposting to support – Help seeking may be low | 9 | 11% |
| | | Signposting to support – A helpful inclusion | 4 | 5% |
| | | Practicalities – Dependent on the nature of support | 4 | 5% |
| | | Conflicted opinion | 4 | 5% |
| | | Safety is important | 3 | 4% |
| | | Signposting to support – Clarity on method required | 3 | 4% |
| | | Midwives – In control of their own help seeking behaviours | 3 | 4% |
| | | Signposting to support – intervention itself is sufficient | 2 | 3% |
| | | Automatic signposting - Clarity on method required | 2 | 3% |
| | | Prompts - unsuitable | 2 | 3% |
| | | Signposting to support – Support must be high quality | 2 | 3% |
| | | Signposting to support - Could lead to users pathologising symptoms | 1 | 1% |

| | | | | |
|---|---|---|---|---|
| | | Prompting - Consider alternative delivery | 1 | 1% |
| | | Consider using third sector groups and organisations | 1 | 1% |
| | | Practicalities – Needs a personalised tailored response | 1 | 1% |
| | | Intervention – if evidence-based | 1 | 1% |
| | | **Total** | **79** | |
| **Item** | **Round** | **Theme** | **Count** | **%** |
| An online intervention designed to support midwives in work-related psychological distress should prioritise the inclusion of web based videos, multimedia resources and tutorials which explore topics around psychological distress | 1 - Inclusions of Therapeutic Support | Multimedia tutorials – Helpful inclusion | 32 | 40% |
| | | Multimedia – Variety in content presentation useful | 13 | 16% |
| | | Multimedia resources – Helpful inclusion | 8 | 10% |
| | | Multimedia resources – Unhelpful inclusion | 5 | 6% |
| | | Multimedia resources – Require a variety of options | 5 | 6% |
| | | Multimedia resources – Conflicted opinion | 4 | 5% |
| | | Multimedia resources – Evidence based/high quality resources required | 3 | 4% |
| | | Midwives – Greater need for alternative support | 3 | 4% |
| | | Multimedia resources – Benefit dependent upon the nature of resource | 3 | 4% |
| | | Multimedia resources – Benefit dependent upon the nature of distress | 1 | 1% |
| | | Midwives – Feel like failures | 1 | 1% |
| | | Midwives – Material needs to be matched to user needs | 1 | 1% |
| | | Multimedia resources - Unhelpful | 1 | 1% |
| | | Ability - depends upon the content | 1 | 1% |
| | | **Total** | **81** | |
| **Item** | **Round** | **Theme** | **Count** | **%** |
| An online intervention designed to support midwives in work-related psychological distress should prioritise the inclusion of informative multimedia designed to assist midwives to recognise the signs and symptoms of psychological distress | 1 - Inclusions of Therapeutic Support | Informative Multimedia – Helpful inclusion | 37 | 46% |
| | | Midwives – Do not always recognise own distress | 13 | 16% |
| | | Conflicted – Depends upon objectives/content | 7 | 9% |
| | | Informative Multimedia - could lead to inappropriate self-diagnosis | 3 | 4% |
| | | Informative multimedia - helpful inclusion | 3 | 4% |
| | | Informative Multimedia – Unhelpful inclusion | 3 | 4% |
| | | Multimedia – Need a variety of resources | 2 | 2% |
| | | Multimedia - Not required | 2 | 2% |

| | | | | |
|---|---|---|---|---|
| | | Midwives – Help Seeking is important | 2 | 2% |
| | | Informative Multimedia – Unhelpful inclusion | 1 | 1% |
| | | Multimedia - Resources must be high quality/evidence based | 1 | 1% |
| | | Multimedia - Needs to be unique | 1 | 1% |
| | | Midwives – Support is important | 1 | 1% |
| | | Organisational – Distress is a normal response to organisational issues | 1 | 1% |
| | | Informative Multimedia – resource should be clear and simple. | 1 | 1% |
| | | Informative multimedia – To be used in initial engagement | 1 | 1% |
| | | Informative media – Requires a variety of options | 1 | 1% |
| | | Midwives – Do not always recognise own distress | 1 | 1% |
| | | **Total** | **81** | |
| **Item** | **Round** | **Theme** | **Count** | **%** |
| An online intervention designed to support midwives in work-related psychological distress should prioritise the inclusion of multimedia resources which disseminate self-care techniques | 1 - Inclusions of Therapeutic Support | Multimedia self-help resources – Helpful inclusion | 28 | 41% |
| | | Need a variety of resources | 6 | 9% |
| | | Midwives – additional support may be needed | 6 | 9% |
| | | Midwives - do not prioritise self-care | 5 | 7% |
| | | Multimedia self-help resources – Needs to be useful | 3 | 4% |
| | | Multimedia self-help resources – Helpful inclusion | 2 | 3% |
| | | Resource – Must be multiple options available | 2 | 3% |
| | | Multimedia self-help resources – Unhelpful inclusion | 2 | 3% |
| | | Multimedia self-help resources - ease of use important | 2 | 3% |
| | | Neutral | 2 | 3% |
| | | Midwives – Need support and understanding | 2 | 3% |
| | | Midwives – Do not always recognise own distress | 2 | 3% |
| | | Multimedia self-help resources – unhelpful inclusion | 1 | 1% |
| | | Midwives – Need assessment | 1 | 1% |
| | | Midwives – Must be accountable | 1 | 1% |
| | | Midwives – Provision of coaching | 1 | 1% |
| | | Midwives - Meaning of self-care unclear | 1 | 1% |
| | | Resources - Should be simple | 1 | 1% |
| | | Resources - Peer support is useful | 1 | 1% |
| | | **Total** | **69** | |
| **Item** | **Round** | **Theme** | **Count** | **%** |

| | | | | |
|---|---|---|---|---|
| An online intervention designed to support midwives in work-related psychological distress should prioritise the inclusion of multimedia resources which disseminate relaxation techniques | 1 - Inclusions of Therapeutic Support | Relaxation techniques - A helpful inclusion | 18 | 31% |
| | | Relaxation techniques - An unhelpful inclusion | 8 | 14% |
| | | Relaxation techniques – Requires a range of options | 4 | 7% |
| | | Resources – Need a variety of options | 4 | 7% |
| | | Relaxation techniques - A helpful inclusion | 3 | 5% |
| | | Relaxation is a self-care technique | 2 | 3% |
| | | Resources - Must be easy to use | 2 | 3% |
| | | Outside pressures – May inhibit use | 2 | 3% |
| | | Midwives – often feel guilty | 2 | 3% |
| | | Relaxation techniques – May convey the wrong message | 1 | 2% |
| | | Resources - could/should explain theory behind relaxation | 1 | 2% |
| | | Midwives – Can apply their own knowledge | 1 | 2% |
| | | Resources - Need to simple and comprehensive | 1 | 2% |
| | | Relaxation - Limited evidence base | 1 | 2% |
| | | Techniques - Consider mindfulness | 1 | 2% |
| | | Organisational - distress can have organisational cause | 1 | 2% |
| | | Resources – Could emulate comparable alternatives | 1 | 2% |
| | | Resources must be safe to use | 1 | 2% |
| | | Midwives – shortage of support and understanding | 1 | 2% |
| | | Relaxation techniques– benefit dependent on technique used | 1 | 2% |
| | | Need to generate viral content | 1 | 2% |
| | | Midwives – May need additional support | 1 | 2% |
| | | **Total** | **58** | |
| **Item** | **Round** | **Theme** | **Count** | **%** |
| An online intervention designed to support midwives in work-related psychological distress should prioritise the inclusion of mindfulness tutorials and multimedia resources | 1 - Inclusions of Therapeutic Support | Mindfulness - A helpful inclusion | 26 | 44% |
| | | Resources – Must offer a variety of options | 3 | 5% |
| | | Resource – Need a variety of options available | 3 | 5% |
| | | Mindfulness – Meaning unclear | 3 | 5% |
| | | Mindfulness - An unhelpful inclusion | 2 | 3% |
| | | Mindfulness – Neutral opinion | 2 | 3% |
| | | Mindfulness – Degree of evidence | 2 | 3% |
| | | Mindfulness - Midwives may be skeptical | 2 | 3% |
| | | Mindfulness – Conflicted opinion | 2 | 3% |
| | | Midwives – Do not always recognise own distress | 2 | 3% |

| | | | | |
|---|---|---|---|---|
| | | Resources - May send unwanted messages | 1 | 2% |
| | | Midwives – Must be risk assessed | 1 | 2% |
| | | Midwives – Must be encouraged to seek professional help | 1 | 2% |
| | | Resources – Must be accessible | 1 | 2% |
| | | Midwives - should know this technique already | 1 | 2% |
| | | Midwives – Face stigma | 1 | 2% |
| | | Midwives - May not want face to face support | 1 | 2% |
| | | Resources - need to be simple and safe to use | 1 | 2% |
| | | Midwives – other pressures may inhibit use | 1 | 2% |
| | | relaxation - synonymous with mindfulness | 1 | 2% |
| | | Effectiveness - dependent on the degree of distress | 1 | 2% |
| | | Mindfulness - A supportive professional friend would be better | 1 | 2% |
| | | **Total** | **59** | |
| **Item** | **Round** | **Theme** | **Count** | **%** |
| An online intervention designed to support midwives in work-related psychological distress should prioritise the inclusion of Cognitive Behavioral Therapy (CBT) tutorials and multimedia resources. | 1 - Inclusions of Therapeutic Support | CBT tutorials - A helpful inclusion | 18 | 29% |
| | | Intervention - Users may need additional support | 9 | 15% |
| | | CBT tutorials – An unhelpful inclusion | 3 | 5% |
| | | Resources - Need a variety of options to suit all | 2 | 3% |
| | | CBT tutorials – Unclear meaning | 2 | 3% |
| | | Resources - too many interventions may weaken the effect | 2 | 3% |
| | | Midwives – May not access other CBT support | 2 | 3% |
| | | Midwives – Need safety to disclose | 2 | 3% |
| | | Effectiveness – Dependent on evidence and context | 2 | 3% |
| | | Resources - Consider Dialectical Behavioral Therapy (DBT) | 2 | 3% |
| | | Therapies - Evidence base instils confidence | 2 | 3% |
| | | EMDR – Works well | 2 | 3% |
| | | CBT tutorials - reduced evidence base | 1 | 2% |
| | | CBT tutorials - Needs to be easy and safe to use | 1 | 2% |
| | | Midwives – face stigma | 1 | 2% |
| | | Midwives – other pressures may inhibit use | 1 | 2% |
| | | Midwives – May not be convinced of positive effect | 1 | 2% |
| | | Midwives – may need a targeted intervention | 1 | 2% |

| | | | | |
|---|---|---|---|---|
| | | Resources - Need to offer as many options as possible | 1 | 2% |
| | | Midwives – May be impractical | 1 | 2% |
| | | Midwives - Face stigma | 1 | 2% |
| | | CBT tutorials - need to be professional and simple to use | 1 | 2% |
| | | (CBT) tutorials - Question evidence base | 1 | 2% |
| | | Midwives – Need risk assessment | 1 | 2% |
| | | Midwives – Need encouragement to seek help | 1 | 2% |
| | | CBT – Unfamiliar with the therapy | 1 | 2% |
| | | **Total** | **62** | |
| **Item** | **Round** | **Theme** | **Count** | **%** |
| An online intervention designed to support midwives in work-related psychological distress should prioritise the inclusion of information designed to inform midwives where they can access alternative help and support | 1 - Inclusions of Therapeutic Support | Signposted to help and support – A helpful inclusion | 18 | 44% |
| | | Help and support – Need a variety of options available | 6 | 15% |
| | | Help and support – Face to face support preferable | 3 | 7% |
| | | Help and support – Must be evidence based | 2 | 5% |
| | | Midwives – Impaired functioning when distressed | 2 | 5% |
| | | Therapies - EFT (Emotional Freedom Technique) can be useful | 2 | 5% |
| | | Therapies – Suggest peer group debriefing | 2 | 5% |
| | | Alternative help and support – Unclear meaning | 1 | 2% |
| | | Help and support - few resources actually available | 1 | 2% |
| | | Therapies – Must be real and local | 1 | 2% |
| | | Therapies – Too many = Confusion | 1 | 2% |
| | | Help and support – Need a variety of options available | 1 | 2% |
| | | Therapies – Suggest links to local occupational Health resources | 1 | 2% |
| | | **Total** | **41** | |
| **Item** | **Round** | **Theme** | **Count** | **%** |
| An online intervention designed to support midwives in work-related psychological distress should prioritise the inclusion of information designed to inform midwives as to where they can access legal help and advice. | 1 - Inclusions of Therapeutic Support | Legal help and advice - A helpful inclusion | 24 | 60% |
| | | Legal help and advice - An unhelpful inclusion | 4 | 10% |
| | | Legal help and advice - Unnecessary | 4 | 10% |
| | | Legal help and advice – conflicted opinion | 3 | 8% |
| | | Legal help and advice – Question evidence base for this | 1 | 3% |
| | | Legal help and advice - Few resources available | 1 | 3% |
| | | Legal help and advice - Not a priority | 1 | 3% |
| | | Legal Help and advice – Varies | 1 | 3% |

| | | | | |
|---|---|---|---|---|
| | | globally | | |
| | | Legal help and advice - Consider providing personal legal advice | 1 | 3% |
| | | **Total** | **40** | |
| **Item** | **Round** | **Theme** | **Count** | **%** |
| An online intervention designed to support midwives in work-related psychological distress should prioritise giving platform users the ability to share extended personal experiences for other platform users to read | 1 - Inclusions of Therapeutic Support | extended personal experiences - A helpful inclusion | 52 | 60% |
| | | extended personal experiences – Requires moderation | 7 | 8% |
| | | extended personal experiences - Must protect confidentiality | 7 | 8% |
| | | extended personal experiences – conflicted opinion | 6 | 7% |
| | | extended personal experiences – An unhelpful inclusion | 5 | 6% |
| | | extended personal experiences - Must protect anonymity | 3 | 3% |
| | | extended personal experiences – Could be misused | 2 | 2% |
| | | extended personal experiences - Must be optional | 1 | 1% |
| | | extended personal experiences - ethically problematic | 1 | 1% |
| | | Midwives – if conducted within professional codes | 1 | 1% |
| | | extended personal experiences - Requires anonymity | 1 | 1% |
| | | extended personal experiences - Must remain professional | 1 | 1% |
| | | **Total** | **87** | |
| **Item** | **Round** | **Theme** | **Count** | **%** |
| An online intervention designed to support midwives in work-related psychological distress should prioritise the inclusion of a web based peer to peer discussion chat room | 1 - Inclusions of Therapeutic Support | Peer to peer discussion - A helpful inclusion | 31 | 40% |
| | | Peer to peer discussion - Needs moderation | 15 | 19% |
| | | Peer to peer discussion - An unhelpful inclusion | 9 | 12% |
| | | Peer to peer discussion - Risk of unethical use | 7 | 9% |
| | | Peer to peer discussion - Could risk confidentiality/anonymity | 3 | 4% |
| | | Peer to peer chatroom - May not be used | 2 | 3% |
| | | Peer to peer chatroom - Requires rules and standards | 2 | 3% |
| | | Peer to peer discussion - May risk anonymity/confidentiality | 2 | 3% |
| | | Peer to peer chatroom - Requires high volume site traffic | 1 | 1% |
| | | Peer to peer chatroom - May require trained supporters | 1 | 1% |
| | | Effectiveness – Depends upon help seeking behaviour | 1 | 1% |
| | | Professional – Legal/Regulatory obligations | 1 | 1% |

| | | | | |
|---|---|---|---|---|
| | | Midwives - May need local chat rooms | 1 | 1% |
| | | Peer to peer discussion - Should be an optional choice | 1 | 1% |
| | | Peer to peer discussion - May be local variations | 1 | 1% |
| | | **Total** | **78** | |
| **Item** | **Round** | **Theme** | **Count** | **%** |
| An online intervention designed to support midwives in work-related psychological distress should prioritise giving platform users the ability to communicate any work or home based subjects of distress | 1 - Inclusions of Therapeutic Support | Discussions re: work or home based subjects of distress – A helpful inclusion | 13 | 26% |
| | | Discussions re: work or home based subjects of distress - intertwined | 13 | 26% |
| | | Discussions – unhelpful inclusion | 8 | 16% |
| | | Discussions - Should be kept separate | 4 | 8% |
| | | Priority – Depends upon the context | 3 | 6% |
| | | Discussions – Require moderation | 2 | 4% |
| | | Discussions re: work or home based subjects of distress – An unhelpful inclusion | 2 | 4% |
| | | Discussions - uncontrollable | 1 | 2% |
| | | Discussions – May risk anonymity/confidentiality | 1 | 2% |
| | | Discussions – Require support | 1 | 2% |
| | | Discussions re: work or home based subjects of distress – chaotic | 1 | 2% |
| | | Discussions – Require moderation | 1 | 2% |
| | | **Total** | **50** | |
| **Item** | **Round** | **Theme** | **Count** | **%** |
| An online intervention designed to support midwives in work-related psychological distress should prioritise an interface which does not resemble NHS, employer or other generic healthcare platforms | 1 - Intervention design and practical inclusions | Resemblance – Should be authority neutral | 28 | 56% |
| | | Priority – user friendliness | 5 | 10% |
| | | Resemblance – Should be authority based | 3 | 6% |
| | | Resemblance – Not important | 3 | 6% |
| | | Question – relevance unclear | 2 | 4% |
| | | Midwives – Fearful of detection | 2 | 4% |
| | | Resemblance - Variants on a global scale | 1 | 2% |
| | | This would not matter if the intervention was clearly independent. | 1 | 2% |
| | | Question – Cannot answer | 1 | 2% |
| | | Intervention – Needs support of authorities | 1 | 2% |
| | | Prioritise – visually safe space | 1 | 2% |
| | | Intervention –confidentiality and anonymity important | 1 | 2% |
| | | Intervention - Consider analysing feedback | 1 | 2% |
| | | **Total** | **50** | |

| **Item** | **Round** | **Theme** | **Count** | **%** |
|---|---|---|---|---|
| An online intervention designed to support midwives in work-related psychological distress should prioritise a simple, anonymised email login procedure which allows for continued contact and reminders which may prompt further platform usage | 1 - Intervention design and practical inclusions | Anonymised email login procedure - A helpful inclusion | 19 | 36% |
| | | Anonymised email login procedure - An unhelpful inclusion | 13 | 25% |
| | | Priorities – A user-friendly intervention | 9 | 17% |
| | | Prompting – A helpful inclusion | 4 | 8% |
| | | Prompting – An unhelpful inclusion | 4 | 8% |
| | | Anonymised email login procedure – must be optional | 1 | 2% |
| | | Anonymised email login procedure – Unsure of alternatives | 1 | 2% |
| | | Midwives – May require alternative support | 1 | 2% |
| | | Confidentiality must be upheld | 1 | 2% |
| | | **Total** | **53** | |
| **Item** | **Round** | **Theme** | **Count** | **%** |
| An online intervention designed to support midwives in work-related psychological distress should prioritise an automated moderating system where 'key words' would automatically initiate a moderated response | 1 - Intervention design and practical inclusions | 'key words' initiating a moderated response – A helpful inclusion | 15 | 22% |
| | | 'key words' initiating a moderated response – an unhelpful inclusion | 13 | 19% |
| | | Question – Need to know more | 11 | 16% |
| | | 'key words' initiating a moderated response – May not be adequate | 8 | 12% |
| | | 'key words' initiating a moderated response – Moderation is required | 6 | 9% |
| | | 'key words' initiating a moderated response – conflicted | 5 | 7% |
| | | Midwives – Must be protected from suicide | 3 | 4% |
| | | Midwives – Need to be risk assessed | 2 | 3% |
| | | 'key words' initiating a moderated response –must be supportive in nature | 1 | 1% |
| | | 'key words' initiating a moderated response – Confusing | 1 | 1% |
| | | 'key words' initiating a moderated response – Must be sophisticated | 1 | 1% |
| | | 'key words' initiating a moderated response – Moderation = high maintenance | 1 | 1% |
| | | **Total** | **67** | |
| **Item** | **Round** | **Theme** | **Count** | **%** |
| An online intervention designed to support midwives in work-related psychological distress should prioritise mobile device compatibility for platform users | 1 - Intervention design and practical inclusions | Mobile device compatibility – High priority | 35 | 73% |
| | | Mobile device compatibility – Must work | 6 | 13% |
| | | Midwives – Require support | 2 | 4% |
| | | Mobile device compatibility - Unhelpful | 1 | 2% |
| | | mobile device compatibility – Neutrality important | 1 | 2% |

| | | | | |
|---|---|---|---|---|
| | | mobile device compatibility – Must be secure | 1 | 2% |
| | | Midwives – may not seek alternative support | 1 | 2% |
| | | Intervention - Risky | 1 | 2% |
| | | **Total** | **48** | |
| **Item** | **Round** | **Theme** | **Count** | **%** |
| An online intervention designed to support midwives in work-related psychological distress should prioritise amnesty for all platform users in that they will not be referred to any law enforcement agencies, their employer or regulatory body for either disciplinary or investigative proceedings in any case. | 2 - Items which did not achieve a consensus of opinion within the first round of questioning. | Amnesty – Required for open and honest disclosure | 27 | 26% |
| | | Amnesty - Cannot be given in all cases | 25 | 24% |
| | | Midwives - Fear retribution | 11 | 11% |
| | | Amnesty - Conflicted in opinion | 10 | 10% |
| | | Amnesty - Required for change/Help seeking | 9 | 9% |
| | | Amnesty - Conflicted in opinion | 5 | 5% |
| | | Amnesty can enable resolution of situations | 4 | 4% |
| | | Amnesty - A helpful inclusion | 3 | 3% |
| | | Midwives - Have little support | 2 | 2% |
| | | Intervention - Requires disclaimer policies | 2 | 2% |
| | | Amnesty – Cannot be given in any circumstances | 1 | 1% |
| | | Amnesty - required for privacy | 1 | 1% |
| | | Amnesty - Difficult to moderate | 1 | 1% |
| | | Amnesty - An unhelpful inclusion | 1 | 1% |
| | | Question - Meaning unclear | 1 | 1% |
| | | **Total** | **103** | |
| **Item** | **Round** | **Theme** | **Count** | **%** |
| An online intervention designed to support midwives in work-related psychological distress should prioritise prompting platform users automatically to remind them of their responsibilities to their professional codes of conduct. | 2 - Items which did not achieve a consensus of opinion within the first round of questioning. | Prompting professional codes - A helpful inclusion | 21 | 31% |
| | | Prompting professional codes - An unhelpful inclusion | 16 | 24% |
| | | Prompting professional codes - Ethically essential | 9 | 13% |
| | | Prompting professional codes - Alternative approach required | 7 | 10% |
| | | Midwives - Already aware of codes - Not required | 4 | 6% |
| | | Prompting professional codes - Not the purpose of the intervention | 3 | 4% |
| | | Midwives - Duty of care should be priority | 2 | 3% |
| | | Codes - Inadequate | 1 | 1% |
| | | Midwives - Need support | 1 | 1% |
| | | Midwives - Should remain professional even in distress | 1 | 1% |
| | | Midwives - If needing reminders, should not be working | 1 | 1% |
| | | Prompting professional codes - Requires sensitivity | 1 | 1% |
| | | **Total** | **67** | |

| Item | Round | Theme | Count | % |
|---|---|---|---|---|
| An online intervention designed to support midwives in work-related psychological distress should prioritise the inclusion of information designed to inform midwives as to where they can access legal help and advice. | 2 - Items which did not achieve a consensus of opinion within the first round of questioning. | Information, legal help and advice - A helpful inclusion | 32 | 60% |
| | | Midwives - Need support | 6 | 11% |
| | | Intervention - A range of options should be made available | 5 | 9% |
| | | Information, legal help and advice - An unhelpful inclusion | 4 | 8% |
| | | Question - Meaning unclear | 2 | 4% |
| | | Information, legal help and advice - Can be found elsewhere | 2 | 4% |
| | | Midwives - Fear retribution | 1 | 2% |
| | | Midwives - Blame themselves | 1 | 2% |
| | | **Total** | **53** | |
| **Item** | **Round** | **Theme** | **Count** | **%** |
| An online intervention designed to support midwives in work-related psychological distress should prioritise giving platform users the ability to share extended personal experiences for other platform users to read. | 2 - Items which did not achieve a consensus of opinion within the first round of questioning. | Sharing extended personal experiences - A helpful inclusion | 41 | 58% |
| | | Sharing extended personal experiences - Moderation required | 11 | 15% |
| | | Sharing extended personal experiences- An unhelpful inclusion | 6 | 8% |
| | | Sharing extended personal experiences - Should be optional | 6 | 8% |
| | | Sharing extended personal experiences - Risky | 3 | 4% |
| | | Sharing extended personal experiences - Undecided | 2 | 3% |
| | | Sharing extended personal experiences - effect may be context dependent | 2 | 3% |
| | | **Total** | **71** | |
| **Item** | **Round** | **Theme** | **Count** | **%** |
| An online intervention designed to support midwives in work-related psychological distress should prioritise the inclusion of a web based peer to peer discussion chat room. | 2 - Items which did not achieve a consensus of opinion within the first round of questioning. | Discussion chat room - A helpful inclusion | 37 | 51% |
| | | Discussion chat room- An unhelpful inclusion | 11 | 15% |
| | | Discussion chat room - Moderation required | 10 | 14% |
| | | Discussion chat room - Risky | 7 | 10% |
| | | Discussion chat room - More information required | 6 | 8% |
| | | Discussion chat room- Challenging to facilitate | 1 | 1% |
| | | Discussion chat room - Consider additional features | 1 | 1% |
| | | **Total** | **73** | |
| **Item** | **Round** | **Theme** | **Count** | **%** |
| An online intervention designed to support midwives in work-related psychological distress should prioritise giving platform users | 2 - Items which did not achieve a consensus of opinion within the first round of questioning. | Communicating any work or home based subjects of distress - A helpful inclusion | 15 | 28% |
| | | Communicating any work or home based subjects of distress - Both subjects intertwined | 16 | 30% |
| | | Communicating any work or home | 9 | 17% |

| | | | | |
|---|---|---|---|---|
| the ability to communicate any work or home based subjects of distress. | | based subjects of distress - An unhelpful inclusion | | |
| | | Communicating any work or home based subjects of distress -Inevitable | 7 | 13% |
| | | Communicating any work or home based subjects of distress - Requires moderation | 2 | 4% |
| | | Communicating any work or home based subjects of distress - Risk of breaching confidentiality | 1 | 2% |
| | | Communicating any work or home based subjects of distress - Undecided | 1 | 2% |
| | | Communicating any work or home based subjects of distress - Difficult to engage whilst at work | 1 | 2% |
| | | Communicating any work or home based subjects of distress - discussions need to remain workplace based | 1 | 2% |
| | | Communicating any work or home based subjects of distress - Consider links to outside agencies | 1 | 2% |
| | | **Total** | **54** | |
| **Item** | **Round** | **Theme** | **Count** | **%** |
| An online intervention designed to support midwives in work-related psychological distress should prioritise an interface which does not resemble NHS, employer or other generic healthcare platforms. | 2 - Items which did not achieve a consensus of opinion within the first round of questioning. | Online intervention interface - Should not resemble NHS, employer or other generic healthcare platforms | 20 | 38% |
| | | Midwives - May not engage if they fear organisational involvement | 11 | 21% |
| | | Online intervention interface - Should look professional | 8 | 15% |
| | | Online intervention interface - Should prioritise usability | 6 | 11% |
| | | Conflicted opinion | 3 | 6% |
| | | Midwives - Fear bringing the profession into disrepute | 1 | 2% |
| | | Online intervention interface - Should not resemble NHS, employer or other generic healthcare platforms | 1 | 2% |
| | | Intervention - Should be a safe haven | 1 | 2% |
| | | Intervention - Must appear to be for midwives only | 1 | 2% |
| | | Online intervention interface - Options should be researched | 1 | 2% |
| | | **Total** | **53** | |
| **Item** | **Round** | **Theme** | **Count** | **%** |
| An online intervention designed to support midwives in work-related | 2 - Items which did not achieve a consensus of opinion within the | Anonymised email login procedure - A helpful inclusion | 19 | 40% |
| | | Anonymised email login procedure - Ease of use a priority | 8 | 17% |

| | | | | |
|---|---|---|---|---|
| psychological distress should prioritise a simple, anonymised email login procedure which allows for continued contact and reminders which may prompt further platform usage. | first round of questioning. | Anonymised email login procedure - An unhelpful inclusion | 4 | 9% |
| | | Anonymised email login procedure - Can be used to intervene | 3 | 6% |
| | | Anonymised email login procedure - Risky | 3 | 6% |
| | | Anonymised email login procedure - Anonymity may not be possible | 2 | 4% |
| | | Undecided | 2 | 4% |
| | | Anonymity - Essential | 2 | 4% |
| | | Anonymised email login procedure - Security a priority | 2 | 4% |
| | | Anonymised email login procedure - Should be optional | 2 | 4% |
| | | **Total** | **47** | |
| **Item** | **Round** | **Theme** | **Count** | **%** |
| An online intervention designed to support midwives in work-related psychological distress should prioritise an automated moderating system where 'key words' would automatically initiate a moderated response. | 2 - Items which did not achieve a consensus of opinion within the first round of questioning. | An automated moderating system - A helpful inclusion | 16 | 31% |
| | | An automated moderating system - An unhelpful inclusion | 9 | 18% |
| | | An automated moderating system - Meaning unclear | 8 | 16% |
| | | An automated moderating system - Must be appropriate | 6 | 12% |
| | | Undecided | 5 | 10% |
| | | Moderation - Should be a priority | 3 | 6% |
| | | Moderation - Should be a human response | 2 | 4% |
| | | Intervention - Must be a safe space | 1 | 2% |
| | | An automated moderating system - Should allow users to flag concerns | 1 | 2% |
| | | **Total** | **51** | |
| **Item** | **Round** | **Theme** | **Count** | **%** |
| An online intervention designed to support midwives in work-related psychological distress should prioritise an interface which resembles and works in a similar way to current popular and fast pace social media channels: e.g. Facebook | 2 - New items for consideration | An interface which resembles and works in a similar way to current popular and fast pace social media channels - Helpful | 20 | 37% |
| | | An interface which resembles and works in a similar way to current popular and fast pace social media channels - unhelpful | 15 | 28% |
| | | Usability should be the priority | 11 | 20% |
| | | An interface which resembles and works in a similar way to current popular and fast pace social media channels - Undecided | 6 | 11% |
| | | Question - Misunderstood | 1 | 2% |
| | | Usability should be the priority | 1 | 2% |
| | | **Total** | **54** | |
| **Item** | **Round** | **Theme** | **Count** | **%** |
| An online intervention designed to support midwives in work-related | 2 - New items for consideration | The inclusion of midwives from around the world - Helpful | 24 | 42% |
| | | The inclusion of midwives from around the world - unhelpful | 14 | 25% |

| | | | | |
|---|---|---|---|---|
| psychological distress should prioritise the inclusion of midwives from around the world | | The inclusion of midwives from around the world - Challenging to facilitate | 10 | 18% |
| | | The inclusion of midwives from around the world - Undecided | 6 | 11% |
| | | The inclusion of midwives from around the world - Could be made fit for purpose | 2 | 4% |
| | | The inclusion of midwives from around the world - Challenging to facilitate | 1 | 2% |
| | | **Total** | **57** | |
| **Item** | **Round** | **Theme** | **Count** | **%** |
| An online intervention designed to support midwives in work-related psychological distress should prioritise proactive moderation (i.e, users are able to block unwanted content and online postings are 'pre-approved') | 2 - New items for consideration | Proactive moderation - Helpful | 24 | 41% |
| | | Proactive moderation - Unhelpful | 11 | 19% |
| | | Proactive moderation - Must be tailored to suit context | 11 | 19% |
| | | Moderation - essential | 9 | 16% |
| | | Proactive moderation - Meaning unclear | 2 | 3% |
| | | Midwives - Able to self-moderate | 1 | 2% |
| | | **Total** | **58** | |
| **Item** | **Round** | **Theme** | **Count** | **%** |
| An online intervention designed to support midwives in work-related psychological distress should prioritise reactive moderation (i.e., users are able to report inappropriate content to a system moderator for removal) | 2 - New items for consideration | Reactive moderation - Helpful | 24 | 67% |
| | | Reactive moderation - Design challenges | 7 | 19% |
| | | Reactive moderation - Unhelpful | 5 | 14% |
| | | **Total** | **36** | |
| **Item** | **Round** | **Theme** | **Count** | **%** |
| An online intervention designed to support midwives in work-related psychological distress should prioritise 24/7 availability of the platform | 2 - New items for consideration | 24/7 availability - Helpful | 37 | 90% |
| | | Midwives - Have no time | 2 | 5% |
| | | 24/7 availability - Undecided | 1 | 2% |
| | | Midwives - Need confidentiality | 1 | 2% |
| | | **Total** | **41** | |
| **Item** | **Round** | **Theme** | **Count** | **%** |
| An online intervention designed to support midwives in work-related | 2 - New items for consideration | Simple user assessment - Helpful | 25 | 56% |
| | | Simple user assessment - Context for use required | 14 | 31% |
| | | Simple user assessment - | 3 | 7% |

| | | | | |
|---|---|---|---|---|
| psychological distress should prioritise the implementation of an initial simple user assessment using a psychological distress scale to prompt the user to access the most suitable support available | | Undecided | | |
| | | Simple user assessment - unhelpful | 2 | 4% |
| | | Simple user assessment - Should be optional | 1 | 2% |
| | | **Total** | **45** | |
| **Item** | **Round** | **Theme** | **Count** | **%** |
| An online intervention designed to support midwives in work-related psychological distress should prioritise the gathering of anonymised data and concerns from users, only with explicit permission, so that trends and concerns may be highlighted at a national level. | 2 - New items for consideration | The gathering of anonymised data and concerns - Helpful | 32 | 64% |
| | | The gathering of anonymised data and concerns - Unhelpful | 11 | 22% |
| | | The gathering of anonymised data and concerns - Requires ethical consideration | 3 | 6% |
| | | Midwives - Require anonymity | 2 | 4% |
| | | The gathering of anonymised data and concerns - Undecided | 1 | 2% |
| | | Midwives - Require confidentiality | 1 | 2% |
| | | **Total** | **50** | |
| **Item** | **Round** | **Theme** | **Count** | **%** |
| An online intervention designed to support midwives in work-related psychological distress should prioritise access for a midwife's friends and family members | 2 - New items for consideration | Access for a midwife's friends and family - Unhelpful | 17 | 36% |
| | | Access for a midwife's friends and family - Ethical considerations must be recognized | 10 | 21% |
| | | Access for a midwife's friends and family - Undecided | 7 | 15% |
| | | Access for a midwife's friends and family - Could require a separate, designated area | 6 | 13% |
| | | Access for a midwife's friends and family - Helpful | 4 | 9% |
| | | Access for a midwife's friends and family - Need more information | 3 | 6% |
| | | **Total** | **47** | |
| **Item** | **Round** | **Theme** | **Count** | **%** |
| An online intervention designed to support midwives in work-related psychological distress should prioritise the following up and identification of those at risk | 2 - New items for consideration | The following up and identification of those at risk - Helpful | 23 | 42% |
| | | The following up and identification of those at risk - Challenging | 14 | 25% |
| | | The following up and identification of those at risk - Unhelpful | 9 | 16% |
| | | The following up and identification of those at risk - Undecided | 6 | 11% |
| | | The following up and identification of those at risk - Beyond the scope of this project | 3 | 5% |

| | | Total | 55 | |
|---|---|---|---|---|
| **Item** | **Round** | **Theme** | **Count** | **%** |
| An online intervention designed to support midwives in work-related psychological distress should prioritise the provision of a general statement about professional codes of conduct and the need for users to keep in mind their responsibilities in relation to them | 2 - New items for consideration | A general statement about professional codes of conduct - Helpful | 15 | 38% |
| | | A general statement about professional codes of conduct - Unhelpful | 8 | 21% |
| | | Achieving consensus - Frustrated by survey questions | 5 | 13% |
| | | Midwives - Already aware of codes | 4 | 10% |
| | | A general statement about professional codes of conduct - Requires sensitivity | 3 | 8% |
| | | A general statement about professional codes of conduct - Must be applicable | 2 | 5% |
| | | Online community - Should develop its own codes of conduct | 2 | 5% |
| | | **Total** | **39** | |
