# Supplementary material for: Achieving Consensus for the Design and Delivery of an Online Intervention to Support Midwives in Work-Related Psychological Distress: Results From a Delphi Study
Source: JMIR Ment Health. 2016 Jul 12;3(3):e32. doi: 10.2196/mental.5617 (PMC4961877; doi:10.2196/mental.5617)
Supplement: Multimedia Appendix 5 [file mental_v3i3e32_app5.pdf]

Delphi Study Analysis Report: Delphi Study to achieve Consensus in the Development of an Online Intervention Designed to Effectively Support Midwives in Work related Psychological Distress.

Introduction

During the summer of 2015, experts in the fields of midwifery, midwifery education, psychology, psychological trauma, psychiatry, health care services, research, therapies, patient experience and staff experience were recruited to join a Delphi panel. This expert Delphi panel was recruited in order to achieve consensus in the development of an online intervention designed to support midwives in work-related psychological distress. 185 participants in total were recruited via social media channels and through the academic literature. Many had approached the research team in advance to express an interest in joining this study. In the autumn of 2015, of the 185 participants recruited, 66 participants (35%) completed the first round of questioning. Responses to this first round of questioning are reported here. Please note that spelling mistakes have been corrected accordingly.

This data will inform the development of this second Delphi round. The purpose of the second round of questioning is to allow study participants an opportunity to revise or rethink their original responses in light of the group response, for those items for which consensus has not been reached. Consensus will be reached if 60% of respondents are within 2 adjacent response points on the 7-point scale. This opportunity for a changed response may or may not result in consensus. New questions will also be asked within this second round of questioning as a result of this analysis of round 1 responses.

1

All participants gave their full consent so that the research team could verify that participants fitted the inclusion criteria, had understood the participant information in full, had given their consent for their anonymised quotes and results to be used for publication, and had given their full consent to participation.

2

An online intervention designed to support midwives in work-related psychological distress should prioritize confidentiality for all platform users and service users in all matters of discussion.

2.1 Confidentiality for all platform users and service users in all matters of discussion

Rank value	Option	Count
1	Not a priority	0
2	Low priority	0
3	Somewhat a priority	3
4	Neutral	2
5	Moderate priority	1
6	High priority	6
7	Essential priority	54

Mean rank	6.61
Variance	1.0
Standard Deviation	1.0
Lower Quartile	7.0
Upper Quartile	7.0

Consensus Achieved = Yes (High/Essential Priority) 90.9%

Minimum score = Not a priority/Low priority 0 (0%)

Maximum score = Essential Priority 54 (81.8%)

Confidentiality for all platform users and service users in all matters of discussion

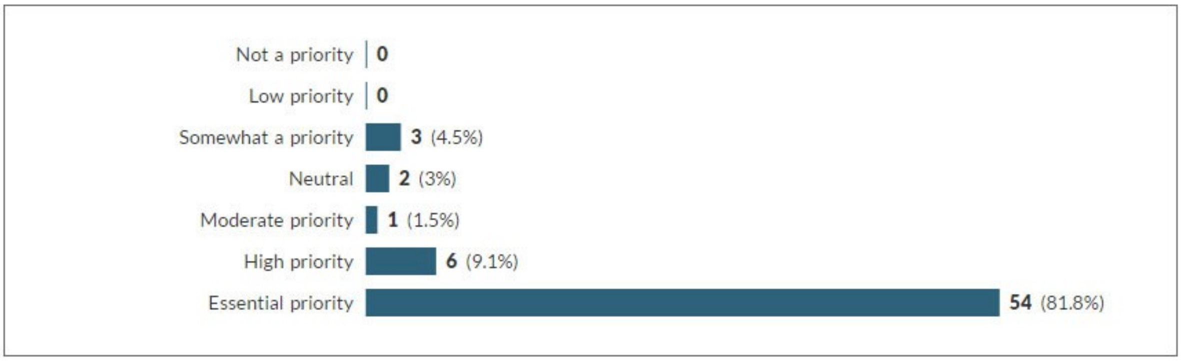

Thematic analysis of open text responses

Why did you choose this rating of priority?

Comment	Themes assigned to
[This can be decided by the individuals concerned][3].	3
[Sharing of personal information and thoughts should be the user's choice to share.][3]	3
[Cos folk might hold back on their responses without this][1].	1
Many midwifery forums have [concerns about posting on line for fear of retribution by the NMC/public] [1, 2]. Their traffic has reduced in recent years and therefore the support that they provided has now reduced because of this.	1, 2
[To enable midwives to be completely open][1]	1
[People unlikely to be honest if they think information may be shared][1].	1
[Individuals could be concerned about other being aware] [12, 7].	12, 7
[it is essential that staff can share experiences and feelings in an open and honest way][1] [without fear of reprisals][2]	1, 2
Psychological distress may be attributed to a specific event involving a client so [important to protect their identity][4] whilst also [maintaining the professional reputation of midwives and other healthcare professionals] [9] involved who [may be experiencing distress] [12]	4, 9, 12
[People need to be able to be honest] [1] [if they know information will be shared they may hold back] [1] but maybe a [choice to disclose who they are if they wish?][3]	1, 1, 3
[Depends on the context] [10]. Confidentiality between service users [should work as an ethical codex] [4], but who/how should enforce this?	10, 4
[people will be ready to open up if confidentiality is guaranteed][1]	1
[To ensure that answers given of a sensitive and personal nature are assured] [12]. Also confidentiality reduces bias and gives greater validity and reliability to the findings of this study.	12
[In any research confidentiality beheld in high esteem][4]	4
[it will help midwives to share their fillings more freely and with openness][1]	1
[People may not utilise the service if they think they can be identified or 'outed' in terms of feeling distressed][1, 7]	1, 7
[Some platform users may want to share experience][1] in a group setting	1
I believe some [midwives would be fearful of people finding out][2] [they were finding it difficult to cope][6] and would therefore [seek anonymity to feel safe] [12] to access support	2, 6, 12
[Midwives have professional responsibilities to not break confidentiality] [4], and [can't be supported fully unless this requirement is met] [11]. They [won't be able to concentrate on getting support and recovering from distress unless this is covered] [11].	4, 11, 11

[The user needs to feel safe][12]	12
[Confidentiality is the cornerstone to enabling an individual to freely and expansively express their concerns, emotions, thoughts etc.][1, 11]	1, 11
[it is the only way people will feel able to open up][1]	1
[It is important to maintain confidentiality for all who could be involved to protect against further stress] [12, 4].	12, 4
[Participation would be determined by confidentiality statements][12]	12
[Midwives need to feel safe] [12] about exploring options for their wellbeing without [concern that colleagues or mother being able to check up on them][2, 7] and make possible negative judgements on them as individuals or on their practice	12, 2, 7
[Important to have a confidential 'space'] [11] if that's what people need at that time otherwise there could be [fear of criticism and recriminations] [2] which might lead to further stress. [I just don't believe people would use it if not confidential] [11]. People need to be able to trust in order to find support. [Confidentiality is an important part of making a safe place and fostering trust][9]	11, 2, 11, 9
[Midwives need to feel safe] [12] to explore these vulnerable issues and [confidentiality is an important part of creating a safe space] [12]	12, 12
[1)An essential expectation and right][4]<br>[2) individuals may fear consequences and being identified][11]	4, 11
I believe that this is an area which has not been previously addressed. There is evidence that work related psychological distress is very disabling and currently [midwives have none or very little support][13]	13
[It will enable openness and honesty in discussions][1]	1
[To be able to express myself honestly then confidentiality is paramount][1]	1
This is a nice idea and [could be achieved in a one- on- one session if this were available] [10] but [the idea of posting something confidentially on the web is surely impossible?][8] Once it's out, it's out.	8, 10
[People tend to be more willing to discuss own psychological distress when information is confidential][1]	1
[If it is not confidential people asking for help will not be honest] [1] and may [need to offload about work situations] [1]. [It will also protect those they work with] [4]	1, 1, 4
[Midwives are unlikely to be able to consult faceless emotional support regarding professional issues if it is not confidential][1]. [Trust is an issue] [12].	1, 12
Work related psychological distress is related to other issues such as bullying and loss of Trust. [Midwives need to feel that they can share their experiences frankly] [1] and [without fear of reprisal] [2]; [this necessitates confidentiality] [1]. [However, midwives may decide to whistle blow and voluntarily forgo confidentiality][3].	1, 2, 1, 3, 10
[To enable trust in the intervention] [12] to obviously providing the scope and limitations of the confidentiality are explained.	12
[This may encourage use of the service][11]	11
[We must guard against any possible discrimination against someone engaging with the tool] [12]. [However, in extreme cases, intervention may be needed to properly support a midwife with profound distress][5].	5, 12
[People are more likely to use it if confidential][1, 11] [due to employer and regulator expectations and rules][2]	1, 11, 2
Has been part of midwifery education that they should be able to cope with all their profession exposes them to hence [can feel shame if not managing][6]	6
[This should be the same as if you were in a face to face intervention where confidentiality is a paramount][4].	4
As it is online it [must always be 100% confidential] [4]. A computer is not a person and able to give feedback or take part in a decision making process	4
[In order to create a safe space] [12]	12
[To enable full disclosure][1]	1

[don't think people will use it if they don't believe it is confidential][11]		11
For some participants this will be a high priority, [others may want to share more] [3]. [If safeguarding issues arise, action may be needed][5].		3, 5
[If the online intervention is purely focused on the individual] [10] then yes [confidentiality is key] [11] - however peer support can be powerful & if this is a component then confidentiality may need further consideration.		11, 10
	**Theme**	**Number of times categorised**
1.	Confidentiality – Required for open and honest disclosure	23
2.	Midwives - Fear retribution	6
3.	Confidentiality – Decided by user	5
4.	Confidentiality – for third parties	9
5.	Midwives - May need further support/ intervention	2
6.	Midwives – Feel shame if not managing	2
7.	Confidentiality – Needed to avoid public identification	3
8.	Confidentiality – Not possible online	1
9.	Confidentiality - Needed to protect the reputation of the profession	2
10.	Confidentiality – Context dependent	4
11.	Confidentiality – Essential criterion for provision of support	10
12.	Midwives – Need to feel safe	10
13.	Midwives – Have little existing provision	1

Do you have any additional comments you would like to share?

**Comment**	**Themes assigned to**
however, [there needs to be clarification of legal / professional obligations][1] in terms of dangerous practice highlighted	1
there can be consequences to posting experiences on existing social media groups as not always closed /confidential groups - and [may lead to disciplinary action][2]	2
NO	0
No	0
Online confidentiality is hard to guarantee, [how will you do that?][3]	3
In a study we have undertaken in Australia [confidentiality was rated very highly][4] (not yet published)	4
[Perhaps a statement saying that no identifying information will be collected including IP addresses?][3]	3
[Is important as it gives the respondent "free" space][4]	4
I have used a service which was independent of the workplace/employer but supplied by the employer. [If I thought that my discussions with the counsellor would go back to my employer I would not have engaged in the process of counselling] [6] with them. [I needed	6, 5

assistance to work through the issues and having complete confidence that any discussion was confidential][5].		
[Can an in built 'alert' highlight importance of seeking support…?][3]		3
	**Theme**	**Number of times categorised**
1.	Professional – Legal/Regulatory obligations	1
2.	Midwives – Fear consequences	1
3.	Confidentiality – Not possible online	1
4.	Confidentiality – High priority	2
5.	Midwives – Need reassurance	1
6.	Confidentiality - required to promote disclosure	1

3

An online intervention designed to support midwives in work-related psychological distress should prioritise anonymity for all platform users and service users in all matters of discussion.

3.1 Anonymity for all platform users and service users in all matters of discussion

Rank value	Option	Count
1	Not a priority	0
2	Low priority	1
3	Somewhat a priority	3
4	Neutral	2
5	Moderate priority	4
6	High priority	17
7	Essential priority	39

Mean rank	6.27
Variance	1.35
Standard Deviation	1.16
Lower Quartile	6.0
Upper Quartile	7.0

Consensus Achieved = Yes (High Priority) 84.9%

Minimum score = Not a priority 0 (0%)

Maximum score = Essential Priority 39 (59.1%)

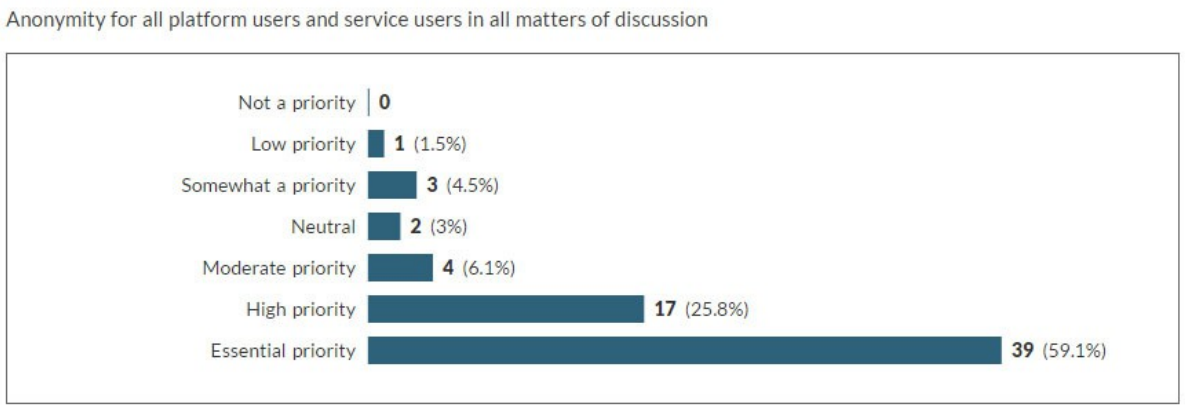

Thematic analysis of open text responses

Why did you choose this rating of priority?

Comment	Themes assigned
See the answer above [(This can be decided by the individuals concerned.)][4] -and [when there is real trauma the psychological response is more important that covering everyone's backside][7].	4, 7
[Without anonymity users may well be put off using the service][3] in ties of dire need.	3
Essentially the same reason as (1) (cos folk might hold back on their responses without this)[1]	1
Same answer as above (Many midwifery forums have [concerns about posting on line for fear of retribution][6, 1] by the NMC/public. Their traffic has reduced in recent years and therefore the support that they provided has now reduced because of this.)	6, 1
[People will feel more relaxed about being completely open about issue that is worrying them][1]	1
[see above re caveat about legal/professional need to report dangerous practice or abuse][9]	9
Same Question as 2? [(individuals could be concerned about other being aware)][1]	1
[users need to be protected from identification][5] - this [will facilitate more open and honest discussion][1]	1, 5
As above (Psychological distress may be attributed to a specific event involving a client so [important to protect their identity][10] whilst [also maintaining the professional reputation of midwives and other healthcare professionals][10] involved who may be experiencing distress)][3]	10, 10 , 3
again [people need to be honest][1] but have [choice to disclose who they are if they wish][4]	1, 4
Have [anonymity will hopefully encourage more people to access the platform][1]	1
Same as above answer:<br>[To ensure that answers given of a sensitive and personal nature are assured][5]. Also confidentiality reduces bias and gives greater validity and reliability to the findings of this study.	5
In other to avoid biases	0
[it will help midwives to share their fillings more freely and with openness][1]	1
[People generally may feel more able to 'bare their souls' if they can't be recognised][1]	1
As above (Some platform [users may want to share experience][1] in a group setting)	1
same reasons as above (I believe [some midwives would be fearful of people finding out they were finding it difficult to cope][6] and [would therefore seek anonymity to feel safe to access support)][1] & (In a study we have undertaken in Australia [confidentiality was rated very highly (not yet published))][7]	6, 1, 7
[I don't see how you'd separate this from confidentiality][1] - see previous answers. ([Midwives have professional responsibilities to not break confidentiality][9], and [can't be supported fully unless this requirement is met][3]. [They won't be able to concentrate on getting support and recovering from distress unless this is covered.)][3]	9, 3, 3, 12

as above [(The user needs to feel safe.)][5] & (Perhaps a statement saying that no identifying information will be collected including IP addresses?)[11]	5, 11
[Anonymity goes hand in hand with confidentiality][12] in the sense of [enabling the individual to freely express themselves][1]	12, 1
[You need to be able to signpost those users who are struggling and need further intervention][7]. [Anonymising would prevent this][2]	2, 7
Same as previous answer ([Midwives need to feel safe about exploring options for their wellbeing][5] without [concern that colleagues or mother being able to check up on them and make possible negative judgements on them as individuals or on their practice)][6]	5, 6
[It is important to have to option to be anonymous][3]. [I have not put it as essential as I wonder if it may be necessary and possible to build in a system which can respond to circumstances when anonymity is not appropriate][4,9]. Perhaps the platform administrator needs to be able to trace who people are for example. But this should only be utilised in rare and exceptional circumstances. I am thinking about [a situation when there appears to be a serious risk to health, either for the platform user or another individual] [2].	3, 4, 9, 2
[again safe space] [5]	5
as above (1) [An essential expectation and right] [3, 10]<br>2) individuals may fear consequences and being identified)[6]	3, 10, 6
sorry I find this a little confusing as it is [very similar to the above question][12] therefore I have scored the same	12
[Complete anonymity might mean that serious risk to service users goes unchecked][8, 9]	8, 9
[To encourage openness][1, 3]	1, 3
[To be able to speak without judgement of role] [1, 6] etc. . . .	1, 6
[I don't feel that the service would function as well otherwise][3]. [Anonymity would enable honesty] [1] and a [true space to unburden and discuss deep seated fears] [1].	3, 1, 1
Same reason as above [(If it is not confidential people asking for help will not be honest] [1] and [may need to offload about work situations] [1]. [It will also protect those they work with)] [10]	1, 1, 10
For the same reasons [confidentiality is necessary] [3]. (Work related psychological distress is related to other issues such as bullying and loss of Trust. [Midwives need to feel that they can share their experiences frankly and without fear of reprisal] [6]; this [necessitates confidentiality] [3]. However, [midwives may decide to whistle blow and voluntarily forgo confidentiality.)] [4]	3, 6, 3, 4
This depends what the aims and outcomes are and [how the support will be provided if this is the case][2]	2
Same as prior answer (Has been part of midwifery education that they should be able to cope with all their profession exposes them to hence [can feel shame if not managing)][13]	13
I feel [it is important that information and details will not be able to be leaked or distributed elsewhere] [5] – [concerned about online hacking] [8] – [could discussions be tracked?] [8]	5, 8, 8
as above (As it is online it [must always be 100% confidential][10]. A computer is not a person and able to give feedback or take part in a decision making process)	10
as above (In order to create a safe space)[5]	5
There [should be a choice for participants over the way their identity is expressed and what they are prepared to share] [4]. [Usernames should provide confidentiality][3].	4, 3
[participants should be able to choose what they feel most comfortable with] [4]	4

	**Theme**	**Number of times categorised**
1.	Anonymity – Required for open and honest disclosure	18
2.	Anonymity – May prevent further intervention	3
3.	Anonymity – Needed for support	10
4.	Anonymity - Decided by user	6
5.	Feeling safe/safety - Required	4
6.	Midwives – Fear retribution	6

7.	Midwives – Support is highest priority	3
8.	Anonymity – Not possible online	3
9.	Professional – Legal/Regulatory obligations	4
10.	Anonymity – for third parties	5
11.	Midwives – Need assurances	1
12.	Anonymity is synonymous with confidentiality	2
13.	Midwives – Feel shame if not managing	1

Do you have any additional comments you would like to share?

**Comment**	**Themes assigned**
[Maybe an option to choose to disclose identity could be added] [3] if the [user to would like advice, feedback or referral to psychological therapies] [8.]	3, 5
[A nom de plume could be used to protect users being identified] [1]	1
NO	0
No	0
[People need to feel safe] [12] and so [anonymity provides that sense of safety] [12]; however, [there may be some who hide and take advantage of that anonymity] [2], creating stories that are not true - always possible I guess. [Whether that matters or not, I'm not sure] [13] - perhaps for statistical purposes? [Also distress for the person reading/listening to the story] [2]. I just had situation where a student made up a horrific story of domestic violence for her reflective piece - as it turns out, she fabricated and owned up to the whole thing, but not before [causing a certain amount of distress for those who read the story] [2].	2, 2, 2, 12, 12, 13
see previous response (I believe [some midwives would be fearful of people finding out they were finding it difficult to cope] [9] and [would therefore seek anonymity to feel safe to access support)][10, 4] & [(In a study we have undertaken in Australia confidentiality was rated very highly (not yet published))] [11]	9, 10, 4, 11
[Consider how you would risk assess those individuals who are suffering from psychological disturbance needing support beyond the online package] [5]	5
Thinking about how The Samaritans work. They have a [policy on confidentiality and anonymity which could be emulated] [6]	6
However, this does bring into question [obligations should dangerous practice or illegalities arise] [7] and the [responsibilities of the service owners and those concerned by posts] [7].	7, 7
I am hesitating already as online help is always limited and ultimately the [person will need to get help face to face] [5].	5
[Could a user engage with the resource using a pseudonym?][1] Names, even if not real, [help people to interact with each other] [4].	1, 4
Is there a [risk of anonymity being abused by participants] [2] (e.g. employers seeking information)? [Is there verification?][8]	2, 8

	**Theme**	**Number of times categorised**
1.	Anonymity – Use of pseudonyms	2
2.	Anonymity - could be misused/cause distress	4
3.	Anonymity – Optionality required	1
4.	Anonymity - Required for open disclosure	2
5.	Practicalities – Additional support may be required	2
6.	Anonymity – Requires policy	1

7.	Practicalities – Legal obligations over raising concerns	2
8.	Practicalities – User verification	1
9.	Midwives – Fearful of disclosure	1
10.	Anonymity – Needed to seek support	1
11.	Midwives – Support is a high priority	1
12.	Anonymity – Needed to feel safe	2
13.	Anonymity – Unsure of relevance	1

4

An online intervention designed to support midwives in work-related psychological distress should prioritise amnesty for all platform users in that they will not be referred to any law enforcement agencies, their employer or regulatory body for either disciplinary or investigative proceedings in any case.

4.1 Amnesty for all platform users in that they will not be referred to any law enforcement agencies, their employer or regulatory body for either disciplinary or investigative proceedings in any case

Rank value	Option	Count
1	Not a priority	5
2	Low priority	3
3	Somewhat a priority	3
4	Neutral	15
5	Moderate priority	7
6	High priority	11
7	Essential priority	22

Mean rank	5.08
Variance	3.52
Standard Deviation	1.88
Lower Quartile	4.0
Upper Quartile	7.0

Consensus Achieved = No

Minimum score = Low/Somewhat a priority 3 (4.5%)

Maximum score = Essential Priority 22 (33.3%)

Amnesty for all platform users in that they will not be referred to any law enforcement agencies, their employer or regulatory body

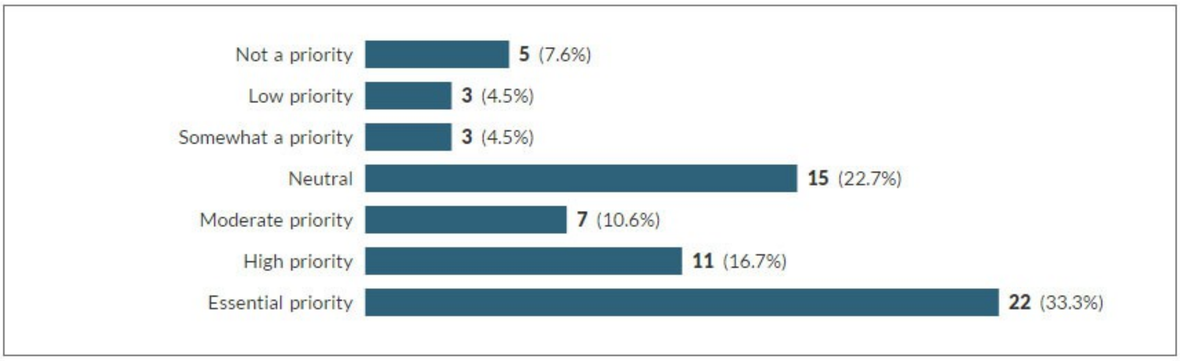

Thematic analysis of open text responses

Why did you choose this rating of priority?

Comment	Themes assigned to
I find this a strange question to have at this stage of a questionnaire. There are protocols for this-[no matter what amnesty is claimed if someone says they've killed someone they will be reported] [3, 4.]	3, 4
Again, without this, [users may well be afraid of the consequences of disclosure] [7] and [not access vital support services] [1].	7, 1
Would you want to do that (referral) in voluntary qualitative research, [sure recipe for failure] [5]?	5
Same answer as the first one. (Many midwifery forums have concerns about posting on line for [fear of retribution by the NMC/public] [7]. Their traffic has reduced in recent years and therefore the [support that they provided has now reduced because of this.)] [10]	7, 10
[This is a non-issue if midwives remain anonymous] [6]. I do feel [conflicted over this issue] [2] - what if the person has committed some heinous crime?	6, 2
If the person knows they are practicing inappropriately then [this should not be perpetuated] [3] or the mechanism could be [argued to be complicit in the abuse] [4]	3, 4
[Information that puts members of the public at risk should be disclosed] [3]	3
[users will not be encouraged to use it if] [1] this clause is not upheld for [fear of reprisals] [7]	1, 7
[Difficult] [2] as [amnesty is an ethical issue particularly relating to criminal matters] [4], however [without it midwives may not feel able to disclose their concerns causing distress] [1, 7]	2, 1, 7, 4
[Difficult] [2] [if someone is clearly in need of help hard to ignore] [8, 4]	2, 8, 4
[This was a difficult one to answer] [2], you hope that many midwives are aware of the responsibility of being open of our own capacity of fitness to practice. Therefore being aware that [some information for our own safety and that of others] may result in some information sharing with those mentioned in the question.	2, 4
Although, [amnesty is important] [1] there is a [duty of care to protect any platform and service user from harm] [4]. Therefore [if illegal acts or safeguarding issues are apparent then I would hope that the researchers would act accordingly and not immorally] [3].	1, 4, 3
[If there is any element of criminality or disciplinary or unsafe practice there cannot and should not be an amnesty] [3]	3
So [people are able to explore the consequences of their actions] [1], free of reprisals [7]	1, 7
Experience with open disclosure evaluation shows [reluctance to disclosure due to fear of reflection being requested in a court of law as evidence] [7]	7
[This is difficult] [2] as the person reading it /responding would have an [ethical obligation to take action if they believed the person was still providing care but unsafe based on what is revealed] [4]	2, 4

Midwives are human just like everyone else and [need somewhere safe to discuss some of the most difficult times of their lives] [11]. [If they think there's a chance they'll get reported to employers or the police, this won't be possible] [1, 5, 7]	1, 5, 7, 11
This makes me wonder about international law? IS the platform going to be available internationally? [If so such an amnesty might not be able to be guaranteed in some countries?] [9]	9
[[There is a requirement for some level of amnesty] [1, 5] [as there is the need to report criminality] [3].] [2, 4]	1,2,3,4,5
[I need to reflect on this more] [2] - if they were describing safeguarding issues for example or could present a risk to women/children then [I don't think there can be an amnesty] [3]. However [some degree of safety for them needs to be assured if they are going to open up and gain any benefit] [1, 7, 5.]	2, 1, 7, 5
[Not sure that any professional could expect such an undertaking] [3]	3
Again [midwives need to feel safe with exploring their own wellbeing without feeling watched or censored] [7, 5,]	7, 5
I think this [goes with the confidentiality and anonymity] [6]. [This is necessary] [5] to [reduce fear] [7] and [build trust and make it a safe place to 'visit'] [1].	1,5,6,7
[safe space] [1, 5] and [reduce fear] [7, 1] of litigation [will encourage openness and reflection] [1] which are key elements	1, 1 ,1 , 7, 5
[I think this is difficult] [2] as it [may impact on the wellbeing of women and families in the midwives' care] [4]	2, 4
Midwives would use their SOM for any issues regarding regulation. I do [feel uncomfortable with this] [2] as I believe that if something is so serious that it is causing psychological distress then [clearly the midwife needs support without fear] [5, 7.] However [if a woman or baby has come to harm then it would need to follow the safeguarding and require investigation in a no-blame culture] [3, 4]	2, 3, 4, 5, 7
[Would like to tick essential priority] [2] but [serious criminal activity cannot be ignored] [4, 8]	2, 4, 8
To allow honesty] [1, 5] [without prejudice] [7]	1,5, 7
[this is difficult] [2] if [issues were needed to be highlighted to protect the public] [8]	2, 8
Because I think [it's a good idea] [1] on some levels. [More good than bad] [1], but [worrying on others] [2].	1, 1, 2
[Ethically and legally] [4] [I do not think you can give these assurances] [3.]	4, 3
Because there is [currently a climate of fear] [7] when mistakes are made/serious incidents happen.	7
[I don't think this can be ensured] [3]. [If there is a situation that someone is declaring they have been involved in a serious case that should be referred then there should be a mechanism to do this] [8]	3, 8
[Is it possible to achieve this?] [9] [Based on safeguarding and Codes of conduct?] [9, 4]	9, 9, 4
[This will encourage midwives to share experiences] [1, 5.]	1, 5
[Again it depends on the aims and objectives of the approach] [2]	2
If there were an [unlawful concern then this must be escalated] [3, 4, 8] [not hidden, although dealt with professionally] [8]	3, 4, 8, 8
[Amnesty would give rise to concerns about safeguarding members of the public] [3, 4.]	3, 4
But [must allow for identification of serious breach of public safety to assure professional accountability] [3, 4, 8]	3, 4, 8
[If there is a requirement to report to someone - midwives would not want to use the resource to debrief if having psychological issues] [1, 5.]	1, 5
[In the case of causing harm or self-harm there must be intervention] [8] - the [platform must be ethical] [4]	8, 4
as above (In order to create a safe space) [1]	1
[unless amnesty is assured confidentiality/anonymity won't be maintained] [1, 5] – [staff are unlikely to use if they think it could lead to disciplinary issues] [5, 7]	1, 5, 5, 7
[Don't think this would be realistic] [9], as [if there was a legal requirement (eg. over safeguarding), it could be enforced by regulator or police] [8], [Better to be honest and state that information will not be shared unless required by law in certain rare	9,8, 8, 3

circumstances] [8, 3.]	
Ideally an online platform should encourage the professional themselves to take action if appropriate & support the NMC Code of Conduct] [4, 12.]	4, 12

	Theme	Number of times referenced in free text
1.	Amnesty – Important/Helpful	19
2.	Amnesty - Conflicted in opinion	13
3.	Amnesty - Cannot be supported	14
4.	Amnesty – Legal and ethical obligations – duty of care	18
5.	Amnesty – Required to facilitate support	13
6.	Amnesty – Automatic if confidentiality/anonymity is afforded	2
7.	Midwives – Fear speaking openly/retribution	15
8.	Practicalities – Intervention may be required	10
9.	Amnesty – may not be possible	4
10.	Midwives – Have little existing provision	1
11.	Midwives – Need support	1
12.	Midwives – Should self-report	1

Do you have any additional comments you would like to share?

**Comment**	**Themes assigned to**
[this is a very difficult point] [10] as [unless they have an opportunity to reflect they may not change practice or report] [4, 6]	10, 4, 6
NO	0
This situation is where the skill of those who are fielding this information comes in – [the support could include the opportunity for that person to reflect on their experiences to the degree that they would take 100% responsibility for their actions] [4] and [talk to management about it] [4, 6] – [in cases of misconduct, then management would have to work out how to manage the situation and the person best] [3].	4, 4, 6, 3
[Disclosure in a closed group or one to one needs protection and support] [5]	5
This [could be tricky to achieve] [10] and [might need a "we won't disclose unless required by law" proviso?] [2]	10, 2
[Users of the platform should be aware that there is the possibility of relevant criminal activity] [11] for example [being reported to law enforcement agencies, but that this would only be done after consultation with the individual] [1]. [It would be preferable if the individual were encouraged to self-report in the first instance] [8].	11, 1, 8
[As part of the professional code of conduct you would expect to be referred] [1]	1
Again [using the principles that The Samaritans use might be appropriate] [9].	9
Unless [someone is at risk of harm or is a danger to themselves or others] [3].	3
[If actions and or omissions had breached NMC code then obligation to be referred?] [10, 1]	10, 1
[This would be important for the midwife sharing her worries and is truly admirable] [4,] but [what position would it put other users in to read about it and then feel that that person's practice was potentially continuing unchecked and could endanger lives?] [7] That [could in turn worry them] [7] as it could be their sister or friend that that midwife then looked after.	4 , 7, 7
[This may put the organisers of the service in a difficult position] [7] and [may not be workable] [12] as there will be [obligations with the Code] [1].	7, 1, 1
On the other hand if [it became obvious that this person needs greater assistance and or treatment and shouldn't be working how this could be managed?] [3]	3
A [declaration can also be a cry for help to someone] [4] "else" ie not in the immediate group or hospital. A step removed	4

	Theme	Number of times

		categorised
1.	Practicalities – There is a duty to report concerns	5
2.	Intervention – Disclaimers may be required	1
3.	Practicalities – Further intervention by management required	2
4.	Amnesty – Required for recovery	5
5.	Midwife – Needs support	1
6.	Midwives – Ideally should self-report concerns	2
7.	Amnesty – May cause distress to others	3
8.	It would be preferable if the individual were encouraged to self-report.	1
9.	Intervention – Consider emulating the principles of comparable interventions.	1
10.	Amnesty – Conflicted in opinion	3
11.	Intervention – Warnings may be required	1

5

An online intervention designed to support midwives in work-related psychological distress should prioritise prompting platform users automatically to remind them of their responsibilities to their professional codes of conduct.

5.1 Prompting platform users automatically to remind them of their responsibilities to their professional codes of conduct.

Rank value	Option	Count
1	Not a priority	7
2	Low priority	9
3	Somewhat a priority	0
4	Neutral	3
5	Moderate priority	13
6	High priority	16
7	Essential priority	18

**Mean rank**	4.91
**Variance**	4.29
**Standard Deviation**	2.07
**Lower Quartile**	4.0
**Upper Quartile**	7.0

Consensus Achieved = No

Minimum score = Somewhat a priority 0 (0%)

Maximum score = Essential Priority 18 (27.3%)

Prompting platform users automatically to remind them of their responsibilities to their professional codes of conduct.

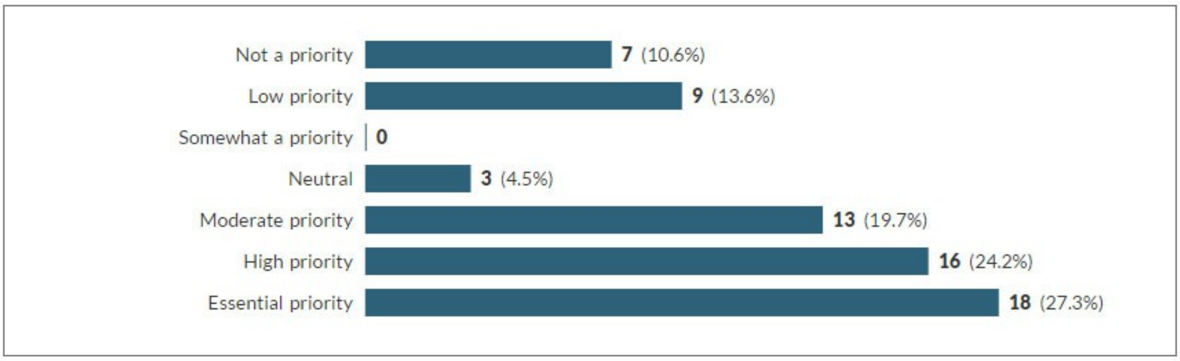

Thematic analysis of open text responses

Why did you choose this rating of priority?

Comment	Themes assigned to
[For goodness sake-if this is linked to trauma why we all of this be considered] [7] – [bit confused by these questions now] [7.]	7, 7
[As long as] [4] this is [done in a gentle and sensitive manner] [3] during a vulnerable time...the [user shouldn't feel threatened in anyway] [3] as the whole purpose of this is to preserve good mental health.	3, 4
Well [it’s the basic benchmark for prof. behaviour no?][6]	6
[One would hope that it wouldn't be necessary] [2] and [sounds like a defensive measure on behalf of the website....] [5]	2, 5
I think [someone in psychological distress won't feel any better being reminded about The Code] [5]. [This may cause more harm][1].	5, 1
it is a [duty to report bad care] [6] or activity which puts [vulnerable people at risk] [6]	6, 6
This give individuals the [opportunity to reflect] [4] on their own actions	4
this would be a [helpful intervention][4]	4
[This is essential][4], where psychological distress is experienced judgement can be seriously altered so if amnesty and confidentiality cannot be guaranteed it will act as a reminder of [midwives professional responsibility to their clients and colleagues][6]	4, 6
we [should always remain professional] [6] no matter what the circumstances	6
For the above reasons for my last answer ([This was a difficult one to answer] [8], you hope that many midwives are aware of the [responsibility of being open of our own capacity of fitness to practice] [6]. Therefore [being aware that some information for our own safety and that of others may result in some information sharing] [4] with those mentioned in the question.)	8, 6, 4
To me, this [seems contradictive] [1, 5] in the context of offering support.	1, 5
As above. This is of [utmost importance] [4, 6.]	4,
[Code of conduct are moral standard] [6] that guide any profession	6
Yes, that is part of the reasoning behind my comment above (So people are able to explore the consequences of their actions, free of reprisals)[4]	4
Our code of practice [cannot be altered][6]	6
this [could be distracting] [5] and seen as [obtrusive] [5] and [judgemental] [5]	5, 5, 5
In my view, there's a chance this [could come across as condescending] [1], when midwives in this state of distress are almost always giving their all (health, family wellbeing, mental wellbeing, etc.) to [fulfil their code of conduct] [6]. The [reminder would have to be written very carefully and supportively] [3] [to avoid hurting] [1] midwives in psychological distress coming to an online 'sanctuary' for support. You [can't underestimate the guilt/shame midwives feel at not meeting the code of	1, 1, 6, 6, 3

conduct] [6].	
[Not sure that I would want] [5] to be reminded that I have to behave within my professional code of conduct if I am feeling distressed.	5
Reminding the user of their professional code of conduct [could play a role in them crystallizing insight] [4] into their situation	4
mws [may be less likely to use a tool][5] like this if it is only going to quote the rules and the code at them.	5
[that is not the purpose of the platform][2]	2
[We all know this anyway][2]. If I were to explore in person wellbeing support services [I would not expect to be told what my responsibility as a midwife are] [2, 5]. And if I did it [may come across as a limit] [5] to what support could be given by this provider of care and support.<br>[I don't feel it's appropriate] [5] for this to become anything connected to regulation of the profession. Supervision and management is part of that system which [may be part of the stress causing factors] [1] for the individual midwife.	2, 2, 5, 5, 5, 1
This is [useful and sensible] [4]. People can get 'carried away' online but they are professionals [need to abide by their professional rules] [6], standards and The Code. I think this would be welcomed as long as it is [a supportive reminder] [3] rather than a threatening tone.	4, 6, 3
this [presupposes midwives are not aware of the code][2] and think you need to [be careful][1] to offer psychological support not be more like line manager or corporate trust	2, 1
People in distress [may lose sight of key issues][4]	4
This is [off putting] [5] when you are already distressed.	5
This [should be second nature] [2]	2
[NMC code should be reiterated] [4, 6] but [profession does know the code] [2] - embedded throughout education and career.	4, 6, 2
When midwives would access this service they would presumably be feeling insecure and that they may not have done all that they could. To have 'This is how you should be' prompted at them would [potentially make them feel much worse] [1] and [leave the website] [4].	1, 4
They [should know this already][2]	2
I [can't see why] [7] this would help them	7
Because an amnesty is hard to achieve and consequently reminding Midwives of their professional responsibilities [should help to clarify] 4, [remind] [4] and [protect] [4] their reflective process. process	4, 4, 4
This [may not be received well] [5]	5
[Could be helpful] [4, 8] depending on the nature of the users issue although [need to be balanced] [3] so not interpreted as a performance measure.	4, 8, 3
We [should abide by our code of conduct at all times] [6]	6
The majority of [midwives do not need reminding] [2] of their responsibilities under the Code. The [tool, will be undermined] [1] if platform users feel chastised.	2, 1
it is a [good idea] [4], but [don't tell them off] [3]	4, 3
Most midwives [already have an awareness of this] [2] but [need to link it to their performance] [4] to ascertain where non achieving	2, 4
Midwives [need to constantly keep in mind their professional codes of conduct] [6]. However there can be great challenges in the workplace in a number of areas when things are going pear-shaped in an emergency situation, with a lack of support from the employer (e.g. not enough staff, turf wars resulting in bad communication or lack of support of other medical colleagues)	6
[Yes this is a priority] [4] but a [job should not come before self] [3]	4, 3
Most [will have an awareness already] [2].	2
[better to encourage staff to come forward and admit errors] [2] so they can receive support (not blame) and help others learn from the mistake etc.	2
Think this [would alter the character of the intervention] [5] and [make it less personal] [5]. Can be a resource for people to use [rather than automatic prompts] [5].	5, 5

Critically we must support staff yet also [protect the public][6] in all of our actions		6
	**Theme**	**Number of times Categorised**
1.	Prompting - May be harmful	8
2.	Midwives – Will already be aware	11
3.	Prompting – Should be done sensitively	6
4.	Prompting – Helpful inclusion	18
5.	Prompting – Unhelpful inclusion	14
6.	Professional codes – adherence a professional responsibility	14
7.	Prompting – Need unclear	3
8.	Conflicted opinion	2

Do you have any additional comments you would like to share?

**Comments**		**Themes assigned**
NO		0
No		0
This requirement would [have to be handled sensitively and carefully] [3] and that's where the skill of the support person/s is so important		3
Maybe if others support this it can be part of the lead in material with a [tick box "I agree"][2] prior to getting into the intervention		2
As a platform that aims to provide psychological support, having the code of conduct 'front and centre' in the process would appear very rules based [not as supportive as if could be] [1.]		1
This could mean that interaction [may be seen as a way of "checking up”] [1] on the profession. Re: Kirkup		1
Maybe provide links or visible ways of accessing these codes [IF they're wanted] 2.		2
Important to [foreground the professional ethical dimension].		4
	**Theme**	**Number of times Categorised**
1.	Prompts – Not supportive	2
2.	Prompts – adherence to code a pre-condition of use	1
3.	Prompts – Sensitivity needed	1
4.	Codes of conduct – important to highlight	1

6

An online intervention designed to support midwives in work-related psychological distress should prioritise prompting platform users automatically to seek help, by signposting them to appropriate support

6.1 Prompting platform users automatically to seek help, by signposting them to appropriate support

Rank value	Option	Count
1	Not a priority	0
2	Low priority	0
3	Somewhat a priority	0
4	Neutral	7
5	Moderate priority	7
6	High priority	21
7	Essential priority	31

Mean rank	6.15
Variance	0.98
Standard Deviation	0.99
Lower Quartile	6.0
Upper Quartile	7.0

Consensus Achieved = Yes (High priority/Essential Priority) 78.8%

Minimum score = Not a priority/Low priority/Somewhat a priority 0 (0%)

Maximum score = Essential Priority 31 (47%)

Prompting platform users automatically to seek help, by signposting them to appropriate support

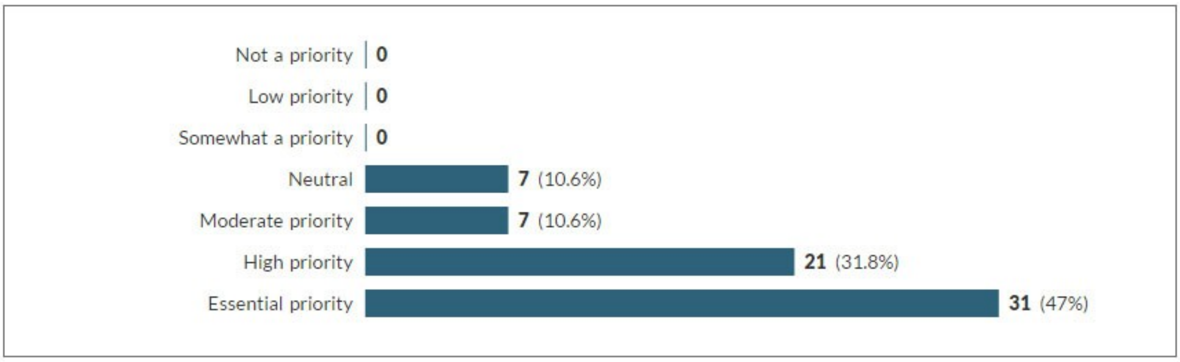

Thematic analysis of open text responses

Why did you choose this rating of priority?

Comments	Theme assigned to
[Because some suicides have been reported] [1] in Australia.	1
I would envision that there [would be a help section, rather than requiring a prompt] [8]. I imagine that [would get annoying] [2] when using a website if prompts kept flashing up!	8, 2
[Vital to refer on if in distress] [1] and requiring support - but [who are you referring in to?][3]	1, 3
[would not be acceptable][6] to give them a platform to vent and then [leave them nowhere to go for help][1]	6, 1
When staff are under extreme stress they 'may not see the wood for the trees' [this could be all they need for self-help][1]	1
Links to support and counselling [would be very beneficial][1]	1
people will often only get help when they are ready so [signposting good idea][1]	1

[Not sure about the 'automatically' part][7]. Certainly, [signposting to appropriate support is vital] [1], but I'm [not sure I understand] [8] how this would be performed in reality.	7, 1, 8
[Extremely important factor][1] to reduce the risk of harm to platform users and others they come in contact with i.e. patients, family, friends, wider society. However, it is up to the platform user to engage in/accept appropriate support so this is an area where [difficulty in uptake may arise] [9].	1, 9
[Nobody is an island of knowledge][1]	1
[it will help midwives][1] obtain the help they need in a most efficient way	1
Yes, that [would be essential][1] as many people don't know where to go or who to trust	1
[Clarity With a Clear process][8] or [pathway to access Support is essential][1]	1, 8
[It would be supportive] [1] and [encouraging][1] and [demonstrate empathy][1]	1, 1, 1
[Yes, important] [1] - but only if the support they are referred to is [high quality and easy to follow] [10, 3]. Depression comes with a lack of ability to take action, and depression is often associated with the kind of burnout midwives experience. The support midwives are offered [must be very high quality] [10] or it could leave them feeling even more defeated.	1, 3, 10, 10
This [would only work if] [4] the online intervention said something like " your answers suggest that you would benefit from face-to-face- support in addition to the online support given here."	4
[It is essential][1] that the user is made aware of the various support modalities available to them	1
Choosing the [appropriate support may be tricky] [3] [though] [1].	1, 3
[Not sure all need to be prompted][2, 7] assumes dysfunction rather than resilience	2, 7
I guess like an online mental health triage service. [Yes I believe this w oh kid be helpful for midwives][1] to get a handle on what options of personal one to one support is available, and [especially good for those][1] personalities that tend to down play significant issues for themselves. [This could be enough] [1] to highlight they do have a significant need for extra support in their life and what that might look like for them.	1, 1, 1
This [could be very useful] [1]. Information about where to go for further help, what can be expected from different agencies and contact details [would be a valuable aspect of the platform][1]	1, 1
[Important] [1] but [depends how this is done and what it is] [3]… could be annoying or could be great][7]	1, 3, 7
[I have difficulty] with the response options here[7]. I think there [may be a step before automatic signposting] [5] and the evidence base is thin for where that should be.	7, 5
[Knowing where and how to seek help is a benefit][1] but [only the platform user knows the correct time to action this][11]	1, 11
[Platform users should have a want to access help] [11]	11
[Should enable them to "work" out the appropriate support for them] [1] [not tell them] [11.]	1, 11
[It's a good idea] [1]	1
[Always to be encouraged] [1] to enable rapid access and support for the individual.	1
Remember, [people may use the tool as an alternative to seeking other forms of support] [1], for whatever reason.	1
[Safety netting important] [6]	6
[Because in extreme distress the person may be unable to self-initiate help][1]	1
This [would be very helpful] [1] as it may not always be easy to access appropriate assistance or know where to obtain it.	1
[Yes this is essential] [1], if someone is accessing an intervention then they are looking for help	1
I think that [using the online intervention is simply a beginning to a helping process] [1] and [should not be seen as the process itself] [5].	1
[staff often don't know what is available to help them][1]	1
[Important][1] to remind participants that there are sources of help	1

Flagging importance of seeking additional support is [critical][1] – [in order to safeguard the individual & the system][6]	1, 6

	Theme	Number of times Categorised
1.	Signposting to support – A useful inclusion	36
2.	Prompts - unsuitable	2
3.	Practicalities – Dependent on the nature of support	4
4.	Practicalities – Needs a personalised tailored response	1
5.	Intervention – if evidence-based	1
6.	Safety is important	3
7.	Conflicted opinion	4
8.	Signposting to support – Clarity on method required	3
9.	Signposting to support – Help seeking may be low	9
10.	Signposting to support – Support must be high quality	2
11.	Midwives – In control of their own help seeking behaviours	3

Do you have any additional comments you would like to share?

Comments	Themes assigned to
Professional support [would benefit some users][1] rather than solely peer support - a link signposting them to sources of support [may enable them to seek help immediately][1]	1, 1
My point is, that one should thread carefully when referring / suggesting people to seek help automatically. This [could lead to pathologization/medicalization of normal reactions to adverse events] [2]. For this reason I am sceptical towards the 'automatically', but it [might be just me reading and understanding it wrongly] [6].	2, 6
No	0
No	0
No	0
This [confuses me a little] [6]. The [online intervention IS support yet you think you need to point them to "appropriate support?"!][3]	3, 6
Accessing the platform [can provide the prompt the user requires][1] to encourage them to seek the ongoing support they may benefit from	1
[Consider prompting via matrix] [4]	4
The [help may just be engaging] [3] with the platform?	3
[Could this be done alongside the main content?] [4] As in in advert format but always present rather than an automatic prompt? So that people could access it when it was right for them rather than feeling beaten into it.	4
[Sign posting very important][1], [use third sector groups and organisations][5]	1, 5

	Theme	Number of times Categorised
1.	Signposting to support – A helpful inclusion	4

2.	Signposting to support - Could lead to users pathologising symptoms	1
3.	Signposting to support – intervention itself is sufficient	2
4.	Prompting - Consider alternative delivery	1
5.	Consider using third sector groups and organisations	1
6.	Automatic signposting - Clarity on method required	2

7

An online intervention designed to support midwives in work-related psychological distress should prioritise the inclusion of web based videos, multimedia resources and tutorials which explore topics around psychological distress

7.1 The inclusion of web based videos, multimedia resources and tutorials which explore topics around psychological distress

Rank value	Option	Count
1	Not a priority	1
2	Low priority	1
3	Somewhat a priority	1
4	Neutral	9
5	Moderate priority	18
6	High priority	27
7	Essential priority	9

Mean rank	5.41
Variance	1.36
Standard Deviation	1.17
Lower Quartile	5.0
Upper Quartile	6.0

Consensus Achieved = Yes (Moderate priority/High priority) 68.2%

Minimum score = Not a priority/Low priority/Somewhat a priority 1 (1.5%)

Maximum score = High Priority 27 (40.9%)

The inclusion of web based videos, multimedia resources and tutorials which explore topics around psychological distress

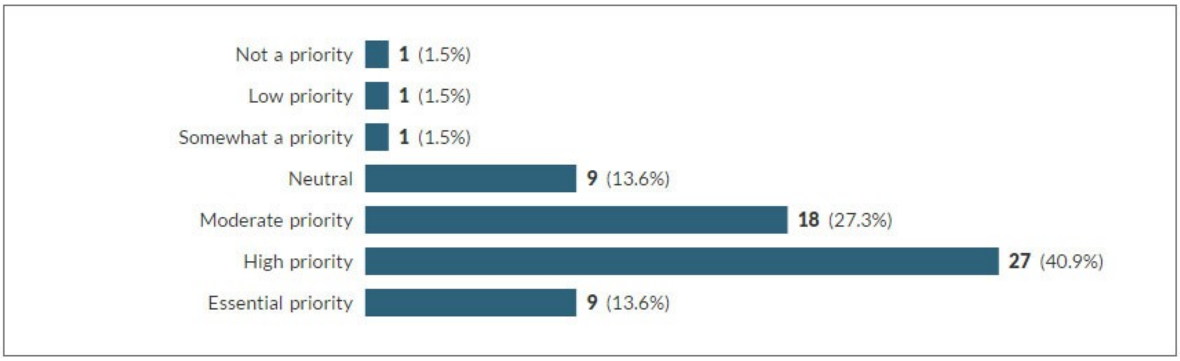

Thematic analysis of open text responses

Why did you choose this rating of priority?

Comments	Themes assigned to
People vary in how they process information-but it's made me ask [what sort of distress are you imagining and why?][6]	6
[This would be beneficial] [1] but would [need to be location specific as not all areas provide the same services] [8].	1,8
I believe it's of only some of [a suite of aids] [3] to be employed.	3
[I am not sure about this][5]. I suppose I'd want to know if there was [evidence to support][4] it as a tool for improving psychological distress	5, 4
[May be a helpful feature][1] [depending on the resources][8] you link to	1, 8
people [need accessible][1] and [varied methods of learning][3] and online gives anonymity	1, 3
[Not a platform that would interest me][2]	2
the inclusion of [different visual mediums will be useful][1, 3] for users who prefer not to share their experiences with others	1, 3
This would be[really useful][1] and make a valuable tool	1
[Great idea] [1] often [people feel failure] by seeking help, so online from own home where accessible from home with peoples busy lives is [great idea] [1.]	1, 1,
I think information should be provided and [presented in a variety of ways] [3], as suggested, to [encourage people to engage] [1] in support services. However, ethical considerations must be given as [some individuals may find the content of such material offensive or distressing] [2].	1, 2, 3
It enhances [more insight into the research][1]	1
[a good way to expose midwives to adequate and helpful information][1]	1
Having online resources for people to explore would be a [very useful and helpful][1] [adjunct to a human being on the end of the line/email/chatroom][3] etc.	1, 3
[Multimedia is important][1] as individuals [need a pick in mix method][3] to cope with stress one size does not fit all	1, 3
it would [depend on the nature of the therapeutic intervention][8] - these resources should only be used in context	8
[Yes,] [1] a [range of resources is a great idea] [3]. Videos work for many people, storytelling is often how we learn hard concepts best, [examples can provide the lightbulb moments][1]	1, 1, 3
if this is an intervention [for those already in distress][2] I [don't think "exploring topics around distress" should be a priority][7]	2, 7
The provision of such 'self-help' [materials can provide an important first step][1] in reducing any psychological distress being experienced	1
[I don't know] [5] if these would be helpful and [id be sceptical] [2] if this was employed if I signed up to something that was supposed to help me if I was in psychological distress.	5, 2
people relate to information in different ways so [a range of resources and tools are	3

required] [3]	
This [would provide a good visual and audio information sharing tool] [3] for those that are visual and auditory learners. It can get overwhelming reading heaps of psychology info. Also when an individual feeling stressed and having issues with depression reading and focusing is not always doable. Or only doable in variable degrees	3
Supporting people to find ways of helping themselves and showing them how to do this with examples would be another [important aspect of a support platform] [1]. The [emphasis should be on sharing, caring and support and resilience] [3] rather than just a place to moan (although there will obviously be a need for that too).	1, 3
1. [I am not aware that this is evidence based] [4]<br>2. This [may increase the likelihood of a midwife not receiving support] [2] as he/she may not be able to access these in their work time/ location thus encroaching on personal time	4, 2
This is a [great idea] [1] as it would signpost and midwives to real support and resources at a time when they may feel overwhelmed	1
[Increase awareness of condition] [1] and/or [to aid in understanding of it] [1].	1, 1
This is [very important] [1] as the need to explore issues first would help and this is accessible any time day or night.	1
This is a [fantastic idea] [1]	1
If someone is in such a state of distress [will they be wanting to look at videos, or read something] [5] [rather than talk to someone?] [7]	5, 7
I imagine this [could be a very useful] [1] means of support to some midwives.	1
[Helpful as an immediate intervention] [1]. Although [not everyone's preferred learning style] [3] and [need to be high quality] [4.]	1, 3, 4
[As long as] [1] they [don't detract from the individual] [5.]	1, 5
This is a [useful] [1] way for [some people] [3] to share, reflect etc.	1, 3
I see [greater need for one on one counselling support] [7] rather than an educative process that further [implies failure] [9]	7, 9
This [would be good] [1] for the time before a crisis situation or in the time of healing after an event once some work had been done to resolve issues.	1
[Yes] [1], this may open their mind that they are not alone, [be able to put into place ways of keeping themselves safe] [1], [give them strategies to help themselves] [1]	1, 1, 1
inclusion [will help] [1] support/educate staff	1
Evidence from Health talk Online and elsewhere that appropriate video material [can help participants understand their feelings] [1] and [realise that they are not alone] [1].	1, 1
Inclusion of [resources that participants can chose to access is key] [3]	3

	**Theme**	**Number of times Categorised**
1.	Multimedia tutorials – Helpful inclusion	32
2.	Multimedia resources – Unhelpful inclusion	5
3.	Multimedia – Variety in content presentation useful	13
4.	Multimedia resources – Evidence based/high quality resources required	3
5.	Multimedia resources – Conflicted opinion	4
6.	Multimedia resources – Benefit dependent upon the nature of distress	1
7.	Midwives – Greater need for alternative support	3
8.	Multimedia resources – Benefit dependent upon the nature of resource	3
9.	Midwives – Feel like failures	1

Do you have any additional comments you would like to share?

Comments	Themes assigned to
[It enhances the resource] [1] and [gives more choice to users] [1]	1, 1
This [would work for some] [1], [not for others] [5]. [Could not stand alone] [2].	1, 5, 2
NO	0
No	0
sometimes you [fill more comfortable to seek help not from people but from multimedia resources] [1, 2]	1, 2
Short videos and downloadable podcasts explaining aspects [would be readily accessed] [1] - people are seeking resources on line more and more these days and to have specially targeted, evidence informed resources [would be very beneficial] [1]	1, 1
This question makes me [wonder if there needs to be two parts to this] [2] a "prevention and information " section and a "support"	2
A balance has to be found between the material provided and [ensuring that those with more severe psychological distress do not rely upon the available information as their crutch] [3]	3
[Content would determine how you expect these to be used] [4]	4
It [can be difficult to find the information required] [1] as well as what words to search with	1
[Using multi media will add variety and depth] [2]	2
[Have them available] [1] so they can be accessed if needed, [don't force them on the midwives!] [2]	1, 2

	Theme	Number of times referenced in free text
1.	Multimedia resources – Helpful inclusion	8
2.	Multimedia resources – Require a variety of options	5
3.	Midwives – Material needs to be matched to user needs	1
4.	Usability - depends upon the content	1
5.	Multimedia resources - Unhelpful	1

8

An online intervention designed to support midwives in work-related psychological distress should prioritise the inclusion of informative multimedia designed to assist

midwives to recognise the signs and symptoms of psychological distress

8.1 The inclusion of informative multimedia designed to assist midwives to recognise the signs and symptoms of psychological distress

Rank value	Option	Count
1	Not a priority	1
2	Low priority	1
3	Somewhat a priority	0
4	Neutral	5
5	Moderate priority	12
6	High priority	26
7	Essential priority	21

**Mean rank**	5.85
**Variance**	1.4
**Standard Deviation**	1.18
**Lower Quartile**	5.0
**Upper Quartile**	7.0

Consensus Achieved = Yes (High priority/Essential priority) 71.3%

Minimum score = Somewhat a priority 0 (0%)

Maximum score = High Priority 26 (39.4%)

The inclusion of informative multimedia designed to assist midwives to recognise the signs and symptoms of psychological distress

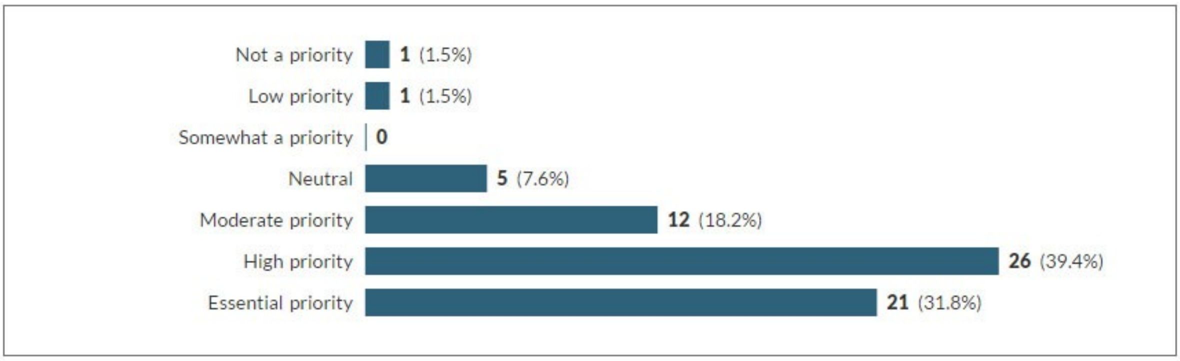

Thematic analysis of open text responses

Why did you choose this rating of priority?

**Comments**	**Themes assigned to**
[No idea whether this would be effective or not] [4] – [what outcome is wanted, what problems are anticipated?] [4]	4, 4
I would assume they [midwife would have already recognised signs and symptoms] [7] if they have chosen to use the online tool.	7
[Anything helpful in raising awareness is valuable] [1].	1
[Signs and symptoms are really important] [1] as [we often don't see them in ourselves] [8] until we are way passed a certain point. [Info on burn out would be particularly helpful] [1.]	1, 1, 8
As above ([May be a helpful feature] [1] depending on the resources you link to) [4]	1, 4

[varied and accessible methods of learning] [3]	3
[This may help] [1, 4]	1, 4
this [could be beneficial to midwives] [1] who are [unaware that they are struggling] [8]	1, 8
even as a trained healthcare professional [distress can affect judgement particularly of your own wellbeing] [8]	8
often [we don't realise how stressed or traumatised we are] [8] and we keep carrying on so [highlighting these symptoms great idea] [1]	1, 8
Once again; I worry that ["counting symptoms" could lead to pathologization of a normal condition.] [9]	9
Extremely important as [individuals may not realise that they are in psychological distress] [8] and [may enable self-help activities] [1]/ [inform where to seek further help and support] [1].	1, 1, 8
[This aid learning] [1]	1
a [good way to expose midwives to adequate and helpful information] [1]	1
Having a one stop shop, including signs and symptoms would [make it so easy for people to access] [1] and [get validation for how they feel] [1] - a [vital feedback tool] [1.]	1, 1, 1
Sometimes working under stress is becoming the normal practice. We [need to highlight the signs and symptoms] [1, 8] to midwives to create clear awareness	1, 8
Again [depends on the nature of the intervention] [4] [if synchronous no but maybe if it's purely an asynchronous resource] [4]	4, 4
A [huge part] [1] of addressing psychological distress is [making sure midwives can identify when they are at risk] [8.]	1, 8
see response at 7 (if this is an intervention for those already in distress I [don't think "exploring topics around distress" should be a priority)] [2]	2
As what would most likely be a first point of contact [it would be advantageous] [1] for the user to be able to understand the apparent degree of stress they are experiencing with information/suggestions on an appropriate course of action	1
I can see how [this would work] [1] as I think [many mws don't recognise the sign and symptoms of stress, PTSD, depression or anxiety] [8.]	1, 8
I believe [this is what most platform uses would be wanting] [1] to have more awareness around. I know when I was having a work triggered emotional breakdown and especially when I wanted to understand what had happened as I was recovering something specific to being a midwife that would clearly identify what this distress was or had been [would have been great for me] [1]. Instead I checked out many different websites and at times found this overwhelming as too much info to process at a time of vulnerability	1, 1
I think [this is useful] [1] up to a point but I think [midwives will already have recognised this if they are visiting the platform] [7]. I would be slightly [cautious about encouraging midwives to pathologies their distress] [9.] But I can see that some common sense guidance on recognising depression and suicidal thoughts (and what to do about this) [would be essential] [1].	1, 7, 9, 1
See previous comment re the evidence-base for these modalities (I am not aware that this is evidence based) [5]	5
[Difficult at the time] [1] to [acknowledge signs/symptoms] [10]	1, 8
[Training helps to see it in others] [1] but [very difficult to see in oneself] [8] #johari window.	1, 8
This [would be really useful] [1] both for the [individual] [1] and as [a means of supporting their colleagues] [1].	1, 1, 1
I think self-assessment of distress [would be helpful] [1] but [not on its own] [3] -needs solutions and was forward too	1, 3
If this is going to be a resource for support in the future [this would be helpful] [1]	1
This [could offer clarity and context] [1] for a distressed midwife seeking support.	1
[Helpful to raise awareness] [1] and [support the individual] [1] and [others] [1.]	1, 1, 1
[May help midwives] [1] recognise when they are suffering from psychological distress.	1

[supporting peers is key] [10]<br>[asking for help is also important] [11]	10, 11
There is a big [difficulty in self-identifying you are at risk] [8] and as it is not spoken about isolation occurs	8
A tool [would be useful] [1] as it is [not always easy to see the symptoms in ourselves] [8] or [acknowledge that we need help] [11]	1, 8, 11
[Could be useful] [1], depends though – [could end up self-diagnosing!] [9]	1, 9
[will help] [1] to educate and support staff	1
Done effectively, this [could help people] [1] [recognise and accept that they are experiencing distress] [8]	1, 8
in order [to engage professionals it will need to provide an unique offer] [4, 6]	4, 6

	Theme	Number of times referenced in free text
1.	Informative Multimedia – Helpful inclusion	37
2.	Informative Multimedia – Unhelpful inclusion	1
3.	Multimedia – Need a variety of resources	2
4.	Conflicted – Depends upon objectives/content	7
5.	Multimedia - Resources must be high quality/evidence based	1
6.	Multimedia - Needs to be unique	1
7.	Multimedia - Not required	2
8.	Midwives – Do not always recognise own distress	13
9.	Informative Multimedia - could lead to inappropriate self-diagnosis	3
10.	Midwives – Support is important	1
11.	Midwives – Help Seeking is important	2

Do you have any additional comments you would like to share?

Comments	Themes assigned to
it [will be a way of assessing] [1] whether they are at risk of developing psychological distress	1
Is it always due to individual problems if a person experience psychological distress?... Maybe it is a [completely healthy and normal response to some structural problems] [3] that should be solved at an organizational level, not an individual level?	3
NO	0
No	0
the [information should be clear and simple] [4] (not complex)	4
A lot of hazing and bullying that goes on tends to undo people's sense of self and self of understanding what's happening; [good to have a checkpoint] [1]	1
see response at 7 (if this is an intervention for those already in distress I [don't think "exploring topics around distress" should be a priority)] [2] & (This question makes me wonder if there [needs to be two parts to this a "prevention and information " section and a "support")] [6]	2, 6

[It is only now that I can look back and realise what I was experiencing was a sign/ symptom of PTSD] [7]		7
In themselves and in others? if so [they can offer peer support] [1]		1
A [midwife may have a critical incident occur and go through the routine process and think it has been dealt with only to discover that it is affecting her life and work] [7] in subtle ways. Or there can be a vicarious complaint that comes much later which [can set the whole thing off again] [2.]		7, 2
[People asking for help are likely already to be further along the journey of recognition and acceptance] [2]. [Could this be used for initial engagement with the site?] [5]		2, 5
	**Theme**	**Number of times Categorised**
1.	Informative multimedia - helpful inclusion	3
2.	Informative Multimedia – Unhelpful inclusion	3
3.	Organisational – Distress is a normal response to organisational issues	1
4.	Informative Multimedia – resource should be clear and simple.	1
5.	Informative multimedia – To be used in initial engagement	1
6.	Informative media – Requires a variety of options	1
7.	Midwives – Do not always recognise own distress	1

9

An online intervention designed to support midwives in work-related psychological distress should prioritise the inclusion of multimedia resources which disseminate self-care techniques

9.1 The inclusion of multimedia resources which disseminate self-care techniques

Rank value	Option	Count
1	Not a priority	1
2	Low priority	0
3	Somewhat a priority	1
4	Neutral	4
5	Moderate priority	11
6	High priority	29
7	Essential priority	20

Mean rank	5.89
Variance	1.22
Standard Deviation	1.1
Lower Quartile	5.25
Upper Quartile	7.0

Consensus Achieved = Yes (High priority/Essential priority) 74.2%

Minimum score = Low priority 0 (0%)

Maximum score = High Priority 29 (43.9%)

The inclusion of multimedia resources which disseminate self-care techniques

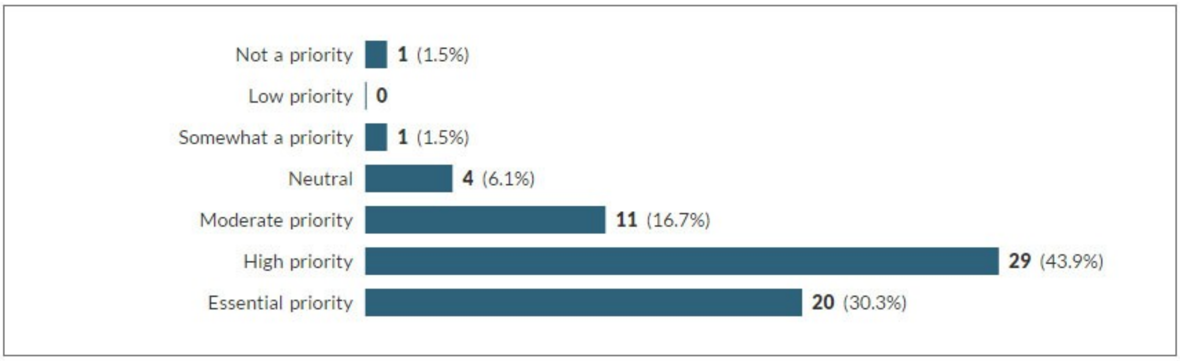

Thematic analysis of open text responses

Why did you choose this rating of priority?

**Comments**	**Themes assigned to**
Completely [neutral about whether or not this can help] [8]	8
This [would be very useful] [1.]	1
As above [(Anything helpful in raising awareness is valuable.)] [1]	1
I think it [needs to go on beyond the normal trite] [4]: have a bath, make sure you can some 'you time', spend time with the family stuff....maybe [some actual stories][4] of how midwives have overcome particular situations with self-care techniques	4, 4
Now this [would be a helpful feature] [1] to enable midwives to begin to build emotional toolkit. However, I worry that it may [miss people in need of more professional input] [6]	1, 6
[best way to get information out] [1]	1
As above [this mode of support would not interest me] [2]	2
As there is a real shortage of support and understanding for midwives who maybe experiencing stress - learning self-help techniques are [extremely important] [1]	1,
again people are busy and face to face not for everyone so [giving choices is essential] [3]	3
As above. ([Extremely important] [1] as individuals [may not realise that they are in psychological distress] [11] and [may enable self-help activities/inform where to seek further help and support] [6]) However, this relies on the individual to engage fully with such material in order to reap the benefits.	1, 6, 10
[nice to have] [1], but should be very [easy and safe to use] [7]	1, 7
too many people [don't take care of themselves] [5]; a readily accessible list of self-care modalities can[give people ideas of what steps to take] [1]	1, 5
Self-care is so important and [often ignored or always last on an individuals to do list] [5]	5
would be a [good support] [1] – [self-directed strategy] [1]	1, 1
If you can get a resource that spreads and helps a lot of midwives (viral content) [this would be amazing] [1] - it's hard to design viral content for lots of reasons, and impossible to guarantee a piece of content will disseminate, but aiming for this will often mean content is engaging and will communicate a message very well. This isn't an academic resource, but could be a very helpful book Sally: http://www.amazon.com/Contagious-Things-Catch-Jonah-Berger/dp/1451686579/ref=asap_bc?ie=UTF8	1
see response at 7 (if this is an intervention for those already in distress [I don't think "exploring topics around distress" should be a priority)] [2]	2
[It is essential] [1] that midwives have to hand information that can immediately facilitate them taking steps to alleviate some of the distress they are experiencing	1
Again, [not sure if this would make any difference] [8.] [But suppose it wouldn't do any	8

harm]...[8]	
[useful first step] [1] for some people so [good to include it] [1]	1, 1
In most services this is all that is available [promoting individual resilience is the key to sustainability] [3]	3
[Goes without saying] [1]	1
[I see this as key] [1] and one of the big problems in midwifery. Midwives [tend to be ignorant about how and why they need to care for themselves] [5, 10]. It seems to be a cultural thing. Many midwives treat tea/meal breaks as an [optional extra] [5]. I think that tells us something about how they view self-care. Yes [help with self-care techniques is really important] [1] and [has the potential to turn things around for individual midwives] [1.]	1, 10, 5, 1, 1
[if diagnosing symptoms needs to provide help and not just by referral] [6]	6
Only [relevant if aware and prepared to help one's self] [14]	5
[important to give strategies and self-care] [1] - however [people are unique] [3] so how do we account for that	1, 3
I think that this [would be really helpful.] [1]	1
[Useful]. [1]	1
[Good to include] [1] provided [balanced with further support] [6] and [not just provided as only solution] [3.]	1, 6, 3
Someone using this tool [may well be looking for self-help] [1] as they do not wish to engage with other professionals about their issues.	1
Self-care [very important as a midwife] [1, 9]	1, 9
This [has to be with the inclusion of personal support] [6, 3] as resources not able to recognise persons current ability to up lift or put strategies in place	6, 3
[Helpful] [1] but at the time of crisis [links to outside resources to assist or facilitate maybe more supportive] [6, 3.]	1, 6, 3
[Yes] [1,] if they are accessing this resource they are looking for help and support. Again don't force it on them!	1
[need to encourage staff to look after themselves more] [9] – [always put patients first sometimes at the expense of themselves] [5]	5, 9
[Practical means of self-help] [1, 4,] [possibly supported by app] [7.]	1, 4, 7
[this will allow professionals to take immediate action] [1]	1

	**Theme**	**Number of times Categorised**
1.	Multimedia self-help resources – Helpful inclusion	28
2.	Multimedia self-help resources – Unhelpful inclusion	2
3.	Need a variety of resources	6
4.	Multimedia self-help resources – Needs to be useful	3
5.	Midwives - do not prioritise self-care	5
6.	Midwives – additional support may be needed	6
7.	Multimedia self-help resources - ease of use important	2
8.	Neutral	2
9.	Midwives – Need support and understanding	2
10.	Midwives – Do not always recognise own distress	2

Do you have any additional comments you would like to share?

**Comments**	**Themes assigned to**
NO	0
I don't think it is enough to provide the ideas; many people [need to be 'accountable'] [5] to someone to say they've done it or not; what about [access to a coach or mentor] [6] so that the person has their interest at heart, but not emotionally attached to check in with and [help the person stay on track?] [4]	4, 5, 6

see response at 7 (This question makes me wonder if there [needs to be two parts to this a "prevention and information " section and a "support")] [3]		3
Making such information available [also has a preventative component] [1]		1
I thought I felt fine, I [thought self-help was to continue at work and work through it] [7]		7
[Self-care for one person can be very different to another?] [7]<br>[Simplicity is the word I think of in self-care] [8] - one thing not a big list as that seems overwhelming like a set of tasks to achieve and then [you may measure yourself as failing if you do not achieve them ?] [2]		7, 8, 2
[highlight importance of self-awareness] [1]		1
Would have to [have a range of strategies] [3] to cover difference personalities and different issues		3
[Peer support likely to be a significant] [9] feature of the intervention, how it is envisaged that this might develop? Under what circumstances?		9
	**Theme**	**Number of times Categorised**
1.	Multimedia self-help resources – Helpful inclusion	2
2.	Multimedia self-help resources – unhelpful inclusion	1
3.	Resource – Must be multiple options available	2
4.	Midwives – Need assessment	1
5.	Midwives – Must be accountable	1
6.	Midwives – Provision of coaching	1
7.	Midwives - Meaning of self-care unclear	1
8.	Resources - Should be simple	1
9.	Resources - Peer support is useful	1

10

An online intervention designed to support midwives in work-related psychological distress should prioritise the inclusion of multimedia resources which disseminate relaxation techniques

10.1 The inclusion of multimedia resources which disseminate relaxation techniques

Rank value	Option	Count
1	Not a priority	1
2	Low priority	1
3	Somewhat a priority	1
4	Neutral	8
5	Moderate priority	23
6	High priority	20
7	Essential priority	12

Mean rank	5.41
Variance	1.42
Standard Deviation	1.19
Lower Quartile	5.0
Upper Quartile	6.0

Consensus Achieved = Yes (Moderate priority/High priority) 65.1%

Minimum score = Not a priority/Low priority/Somewhat a priority 1 (1.5%)

Maximum score = Moderate Priority 23 (34.8%)

The inclusion of multimedia resources which disseminate relaxation techniques

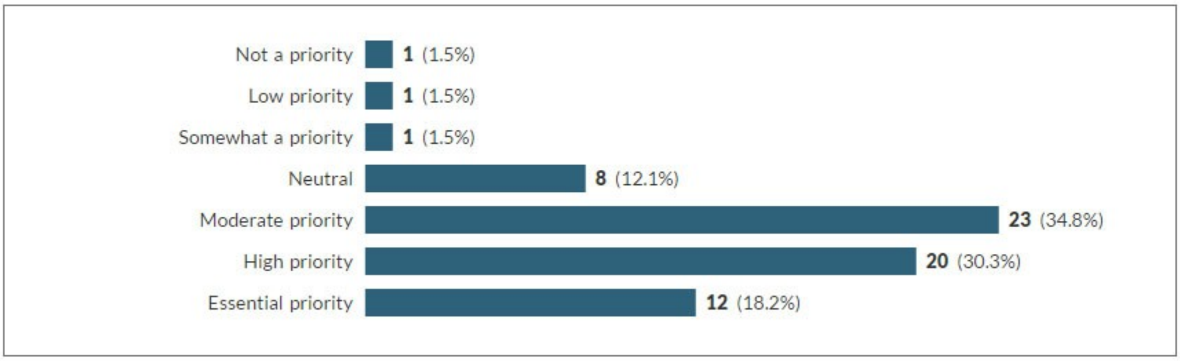

Thematic analysis of open text responses

Why did you choose this rating of priority?

**Comment**	**Themes assigned to**
All these options imply that the problem is located in individuals-maybe the distress is a [reasonable response to a dysfunctional environment] [6]	6
One imagines [Midwives should be au fait with this one more than most?] [5]	5
Headspace app has proven useful to some people I know. [This feature could do the same thing.] [7, 1]	7, 1
[best way to reach majority] [1]	1
as above (this mode of support would not interest me) [2]	2
As last question (As there is a real [shortage of support and understanding for midwives] [9] who maybe experiencing stress - learning [self-help techniques are extremely important)] [1]	1, 9
[might help some] [1], but I have found my own personal ways to relax	1
[This is a good idea to relieve stress] [1] however [time constraints/family commitment/outside pressures may have a negative impact on uptake and engagement] [10]	1, 10
Any intervention [should not further compound the stress] 9	9
[need to be very simple and safe to use] [5, 8]	5, 8
Most of us haven't been taught how to relax, so [having these resources would be so beneficial] [1]	1
[Depends on the technique] 11	11
[good if contextualised within a program] [1]- lots of relaxation resources available already- therefore [needs to be in the context of a bigger picture guided intervention] [13]	1, 2
As in last comment. [(If you can get a resource that spreads and helps a lot of midwives (viral content) this would be amazing)] [13] Also, relaxation is often quite a personal thing, [hard to recommend effective techniques for all I think?] [3] Perhaps midwives need reminding that they're doing an incredible job. Events and demands they come across are so stressful and life changing that most of the population would need counselling to cope. Midwives often [feel guilty] [12] for catching up on sleep, having time out watching TV, gently exercising with friends etc. – it's not that they don't know how to relax, more that they're under tremendous time pressure and [feel guilty for taking time out] [12]. Just my thoughts.	3, 12, 13, 12
see 7 (if this is an intervention for those already in distress I [don't think "exploring topics around distress" should be a priority)] [2]	2

[Relaxation techniques go hand in hand with self-care techniques] [4]	4
[Needs individuals to buy into finding time to undertake techniques] [5, 10]	5 , 10
[Part of the self-help] [4.] Also [good for all midwives to know well] [1,] and be able to personally and help their clients use.<br>I know this is one of the most basic but helpful areas of self-development that [is given me much more resilience] [1]. Especially in relation to work related inter professional conflict and horizontal violence issues. If I'm more relaxed I manage to not own other people's bullshit.	4, 1, 1
[Yes] [1] this would be useful for some. How many [midwives advocate breathing and relaxation for women in labour but never try this out themselves as a relaxation technique?] [15] The majority I would guess. [Several different techniques should be offered] [3] to suit different personal preferences.	1, 3
[Strength of evidence][1]	1
[Different methods must be explored] [3] as not one size fits all.	3
I think these are fairly well publicised already but it [wouldn't do any harm] [1].	1
[Helpful] 1 but [may not suit everyone] [3]	1, 3
[this kind of stuff can be accessed elsewhere] [2]	2
[Possibly useful] [1]	1
Again [good resource to include] [1] as an immediate support balanced with others and as prioritised in line with evidence base.	1
A ["nice to have"] [1] but other factors more important	1
[not everyone finds 'relaxation' easy] [2]	2
[May not necessarily be appropriate to an individual] [2,]	2
Again, [would be helpful] [1] but there [an outside resource may be more helpful] [14] during the crisis - having it only online [may add to the isolation and depression a midwife may be feeling] [2]	1, 14, 2
[Some may find this useful] [1] – [don't think this is a priority] [2]	1, 2

	**Theme**	**Number of times Categorised**
1.	Relaxation techniques - A helpful inclusion	18
2.	Relaxation techniques - An unhelpful inclusion	8
3.	Resources – Need a variety of options	4
4.	Relaxation is a self-care technique	2
5.	Resources - Must be easy to use	2
6.	Organisational - distress can have organisational cause	1
7.	Resources – Could emulate comparable alternatives	1
8.	Resources must be safe to use	1
9.	Midwives – shortage of support and understanding	1
10.	Outside pressures – May inhibit use	2
11.	Relaxation techniques– benefit dependent on technique used	1
12.	Midwives – often feel guilty	2
13.	Need to generate viral content	1
14.	Midwives – May need additional support	1

Do you have any additional comments you would like to share?

**Comments**	**Theme assigned to**
Are they not able to [apply their unique knowledge to themselves] [4]	4
(Yet again; be careful about this approach. [Is the message that you have to learn to relax more/better in order to handle with your work?)] [2]	2
NO	0
[People need to understand the toll that stress takes] [1] and [why deliberate relaxation practice is important for their ongoing health and wellbeing] [1]; the resources [could explain	1, 1, 3

the Polyvagal theory] [3] and how the ANS works so that they understand scientifically, the benefits of relaxation practices	
[Needs to be very simple short] [5] and with [reference to further prolonged techniques] [7]	5, 7
see 7 (This question makes me wonder if there [needs to be two parts to this a "prevention and information " section and a "support")] [7]	7
[Limited evidence of relaxation] [6] over resilience techniques in effectively reducing distress	6
I had counselling and it [was one of the first techniques discussed and practised] [1]	1
A [variety of relaxation techniques] [7] – [visualisation][7] - or [suggestions of techniques they may try - yoga class etc] [7]	7, 7, 7
[Mindfulness could be useful too] [8]	8

	**Theme**	**Number of times Categorised**
1.	Relaxation techniques - A helpful inclusion	3
2.	Relaxation techniques – May convey the wrong message	1
3.	Resources - could/should explain theory behind relaxation	1
4.	Midwives – Can apply their own knowledge	1
5.	Resources - Need to simple and comprehensive	1
6.	Relaxation - Limited evidence base	1
7.	Relaxation techniques – Requires a range of options	4
8.	Techniques - Consider mindfulness	1

11

An online intervention designed to support midwives in work-related psychological distress should prioritise the inclusion of mindfulness tutorials and multimedia resources

11.1 The inclusion of mindfulness tutorials and multimedia resources

Rank value	Option	Count
1	Not a priority	1
2	Low priority	0
3	Somewhat a priority	1
4	Neutral	12
5	Moderate priority	17
6	High priority	27
7	Essential priority	8

**Mean rank**	5.38
**Variance**	1.24
**Standard Deviation**	1.11
**Lower Quartile**	5.0
**Upper Quartile**	6.0

Consensus Achieved = Yes (Moderate priority/High priority) 66.7%

Minimum score = Low priority 0 (0%)

Maximum score = High Priority 27 (40.9%)

The inclusion of mindfulness tutorials and multimedia resources

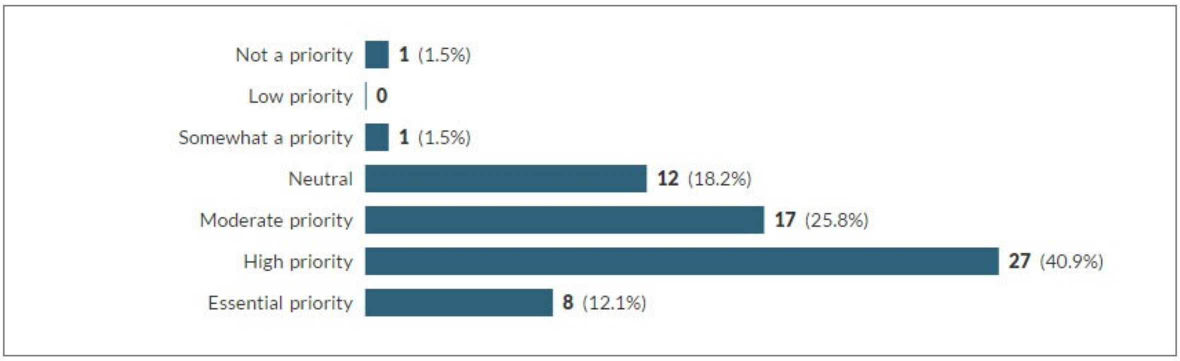

Thematic analysis of open text responses

Why did you choose this rating of priority?

Comment	Themes associated with
All the things I've said already [(Neutral)] [4]	4
Essentially same as above (10) [(Midwives should be au fait with this one more than most?)][7]	7
I have found mindfulness [particularly helpful] [1.]	1
it is topical and [helps many] [1] but [some may not respond to it][2]	1, 2
Many individuals are [not aware of what is happening as it is happening] [14.] They are concentrating on other things, completing electronic records but [not seeing what is happening in the room] [14].	14, 14
a [useful link] [1] that many would be interested to learn more about	1
[not sure what is meant by mindfulness] [6]	6
My reasons for choosing the last 3 questions as high priority is [due to stigma] [8] midwives [may not be ready to seek face to face help and support] [9] however [seek help using the highlighted resources] [1]	8, 9, 1
As above. (This is a [good idea to relieve stress] [1,] however [time constraints/family commitment/outside pressures may have a negative impact on uptake and engagement.)][13]	1, 13
It [reinforces learning] [1]	1
[need to be very simple and safe to use][10]	10
See previous answer; [relaxation and mindfulness practices are twins (in my view)][15] – [essential tools] [1] to manage stress and support our psychophysiological wellbeing	1, 15
Mindfulness is the new buzz word and [midwives may glaze over this as a fad][11]	11
[good emerging evidence re mindfulness as a strategy] [1]	1
Could be [very helpful] [1] in getting midwives to take action on mental wellbeing.	1
mindfulness is [certainly on way of helping] [1] those in distress	1
The inclusion of psychological techniques [may be beneficial to some] [1], [dependent on the degree of distress being experienced] [16]	1, 16
This [could be really useful.] [1]	1
[Excellent skills to foster] [1]	1
[So much benefit to Mindfulness] [1], and an increased general population awareness of its lifelong benefits. I wish all kids were taught this from a young age.<br>Before I go into the hospital I do a short Mindfulness and relaxation technique. This [helps me let go of past hurts] [1] created by maternity power Struggles and previous horizontal violence. It [helps me present to the now] [1], and [see today's issues with today's eyes] [1]. Not reactive eyes from days gone by.	1, 1, 1, 1
Again it [will not appeal to all] [3] but it certainly [has a place] [1].	3, 1
[More focussed than just relaxation and?] [3] [more evidence base of efficacy] [5]	3, 5
as above (Strength of evidence) [5]	5

[Not sure what this is] [6]	6
[Can be a bit narrow] [12]. [Problem specific whereas if your diagnosis crosses across a couple of areas] [12]	12, 12
This is the [most relevant to me] [1] as I believe [this is the way forward when I feel psych distress] [1.]	1, 1
[I don't know very much about mindfulness.][6]	6
I think the [evidence on the benefits of mindfulness are getting stronger] [1]	1
mindfulness geared to practitioners [would be helpful][1] and it has been shown to increase resilience	1
As above this [may be helpful for some][1]	1
[Need to be balanced with evidence base] [5] on interventions and impact on targeted intervention.	5
[I have yet to evaluate mindfulness and its effectiveness].[4]	4
Again [many people are not familiar or sceptical re mindfulness][11]	11
As above [(May not necessarily be appropriate to an individual)] [2, 3]	2, 3
This should be a part of all midwifery training and [useful] [1] but [not necessarily at the time of a crisis] [2.]	1, 2
There is so much on the internet but some [links maybe helpful][1]	1
[should be included][1] because different techniques help different people	1

	**Theme**	**Number of times Categorised**
1.	Mindfulness - A helpful inclusion	26
2.	Mindfulness - An unhelpful inclusion	2
3.	Resource – Need a variety of options available	3
4.	Mindfulness – Neutral opinion	2
5.	Mindfulness – Degree of evidence	2
6.	Mindfulness – Meaning unclear	3
7.	Midwives - should know this technique already	1
8.	Midwives – Face stigma	1
9.	Midwives - May not want face to face support	1
10.	Resources - need to be simple and safe to use	1
11.	Mindfulness - Midwives may be sceptical	2
12.	Mindfulness – Conflicted opinion	2
13.	Midwives – other pressures may inhibit use	1
14.	Midwives – Do not always recognise own distress	2
15.	relaxation - synonymous with mindfulness	1
16.	Effectiveness - dependent on the degree of distress	1

Do you have any additional comments you would like to share?

**Comment**	**Themes assigned to**
[A supportive prof. friend would be better] [1] than most of these modalities or complement them!	1
As above, no. 11 ((Yet again; [be careful about this approach. Is the message that you have to learn to relax more/better in order to handle with your work?))] [2]	2
NO	0
No	0
[Offer A pick in mix of options to avoid one term] [3]:<br>Breathing techniques<br>Soothing music<br>Mindfulness<br>Etc.	3

[It is important to be able to identify those users who are more severely distressed] [4] and [encourage them to seek professional help rather than have them self-manage] [5]		4, 5
I suffered from PTSD and severe reaction to stress [difficult to access both areas of relevance] [3, 6]		3, 6
[links and signposts to mindfulness websites etc.][3]		3
	**Theme**	**Number of times Categorised**
1.	Mindfulness - A supportive professional friend would be better	1
2.	Resources - May send unwanted messages	1
3.	Resources – Must offer a variety of options	3
4.	Midwives – Must be risk assessed	1
5.	Midwives – Must be encouraged to seek professional help	1
6.	Resources – Must be accessible	1

12

An online intervention designed to support midwives in work-related psychological distress should prioritise the inclusion of Cognitive behavioural Therapy (CBT) tutorials and multimedia resources.

12.1 The inclusion of Cognitive behavioural Therapy (CBT) tutorials and multimedia resources

Rank value	Option	Count
1	Not a priority	3
2	Low priority	1
3	Somewhat a priority	0
4	Neutral	13
5	Moderate priority	22
6	High priority	18
7	Essential priority	9

**Mean rank**	5.12
**Variance**	1.86
**Standard Deviation**	1.37
**Lower Quartile**	4.25
**Upper Quartile**	6.0

Consensus Achieved = Yes (Moderate priority/High priority) 60.6%

Minimum score = Somewhat a priority 0 (0%)

Maximum score = Moderate Priority 22 (33.3%)

The inclusion of Cognitive behavioural Therapy (CBT) tutorials and multimedia resources

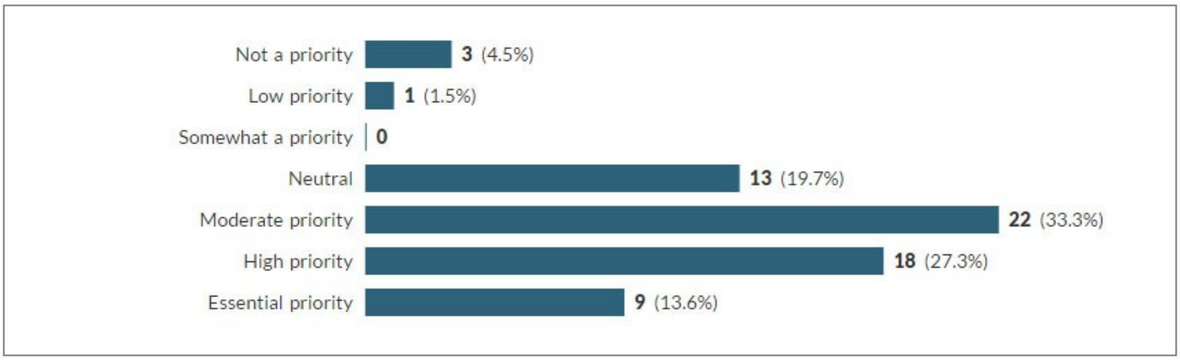

Thematic analysis of open text responses

Why did you choose this rating of priority?

Comment	Themes assigned to
There is recent psychological [evidence that the effectiveness of this technique is declining] [5]	5
The [self-help would be very useful] [1], especially to those so [don't feel able to access outside services] [9] or [where there is no provision of services] [9]	1, 9, 9
Same reasons as above. [(A supportive prof. friend would be better than most of these modalities or complement them!)] [3] Most [info will only be shared in a "safe" environment.] [10]	3, 10
[this is the most appropriate technique for dealing with anxiety] [1] and [can be empowering][1]	1, 1
As above, not a medium I would use.[2]	2
Again like mindfulness [some midwives would wish to learn more about this intervention][1]	1
this therapy is [helpful for some][1] but again a [wide range of choices will help][3] individualise the support required	1, 3
For again the reasons stated above ([due to stigma][11] [midwives may not be ready to seek face to face help and support] [10] however [seek help using the highlighted resources)][1]	11, 10, 1
As above (question 10) (This is a [good idea to relieve stress] [1], however [time constraints/family commitment/outside pressures may have a negative impact on uptake and engagement.)] [12]However, I am unsure if individuals would [need outside support] [4] with this to ensure effectiveness.	1, 12, 4
To [assess the level of reasoning] [1]	1
[need to be very simple and safe to use][7]	7
These tools have been [found to be beneficial][1] through research, so they [would be beneficial with this work too][1]	1, 1
See above (Mindfulness is the new buzz word and [midwives may glaze over this as a fad)][13]	13
need to undertake primary research to [compare which technique to use][2]- [putting too much in may weaken the effect and cause confusion]8	8, 2
[Could be great] [1], and the presence of CBT itself [could act as a great reminder] [1] that what they do is very challenging and worthy of this kind of support.	1, 1
[CBT is useful][1] for those in distress	1
The inclusion of psychological techniques [may be beneficial to some][1], [dependent on the degree of distress][14] being experienced	1, 14
[How would mws know which therapy route to follow?][8]	8
Personally feel it [could be more useful than some of the other resources] [1].	1
[Needs to be supported][4] for professional to utilise fully	4

I wonder if this is [more appropriate to do with a psychologist personally] [4], as safer for the midwife. By putting it on the platform to give tutorials of how to do it [could result in a vulnerable midwife self-managing more serious issues] [2] when [professional support would be better for her] [4].<br>I guess the psychologists would be better served to answer this.<br>I think a brief overview on what CBT or EMDR is and the research around its benefits [would be beneficial] [1]…	4, 2, 4, 1
This is [not an area I know much about][6]	6
again [better than relaxation alone][1]	1
Only concern is that if CBT is required then the individual [should undergone proper and robust assessment and support] [4]	4
Have [been advised to use online CBT training] [1]	1
Think this is the realm of therapy and [needs to be "real"] [4.]	4
[CBT needs expert input][4], raising awareness of CBT would be good however	4
As above (Need to be [balanced with evidence base] [14] on interventions and [impact on targeted intervention] [15].)	14, 15
Possibly helpful, but is it even possible to [design a "one size fits all" CBT algorithm?][17]	17
[Not sure of what CBT is] [6]	6
As above (May not necessarily be appropriate to an individual)[2]	2
Again [another useful tool] [1] to know about but [should be a part of training or ongoing continuing professional development] [4] before a critical incidence where it could be a useful tool to deal with what's happening.	1, 4
If they are requiring therapy they really [need some professional help] [4]	4
Think therapy is [best provided face to face] [4]	4

	**Theme**	**Number of times Categorised**
1.	CBT tutorials - A helpful inclusion	18
2.	CBT tutorials – An unhelpful inclusion	3
3.	Resources - Need a variety of options to suit all	2
4.	Intervention - Users may need additional support	9
5.	CBT tutorials - reduced evidence base	1
6.	CBT tutorials – Unclear meaning	2
7.	CBT tutorials - Needs to be easy and safe to use	1
8.	Resources - too many interventions may weaken the effect	2
9.	Midwives – May not access other CBT support	2
10.	Midwives – Need safety to disclose	2
11.	Midwives – face stigma	1
12.	Midwives – other pressures may inhibit use	1
13.	Midwives – May not be convinced of positive effect	1
14.	Effectiveness – Dependent on evidence and context	2
15.	Midwives – may need a targeted intervention	1

Do you have any additional comments you would like to share?

**Comment**	**Themes assigned to**
it’s about [offering as many options as possible] [1] as not all users will want the same kind of support	1
Having sought CBT myself you have to commit to set sessions and this is [not always possible with shift patterns] [2] and midwives may [not be happy with employers knowing they are having treatment due to stigma] 3.	2, 3
NO	0
I'm [not familiar with CBT tutorials for self-use] [10], they [need to be professional and simple to use] [4.]	10, 4
I'm a [fan of Dialectical Behavioural Therapy (DBT)] [5] and it [would be good to incorporate into this set of tools too] [5]	5, 5
[Has online CBT been shown to be effective?] [6]	6
It is [important to be able to identify those users who are more severely distressed] [7] and [encourage them to seek professional help] [8] rather than have them self-manage	7, 8
I had some EMDR following worked related PTSD and this [worked very well] [11] at helping re fame it in my mind and stop the flash backs and night mares I was having 3 years after the traumatic birth of an IUD term induction and v near death of my independent clients. While being a NZ 24-7 on call independent midwife.<br>I [needed to be able to see research evidence] [9] that it was a useful tool before I would let myself have it. [Knowing that it was recommended on the NICE PTSD guideline really helped me trust it was worth trying] [9]. It [worked fab] [11] thank goodness, and I no longer hold these images and distress in my everyday life.	11, 9, 9, 11

	**Theme**	**Number of times Categorised**
1.	Resources - Need to offer as many options as possible	1
2.	Midwives – May be impractical	1
3.	Midwives - Face stigma	1
4.	CBT tutorials - need to be professional and simple to use	1
5.	Resources - Consider Dialectical Behavioural Therapy (DBT)	2
6.	(CBT) tutorials - Question evidence base	1
7.	Midwives – Need risk assessment	1
8.	Midwives – Need encouragement to seek help	1
9.	Therapies - Evidence base instils confidence	2
10.	CBT – Unfamiliar with the therapy	1
11.	EMDR – Works well	2

An online intervention designed to support midwives in work-related psychological distress should prioritise the inclusion of information designed to inform midwives where they can access alternative help and support

13.1 The inclusion of information designed to inform midwives where they can access alternative help and support

Rank value	Option	Count
1	Not a priority	0
2	Low priority	0
3	Somewhat a priority	0
4	Neutral	1
5	Moderate priority	8
6	High priority	26
7	Essential priority	31

**Mean rank**	6.32
**Variance**	0.55
**Standard Deviation**	0.74
**Lower Quartile**	6.0
**Upper Quartile**	7.0

Consensus Achieved = Yes (High priority/Essential priority) 86.4%

Minimum score = Not a priority/Low priority/Somewhat a priority 0 (0%)

Maximum score = Essential Priority 31 (47%)

The inclusion of information designed to inform midwives where they can access alternative help and support

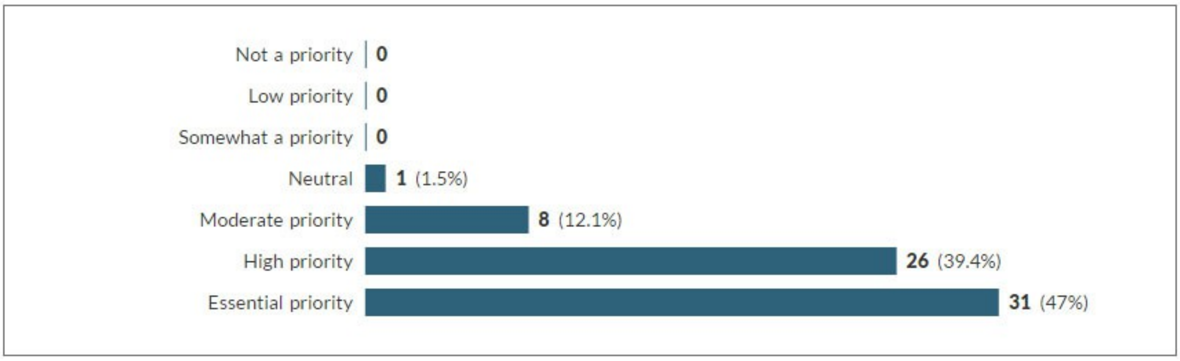

Thematic analysis of open text responses

Why did you choose this rating of priority?

Comment	Themes assigned to
I suspect that in difficult circumstances the unfortunate and costly [reality that we need to talk to other people] [4] will be the main need	4
[Yes] [1], [if they don't have a close colleague who is an unbiased listener] [4].	1, 4
it's [a 'must have'][1]	1
[Giving choice] [2]	2
as previous question (like mindfulness [some midwives would wish to learn more about this intervention)] [1]	1

again [choice is imperative] 2	2
[Don't know what is meant by alternative help and support] [5].	5
[Very important] [1] as [some individuals naturally will need more support than others] [2].	1, 2
[A lot of us appreciate and use] [1] alternative or complementary therapies (assuming that's what you mean here).	1
[Need to be real and local][7] and [shown to be effective] [3]	7, 3
[should be directive to other sources of support][1] but [not so much that they cannot see the wood for the trees] [8], [concentration may be an issue if they are very stressed] [9]	1, 8, 9
[Seems like a very sensible idea] [1]. [Sometimes you need the humanity of taking in person to someone] [4].	1, 4
I believe [many midwives would prefer this option 1st.] [1]<br>However is they flag up serious symptoms. Then [recommended professional service][2] should also be recommended	1, 2
[Supported by evidence] [3]	3
[Essential in aiding your recovery] [1]. If it is signposted for you rather than having to trawl through the web	1
[Very important] [1] to know where to go and access.	1
This [acknowledges that it's OK to ask for help and that it is out there] [1].	1
[Very important] [1] to continue support.	1
[Could be the prompt a midwife is looking for] [1].	1
They [can find the support that suits them] [1]	1
The reality is however [there are few resources available] [6]	6
During a period of psychological distress a [midwife may isolate her/himself due to depression] [9], may leave the profession – [personal professional assistance may be required] [2] to deal with whatever is happening in their life as a consequence. [A link to local alternative help and support would be essential] [1].	1, 9, 2
Again if they are accessing this resource [they are looking for help and support][1]	1
The relational dimension is [very important to offer psychological support.] [1]	1
Online support [will not suit all staff] [2] –[some may respond better to alternative methods of support] [2] so online [should be part of what is on offer][1]	2, 2, 1

	**Theme**	**Number of times Categorised**
1.	Signposted to help and support – A helpful inclusion	18
2.	Help and support – Need a variety of options available	6
3.	Help and support – Must be evidence based	2
4.	Help and support – Face to face support preferable	3
5.	Alternative help and support – Unclear meaning	1
6.	Help and support - few resources actually available	1
7.	Therapies – Must be real and local	1
8.	Therapies – Too many = Confusion	1
9.	Midwives – Impaired functioning when distressed	2

Do you have any additional comments you would like to share?

**Comment**	**Themes associated with**
The ["other" supports are still needed] [1] as a "Failsafe"	1
NO	0
[I really like EFT (Emotional Freedom Technique)] [2] as a way of defusing troublesome emotions and the emotions associated with distressing experiences. [I find it very effective] [2], both for myself and when working with others	2, 2
Perhaps [set up a local peer group][3] having gone through an unexpected event at work to go to cinema a coffee morning to [informally debrief] [3] and listen to each other	3, 3

it is important for midwives to know what they can access within their own trust [Could there be links to employee support in Trusts - Occupational Health depts?][4]		4
	**Theme**	**Number of times Categorised**
1.	Help and support – Need a variety of options available	1
2.	Therapies - EFT (Emotional Freedom Technique) can be useful	2
3.	Therapies – Suggest peer group debriefing	2
4.	Therapies – Suggest links to local occupational Health resources	1

14

An online intervention designed to support midwives in work-related psychological distress should prioritise the inclusion of information designed to inform midwives as to where they can access legal help and advice.

14.1 The inclusion of information designed to inform midwives as to where they can access legal help and advice.

Rank value	Option	Count
1	Not a priority	1
2	Low priority	1
3	Somewhat a priority	1
4	Neutral	8
5	Moderate priority	16
6	High priority	15
7	Essential priority	24

**Mean rank**	5.7
**Variance**	1.76
**Standard Deviation**	1.33
**Lower Quartile**	5.0
**Upper Quartile**	7.0

Consensus Achieved = No

Minimum score = Not a priority/Low priority/Somewhat a priority 1 (1.5%)

Maximum score = Essential Priority 24 (36.4%)

The inclusion of information designed to inform midwives as to where they can access legal help and advice.

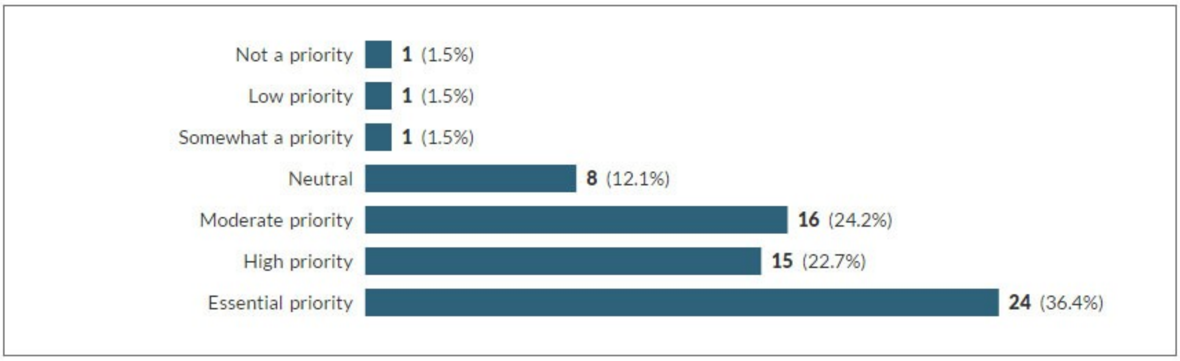

Thematic analysis of open text responses

Why did you choose this rating of priority?

Comment	Themes assigned to
If the environment is that problematic [an advocate is always helpful] [1]	1
[Essential] [1] as we live in a litigious and unforgiving world.	1
[Many are unaware of the options] [1] and stress situations make accessing help difficult at times.	1
this is an [essential part of the intervention] [1] - a [link to where unbiased confidential advice can be sought] [1]	1, 1
[Not sure about this one] [3] I am [not keen on encouraging legal advice] [2], [unions would give this is required] [4].	2, 3, 4
As above. ([Very important] [1] as some individuals naturally will need more support than others.)	1
it can [help midwives to feel safe][1]	1
[Yes][1,] Legal issues are very important and [should be part of the tool kit] [1]	1, 1
[Many midwives don't know the difference between the NMC and RCM or INMO] [1]	1
[Maybe] [3]<br>but again in context- the [mere suggestion that they might want to access legal support may add further stress to midwives] [2] who hadn’t previously considered that option	3, 2
If offering holistic support to midwives, [this is crucial] [1,] and obstetrics/midwifery comes with the highest level of litigation in NHS.	1
again- [not sure that is its prime purpose] [7]- they [can access legal advice through work] [4]	7, 4
[Not sure if this would be appropriate or not] [3]	3
[?evidence base] [5]	5
Most if not all [would belong to RCM or other union] [4]	4
think this is [an issue that causes much psych distress][1]	1
This isn't something midwives will automatically have prior knowledge of so [will be quite useful] [1].	1
Depends whether this is a target of the work. [Focus should be on support and prevention in first instance] [7]. [Individual may not be in a position to use this to help and support them at the time of use] [2].	7, 2
Midwives who are fearful in a litigious society [may need the reassurance of legal advice] [1].	1
They [need to know there rights] [1] i.e. employment law, the employers duty of care etc	1
As above (The reality is however there are [few resources available)][6]	6
[Each legal jurisdiction in different countries will be different] [8]. If this is to be generic an accessible by midwives from all over the world, then there would be may different	8, 1

avenues for obtaining legal access and advice. [Every working midwife should know what is available] [1] locally for her through her midwifery association, union or insurance. So there would be a lot of work setting up the local links for every country where this information/support is available.	
Yes [this is essential][1] for some problems	1
[Can find this out from RCM] [4]	4
Needs careful thought - legal route [might add to stress and have negative outcomes] [2]. How could this be contextualised?	2

	Theme	Number of times referenced in free text
1.	Legal help and advice - A helpful inclusion	17
2.	Legal help and advice - An unhelpful inclusion	4
3.	Legal help and advice – conflicted opinion	3
4.	Legal help and advice - Unnecessary	4
5.	Legal help and advice – Question evidence base for this	1
6.	Legal help and advice - Few resources available	1
7.	Legal help and advice - Not a priority	1
8.	Legal Help and advice – Varies globally	1

Do you have any additional comments you would like to share?

Comment	Theme assigned to
A lot of consumers [don't know any other way to express their grief and anger] [1].	1
complaints, investigations and litigation are [some of midwives biggest fears] [1]	1
NO	0
Whether the person has been bullied, is a bully or has been involved in an adverse outcome, a legal consultation [can be very helpful and even essential] [1]	1
Midwives [need a listening ear after an event] [1]<br>They also [need assistance how to writing statements for legal system] [1]	1, 1
[Start with Unions - RCM, Unison] [1]	1
[Professional body support too][1], [a direct line to someone trained to deal with these issues confidentially and without judgement (one their side)] [2]	1, 2

	Theme	Number of times Categorised
1.	Legal help and advice - A helpful inclusion	7
2.	Legal help and advice - Consider providing personal legal advice	1

15

An online intervention designed to support midwives in work-related psychological distress should prioritise giving platform users the ability to share extended personal experiences for other platform users to read

15.1 Giving platform users the ability to share extended personal experiences for other platform users to read

Rank value	Option	Count
1	Not a priority	1
2	Low priority	3
3	Somewhat a priority	6
4	Neutral	13
5	Moderate priority	17
6	High priority	13
7	Essential priority	13

Mean rank	5.02
Variance	2.2
Standard Deviation	1.48
Lower Quartile	4.0
Upper Quartile	6.0

Consensus Achieved = No

Minimum score = Not a priority 1 (1.5%)

Maximum score = Moderate Priority 17 (25.8%)

Giving platform users the ability to share extended personal experiences for other platform users to read

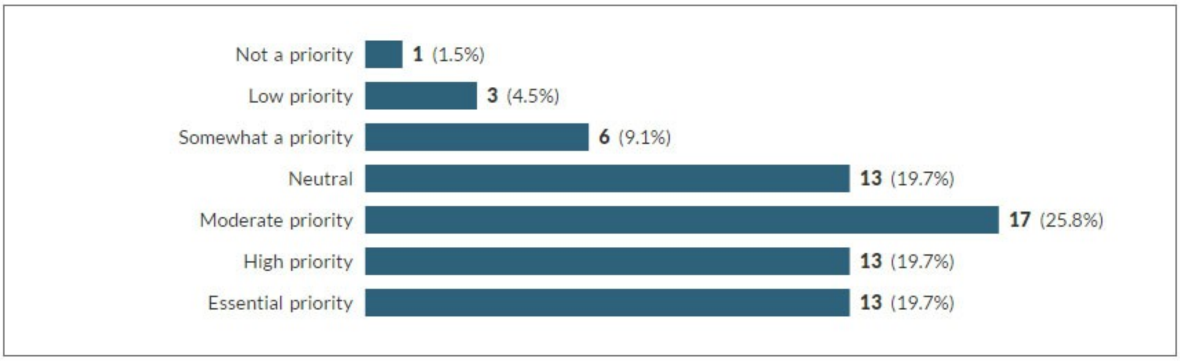

Thematic analysis of open text responses

Why did you choose this rating of priority?

Comment	Themes assigned to
This is a complicated thing to produce and [likely to be fake unless it is real stuff written by real people] [7] and the context is a little tense to be relying on this.	7
[This could ventilate some stress] [1]. You [might not feel so isolated] [1].	1, 1
Peer debriefing and peer support has [got to be one of the most useful aspects of improving psychological work based stress] [1]. Adding to stress/distress is often a sense of isolation it brings therefore [peer support is essential] [1] in my opinion	1, 1
[Might be helpful] [1] to facilitate reflection but [could be counterproductive] [2] if responses are negative – [would require careful moderation] [5]	1, 2, 5
then [people will realise they are not alone] [1]...but only useful if tis on resolution and positive outcome also given	1
This is an [option that could help many] [1], often peer support is [all that is needed] [1].	1, 1

discussing with others is [cathartic and a way of feeling understood] [1]<br>A confidential forum [allows discussion to take place without feeling judged] [1]	1
[Peer support is a valuable tool] [1] but I have seen situations where issues have been shared online become increasingly tense as people are [concerned about confidentiality] [6] and worried about sharing too much information	1, 6
as long as this is anonymous yes [good to hear others experiences] [1]	1
This [may help others] [1] to realise that they are not alone and may [encourage them to have self-belief] [1] to [engage with support systems] [1] and [realise that things will get better over time] [1.]	1, 1, 1, 1
[Collaborative effort is Paramount] [1]	1
writing and sharing is a [very powerful][1] and [helpful tool][1] in treating psychological distress	1, 1
It is [important] [1] to be able to share stories to [help with others supporting] [1] and to [help the person involved feel heard] [1] and [not alone] [1] and also that often just verbalising issues albeit online is a [huge step] [1] to help process it and [prevent people internalising it and making it worse] [1].	1, 1, 1, 1, 1, 1
Other people's stories [can be very helpful] [1]. I [wouldn't make it mandatory for people to share their stories though] [3]; it would be [good to have a repository] [1] of stories for those who would like to read and share their own	1, 3
Blogging needs to be brief and [can be difficult when legalities are underway][4]	4
this has the [risk of inadvertent sharing] [7] – [breach of confidentiality][6] – [information being accessible to legal teams etc.][6]	7, 6, 6
[We learn from stories] [1], and [identifying with others is very powerful] [1]. This is the reason why healthcare blogs are so popular, if this can be harnessed, it [might turn the online intervention into something extremely popular for midwives] [1].	1, 1, 1
[stories are a good vehicle for some] [1]	1
[May help to share experiences] [1].	1
[Not sure this would be helpful] [4] [unless moderated?][5]	4, 5
This [could help] [1] or [hinder] [2] someone else's journey through stress. A bit like woman's birth stories. [Positive ones can be uplifting] [1], traumatic ones [can trigger more fear in the reader] [2].<br>I think with caution and platform [manager to be able to vet the content of] [5], and also to [ensure all parties remain unidentifiable] [5]. Especially around woman's confidentiality.	5, 5, 2, 1, 2, 1
Some midwives [will find this useful] [1] as a reflection and therapeutic. They [will need to be mindful of their codes of conduct when they do this].[9]	1, 9
creating a community of users [would be important] [1] to allow staff not to feel alone and share experiences	1
Experience "lived" has [so much to give to the "reader”] [1]. It [helps them to feel they are "not alone"] [1] and [could aid their psych distress] [1.]	1, 1, 1
[This is so important] [1]. [It's good to read that it isn't just you] [1] and [it's good to share] [1.]	1, 1, 1
This needs care, and [would need to be carefully anonymised][10]	10
Because those who are really asking for help [may not want to read others' lengthy stories that may trigger things for them][2]	2
[Possibly] [4], but it [would need to be monitored] [5.]	4, 5
[Depends if this will be helpful and have an effective impact on users] [4.]	4
[this may conflict with confidentiality] [6]	6
[May be beneficial to read how others have sought assistance and improved their long term health] [1]	1
[May help a midwife feel less alone] [1].	1
[Not sure] [4]<br>[Need a moderator] [5]	4, 5
This [would counter the isolation] [1] most midwives feel with this stress	1
[Not sure how this can be achieved while maintaining confidentiality and anonymity] [6]	6

[Yes][1], [would have to be moderated] [5]	1, 5
This is [tricky ethically] [8]	8
[Not sure of the value of just sharing experiences without the opportunity for other to feedback?] [4]	4
Think this is going to be a [critical element of the intervention] [1]. [First step in reaching inner acceptance] [1].	1, 1
[the value of peer support] [1] - requires focus & encouraging this via the online platform [will be helpful] [1]	1, 1

	Theme	Number of times Categorised
1.	extended personal experiences = A helpful inclusion	48
2.	extended personal experiences – An unhelpful inclusion	4
3.	extended personal experiences - Must be optional	1
4.	extended personal experiences – conflicted opinion	6
5.	extended personal experiences – Requires moderation	7
6.	extended personal experiences = Must protect confidentiality	5
7.	extended personal experiences – Could be misused	2
8.	extended personal experiences - ethically problematic	1
9.	Midwives – if conducted within professional codes	1
10.	extended personal experiences - Requires anonymity	1

Do you have any additional comments you would like to share?

Comment	Themes assigned to
there is [little time to discuss feelings and experiences at work] [1]	1
[As long as they could do so anonymously if required] [1, 2]	1, 2
NO	0
No	0
[Be mindful of identifying people and situations][2, 3]	2, 3
This would obviously need to be done very carefully taking into consideration that then [both anonymity and confidentiality could be breached] [2, 3]	2, 3
Everyone's experiences are different in the midwifery setting so [I don't feel it would be useful] [5]. I haven't found anywhere the same or a close situation to mine.	5
[Should be encouraged to reflect] 1 in a [professional manner] 4	1, 4
[Love midwifery story sharing] [1]	1

	Theme	Number of times Categorised
1.	extended personal experiences = A helpful inclusion	4
2.	extended personal experiences = Must protect anonymity	3
3.	extended personal experiences = Must protect confidentiality	2
4.	extended personal experiences = Must remain professional	1
5.	extended personal experiences = An unhelpful inclusion	1

An online intervention designed to support midwives in work-related psychological distress should prioritise the inclusion of a web based peer to peer discussion chat room

16.1 The inclusion of a web based peer to peer discussion chat room

Rank value	Option	Count
1	Not a priority	3
2	Low priority	7
3	Somewhat a priority	2
4	Neutral	11
5	Moderate priority	16
6	High priority	20
7	Essential priority	7

Mean rank	4.79
Variance	2.65
Standard Deviation	1.63
Lower Quartile	4.0
Upper Quartile	6.0

Consensus Achieved = No

Minimum score = Somewhat a priority 2 (3%)

Maximum score = High Priority 20 (30.3%)

The inclusion of a web based peer to peer discussion chat room

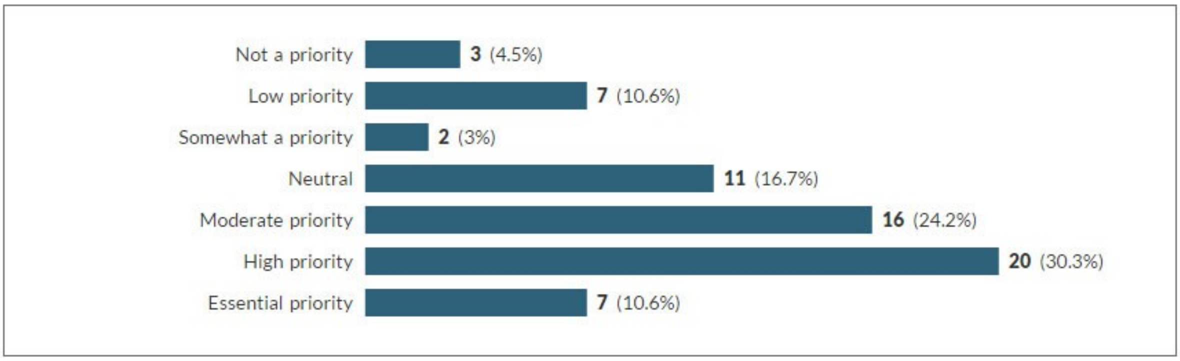

Thematic analysis of open text responses

Why did you choose this rating of priority?

Comment	Themes assigned to
[Do they have time for such things?][6]	6
[Could work for some folk][1]	1
As above (Peer debriefing and peer support has [got to be one of the most useful aspects of improving psychological work based stress] [1]. Adding to stress/distress is often a sense of isolation it brings therefore [peer support is essential in my opinion] [1])	1

As above – [I worry this may be counterproductive] [2]. Also, [would require high volume site traffic to be viable and sustainable] [7].	2, 7
not sure about this as [could bring difficulties and reporting if infiltrated] [5]	5
as above (This is an option that could help many, [often peer support is all that is needed] [1])	1
As previous question (discussing with others is [cathartic and a way of feeling understood] [1]<br>A confidential forum [allows discussion to take place without feeling judged)] [1]	1, 1
Again issues as above re. [over-cautiousness in regards to confidentiality] [4]	4
[as long as this is monitored][3] [yes][1]	3, 1
[Dependant on whether it was peers seeking support from others in same situation] [9]	9
This is essentially [a good idea] [1], but if someone is feeling particularly down, angry or isolated [it may be a platform for inappropriate discussion/actions] [5]. However, talking through something with someone [may help individuals realise that they are not alone] [1] and that [people do care] [1.]	1, 5, 1, 1
It [facilitate online interactions] [1]	1
[good] [1] for those who use web on regular basis	1
This chat room [could be very beneficial] [1] ; you would [have to have it monitored/facilitated though] [3]	1, 3
Having walked in shoes is [very important] [1] but [peer supporters may need training] [8] also to give appropriate advice	1, 8
synchronous engagement [opportunity essential] [1]	1
Feedback there and then [could be fantastic] [1], and [chat could offer this to members] [1] - however, [how do you guarantee these conversations are constructive?] [5] Online because expressions and body language can't be seen [misunderstandings are common] [2]. There's also a tendency to get [angry that you can see on online forums] [2], so the [ethos of the intervention would have to be carefully made and maintained] [10].	1, 1, 5, 2, 2, 10
I think many might [find this helpful] [1]	1
As Q 15. (May [help to share experiences.)] [1]	1
[Moderation required] [3]	3
Again the same as above. I feel [this would open midwives giving each other well meaning advice] [1] when [seeking specialist psychological services would be safer and or more appropriate] [2.]	1, 2
Some midwives will find this [useful as a reflection] [1] and [therapeutic[] [1.] They will [need to be mindful of their codes of conduct when they do this] [11]	1, 1, 11
In my opinion this group of [midwives need access to professional help and support] [2]	2
[I would not enjoy this][2]	2
Would be [good to communicate] [1] with others offering and receiving support from other midwives going or gone through this type of situation.	1
This could be very [difficult to moderate?] [3]. [The culture of the profession could mean "bullying" tactics could be used in such a forum?] [5].	3, 5
This [would be really useful] [1] if there was [any way of guaranteeing] [3] it would not [fall foul of trolls] [5] when people are already emotionally vulnerable.	1, 3, 5
Not sure about this, [may need more expert skills than a peer can offer] [2]	2
I've [not seen these chat rooms as being particularly helpful][2]	2
I think [many people would want this] [1]	1
This [could be very powerful] [1] and [useful] [1] but may [need to be facilitated] [3] by a neutral voice.	1, 1, 3
[Need a moderator] [3]	3
Think this [could be deconstructive] [2]	2
If this is a worldwide available resource then [midwives need to be able to have that peer to peer discussions available] [1] at a [local level] [12] as regulations and work circumstances can be very different. What applies in one place may not apply in another.	1, 12

Would [have to be moderated] [3]. [Can name names, situations etc] [4] [could be difficult legally] [5]		3, 4, 5
As above (This is [tricky ethically.)][5]		5
[would have to be sure confidentiality/anonymity can be maintained] [3, 4]		3, 4
Again, think this will be a [critical element of the intervention] [1].		1
[Will this be used?] [6] Worth investigating further		6
	**Theme**	**Number of times Categorised**
1.	Peer to peer discussion - A helpful inclusion	28
2.	Peer to peer discussion - An unhelpful inclusion	8
3.	Peer to peer discussion - Needs moderation	9
4.	Peer to peer discussion - Could risk confidentiality/anonymity	3
5.	Peer to peer discussion - Risk of unethical use	6
6.	Peer to peer chatroom - May not be used	2
7.	Peer to peer chatroom - Requires high volume site traffic	1
8.	Peer to peer chatroom - May require trained supporters	1
9.	Effectiveness – Depends upon help seeking behaviour	1
10.	Peer to peer chatroom - Requires rules and standards	1
11.	Professional – Legal/Regulatory obligations	1
12.	Midwives - May need local chat rooms	1

Do you have any additional comments you would like to share?

**Comment**		**Themes associated**
NO		0
As per comment 15. My thoughts on this that it would be best to construct it separately so that others [who choose not to participate] [3] in the online intervention still [have access to peer support] [1] and those who are participating in the online intervention [don't risk their anonymity being breached] [6]		3, 1, 6
People are very [social media dependent] [1]. However it raises [issues of confidentiality and risk] [6] of exposure to non-supportive agencies.		1, 6
Have been in contact with other midwives who have had mental health issues and they [have share support through contact] [1]		1
[need a method to ensure against lurkers] [4]		4
[Limit time for peer to peer chat] [4] and [have moderator/lead] [4, 7].<br>I would worry people suffered more distress if chat not moderated.		4, 4, 7
There are however [circumstances that may be generic which cause psychological distress that could be discussed] [4] - the solutions [locally though may be different] [8.]		4, 8
[Not sure that many midwives would access this] [2]		2
[Needs careful governance] [4] to [prevent abuse/ misuse] [5] and [ensure safeguarding is considered] [4.]		4, 5, 4
	**Theme**	**Number of times**

		Categorised
1.	Peer to peer discussion - A helpful inclusion	3
2.	Peer to peer discussion - An unhelpful inclusion	1
3.	Peer to peer discussion - Should be an optional choice	1
4.	Peer to peer discussion - Requires moderation	6
5.	Peer to peer discussion - Risk of misuse	1
6.	Peer to peer discussion - May risk anonymity/confidentiality	2
7.	Peer to peer discussion - Requires guidance	1
8.	Peer to peer discussion - May be local variations	1

17

An online intervention designed to support midwives in work-related psychological distress should prioritise giving platform users the ability to communicate any work or home based subjects of distress

17.1 Giving platform users the ability to communicate any work or home based subjects of distress

Rank value	Option	Count
1	Not a priority	2
2	Low priority	6
3	Somewhat a priority	1
4	Neutral	10
5	Moderate priority	16
6	High priority	16
7	Essential priority	15

Mean rank	5.12
Variance	2.65
Standard Deviation	1.63
Lower Quartile	4.0
Upper Quartile	6.0

Consensus Achieved = No

Minimum score = Somewhat a priority 1 (1.5%)

Maximum score = Moderate Priority/High Priority 16 (24.2%)

Giving platform users the ability to communicate any work or home based subjects of distress

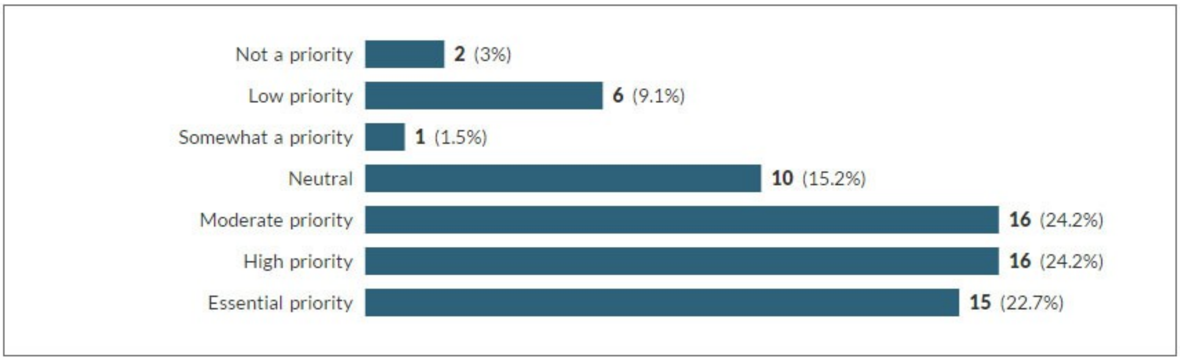

Thematic analysis of open text responses

Why did you choose this rating of priority?

Comment	Themes assigned to
[I don't think this is controllable] [5] if genuine help is offered	5
As above. [(Could work for some folk)][1]	1
Home stress and work are often [heavily influenced by one another][2]	2
[relevant to the whole person][1] but [other mechanisms for this] [4]	1, 4
[Home stress can impact on work and the other way] [2].	2
this is [essential] [1] as issues at [home impact greatly on performance in the workplace][2]	1, 2
I think [work based] [3] would keep things more professional maybe with links to home based subjects. [Personally I like to keep the two separate][4]	3, 4
[Important] 1 in one way to discuss individuals concerns/worries/stresses, however the [issue surrounding anonymity of workplace and colleagues] [6] may not be easy to assure.	1, 6
We know that [trouble at home can mean trouble at work and vice versa] [2], therefore whatever the source of distress, the [intervention could be useful] [1.]	2, 1
[I think to be useful][1] they [need interaction][1] and [guided discussion of some sort][7]	7, 1, 1
If offering holistic support, [this is essential] [1]. [Work and life are not separate] [2], in my opinion.	1, 2
[depends on how this is done] [9]	9
[Often such things are inextricably linked] [2]	2
I think it [should only focus on work based distress] [3.]	3
distress cannot be boxed and [work and life situations are inextricably linked] [2.] However the focus seems to be on work-based distress so this would need to be thought through carefully.	2
They are [often linked] [2]. Holistic in your approach.	2
might need to choose for it to be [work related mainly??] [3]	3
This appears a different source of distress, therefore [I question its inclusion] [3]	3
think this is a "holistic" approach to the stress that [some would welcome] [1] but others like to compartmentalise their lives so [would not want the cross over] [4.]	1, 4
They will be topics that are [not suitable for sharing] [3]	3
This would seem to be the [object of something like this] [1]	1
Probably easiest to [keep it Midwifery related] [3] although [this does include the stresses related to work / home life balance] [2.]	3, 2
[Depends what the outcome of this would achieve?][9]	9
[Working and personal stress have an impact on each other] [2]	2
[Focus must be on work-place causes of distress] [3.]	3
Again [online moderator important] [7]	7
The [need for support at all times is critical] [8]	8
The [two may be intertwined] 2 and interlinked so there [needs to be an avenue to	1, 2

discuss both] [1.]	
[May not have time at work to access the platform] [4]. [More private at home] [9.]	9, 4
The two elements [cannot be separated] [2] in professional life.	2
think it would be better if this had a [work focus][3] but obviously [life/work are inextricably linked] [2]	2, 3
Much of the peer support/ exchange is [likely to focus on this] [1].	1

	**Theme**	**Number of times Categorised**
1.	Discussions re: work or home based subjects of distress – A helpful inclusion	12
2.	Discussions re: work or home based subjects of distress - intertwined	12
3.	Discussions – unhelpful inclusion	8
4.	Discussions - Should be kept separate	4
5.	Discussions - uncontrollable	1
6.	Discussions – May risk anonymity/confidentiality	1
7.	Discussions – Require moderation	2
8.	Discussions – Require support	1
9.	Priority – Depends upon the context	3

Do you have any additional comments you would like to share?

**Comment**	**Themes assigned to**
It's [good to have somewhere] [1] to go with your dark thoughts?	1
NO	0
No	0
Whilst I appreciate that there [could easily be a spill over from work to home] [5]. I would think that it would [make the intervention a little messy] [3] to include home related stress impact on the midwife	5, 3
I'd be [concerned] [2] if someone became [more distressed] without proper support when sharing	2, 2
What facility will there be for peer moderation? [Likely to be important][4]	4

	**Theme**	**Number of times Categorised**
1.	Discussions re: work or home based subjects of distress – A helpful inclusion	1
2.	Discussions re: work or home based subjects of distress – An unhelpful inclusion	2
3.	Discussions re: work or home based subjects of distress – chaotic	1
4.	Discussions – Require moderation	1
5.	Discussions re: work or home based subjects of distress – Intertwined	1

18

An online intervention designed to support midwives in work-related psychological distress should prioritise an interface which does not resemble NHS, employer or other generic healthcare platforms

18.1 An interface which does not resemble NHS, employer or other generic healthcare platforms

Rank value	Option	Count
1	Not a priority	3
2	Low priority	2
3	Somewhat a priority	2
4	Neutral	14
5	Moderate priority	11
6	High priority	16
7	Essential priority	18

**Mean rank**	5.24
**Variance**	2.61
**Standard Deviation**	1.61
**Lower Quartile**	4.0
**Upper Quartile**	7.0

Consensus Achieved = No

Minimum score = Low Priority/Somewhat a priority 2 (3%)

Maximum score = Essential Priority 18 (27.3%)

An interface which does not resemble NHS, employer or other generic healthcare platforms

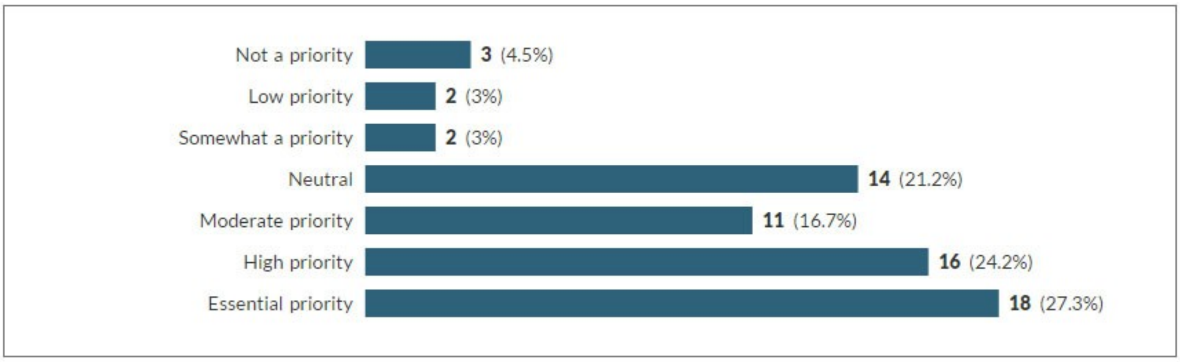

Thematic analysis of open text responses

Why did you choose this rating of priority?

**Comment**	**Themes assigned to**
I [don't know what the question intends to convey][7]	7
[Morally and ethically essential] [1].	1
[So there is no conflict of interest] [1] / [risk of being chastised by the public or NMC] [1] for the issues raised	1, 1

[won't use if think employer can access] [1]	1
Many [would not access if they felt that the employer had access] [1]	1
I feel that the platform [needs to feel very much removed from the workplace] [1]	1
In a way I think [a link to NHS or other professional organisation may provide reassurance] [2] that it is a 'safe' place to access support online and [healthcare workers may be more likely to feel comfortable][2] disclosing personal information online	2, 2
[user friendly is important] [6]	6
[I'm not entirely sure of this, since I have no knowledge of what platforms currently exist so cannot give an accurate answer] [8.]	8
If this is to be online and available to global midwives the [specifics of one country is not necessarily valid?][3, 4]	3, 4
Having the appearance/reality of a site totally independent of any employer [would be reassuring][1]	1
An Irish evaluation and ICM focus group identified a [clear need to keep this external] [1]	1
for reasons in 1-2 (I believe some midwives would be [fearful] [9] of people finding out they were finding it difficult to cope and would therefore [seek anonymity] [1] to feel safe to access support)	1, 9
the interface [could be NHS based] [2] and I don't think this would be a 'deal breaker' meaning it wouldn't be used...but [midwives might feel more comfortable sharing sensitive information if their employed didn't clearly set up the intervention!] [1] I have come across [great anxiety in midwives] [9] using the internet in this way, so anything to make them comfortable is a [good idea] 1.	2, 1, 9, 1
not sure [why this would be important] [7]	7
The platform would be more credible if the it [did not appear to be directly linked to an authority] [1.]	1
if they were [clear it was independent][1] then this [would matter less] [3, 5]	1, 3, 5
[Good] [1] to promote generic use	1
[As long as][1] it [easy to use and visually appealing] [6]	1, 6
agree [should look and feel very different to work and employer based interfaces] [1]	1
As long as it has the required information and is easy to use, [does it matter?][3]	3
to not be faced as an NHS or healthcare platform [would be better] [1] for my use of it - needs to be more like social media?	1
[To make it feel 'safe'.] [1]	1
[avoid potential triggers] [1]	1
This would be [useful to separate] [1] the nature of the interface as support not official.	1
[Main focus in that it is effective] [6]	6
A midwife feeling workplace related psychological distress is likely to feel powerless within "the system". A tool that feels nothing g like the workplace culture [may be a welcome environment] [1.]	1
[As long as] [1] [it is user friendly and easily accessible, not a chore to access or sign up to] [6]	1, 6
The disillusion can be from these sources making people [reticent to engage with a process that interfaces with that source] [1]	1
[Will be able to be used by midwives all over the world] [1] not just in the UK as this is an area that affects midwives worldwide.	1
[Needs to be supported by NHS and look professional] [10] but a [degree of separation may encourage midwives to access the platform] [1]. [A degree of separation could help with confidentiality and honesty] [1.]	10, 1, 1
[Should feel like it belongs to the user community] [1.]	1
[a high quality visually engaging platform is likely to encourage users to access, use & promote the resource] [6]	6

	**Theme**	**Number of times referenced in free text**
1.	Resemblance – Should be authority neutral	26

2.	Resemblance – Should be authority based	3
3.	Resemblance – Not important	3
4.	Resemblance - Variants on a global scale	1
5.	This would not matter if the intervention was clearly independent.	1
6.	Priority – user friendliness	5
7.	Question – relevance unclear	2
8.	Question – Cannot answer	1
9.	Midwives – Fearful of detection	2
10.	Intervention – Needs support of authorities	1

Do you have any additional comments you would like to share?

Comment	Themes assigned to
it [needs to appear visually as a safe supportive place to visit][1]	1
NO	0
This aspect is where the [confidentiality and anonymity is also important] [2]	2
[NHS too formal and too harsh?] [3]. The other platforms seem real and like reality? It seems like real [people are behind them not an employer] [3.]	3, 3
[Information however must be fed back] [4] so that issues can be addressed	4

	Theme	Number of times categorised
1.	Prioritise – visually safe space	1
2.	Intervention –confidentiality and anonymity important	1
3.	Resemblance – Should be authority neutral	2
4.	Intervention - Consider analysing feedback	1

19

An online intervention designed to support midwives in work-related psychological distress should prioritise a simple, anonymised email login procedure which allows for continued contact and reminders which may prompt further platform usage

19.1 A simple, anonymised email login procedure which allows for continued contact and reminders which may prompt further platform usage

Rank value	Option	Count
1	Not a priority	3
2	Low priority	1
3	Somewhat a priority	2
4	Neutral	7
5	Moderate priority	20
6	High priority	18
7	Essential priority	15

Mean rank	5.33
Variance	2.16
Standard Deviation	1.47
Lower Quartile	5.0
Upper Quartile	6.0

Consensus Achieved = No

Minimum score = Low Priority 1 (1.5%)

Maximum score = Moderate Priority 20 (30.3%)

A simple, anonymised email login procedure which allows for continued contact and reminders which may prompt further platform usage

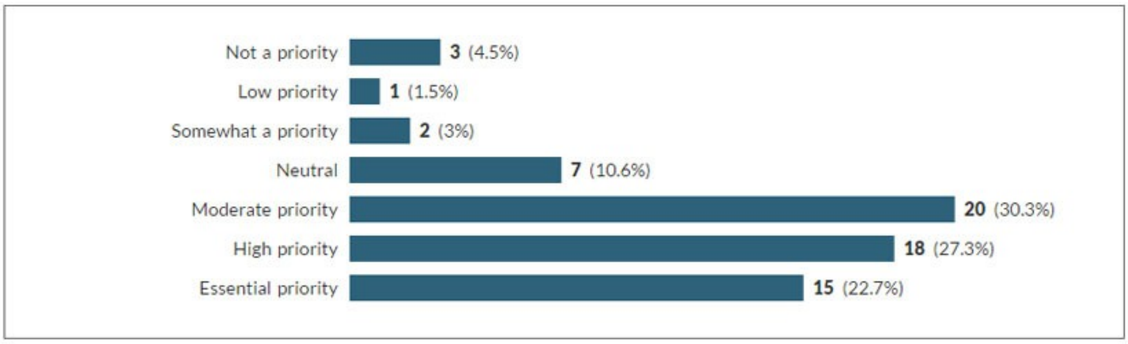

Thematic analysis of open text responses

Why did you choose this rating of priority?

Comment	Themes assigned to
[I don't know what the alternatives are] [6]	6
[As long as] [1] the user has the [option] [3] to opt out of reminders or set how frequently they would like them so that they don't [become irritating] [2.]	1, 3, 2
If you are going to do it [need to get it right] [1] and [user friendly] [4].	1, 4
an [essential priority] [1] to encourage regular usage and [notify the user that there has been activity they may wish to contribute to][5]	1, 5
This is a [really good idea] [1], could provide the [opportunity for the person to monitor their psychological wellbeing on an ongoing basis] [1]	1, 1
[Good idea] [1] in principle however the timing of reminders [may annoy/disengage individuals] [2] if they are too frequent.	1, 2

[Reassuring login system] [1]	1
if [too complicated][2] would not be used	2
I'm not sure about reminders to use, it [could come across as spammy?] [2] It [would have to be very supportive in tone] [1, 4.]<br><br>[An easy log in and easy to use interface] [4] [couldn't be more essential] [1], as many midwives are from a generation which find technology difficult.<br><br>Also, I find in my experience as a midwifery writer online who also provides an online training programme, that many people using the internet become very impatient and have a short attention span - google and other distractions are only a click away, so [if it's not near instantly engaging, you'll miss out on users] [4.]	2, 1, 4, 4, 1, 4
[probably useful][1]	1
These could be read by others. So [not ideal] [2]. No need and a lows the midwife to look at the platform without some longer term reminder. This [would annoy me and become part of my junk mail] [2.]	2, 2
keep it [simple and easy to use] [4]	4
[Ongoing support is usually required] [5] as the period of stress begins to reduce	5
[Easy access is key for use] [4]	4
this [would be useful] [1] but [not too bombard] [2] the user as the frequency of use may be a sign of recovery or lessen of need?	1, 8
will the users then be [reluctant to reveal thins] [2] as they feel they are being followed up?	2
[sounds good] [1.] This could create anonymity.	1
If this is what users would find useful [yes] [1.] Possible [better to focus on local support].[7]	1, 7
[some provision] [5] for follow up and potential evaluation is important	5
[Ease of use is essential] [4]	4
[If the distress is that severe, the midwife will remember to engage with the resource] [2].	2
[Good idea] [1]	1
[Allows for support] [1] but person also needs to move on and feel recovered	1
Busy midwives have so much already coming into their email inboxes – [could stress them out] [2] more with more email so a bit uncertain about this one.	2
[People may want privacy] [2]. If a midwife is under distress [more e-mails will not help] [2.] Let them access support as needed and required.	2, 2
I think prompts are [counterproductive] [2] to foregrounding choice and autonomy.	8
I think it's a [high priority][1] but [may compromise anonymity if linked to email address] [2]	1 , 2
[Agree][1] with need for simple anonymise log in but [not sure reminders to prompt further usage are good idea] [8] - when you are under pressure to get through your emails in limited time the [last thing you want is more reminder emails adding to the load][2]	1, 8, 2
Reminders may have [positive] [5] or [negative] [8] effect. Would work better to [use alerts to new resources/ self-help] [1.]	5, 8, 1
[Easy access is essential] [1, 4] to support continued engagement – [access from a mobile phone as well as tablet & computer needed] 4	1, 4, 4

	**Theme**	**Number of times referenced in free text**
1.	Anonymised email login procedure - A helpful inclusion	19
2.	Anonymised email login procedure - An unhelpful inclusion	13
3.	Anonymised email login procedure – must be optional	1
4.	Priorities – A user-friendly intervention	9
5.	Prompting – A helpful inclusion	4
6.	Anonymised email login procedure – Unsure of alternatives	1
7.	Midwives – May require alternative support	1
8.	Prompting – An unhelpful inclusion	4

Do you have any additional comments you would like to share?

Comment		Themes assigned to
[as long as confidentiality is upheld][1]		1
NO		0
	**Theme**	**Number of times referenced in free text**
1	Confidentiality must be upheld	1

20

An online intervention designed to support midwives in work-related psychological distress should prioritise an automated moderating system where ‘key words’ would automatically initiate a moderated response

20.1 An automated moderating system where ‘key words’ would automatically initiate a moderated response

Rank value	Option	Count
1	Not a priority	3
2	Low priority	3
3	Somewhat a priority	4
4	Neutral	21
5	Moderate priority	15
6	High priority	7
7	Essential priority	13

Mean rank	4.74
Variance	2.52
Standard Deviation	1.59
Lower Quartile	4.0
Upper Quartile	6.0

Consensus Achieved = No

Minimum score = Not a Priority/Low Priority 3 (4.5%)

Maximum score = Neutral 21 (31.8%)

An automated moderating system where ‘key words’ would automatically initiate a moderated response

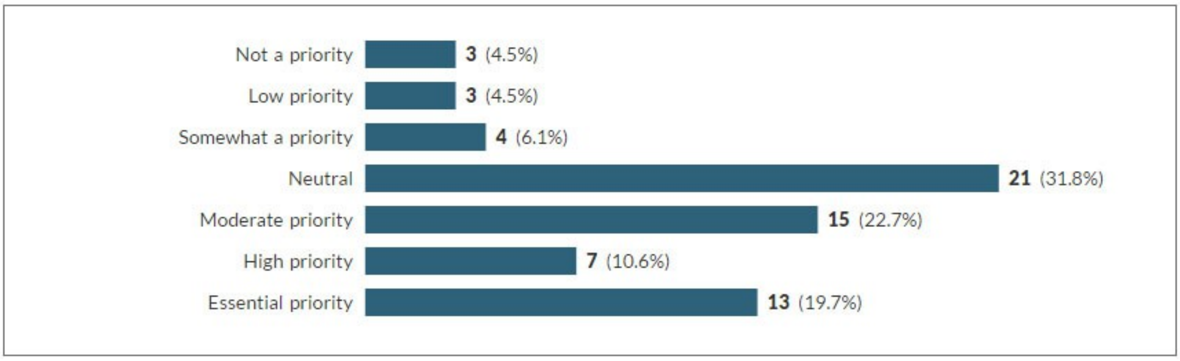

Thematic analysis of open text responses

Why did you choose this rating of priority?

Comment	Themes assigned to
We know that intelligent systems that make users feel more is known about them than they know about the sender generates paranoia-[not likely to be helpful] [2.]	2
[Not sure how well this would work] [4] as language is such an individual thing?	4
[don't know]4	4
There definitely [needs to be some moderation] [3] to keep the users safe	3
[not sure what this question means][9]	9
Many forget that electronic communication is stored and their [words may come to haunt them] [2]	2
The platform [needs to be regulated] [3] to avoid inappropriate posts and language	3
All posts/responses [should be moderated] [3] as an [automated system wouldn't be reliable] [6] enough in this situation	3, 6
[Yes][1] I agree that key words ensure safety	1
This [question needs expansion] [9] before I can provide an answer.	9
I think it [can simplify for some] [1], but [can be considered not serious enough for others] 6	1, 6
? Automatically initiate a moderated response – [how can a response be moderated and automatic?] [9]	9
[Unsure of this process][9]	9
[not sure what you mean by this][9]	9
This [could be very effective] [1] if done well - it would [need to be supportive in tone] [5]. I would also [make it clear it was an automated response] [7], and explain the reasons. Users [can become offended] [2] if they think they're talking to a real moderator and then find it's automated.	1, 5, 7, 2
[vital that key words like "suicide" are picked up] [1, 10]	1, 10
Emerging evidence that [references to suicide] [10] and self-harm such as graphic images, sensationalism, accounts of methods [can promote further self-harm and suicide] [2]. (Contagion.) Emerging evidence that young people in particular may be more vulnerable to this effect. [Moderation vital] [3] to offer to support to the person in distress and also to ensure safety of others in the chat.	2, 3, 10
[Don't really understand what This means][9.]	9
In my experience automated responses [do not work well] [2] and are [not always appropriate] [6]. Systems that detract from a caring, human response [may not be helpful] [2] and [may even be counterproductive] [2.] It would really [depend on how sensitive/cleaver the automated system was] [6.]	2, 6, 2, 2
[need to know more about this] [9] and if possible – [sounds sensible][1] if [triggers such as suicide][10] or other are noted but [need more info please] [9]	9, 1, 9, 10
[Seems impersonal somehow][2]	2
this [makes the interface less human] [2]	2
A space where so many people could potentially share their most traumatic experiences [needs to feel safe] [1]. They also [need to be able to post without expecting judgement, criticism or trolling] [3.]	1, 3
I am [not clear what is meant here] [9] -do you mean that a keyword would be flagged up and trigger a personal response? [yes support this] [1]	9
[Possibly] [4] – [not sure how this would work] [6]	4, 6
[not sure what this means] [9]	9
[Nott sure how automated response would help] [4] with someone's psychological distress. Would [have to be a very sophisticated system] [6.]	4, 6
[Not sure how this would work?] [6]	6
[not clear that this is effective] [4]	4
May make an [efficient response][1] and [save time][1] and [prevent the user from giving up][1]	1, 1, 1
[I would like to know an alert would be raised] [9] if a platform [user mentioned words related	9, 10

to suicide] [10].	
[Great] [1]	1
[Allows for support] [1] but person also needs to move on and feel recovered	1
There [needs to be a mechanism] [1] to [identify those requiring] 8 a higher level of support due to higher distress level.	1, 8
[Could be interesting] [1], however [honesty maybe withheld if midwives feel they are being monitored] [2]	1, 2
[would feel a bit 'big brother'] [2] if you introduced this - people [may feel their anonymity could be under threat] [2]	2, 2
[Maintain user safety] [1]	1
Participants likely to [value real interactions over automated ones] [2]. High [risk of inappropriate intervention] [2, 6]	2, 2, 6
the [system needs to offer a high degree of safeguarding] [6, 1, 8]	6, 1, 8

	**Theme**	**Number of times referenced in free text**
1.	‘key words’ initiating a moderated response – A helpful inclusion	14
2.	‘key words’ initiating a moderated response – an unhelpful inclusion	13
3.	‘key words’ initiating a moderated response – Moderation is required	5
4.	‘key words’ initiating a moderated response – conflicted	5
5.	‘key words’ initiating a moderated response –must be supportive in nature	1
6.	‘key words’ initiating a moderated response – May not be adequate	8
7.	‘key words’ initiating a moderated response – Confusing	1
8.	Midwives – Need to be risk assessed	2
9.	Question – Need to know more	11
10.	Midwives – Must be protected from suicide	3

Do you have any additional comments you would like to share?

**Comment**	**Themes assigned to**
What system could [account for individual expression and regional differences in idiom?] [1]	1
NO	0
If you mean the question would be sent to a moderator if key words were entered, then [yes, that would be a high priority] [2] - the question confused me in that instance	2
Need to be careful about how many words you list in this function, [the moderator could be inundated!] [3]	3
however can see the [need for this moderation] [4]	4

	**Theme**	**Number of times Categorised**
1.	‘key words’ initiating a moderated response – Must be sophisticated	1
2.	‘key words’ initiating a moderated response – A helpful inclusion	1

3.	‘key words’ initiating a moderated response – Moderation = high maintenance	1
4.	‘key words’ initiating a moderated response – Moderation required	1

21

An online intervention designed to support midwives in work-related psychological distress should prioritise mobile device compatibility for platform users

21.1 Mobile device compatibility for platform users

Rank value	Option	Count
1	Not a priority	1
2	Low priority	0
3	Somewhat a priority	0
4	Neutral	6
5	Moderate priority	12
6	High priority	20
7	Essential priority	27

Mean rank	5.97
Variance	1.33
Standard Deviation	1.15
Lower Quartile	5.0
Upper Quartile	7.0

Consensus Achieved = Yes (High Priority/Essential Priority) 71.2%

Minimum score = Low Priority/Somewhat a priority 0 (0%)

Maximum score = Essential Priority 27 (40.9%)

Mobile device compatibility for platform users

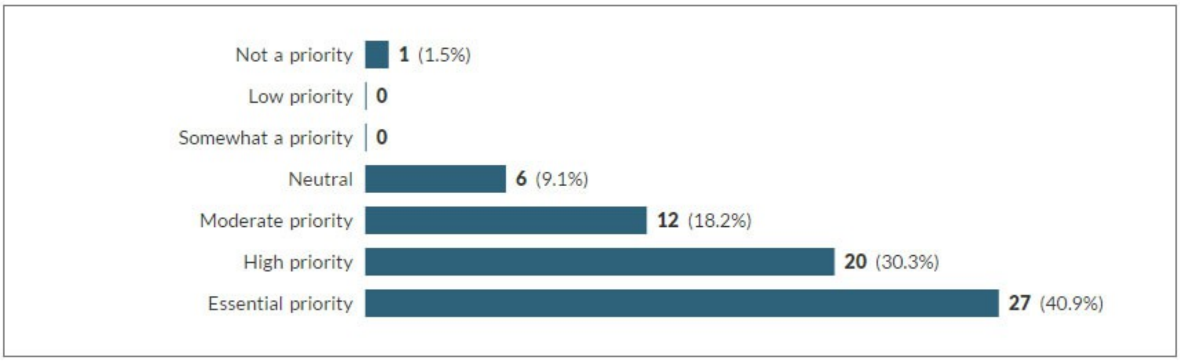

Thematic analysis of open text responses

Why did you choose this rating of priority?

**Comment**	**Themes assigned to**
If you build it, it [needs to work] [3]	3
Personally I use my mobile for all online activities as I rarely have access to a laptop or pc due to the nature of my role. [Mobile compatibility is essential] [1.]	1
well if its work based stuff you [need your own "terminal"] [1.]	1
[Essential] [1] in this time as many people use their phones/tablets etc.	1
An app would be of [great benefit] [1] to many who are unable to access a PC	1
[Yes] [1] anything to improve uptake and usage should be considered	1
[Yes] [1] will improve access anywhere at anytime	1
Being able to have support immediately to hand can be a [big benefit] [1] to some	1
[Very important] [1] as individuals can access support and advice anywhere and whenever/wherever if access to a pc/laptop/tablet is limited/restricted.	1
it will [offer more use] [1] of the online intervention	1
[Available to more people] [1] and allowing online access from mobile devices is [important for this] [1.]	1, 1
That sounds like a really [good idea] [1], as long as the [phone number wasn't able to be identified] [5]	1, 5
Midwives are on the go so [mobile use is important] [1]	1
it’s the [way of the world] [1]	1
90% of my traffic is from mobile devices, my market research suggests a huge amount of [midwives use the internet in this way] [1] above all others.	1
[Important][1] for gen x, y and z	1
[If I cannot use this on the go on my smart phone I'm unlikely to use it] [1]. My phone is my main personal research tool. In fact it's [my main everything tool] [1] as is often the case with today's society. I can now do all my clinical notes directly onto my phone in a secure app that has all my details for each client at my fingertips. This [makes being on call 24-7 much easier] [1.]<br>As all I need on the go is my phone.<br>So regarding my own personal wellbeing all tools [need to be phone usable for me][1].	1, 1, 1, 1
how we live and communicate their days and any [platform needs to feed into this] [1]	1
[most people have smart phones with them at all times] [1] - again make it [easy and accessible] [3]	1, 3
I suppose in today’s electronic times it [would be useful] [1] for access it wherever even at work or when not at home.	1
think in terms of access then [mobile is very current][1.]	1
[To know that you can access this support wherever you need to] [1.]	1
[Definitely] [1] so that it is [easily accessible] [3]	1, 3
[Increase accessibility and usage] [1, 3.]	1, 3
[Good] [1] to [access from anywhere] [3]	1, 3
[Most likely to be used] [1] in a mobile environment.	1
[Another good idea] [1] - most access personal mobile phones more regularly than, especially, a work computer	1
Most midwives on call develop an aversion to mobile devices I think therefore this [would become another source of pressure] [2]	2
[Modern midwives have their smart phones and use them constantly] [1].	1
[Easy access is essential] [1,3]	1, 3
[Most are on smart phones now][1]	1
staff are [often not able to get confidential access pcs easily] [1] -at work or home	1
[Should be platform neutral] [4]	4
Professionals [may wish to access whist mobile] [1] i.e. in work, during travel etc.	1

	**Theme**	**Number of times Categorised**
1.	Mobile device compatibility – High priority	35
2.	Mobile device compatibility - Unhelpful	1

3.	Mobile device compatibility – Must work	6
4.	mobile device compatibility – Neutrality important	1
5.	mobile device compatibility – Must be secure	1

Do you have any additional comments you would like to share?

Comment		Themes assigned to
NO		0
I think that the users need to have a real sense of [follow-up support] [1]; I am concerned that the platform may raise issues that the [users are unsupported] [1] in dealing with. There are [no guarantees that they will seek other support] [2] and this [makes the venture risky] [3].		1, 1, 2 , 3
	**Theme**	**Number of times categorised**
1.	Midwives – Require support	2
2.	Midwives – may not seek alternative support	1
3.	Intervention - Risky	1

**If there are any new questions that you would like to be put forward during the second round of questioning, please list them below.**	**Action taken**
There is nothing about the nature of 'trauma' that is referred to and nothing about why and how people would be asking for help via this mechanism. While an intervention may already have been chosen, there is more than tool design involved in making something like this work.	Psychological distress defined in Round 2 survey introduction
How to cherish any human sounding board you may have!	0
NONE	0
Could friends/family have access/be signposted to support services?<br><br>Would this platform be available 24/7?	24/7 support refined into question for next round.<br>Friends and family access refined into question for next round
How does the 'system' follow up those who are identified as being at risk?	following up and 'identification of those at risk refined into question for next round
Obtain permission for midwives using the online supports to evaluate and trend concerns to highlight same at national level	obtaining permission from users to evaluate and trend concerns in order to highlight them at a national level refined into question for next round
Do you think that a quick assessment using a	The implementation of an initial simple user

scale such as the Kessler psychological distress scale (K10) should be administered so that if the midwife is experiencing significant distress this attracts an immediate moderator response?	assessment using a psychological distress scale to prompt the user to access the most suitable support available refined into question for next round.
would like to know more about ideas and especially moderator etc.<br><br>Also sorry to say while I like the logo / cartoon idea I don't like the images of the midwives - too sexualised to be acceptable / recognisable.	Moderator role put forward in 2 questions for next round. Proactive/reactive moderation questions posed.
This is an excellent idea, however will it allow midwives to access this support in the work environment? I was thinking about fire walls which may not allow access	24/7 support refined into question for next round.
A very clear aim of what you are trying to set up would be helpful. what types of psychological distress? What is the need for this?	Psychological distress defined in Round 2 survey introduction
I feel concerned that the extreme seriousness of this for some practitioners is still not fully understood, for a high percentage it can be life threatening	0
NONE	0
A question about the role of the moderator and confidentiality	Moderator role put forward in 2 questions for next round. Proactive/reactive moderation questions posed.
How much do you know of the diversity of the user community, both in terms of perceived need and user experience? How might intervention address these diverse needs effectively without putting some off?<br><br>Safeguarding and peer moderation to ensure appropriate use/ signposting needs consideration.	Moderator role put forward in 2 questions for next round. Proactive/reactive moderation questions posed.
