# Supplementary material for: Achieving Consensus for the Design and Delivery of an Online Intervention to Support Midwives in Work-Related Psychological Distress: Results From a Delphi Study
Source: JMIR Ment Health. 2016 Jul 12;3(3):e32. doi: 10.2196/mental.5617 (PMC4961877; doi:10.2196/mental.5617)
Supplement: Multimedia Appendix 6 [file mental_v3i3e32_app6.pdf]

Delphi Study Analysis Report: Delphi Study to achieve Consensus in the Development of an Online Intervention Designed to Effectively Support Midwives in Work related Psychological Distress.

Round Two

Following a preliminary round of questioning, a second round of questioning commenced during the autumn of 2015. This report analyses and presents the findings of this second round of a Delphi study, designed to achieve consensus in the development of an online intervention designed to support midwives in work-related psychological distress.

66 participants were invited to take part in this second round of Delphi study. These participants had already completed a preliminary round of questioning, and had been informed that a second round of questioning would be sent accordingly. Initially, these participants were sent a report both thanking them for their participation and detailing all of the data findings from the first round of questioning. They were then asked to read and deliberate upon these results prior to being invited to participate within a second round of questioning. This second round of questioning was developed in response to the first round of questioning. It invited participants to answer previous questions again, where a consensus had not initially been achieved. It also presented new questions, which had either been inspired by the data collected, or suggested by the expert panel of participants within round one of this Delphi study. A total of 19 questions were put forward within this second round of questioning.

44 participants (66%) completed the second round of questioning in full. The purpose of this second round of questioning was to gain further insights and achieve further consensus in

the development of an online intervention designed to support midwives in work-related psychological distress. Consensus has only been confirmed where at least 60% of respondents have arrived within 2 adjacent response points on the 7-point scale. This report details the results gathered from this second round. Please note that spelling mistakes have been corrected accordingly.

1

All participants gave their full consent so that the research team could verify that participants fitted the inclusion criteria, had understood the participant information in full, had given their consent for their anonymised quotes and results to be used for publication, and had given their full consent to participation.

2

An online intervention designed to support midwives in work-related psychological distress should prioritise amnesty for all platform users in that they will not be referred to any law enforcement agencies, their employer or regulatory body for either disciplinary or investigative proceedings in any case.

2.1 Amnesty for all platform users in that they will not be referred to any law enforcement agencies, their employer or regulatory body for either disciplinary or investigative proceedings in any case

Rank value	Option	Count
1	Not a priority	2
2	Low priority	7
3	Somewhat a priority	3
4	Neutral	7
5	Moderate priority	8
6	High priority	9
7	Essential priority	8

**Mean rank**	4.61
**Variance**	3.37
**Standard Deviation**	1.84
**Lower Quartile**	3.0
**Upper Quartile**	6.0

Consensus Achieved = No

Minimum score = Not a priority 2 (4.5%)

Maximum score = High Priority 9 (20.5%)

Amnesty for all platform users in that they will not be referred to any law enforcement agencies, their employer or regulatory body for either disciplinary or investigative proceedings in any case

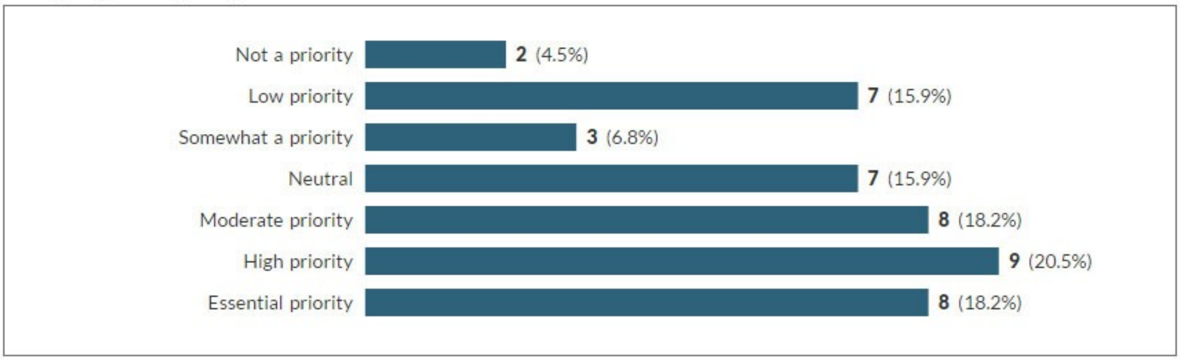

Thematic analysis of open text responses

Why did you choose this rating of priority?

Comment	Themes Assigned to
I'm not neutral but [don't know how else to answer][6]-there are [always boundaries that must not be crossed][4]-without knowing what could be disclosed on such a site it is not possible to make an assessment on this criterion.	6,4
so the midwife will not [feel frightened to discuss][2] the event and [express her feelings][1]	2,1

People [need to feel safe][1] that what they share is able to be shared in a forum that remains confidential; [fear of reprisal][2] may [stop someone speaking][1] about something that is impacting their sense of wellbeing - they may feel guilty, but not be guilty of any wrongdoing	1,2,1
It is [difficult to require an amnesty][6] as it may be something that [requires further investigation][4]	6,4
People are [not going to be fully revealing][1] if they believe they will [suffer as a result!][2]	1,2
this is [a difficult one][6] as it depends on the situation and hard to see how it could be otherwise e.g. [if gross misconduct / ongoing harm][4]. For others it may be appropriate to wave legal aspect as [gives opportunity for change][5]	6,4,5
any organisation that collects information which is seen to breach the law would have [difficult in not reporting these exceptional situations][4].	4
it is [dependent on the situation][4]	4
I am still [not sure][6] that this can be legally done. For example, if the midwife is dealing with a woman who confesses child abuse to her then she would be [legally obliged to report][4] this and it may therefore be better to say, don't say anything that may be illegal requiring us to refer the matter on	6,4
This will [protect their personal privacy.][7]	7
Although platform users [should be able to freely discuss][1] their concerns, it [should not be hiding criminal activity.][4]	1,4
[Encourage access and honesty][1]	1
Clearly if the registrant felt that they would be referred to any agency they [would not seek help from an online platform][1]	1
unless users are [honest open and transparent][1], such intervention is useless. [Only way to seek honesty][1] is by protecting them	1,1
If you're going to provide a safe and supportive online space - [midwives need to be assured][1] that they will [not face any legal recriminations as a result of speaking freely and openly][1]	1,1
[It almost suggests that there may be grounds for this route to be considered][3]	3
[Freedom for practitioner to reflect][1] and [provide a safe space][1]	1,1
Midwives have [few other places they can offload][8] this kind of concern; it's likely some [midwives won't ask for help unless they have this assurance][1].	1,8
I think that the [fear of disciplinary action][2] may [prevent people from participating][1] but it [may be the thing they need][1] to address issues and take action themselves.	2,1,1
As a platform for help, [people should be free to share][1] their thoughts and feelings without [fear of it going though to their work place][2], friends etc. (as this maybe the source of their issues). However, [if a law is being broken then it is unethical to not report it.][4]	1,2,4

You could have someone admit to severe and damaging ongoing abuse of patients or illegal practice; [where would you stand][6] when you read this and know that it was ongoing and that people were continuing to be put at risk?	4
if as a midwife you have done something illegal or questionable in the legal sense, you really [are very unlikely to post on a platform like this]1. However if you do then this can add to the case either against you or for you. But [if you have engaged in criminal activity, this needs to be referred to the regulatory body.][4]	4,1
To allow users the [opportunity to get help][5] and support without [feel of reprisal][2]	2,5
In order to meet the aims of the platform ensuring confidentiality is essential to provide a [safe space in which to debrief][1]. [Fear of reprisal, recrimination or sanctions][2] would [be counter productive][5].	1,2,5
I think this requires [careful consideration][6] in order to [uphold law and prevent further harm][4]	6,4
[Amnesty is important to an extent][1], however, users must be aware that any disclosure which breaks the law (not the Code) [must be referred to law enforcement agencies][4.]	1,4
This should be like a counsellor and have the same confidentiality [so that midwives will use it][5]. If they [fear being reported][2] to any agency they [may be reluctant to use the service][1].	5,2,1
[I think this is important][1] but if absolutely not possible an online intervention could still be useful (this is why I have not scored 'essential priority'. But I do think that it [needs to be free from fear of reprisals][1, 2]. It could achieve this by being anonymous - no names and no places mentioned.	1,1,2
[if they will be fair][0]	0
[I am not sure about the balance][6] between public protection and allowing staff to have a safe place to disclose their concerns.	6
[Midwives need support][8] in decision making and supporting women, much of this is online now due to increased workload, no cohesion in teams, poor leadership	8
In counselling, the notion of [confidentiality is conditional][4] and the conditions of confidentiality are carefully explained	4
Needs to be [considered on an individual basis][4], especially [if negligence or criminal activity is evident][4]	4,4
Anonymity is a luxury we [cannot afford to offer in cases of serious misconduct][4], or where there is [any possibility of harm to the user or another person][4]. At its most serious, workplace stress may lead to such things. Whilst [anonymity may well benefit user engagement][1], this must [only be guaranteed where no serious risk is present.][4]	4,4,1,4
[Without amnesty people may not use the site][5] and therefore a situation may get worse, however, [issues which require action or follow up][4] for reasons of safety may be revealed. It's a [catch 22 situation][6]	5,4,6
Likely to be [important in encouraging participation][5].	5
Because it [needs clear discussion][1]	1

I think it [depends on context][6] would we be collecting identifying data - [how could we refer] [6]- who would be responding - would there be real time response - therefore I chose this response as in the absence of the front end information required this seemed the most appropriate	6,6
I feel [unclear as to how to implement this] [6]. It defeats the object of being [a safe supportive tool][5] if a user [felt inhibited that they may be reported][2]. Whilst also recognizing [a duty for any body to address unsafe practice.][4]	6,2,5,4
[otherwise no one will used the platform][1, 5]	1,5
There [needs to be exoneration][1, 5] but where public safety is at risk there [must also be a professional accountability][4]	1,5,4

	**Theme**	**Number of times categorised**
1.	Amnesty – Required for open and honest disclosure	27
2.	Midwives - Fear retribution	10
3.	Amnesty – Cannot be given in any circumstances	1
4.	Amnesty - Cannot be given in all cases	21
5.	Amnesty - Required for change/Help seeking	9
6.	Amnesty - Conflicted in opinion	10
7.	Amnesty - required for privacy	1
8.	Midwives - Have little support	2

Do you have any additional comments you would like to share?

**Comment**	**Themes Assigned to**
This situation is a [tricky one][6] because if the person truly has done something illegal or out of line professionally, the [moderator may feel compromised][5] if an amnesty is in place. However, the moderator can always refer to professional codes of conduct and ask the kind of questions that can [encourage the person's sense of professional responsibility & self-disclosure][8] to their employer/regulatory authority etc.	6,5,8
The [balance between protecting staff and protecting the public needs to be explored more][6]	6
It is essential that they will be [exceptional occasions when reporting is required][3] at the outset of any tool/resource	3
The [ability to discuss concerns without reprisal from employers is essential][1], [but what would be done if there were vulnerable adults or children identified][6] (other than the platform user).	1,6
sometimes you can’t see a situation clearly when you are in the middle of it so [need a non-biased medium is helpful][1]	1
It would be a case of the [extreme potential spoiling what would actually be helpful][6]; [a space where you could be utterly honest and unburden][1] without [fear of reprimand][2].	1,2,6

I am [not sure I understand the gist of this question][9] which may be why you didn't receive consensus on it originally.	9
[A clear set of Terms and Conditions must be read and agreed][4] prior to use which gives clear rules regarding disclosure of illegal activities.	4
no	0
The amnesty may have to be [conditional upon certain criteria][3] which are [carefully explained and adhered to][4] and in line with professional codes of conduct	3,4
However, there [might be circumstances when there is a duty to refer][3] e.g. over a safeguarding concern. Suspect there [cannot be a guaranteed amnesty in all circumstances][3].	3,3
[I am not comfortable][6] that people could be discussing something where they have clearly been involved in a situation that could put future people they are caring for at risk. It would also [add stress to those who are online with this person][7] of carrying this burden of responsibility.	6,7
If practitioners are obviously so distressed that their judgement/health is impaired to a point they could cause harm to self or others there [needs to be some mechanism that enables protection][8] - I [don't think its about reporting them][8] to the regulator - but there needs to be a [safe supported way][8] that enables the practitioner to develop self awareness and feel safe to seek help	8,8,8

	**Theme**	**Number of times categorised**
1.	Amnesty - A helpful inclusion	3
2.	Midwives - Fear retribution	1
3.	Amnesty - Cannot be given in all cases	4
4.	Intervention - Requires disclaimer policies	2
5.	Amnesty - Difficult to moderate	1
6.	Amnesty - Conflicted in opinion	5
7.	Amnesty - An unhelpful inclusion	1
8.	Amnesty can enable resolution of situations	4
9.	Question - Meaning unclear	1

3

An online intervention designed to support midwives in work-related psychological distress should prioritise prompting platform users automatically to remind them of their responsibilities to their professional codes of conduct.

3.1 Prompting platform users automatically to remind them of their responsibilities to their professional codes of conduct.

Rank value	Option	Count
1	Not a priority	8
2	Low priority	7
3	Somewhat a priority	2
4	Neutral	4
5	Moderate priority	6
6	High priority	9
7	Essential priority	8

Mean rank	4.18
Variance	4.88
Standard Deviation	2.21
Lower Quartile	2.0
Upper Quartile	6.0

Consensus Achieved = No

Minimum score = Somewhat a priority 2 (4.5%)

Maximum score = High Priority 9 (20.5%)

Prompting platform users automatically to remind them of their responsibilities to their professional codes of conduct.

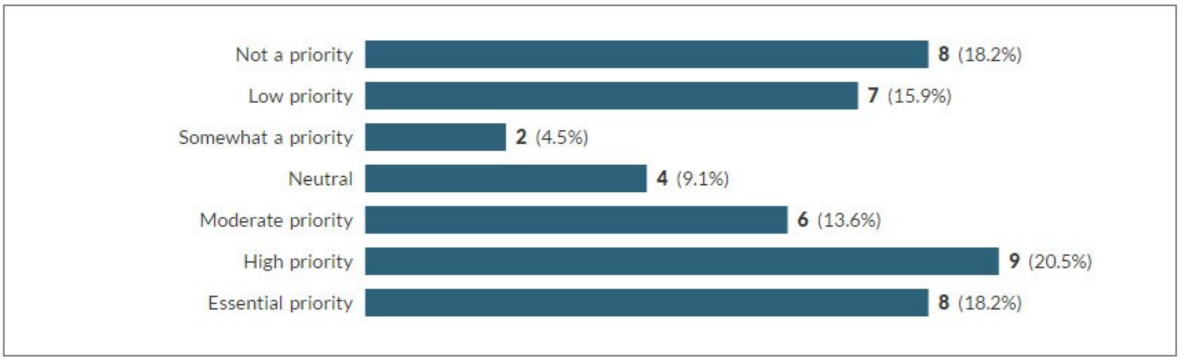

Thematic analysis of open text responses

Why did you choose this rating of priority?

Comment	Themes Assigned to
[Professional codes do not currently give enough attention to self care of the carer] [7]- [reference to the duty of care to self would be more viable][8].	7,8
it is [important that the midwife will know her responsibilities and professional codes at all time][1]	1
[People need reminding and refreshing about their professional responsibilities and accountabilities][1]	1
[An important aspect of support][1]	1

[Mandatory][1, 3]	1,3
[we would be complicit if didnt point this out to them][1, 3]	1,3
[It is an ethical & legal requirement][3] in keeping with the NMC Code of Conduct	3
there is still [an obligation to adhere to their professional codes of conduct][3]	3
I still see this as [a low priority][2], it [doesn't mean it isn't important][1] juts that its a low priority compared with other things you are wanting to do with this platform	2,1
[As professionals they already know and practice following the codes][4].	4
This sounds like the NMC and [would make me reluctant to discuss concerns openly][2].	2
[We must adhere to our code at all times][3]	3
This [may prompt the midwife to seek help from a peer or their supervisor][1]	1
To [ensure that they have a good self awareness][1]	1
If the aim is to support midwives in work-related psychological distress, [reminding them professional codes of conduct will be counterproductive][2]	2
[Could be perceived as a bit big brother][2]	2
It [may be prompt required for someone to take 'time out' and not struggle on and run the risk of making mistakes][1.]	1
[Identifies self accountability][1] [honours safety][3]	1,3
This is a [good idea][1] - so midwives can be reminded of specific responsibilities, but it would have to be done very carefully, worded very carefully [so midwives in psychological distress don't feel even worse/shame][2]. I've [yet to meet a midwife who doesn't know their responsibilities][5] - [I've met many in need of psychological support.][9]	1,2,9,5
[Midwives already know their professional codes of conduct][4] for their own country and I suspect these are different around the world.	4
[A reminder is always helpful.][1]	1
Sounds like a [guilt- inducing feature][2]. If my self confidence were already low and I didn't feel like I'd been able to perform to my best, an automatic reminder of where I *should* be and would probably highlight those inadequacies to me and [lower my self esteem further][2].	2,2
we [all need reminders from time to time of our professional responsibilities][1]	1
Users should be [aware of their responsibilities to the code and shouldn't need reminding][4]	4
Again I feel that actions such as this [will create fear amongst midwives][2] and thus make the service inoperable as it [may seem as a reprimanding reminder][2].	2,2
Just have link to Code? [Constant reminders might become obtrusive][2]	2
I [don't think that midwives in psychological distress need reminding of the Code during the use of a support platform][2].	2

Again - if this in place of face to face counselling - it should be [confidential and non-judgemental][2]. [Once the crisis has been dealt with then any professional issues can be dealt][8] with - eg a requirement to report certain things that may have been done which were not in compliance with professional codes of conduct.	2,8
[Yes it is still the case that they need to be mindful of this.][1]	1
[since it will be act of professionalism][1]	1
[because patient safety is imperative][1, 3]	1,3
[A good idea][1], we are all so busy + have little time to refer to code etc and the code has changed and possibly will again due to changes in SoM	1
If the priority is to address their work-related psychological distress, this is [best not diluted by automatic reminders that shift the focus][2]	2
All [registered health professionals have a duty of care to their patients and this should be at the forefront of their minds prior to engaging in any form of patient contact][6], [especially if they have concerns regarding their emotional and mental well being][6].	6,6
[This is what we all signed up to][3] and [serves to protect the professional as well as the patient][3].	3,3
[Helpful to remind people of professional duties][1].	1
same reasons as above - [prompts should be to seek help][8] - where to get help and then perhaps say why but [prompts to regulation could make people feel worse][2]	8,2
This [should not be the function of of this platform][5, 8]	5,8
[This is about the midwife not her professional responsibilities][5]	5

	**Theme**	**Number of times categorised**
1.	Prompting professional codes - A helpful inclusion	18
2.	Prompting professional codes - An unhelpful inclusion	14
3.	Prompting professional codes - Ethically essential	9
4.	Midwives - Already aware of codes - Not required	3
5.	Prompting professional codes - Not the purpose of the intervention	3
6.	Midwives - Duty of care should be priority	2
7.	Codes - Inadequate	1
8.	Prompting professional codes - Alternative approach required	4
9.	Midwives - Need support	1

Do you have any additional comments you would like to share?

**Comment**	**Themes Assigned to**

[Providing a reminder about professional codes of conduct is essential][1] and ensures integrity for the platform provider; [just because someone is emotionally upset doesn't mean they should lose their professional integrity][5]	1,5
[Midwives in psychological distress are already highly aware of their responsibilities] [3] - thats often why they are the ones who have the distress in the first place	3
I do however think that [it would be valuable to always have a link to these visible][4]. An [automatic prompt sounds very 'in your face'.][2]	4,2
[if as a midwife you are suffering such distress that you need reminding of your professional code of conduct, maybe you need some time off and shouldn't be working?][6]	6
Again, [a reminder of the code should be included within the Ts&Cs][4]	4
The word automatically worries me. I think [automated reminders can turn out to be more irritating than helpful or appropriate.][2]	2
no	0
Sometimes, [a reminder of the Code can be enough to make sense of a situation][1], or [bring clarity of thought to a complex situation].[1]	1,1
[Needs to be done carefully, so is not intrusive][7]	7
[If prompts were there they should be also reminding people of their rights to a safe workplace rights to be resourced- treated with respect etc.][4]	4

	**Theme**	**Number of times categorised**
1.	Prompting professional codes - A helpful inclusion	3
2.	Prompting professional codes - An unhelpful inclusion	2
3.	Midwives - Already aware of codes - Not required	1
4.	Prompting professional codes - Alternative approach required	3
5.	Midwives - Should remain professional even in distress	1
6.	Midwives - If needing reminders, should not be working	1
7.	Prompting professional codes - Requires sensitivity	1

4

An online intervention designed to support midwives in work-related psychological distress should prioritise the inclusion of information designed to inform midwives as to where they can access legal help and advice.

4.1 The inclusion of information designed to inform midwives as to where they can access legal help and advice.

Rank value	Option	Count
1	Not a priority	0
2	Low priority	3
3	Somewhat a priority	2
4	Neutral	3
5	Moderate priority	7
6	High priority	17
7	Essential priority	12

**Mean rank**	5.57
**Variance**	2.02
**Standard Deviation**	1.42
**Lower Quartile**	5.0
**Upper Quartile**	7.0

Consensus Achieved = yes (High/Essential Priority) 65.9%

Minimum score = Not a priority 0 (0%)

Maximum score = High Priority 17 (38.6%)

The inclusion of information designed to inform midwives as to where they can access legal help and advice.

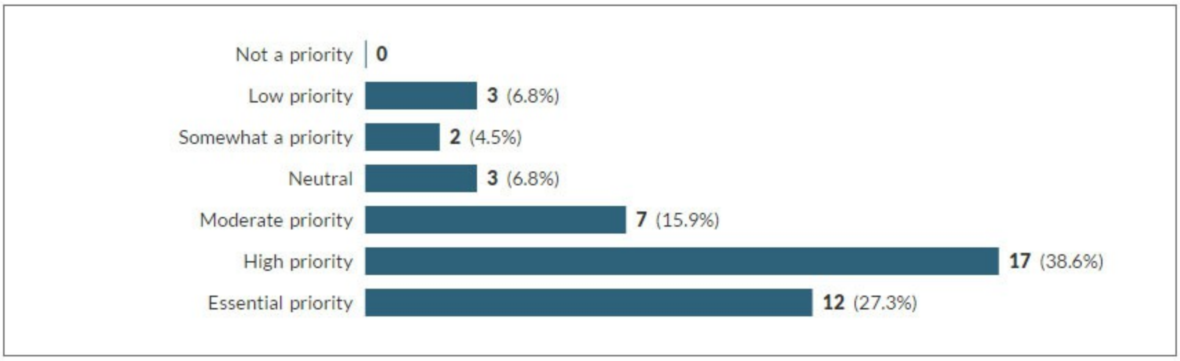

Thematic analysis of open text responses

Why did you choose this rating of priority?

**Comment**	**Themes Assigned to**
Depends what the issue is-legal attention [may help with a dispute][1] but also [elongate conflict][2]-which cannot be in the interest if anyone-so far these questions all raise my concerns about trying to automate what are subtle and difficult decisions in the provision of help to those in psychological distress.	1,2
[Essential to provide that information and links][1] because an [emotionally distressed midwife may not have the presence of mind to even think about such resources][7], [nor know where to access them][7]	1,7,7
[In times of distress they may not think about this avenue][1, 7]	7,1
[This should be part of their professional preparation!][1]	1

if stressed/distressed [need to give positive practical advice which may include lawyers][1]	1
[It's really important this is accessible at the time of need][1]	1
it would be [useful to have recommendations][1] rather than having to source this themselves	1
[I would not think users of your platform would need this][2] ie they [can gain this info elsewhere][2]	2,2
This will [ensure that they are protected][1] and [get additional benefit from the intervention][1].	1,1
This information may or may not be required, but when needed, finding it could be an extra source of worry. The [platform could make a real difference by making this easy][1].	1
[Any help and advice is welcome][1] and [will encourage timely assistance][1]	1,1
[Signposting is an essential priority and means for help][1]	1
[May be needed][1] if they had not recognised the risks beforehand and continued to work	1
[All types of supports available need to be freely available][1, 3]	1,3
[A very good idea][1] so midwives under pressure have access to this information. Perhaps [include alternatives][3] other to the RCM, as some midwives feel the [legal support is not good enough in some instances?][7]	1,3,7
This [would be reminding them to seek guidance][1] from their countries College of Midwives or local support body.	1
The platform may bring us many issues for the midwife and [immediate support could help][1]. Additionally, it [could be quite empowering for the midwife.][1]	1,1
This isn't information that people automatically know off the top of their heads and [can help manage worry].[1]	1
I'm [not sure what this question is asking][4]	4
[providing a one stop shop to get all information in one place][3]	3
This [may be useful][1] depending on the circumstances.	1
Information regarding further advice and legal support I think [would be useful][1]	1
Once the initial crisis is dealt with then will be the time to get legal help and advice. [Getting advise may alleviate some of the psychological distress.][1]	1
This is [essential practical information][1]	1
[better][1]	1
Again [for the benefit of offering support to midwives][1]. [Autonomous practice can be lonely practice][7].	1,7
It is an item that [may be included as part of the general information][3] regarding any amnesty, professional codes of conduct and ethics.	3
A nurse or [midwife who fears for her registration][6] due to workplace stress related issues [would find comfort in this][1].	6,1
[Might be relevant for some][1]	1
again I believe this [could cause additional distress][2] as they may not have even though t that they may need legal support / advice	2
It [should be a specific area not one that is prompted][3]	3

They [may find this information useful][1]	1
Midwives in this situation [often do not know or are incapable of assessing how to put support in place][7]	7

	Theme	Number of times categorised
1.	Information, legal help and advice - A helpful inclusion	29
2.	Information, legal help and advice - An unhelpful inclusion	4
3.	Intervention - A range of options should be made available	5
4.	Question - Meaning unclear	1
5.	Midwives - Should remain professional even in distress (Theme removed following a process of secondary analysis).	0
6.	Midwives - Fear retribution	1
7.	Midwives - Need support	6

Do you have any additional comments you would like to share?

Comment	Themes Assigned to
Many [midwives think it is 'their fault'][5] when they are distressed by work related events or issues; having a reminder about legal help and advice [may help someone take action to address the issues][1], which not only is important to do so, but [in itself can be healing][1]	5,1,1
[Unions & professional guilds usually offer at least preliminary advice][3].	3
is there a link between midwives demonstrating psychological distress because of adverse incidents and therefore they may need legal representation? Is this what the questions are getting at? [I just don't get the point…sorry.][4]	4
no	0
Likely that [there are other sources of assistance beyond legal][3]. [Should be full signposting to relevant agencies and support groups][1].	3,1

	Theme	Number of times categorised
1.	Information, legal help and advice - A helpful inclusion	3
2.	Information, legal help and advice - An unhelpful inclusion (Theme removed following a process of secondary analysis).	0
3.	Information, legal help and advice - Can be found elsewhere	2
4.	Question - Meaning unclear	1
5.	Midwives - Blame themselves	1

An online intervention designed to support midwives in work-related psychological distress should prioritise giving platform users the ability to share extended personal experiences for other platform users to read.

5.1 Giving platform users the ability to share extended personal experiences for other platform users to read.

Rank value	Option	Count
1	Not a priority	0
2	Low priority	6
3	Somewhat a priority	5
4	Neutral	6
5	Moderate priority	10
6	High priority	11
7	Essential priority	6

**Mean rank**	4.75
**Variance**	2.55
**Standard Deviation**	1.6
**Lower Quartile**	3.75
**Upper Quartile**	6.0

Consensus Achieved = No

Minimum score = Not a priority 0 (0%)

Maximum score = High Priority 11 (25%)

Giving platform users the ability to share extended personal experiences for other platform users to read

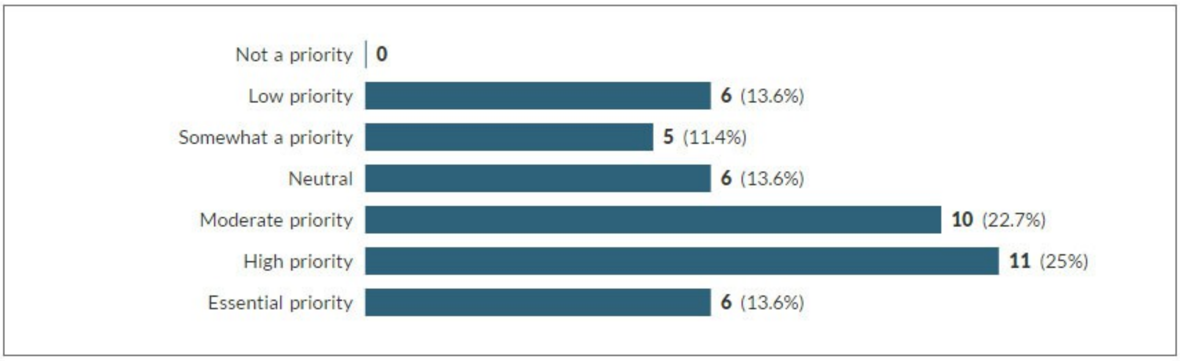

Thematic analysis of open text responses

Why did you choose this rating of priority?

Comment	Themes Assigned to

While sharing stories [can provide support][1] I know of no evidence that just having stories free floating can help without [effective system moderation][3]. I would be concerned that the act of writing would generate an expectation in the writer for a response which may not be forthcoming and so [compound a feeling of aloneness][2].	1,3,2
[sharing can help all users][1] as well as the midwife and [encourage support and assistance][1]	1,1
People's stories [can help each other][1]	1
This would need to be [carefully moderated][3]	3
[The "reality" of scenarios][1]	1
[can be helpful][1] but can also be too long and[potentially identify others][2] especially if smaller unit. Those [others cannot speak for themselves as unaware][2]	1,2,2
Users should be able to [choose to share their information][4]	4
it might be [helpful for others][1] who are feeling isolated in their experience	1
[needs to be done carefully][3] with respect to confidentiality and identifying info shared	3
This [could be really helpful][1] for people in a new distress situation.	1
[May or May not be beneficial][5], if accessing the tool then have already made a choice to seek assistance	5
The [midwife would not feel so isolated][1]	1
[Not all midwives maybe ready to share][4] their concerns or situation when first accessing the online intervention	4
support group is [useful way to reduce stress][1]. Like my 4-year-old says, [sharing is good!][1]	1,1
[Not all are happy to share][4]	4
Sharing experience is [personal choice][4] and [can help others][1]	1,4
Being listened to and feelings/experiences validated is an [essential part of getting support][1].	1
This is a [major key to healing][1]. Sharing stories and being able to talk about the situations in a safe environment is [essential to help each other][1]. Those further down the track of healing [can help those recently wounded][1]. It also [reiterates that this is a difficult job][1] sometimes and some of the things we face as midwives are horrible and [knowing we are not alone is important][1]	1,1,1,1,1
Could be a [good idea][1] to share confidentially, [could have issues if they are identified by friends][3]. Will [need to be moderated][3].	1,3,3
[Sharing helps]1, both the discloser and those who read it and are struggling with their own demons. It [allows them to feel that they are not alone][1] and can [draw strength from others' experiences][1].	1,1,1
because in my experience [peer support is helpful][1].	1
[Do people want to read other peoples experiences?][5] They are usually different.	5
To me that would be the [whole purpose of the forum][1], unless I have misunderstood the project! Of course [confidentiality needs to be maintained][3] but aside from that then yes [to share personal experiences is part of the debriefing process][1] and [may assist others in similar situations][1].	1,3,1,1
I think this [would be helpful][1] to midwife and others reading the account	1

I think that a forum type facility for users to either read or become involved in [would be beneficial][1] for a large majority of users.	1
Sharing the stories [can be invaluable][1] but [not immediately][2] - so less of a priority	1,2
Some ability to do this [would be useful][1] but it would [need to be moderated tightly][3] because it may risk identify the midwife, team members and/or service users which would be unacceptable.	1,3
it [broaden their knowledge][1]	1
sharing [can be helpful][1]	1
Shared experiences [can benefit all][1].	1
While the experience of others may provide some insights into your own situation, [each case is individual and should be managed as such][4]	4
This would be a [useful tool][1], although anonymity and confidentiality must be assured for all parties involved.	1
Reading that someone else has been through the same difficulties that you are experiencing [can help alleviate feelings of isolation][1]. However, the platform cannot be a stress dumping ground as it needs to remain constructive.	1
[Sometimes a form of reflection is needed to allow a person to see a way forward][1]	1
Highly likely that [people will want to tell their stories][1] and others will want to listen. In other fields - e.g. whistle-blowers, patient leaders like James Titcombe, [this has been important][1]	1,1
Context and purpose of the space would need to be set first - Would there be a facilitated response in real time - Extended personal experiences [creates the potential for boundary slip][6] and [potential breach of confidentiality][6] - It [adds the potential for individuals to dominate the space][6]	6,6,6
[Many will not be interested in sharing][2, 4]	2,4
While this [can be a learning opportunity for the future][1] the priority should be on the individual midwife in the present	1

	**Theme**	**Number of times categorised**
1.	Sharing extended personal experiences - A helpful inclusion	39
2.	Sharing extended personal experiences- An unhelpful inclusion	5
3.	Sharing extended personal experiences - Moderation required	7
4.	Sharing extended personal experiences - Should be optional	6
5.	Sharing extended personal experiences - Undecided	2
6.	Sharing extended personal experiences - Risky	3

Do you have any additional comments you would like to share?

**Comment**	**Themes Assigned to**
[Writing is therapeutic for the writer][1]	1

[In Australia you can see the reports of some professional challenges on the registering bodies web site. Not all involve "psychology".][4]	4
I get very little support from friends and family that are not midwives. ALL my support comes from other midwives who have become my trusted friends over time. This is because they actually understand the pressures we work in under and we all generally have a "there but for the grace of God go I" approach. [This kind of connection could be achieved online in a secure forum.][1]	1
This [would have to be monitored][3] by admin staff to ensure confidentiality and appropriate behaviour	3
no	0
There may [need to be moderation][3] of what is made freely available because some comments are not always appropriate or beneficial.	3
[Context important here][4] - lessons can be learned from other on-line support fora	4
could also be resource intensive - [monitoring / responding][3]	3
This may be [difficult to manage][3] as other users experiences may be very emotionally charged and not necessarily accurate. This may [not always be useful for other users][2.]	3,2

	**Theme**	**Number of times categorised**
1.	Sharing extended personal experiences - A helpful inclusion	2
2.	Sharing extended personal experiences- An unhelpful inclusion	1
3.	Sharing extended personal experiences - Moderation required	4
4.	Sharing extended personal experiences - effect may be context dependant	2

6

An online intervention designed to support midwives in work-related psychological distress should prioritise the inclusion of a web based peer to peer discussion chat room.

6.1 The inclusion of a web based peer to peer discussion chat room

Rank value	Option	Count
1	Not a priority	1
2	Low priority	3
3	Somewhat a priority	3
4	Neutral	6
5	Moderate priority	15
6	High priority	13
7	Essential priority	3

**Mean rank**	4.86
**Variance**	1.94
**Standard Deviation**	1.39
**Lower Quartile**	4.0
**Upper Quartile**	6.0

Consensus Achieved = Yes (Moderate/High priority) 63.6%

Minimum score = Not a priority 1 (2.3%)

Maximum score = Moderate priority 15 (34.1%)

The inclusion of a web based peer to peer discussion chat room

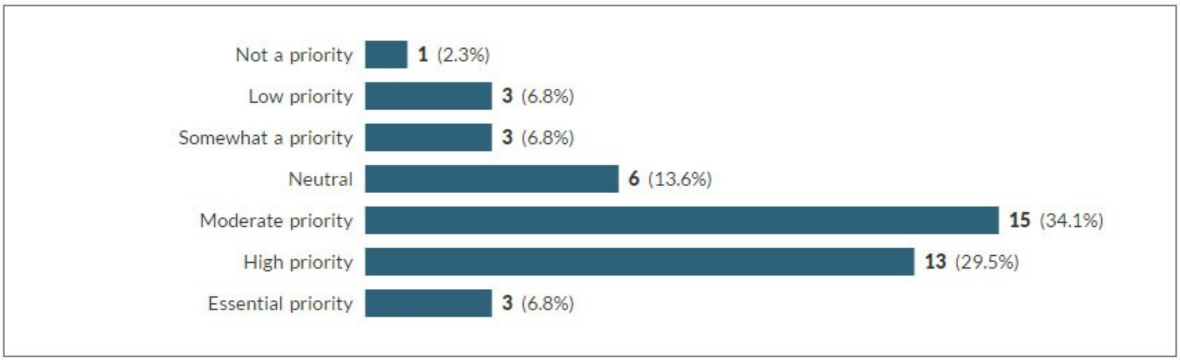

Thematic analysis of open text responses

Why did you choose this rating of priority?

**Comment**	**Themes Assigned to**
[Does the working day allow for this?][4] [How would it be used?][4] Concern that the relative absence of filters that communicating online generates [may lead to unintended harm][2]- would there be other ways of connecting people up that are [less haphazard?][5]	2,4,4,5
Sharing experiences and getting feedback from peers who have experienced similar situations is [very helpful][1]	1
This [may be useful][1] - a form of social networking	1
a [problem shared is a problem Halved?][1]	1
peer support can be both [good][1] and [bad][2]… [can fuel/get things out of proportion][2] and/or can [help gain perspective and practical advice][1]	1,2,1,2

Would be a [good thing to do][1] if funding & supportive [moderation is available][3]	1,3
it [would help the individual][1] to express what they are experiencing in a safe place and [to hear others reactions and opinions][1]	1,1
as above would need to be done carefully with [rules of engagement clearly specified][3]	3
This will [enable professional growth][1] and [networking for support][1]	1,1
[Some may find this helpful][1] but I [don't know that I necessarily would.][2]	1,2
[I would not access a chat room][2]	2
This would be [a real bonus][1] if someone was experiencing problems	1
I think peer-to-peer support [could be helpful][1]	1
This can be a [powerful tool][1] for some staff	1
We need to be aware we are not alone	0
We are all in this profession together	0
Sounds like a [great idea for support][1] in real time. I would suggest the 'ethos' of the chat room, i.e. supportive, being posted as a reminder before every chat starts. Perhaps will be [hard to police in terms of unsupportive comments?][3] But most [chats I think will be very supportive][1] and helpful as long as solid ethos of support is set.	1,3,1
Again, having the ability to share stories [makes people feel they are not alone][1] and will [hopefully aid others to seek help if needed.][1]	1,1
as above (Could be a [good idea][1] to share confidentially, [could have issues if they are identified by friends][5]. [Will need to be moderated][3].) and again [needs to be moderated][3.]	1,5,3,3
[It is important][1] to be able to reach out and support others and share things with them.	1
Because it sounds like [a great idea][1]	1
For some people they [might find it helpful][1] to discuss with other people but [I'm unsure as to its real benefit][4]	1,4
Chat rooms [are more private and can be disabled][1], [reducing the fear][1] of the public accessing the conversations which would not be appropriate.	1,1
I think there is [potential here][5] for [misinformation][2] and creation of further [anxiety][2]	5,2,2
Peer to peer chat for midwives is [beneficial][1] even to those without psychological distress. No one else truly understands the stresses of the role.	1
Many midwives are already doing peer to peer review via electronic means. Setting up another one [would be helpful][1] but maybe not as essential.	1
See comment for question 5 (Some ability to do this [would be useful][1] but it would [need to be moderated tightly][3] because it may [risk][5] identify the midwife, team members and/or service users which would be unacceptable.). I think this relates to this question too.	1,3,5
[yes][1] it will boost their sense of belonging	1

this could be [beneficial][1]- though not the answer for everyone.	1
Look at the success of Tweetchats	0
The notion of peer debriefing is popular and this [would provide that][1] with some anonymity	1
Unsure about this as [anonymity and confidentiality may be breached][5], however speaking to people with similar issues may be [helpful to some][1].	5,1
I like the fact that it's [online and enables a freedom of speech][1] that may not be felt in a face to face situation. Peer chat rooms still leave open the real time interaction which [can lead to things being said without time for moderation and reflection][5]. This [exposes users to unhelpful interactions][2] on the platform.	1,5,2
In my view this is likely to be a [defining characteristic][1] of the intervention, facilitating peer support	1
there are sources of help. I suspect [someone who goes on here would be asking for help from experts rather than peers but I may be wrong][6, 4]	2,4
again [without full context would be difficult to know if this was needed][4]- and [how it would be resourced. moderated et][3, 4]	4,3,4
[many will not be interested][2] in this function -	2
From my experience midwives in the acute phase are very reluctant to go public or even recognise what is happening for them - I also think [this format can lead to mis information][2, 5]	2,5

	**Theme**	**Number of times categorised**
1.	Discussion chat room - A helpful inclusion	33
2.	Discussion chat room- An unhelpful inclusion	11
3.	Discussion chat room - Moderation required	7
4.	Discussion chat room - More information required	6
5.	Discussion chat room - Risky	7

Do you have any additional comments you would like to share?

**Comment**	**Themes Assigned to**
Peers could detect patterns of workplace related stress and see it is the result of certain pressures in the workplace and [take action to address those pressures]1; [research and community action to change those conditions could come from such sharing of experiences][1]	1,1
Perhaps [could screen for any issues to do with self harm/suicide and give help numbers?][4]	4
Online moderated forum [sounds great][1] but would [need real time monitoring][3] 24 hrs a day to ensure appropriate support given and that the service not abused.	1,3
no	0

Raises question of [moderation][3] and rules to prevent misuse, abuse etc.		3
[may be useful][1]. [Difficult to monitor][2,3.]		1,2,3
	**Theme**	**Number of times categorised**
1.	Discussion chat room - A helpful inclusion	4
2.	Discussion chat room- Challenging to facilitate	1
3.	Discussion chat room - Moderation required	3
4.	Discussion chat room - Consider additional features	1

7

An online intervention designed to support midwives in work-related psychological distress should prioritise giving platform users the ability to communicate any work or home based subjects of distress.

7.1 Giving platform users the ability to communicate any work or home based subjects of distress.

Rank value	Option	Count
1	Not a priority	1
2	Low priority	3
3	Somewhat a priority	3
4	Neutral	5
5	Moderate priority	11
6	High priority	10
7	Essential priority	11

Mean rank	5.18
Variance	2.56
Standard Deviation	1.6
Lower Quartile	4.0
Upper Quartile	6.25

Consensus Achieved = No

Minimum score = Not a priority 1 (2.3%)

Maximum score = Moderate/Essential priority 11/11 (25%/25%)

Giving platform users the ability to communicate any work or home based subjects of distress

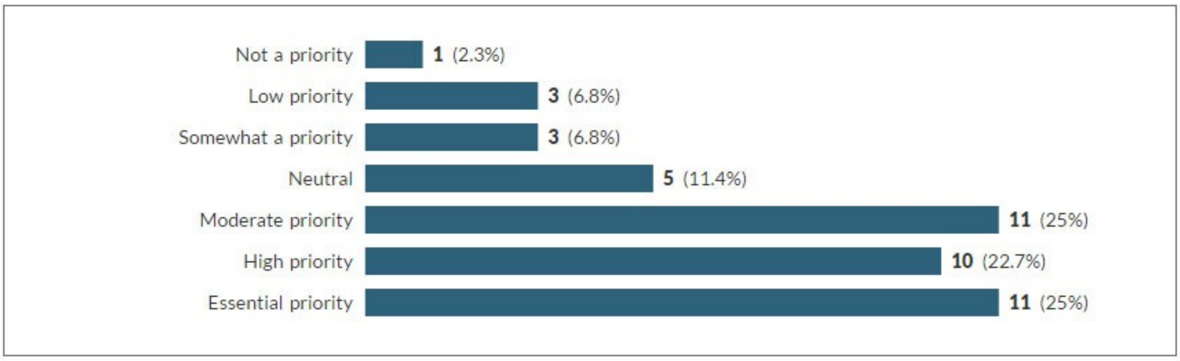

Thematic analysis of open text responses

Why did you choose this rating of priority?

Comment	Themes Assigned to
We don't operate in boxes but [have integrated lives][4]-and support mechanism would need to work with the reality of that.	4
Psychological stress, [no matter its source][3], impacts work and performance	3
[Home based issues will also arise][3]	3
[some folk are not too discriminating!][3]	3
it [is part of bigger picture][1] but [not the reason for the resource][2]	2,1
this is an [essential component][1] in feeling supported	1
Again [rules of engagement][6] would need to be clear as this could degenerate quickly into home related distress, separation, divorce [rather than work related peer support][2]	6,2
Because [these aspects are the source of distress][4]	4
[Home based stress may intensify the situation][2.]	2
Work and personal lives are [entwined][4] and influence wellbeing	4
[Users need to feel that they can talk freely and openly about any cause of distress in their life][1] - people are not [automatons who can separate their work/professional lives][4] although they ultimately have a duty of care to their patients.	1,4
Again [this may be the only opportunity to share their concerns and problems][1]	1
[Unsure re; home based][7]	7
For holistic support, this [sounds very sensible][1].	1
[Home based distress is part of our lives][3] however as this is specific to midwifery I feel it [should be kept at predominantly midwifery related issues][2]. Reality is that [home issues can exaggerate work issues][4].	3,2,4

Lack of time at work and lack of privacy [could be an issue at work][8].	8
[To create a feeling of community][1] and [acknowledge that stresses can come from all different areas of life][1].	1,1
not sure about the home based subjects of distress, [would prefer it if only work based distress][2]	2
[Communication is key to asking for help and support][1]	1
[Work and home stress often come together][4] and affect each other, therefore [to isolate one or the other would be perhaps difficult]3.	4,3
Although midwives may be experiencing stress at home, [I think it should be used mainly for work related stress][2], otherwise the subject could become diluted.	2
[Work and home life are intertwined][4] so it is [important to be able to deal with either][1].	4,1
Midwives in distress [need to be able to communicate their distress][1] as long as no names/places mentioned.	1
is just to [express their feelings][1]	1
it is [hard to separate][3] out the sources of stress- work based from home- the end [results of stress are the same and impact on work][4].	3,4
[The context that we live and work in is salient][10]	10
Frequently when any [psychological distress occurs it does not do so in isolation][4], therefore, for [completeness the intervention has to have a holistic view][1]	1,4
[May be of use][1], however [frustrations may arise][2] at not having immediate or helpful answers/advice/solutions to such problems. [Concerns would also be evident if platform users disclosed domestic or other forms of abuse][6].	2,1,6
[One can impact on the other very easily][4]. [Someone suffering undue workplace stress will be affected at home, and vice versa][4].	4,4
[This platform is for work stress][2] but [home stressors can impinge on work and visa versa][4]	2,4
Participants [need to be able to describe how they feel][1] and [likely that several factors will be combining for them][4].	1,4
[issues at home maybe causing the work related stress][4]	4
[Potential blurring of boundaries][3] and [potential for breach of confidentiality][5]	5,3
I would [prioritize work based distress][2]	2
There [has to be a forum for communicating the level of distress on all aspects][1]	1

	**Theme**	**Number of times categorised**
1.	Communicating any work or home based subjects of distress - A helpful inclusion	15
2.	Communicating any work or home based subjects of distress - An unhelpful inclusion	9
3.	Communicating any work or home based subjects of distress -Inevitable	7

4.	Communicating any work or home based subjects of distress - Both subjects intertwined	14
5.	Communicating any work or home based subjects of distress - Risk of breaching confidentiality	1
6.	Communicating any work or home based subjects of distress - Requires moderation	2
7.	Communicating any work or home based subjects of distress - Undecided	1
8.	Communicating any work or home based subjects of distress - Difficult to engage whilst at work	1

Do you have any additional comments you would like to share?

**Comment**	**Themes Assigned to**
Gender based violence or coercive relationships may be the root cause or [contributory to stress at work][2].	2
[Maybe links to other support forums][3] could be added to the platform i.e. Relate, CAB, MkAct etc	3
no	0
The platform still [needs to be delivered from a workplace][1] perspective, [acknowledging the impact of home life][2].	1,2

	**Theme**	**Number of times categorised**
1.	Communicating any work or home based subjects of distress - discussions need to remain workplace based	1
2.	Communicating any work or home based subjects of distress - Both subjects intertwined	2
3.	Communicating any work or home based subjects of distress - Consider links to outside agencies	1

8

An online intervention designed to support midwives in work-related psychological distress should prioritise an interface which does not resemble NHS, employer or other generic healthcare platforms.

8.1 An interface which does not resemble NHS, employer or other generic healthcare platforms.

Rank value	Option	Count
1	Not a priority	1
2	Low priority	1
3	Somewhat a priority	3
4	Neutral	11
5	Moderate priority	7
6	High priority	8
7	Essential priority	13

Mean rank	5.23
Variance	2.4
Standard Deviation	1.55
Lower Quartile	4.0
Upper Quartile	7.0

Consensus Achieved = No

Minimum score = Not a priority 1 (2.3%)

Maximum score = Essential priority 13 (29.5%)

An interface which does not resemble NHS, employer or other generic healthcare platforms

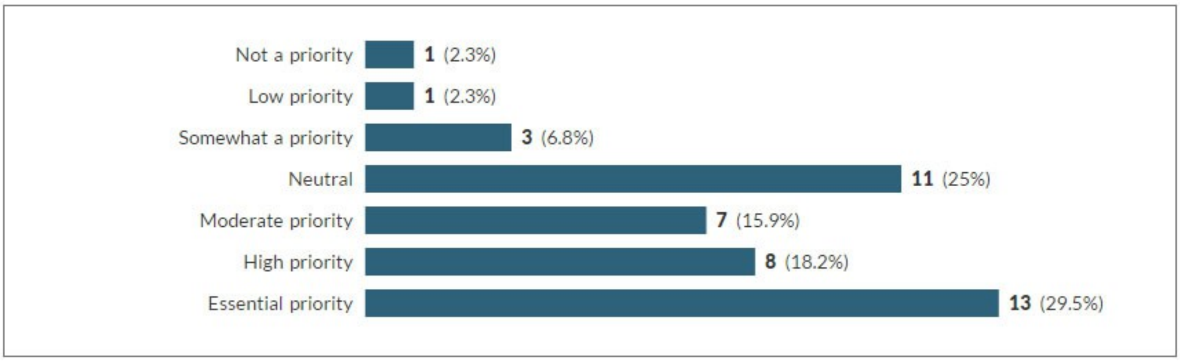

Thematic analysis of open text responses

Why did you choose this rating of priority?

Comment	Themes Assigned to
[I have strong opinions in both directions][5]-it [needs to feel legitimate][4] and to feel safe-this item appears to be operating from the idea that looking different means it will be seen as safe-but it is more complicated than this-need to consider the impact of something looking that [looks authorised] [4] and giving permission to self care as potentially beneficial	5,4,4
[Needs to be independent][1] as if it is perceived as healthcare organisation related, [may inhibit sharing experiences through fear of being identified][6]	1,6
[I am not sure] [5]	5
It [wont be used if it could be viewed as quasi-governmental][1, 6].	1,6

I can see how some would [mistrust a similar platform] 6 and [suspect their views were being monitored by their organisation][6]	6,6
there [are pros & cons][5] to this - it needs to be [professional & credible to be used][4].	5,4
it [needs to appear neutral][1] - otherwise it [may create barriers to communication][6]	1,6
just [needs to look professional][4] and [be user friendly][3]	4,3
I would be much more comfortable with [a neutral interface][1].	1
It needs to be [professional][4] [easy][3] and [secure][3]. I would say [ease is the highest priority[]3]	4,3,3,3
To be seen as a safe and confidential space, I think [it's important to distinguish as separate from their employer][1], the NHS, or their professional body.	1
[Non biased approach is better][1]	1
I think [midwives will be reluctant to share information if support site appears to be NHS based][6], as for most midwives this is their employer. If NHS logo/interface is used, careful reminders of amnesty/support would be a good idea, [otherwise many midwives won't open up][6], in my opinion.	6,6
This [needs to be completely separate][1] and not confused with any health platform as it may trigger symptoms in people who are vulnerable.	1
It would [need to look professional][4].	4
The people accessing this service are likely to have existing troubled relationships with their workplace. They [need to feel that this is a completely separate, safe space][1]. [A clean break][1].	1,1
[this would give the perception that it was completely safe and away from the workplace][1]. Many midwives suffer distress from bullying in the workplace so to create an online safe environment it would make sense if the space [did not look anything like an NHS online place][1]	1,1
As long as it is [user friendly][3]	3
To reduce [concerns about bringing the profession into disrepute][2].	2
Any resemblance to NHS etc. [could deter people from using the platform][6], however, I do feel it [needs to resemble a clean professional image][4].	6,4
It [should be midwife specific, independent, non-judgemental][1]	1
[Yes really important for this to be a different place][1] - not like work	1
[is bad][1]	1
[An opportunity to be creative and forward thinking][6]	6
[This would assist in promoting it as a stand alone or separate service that is confidential and can be trusted][4, 1]	1,4
Sometimes, being part of an unwieldy structure, such as the NHS, is part of the problem. [Resemblance of the same will be detrimental.][1]	1
[To encourage use][1, 6].	1,6
[Perception of impartiality and neutrality likely to be important][1].	1

Many [Midwives may worry about security of data and linkage to an NHS platform may dissuade them from using the site][1, 6]		1,6
an [inviting interface might be encouraging][3]		3
The midwife needs to feel this is a confidential non judgemental [format quite separate from her professional bodies][1]		1
	**Theme**	**Number of times categorised**
1.	Online intervention interface - Should not resemble NHS, employer or other generic healthcare platforms	20
2.	Midwives - Fear bringing the profession into disrepute	1
3.	Online intervention interface - Should prioritise usability	6
4.	Online intervention interface - Should look professional	8
5.	Conflicted opinion	3
6.	Midwives - May not engage if they fear organisational involvement	11

Do you have any additional comments you would like to share?

**Comment**		**Themes Assigned to**
[External to employer is key][1]		1
[A safe haven][2]		2
no		0
The [interface needs to appear to be for midwives only][3]		3
[Perceptions of NHS and other 'brands' should be researched with potential participants][4]		4
	**Theme**	**Number of times categorised**
1.	Online intervention interface - Should not resemble NHS, employer or other generic healthcare platforms	1
2.	Intervention - Should be a safe haven	1
3.	Intervention - Must appear to be for midwives only	1
4.	Online intervention interface - Options should be researched	1

An online intervention designed to support midwives in work-related psychological distress should prioritise a simple, anonymised email login procedure which allows for continued contact and reminders which may prompt further platform usage.

9.1 A simple, anonymised email login procedure which allows for continued contact and reminders which may prompt further platform usage.

Rank value	Option	Count
1	Not a priority	0
2	Low priority	0
3	Somewhat a priority	5
4	Neutral	9
5	Moderate priority	6
6	High priority	14
7	Essential priority	10

Mean rank	5.34
Variance	1.77
Standard Deviation	1.33
Lower Quartile	4.0
Upper Quartile	6.0

Consensus Achieved = No

Minimum score = Not a priority/Low Priority 0 (0%)

Maximum score = High priority 14 (31.8%)

A simple, anonymised email login procedure which allows for continued contact and reminders which may prompt further platform usage

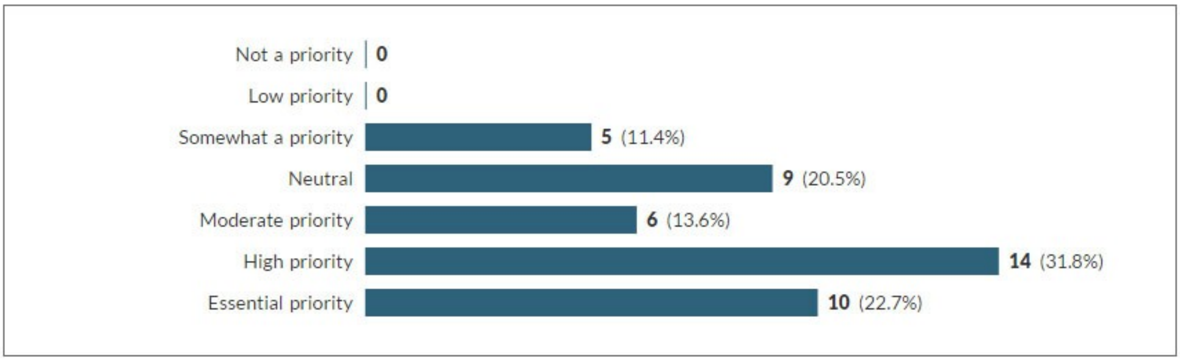

Thematic analysis of open text responses

Why did you choose this rating of priority?

Comment	Themes Assigned to

[Needs to be easy to use] [7]-but do need to consider the privacy question -how it could be accessed during the working day and what might make people feel uncomfortable. Also concerned that sending lots of prompts could encourage the 'always on' issue and so [undermine the very help it is trying to provide][2].	7,2
[Needs to be easy]7 and [anonymous][6]	7,6
[maybe cannot be anonymous?][3]	3
[no brainer][1].	1
[anonymised email helpful][1] and in extreme cases [could be located to identify individual ie potential for grievous harm etc.][5]	1,5
Keeping [access simple][7] is essential yet [security is also key][8]	7,8
[important in terms of being user friendly][1]	1
[this could be useful][1]	1
The platform must be [easy to use and feel inviting][7].	7
It [needs to be as simple as possible][7] - I run an online education product, and even if you have very intelligent people on your hands, online they become very impatient and find it difficult to use a complex entry form. Also, many midwives are from an older generation, some of which will really [benefit from things being simple online][7].	7,7
Encourages [easy access][1] and [engagement][1]	1,1
[Receiving e-mails could be an issue with confidentiality][3, 8].	3,8
[Makes sense.][1]	1
[Easy access][1]	1
[Ease of use][7], and [anonymity is essential][6] in my opinion.	7,6
[Makes it easy to log in][1]	1
I think this [should be completely optional][9]… [some users may only want one-way contact and fear that their use would be discoverable.][2]	9,2
Would [need to be a choice or option][9] for this to occur. [Some midwives may not want continued contact][2] while [others may value it][1].	9,1,2
I am [unsure about this][4]. [Yes to anonymised email login][1] but I [unsure about reminders and prompts][4].	4,4,1
it boot care continuity	0
again [for support and connection][1]	1
While a degree of [anonymity may be beneficial][1], there [may be a time where a situation escalates to one of self harm and there is a want to intervene][5]	1,5
This [facility is essential][1] due to the sensitive nature of the platform	1
To [facilitate useful engagement][1], even when the user feels like withdrawing from the platform due to their stress level.	1
Apps, [quick log in facilities all encourage use][1].	1
[Important for the intervention to have contact details][5], even if participants appear anonymously	5
[Easy access is the key][1, 7]- [some MW may not want their email address logged][2]	1,7,2
[to increase usability][1]	1
Midwives may be incapable of putting in follow up if they are very distressed	0

	**Theme**	**Number of times**

		categorised
1.	Anonymised email login procedure - A helpful inclusion	18
2.	Anonymised email login procedure - An unhelpful inclusion	4
3.	Anonymised email login procedure - Anonymity may not be possible	2
4.	Undecided	2
5.	Anonymised email login procedure - Can be used to intervene	3
6.	Anonymity - Essential	2
7.	Anonymised email login procedure - Ease of use a priority	8
8.	Anonymised email login procedure - Security a priority	2
9.	Anonymised email login procedure - Should be optional	2

Do you have any additional comments you would like to share?

Comment	Themes Assigned to
no	0
[May be safeguarding issues in being able to identify participants][1]. Plus [full anonymity may pose risks to participants][1] - [intervention could be used/ abused by people posing as midwives][1].	1,1,1
[Anonymity could provide a very supportive platform.][2]	2

	Theme	Number of times categorised
1.	Anonymised email login procedure - Risky	3
2.	Anonymity - A helpful inclusion	1

10

An online intervention designed to support midwives in work-related psychological distress should prioritise an automated moderating system where ‘key words’ would automatically initiate a moderated response.

10.1 An automated moderating system where ‘key words’ would automatically initiate a moderated response

Rank value	Option	Count
1	Not a priority	3
2	Low priority	2
3	Somewhat a priority	3
4	Neutral	13
5	Moderate priority	12
6	High priority	8
7	Essential priority	3

Mean rank	4.48
Variance	2.25
Standard Deviation	1.5
Lower Quartile	4.0
Upper Quartile	5.25

Consensus Achieved = No

Minimum score = Low Priority 2 (4.5%)

Maximum score = Neutral 13 (29.5%)

An automated moderating system where ‘key words’ would automatically initiate a moderated response

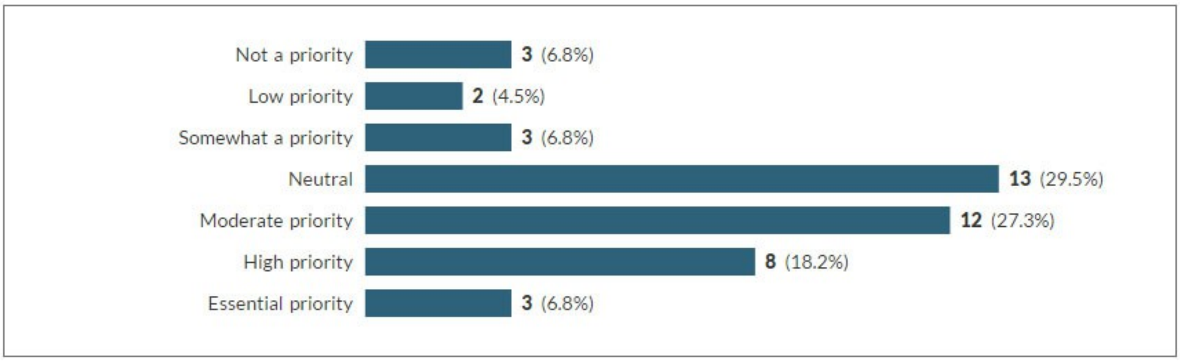

Thematic analysis of open text responses

Why did you choose this rating of priority?

Comment	Themes Assigned to
Feels scary to me to think that certain words would drive a technological knee jerk reaction-if you get my drift.... Also it [signals control is not in the users hands][2] -some research indicating that companies who appear to know more about a 'customer' than the 'customer' knows about the entity behind the IT interface [leads to suspicion and mistrust] [2….so] tricky if we are expecting someone to use this via feeling distressed.	2,2,2
[Useful][1] as certain types of experience can be responded to in a generic way	1
[I do not feel this would be supportive at all][2]	2
[good idea][1]	1
[not sure what is implied here][3]	3
would [make best use of resources][1]	1

[to prevent offensive words and spam messages being posted to the community][1]	1
[You would need to be careful that you didn't have the Microsoft word annoying paperclip pop up!][7] [The midwife might be talking about a mother expressing suicidal thoughts!!!!][7]	7,7
[Don't know enough about this to answer fully][3]. [It might work well or be a little impersonal].[4]	3,4
[May make more refined and user friendly][1]	1
This [suggests that it is robotic and not a personal response][2].	2
[Not sure if this would be perceived as surveillance][2] but [could be helpful][1]. [Tricky one!][4]	2,1,4
As mentioned before, [for suicide/self harm support phone numbers][1]. Would [have to be phrased very carefully to make sure it comes across as supportive, rather than 'lecturing'.][7]	1,7
[Safety feature][1] and [necessary][1]	1,1
[Could be a good idea][1], [could also be annoying!][2]	1,2
This [needs to be a safe space][6] and trolling could utterly destroy that.	6
[This might be more important but difficult for me to imagine what this actually is][3, 4]...again, the [question for me is not that clear.][3]	3,4,3
[Possibly necessary][1] to filter something like swearing or naming NHS trusts etc.	1
[Could be helpful][1]	1
I understand the idea behind this but [don't think that this is always going to be appropriate][2, 7]. [Automated responses do not exude care and compassion which would be needed in some circumstances][2].	2
[Uncertain what this would look like or if it is needed][3, 4.]	3,4
I can see how this [might be helpful][1] but I think [moderated is the important work here][5].	1,5
yes [o]	0
[Moderation and facilitation very important][5]	5
A 'one-size-fits-all' is [not appropriate][2, 7] because some peoples distress may be escalating, as identified by 'key words', and therefore a [moderated response is more appropriate][5] and may better facilitate the best follow-up	2,7,5
[Don't understand the concept of the question][3].	3
This [would have to be at an extreme level though][1], [with trigger words such as suicide leading to a moderator intervention.][7, 8]	1,7,8
In [cases where risk or harm to any party is identified][1,] or [where someone may need extra support][1]	1,1
[Not sure if this would work][4]. [Volunteer moderators likely to target better and give more appropriate responses.][8]	4,8
[Not really sure what is meant by this question][3]	3
[Not really sure what this suggests][3]	3
As I have encountered midwives who were potentially suicidal [I feel there needs to be. Safety net system in place][7]	7

	**Theme**	**Number of times categorised**
1.	An automated moderating system - A helpful inclusion	16
2.	An automated moderating system - An unhelpful inclusion	9
3.	An automated moderating system - Meaning unclear	8
4.	Undecided	5
5.	Moderation - Should be a priority	3

6.	Intervention - Must be a safe space	1
7.	An automated moderating system - Must be appropriate	6
8.	Moderation - Should be a human response	2

Do you have any additional comments you would like to share?

**Comment**		**Themes Assigned to**
no		0
Also [needs facility for any participant to flag concern][1] about a post - might be important in safeguarding		1
	**Theme**	**Number of times categorised**
1.	An automated moderating system - Should allow users to flag concerns	1

11

An online intervention designed to support midwives in work-related psychological distress should prioritise an interface which resembles and works in a similar way to current popular and fast pace social media channels: e.g. Facebook

11.1 An interface which resembles and works in a similar way to current and fast pace popular social media channels: e.g. Facebook

Rank value	Option	Count
1	Not a priority	0
2	Low priority	3
3	Somewhat a priority	3
4	Neutral	12
5	Moderate priority	11
6	High priority	11
7	Essential priority	4

Mean rank	4.82
Variance	1.74
Standard Deviation	1.32
Lower Quartile	4.0
Upper Quartile	6.0

Consensus Achieved = No

Minimum score = Not a Priority 0 (0%)

Maximum score = Neutral 12 (27.3%)

An interface which resembles and works in a similar way to current and fast pace popular social media channels: e.g. Facebook

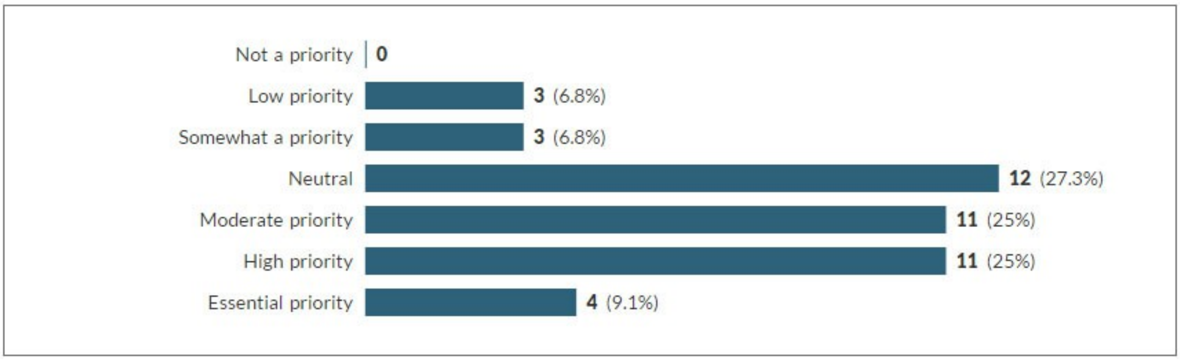

Thematic analysis of open text responses

Why did you choose this rating of priority?

Comment	Themes Assigned to
[This puts the attention control in the users hands][1]	1
[User friendly and familiar to most midwives][1]	1
[people are used to this interface][1]	1
[Not sure that's the model][4]	4
they [could similarly fear views being aired on social media] [2] but the [format is user friendly][1]	2,1
May [aim accessibility][3]	3
[for user friendliness][1]	1
[not necessarily important][2]	2
I am [not a Facebook/social media fan][2] but I know that [others are.][1]	1,2
[There are different reasons for accessing this tool than social media such as Facebook][2]	2
it is the [ease of use which is essential][3] [not its similarity with other media as not everyone would be as familiar][2]	3,2
[Not all are familiar with those media channels][2]	2
[Popular modem][1]	1
Facebook is easy to use and many midwives use it, so [easy transition for midwives wanting support.][1]	1
[Easy to use and connect with][1]	1
[This could work][1] and [relate well with the younger generation.][1]	1,1
It sounds like [quite a good idea][1]	1
Facebook has pros and cons… the thought of a similar look I am [not sure][4] is the way forwards…though [FB def. better than twitter!][1]	4,1
[Needs to be independent of to avoid confusion][2]	2
[It is a tried and tested well used, well loved platform for quick and easy communication.][1]	1
[Not sure][4] this would add value as content so different	4

[Most people are familiar with this type of interface][1]. It needs to be clean and [simple to use][3] to make it as easy as possible for the user.	1,3
Not all midwives use social media so [not certain][4] that this is necessary. Also there are many forms of social media and how do you choose which one to use. [Some may have a distrust of social media][2].	4,2
A system design that is familiar, [easy to use and user-friendly is required][3] - [so yes].[1]	1,3
I do not use Facebook but its [design looks user friendly][1]	1
This would reflect the modern means of communication and therefore [be 'approachable'][1].	1
[No all users may be familiar with social media][2], however a [user friendly platform is essential][3].	3,2
[Ease of use][3] and familiarity for most users will encourage engagement. However, I would not wish it to be as fast paced as Twitter, for example, as threads get lost and interactions are historical within hours.	3
[Not necessarily FB][2, 4] but a [user friendly format is essential][3]	2,4,3
[Interventions that are intuitive][3] - because familiar - are more likely to be used.	3
it [needs to be easy to use][3]	3
I think there is emerging work on the use of FB as a social tool that would [support a different interface]2. People connect FB with social connection - often have a FB identity linked to their personal lives and families- where FB has been used in education student have struggled to mange their student identity within FB as different from their personal identity with blurring across the 2 - leading to [risk of inappropriate non professional behaviour within the FB environment][2]	2,2
[usability is important][3] but the [platform should be unique][2] and therefore have clear links and identity to being a support platform [to prevent confusion and blending of platforms][2] and purpose	3,2,2
[not sure what format is best, can't recommend][4]	4
[Because]1 [distressed people need ease of usage][3]	1,3

	**Theme**	**Number of times categorised**
1.	An interface which resembles and works in a similar way to current popular and fast pace social media channels - Helpful	19
2.	An interface which resembles and works in a similar way to current popular and fast pace social media channels - unhelpful	14
3.	Usability should be the priority	11
4.	An interface which resembles and works in a similar way to current popular and fast pace social media channels - Undecided	6

Do you have any additional comments you would like to share?

**Comment**	**Themes Assigned to**

[Familiarity would make it more appealing and midwives more likely to engage][1]	1
[I think work and personal Facebook should be kept very separate][3]	3
Consideration would also need to be given to [ensuring that the interface was still user friendly][4] to those who may not be familiar with using the more modern means of communication	4
Popular anonymised discussion fora [may be better exemplars than social media like Facebook][2]	2

	**Theme**	**Number of times categorised**
1.	An interface which resembles and works in a similar way to current popular and fast pace social media channels - Helpful	1
2.	An interface which resembles and works in a similar way to current popular and fast pace social media channels - unhelpful	1
3.	Question - Misunderstood	1
4.	Usability should be the priority	1

12

An online intervention designed to support midwives in work-related psychological distress should prioritise the inclusion of midwives from around the world

12.1 The inclusion of midwives from around the world

Rank value	Option	Count
1	Not a priority	3
2	Low priority	7
3	Somewhat a priority	2
4	Neutral	7
5	Moderate priority	11
6	High priority	7
7	Essential priority	7

Mean rank	4.48
Variance	3.39
Standard Deviation	1.84
Lower Quartile	3.0
Upper Quartile	6.0

Consensus Achieved = No

Minimum score = Not a Priority 3 (6.8%)

Maximum score = Moderate priority 11 (25%)

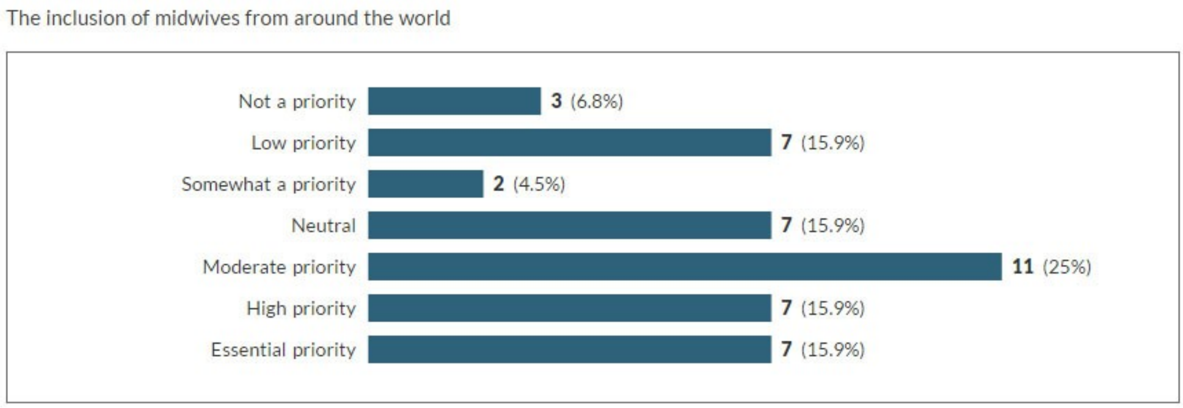

Thematic analysis of open text responses

Why did you choose this rating of priority?

Comment	Themes Assigned to
[Don't have a point of view][3]	3
The issues are similar all over the world; [sharing with international colleagues would help]1 people realise they are not alone	1
[not always possible really][4]	4
its [always useful][1] to review how other cultures might see the problem.	1
I think to be meaningful it [has to begin small][2] and expand…difficult to get context if in other countries	2
[Would be valuable][1] to be global	1
[could be useful][1] but [UK midwives less likely to be able to relate][2] to situations that non UK midwives are experiencing	1,2
Needs to be done carefully as [different countries have different laws etc.][4]. If you did this then the way it looked and the reminders about professional conduct and codes would be even less important	4
The platform [should be open to all midwives.][1]	1
I have [no preference][3] to including midwives from around the world	3
[What happens around the globe is very different][2] in terms of work-related distress and remember not all cultures accept psychological distress	2
[problems n systems are different][2, 4] in different parts	2,4
[Undecided about this][3] as there may be very context specific issues which affect midwives from different countries…or not	3
different working conditions and circumstances [may not translate][2] when discussing the effects of the role	2
[Global village gives perspectives][1]	1
[Yes - good idea][1], but perhaps [concentrate on getting support for UK midwives right first][2], as midwives from different countries may have very different practice styles/formats and therefore [different needs][2, 4].	1,2,2,4

[Midwives no matter where they work, come across similar issues causes psychological distress][1]	1
Access to support is limited overseas. [You may need to consider the format (i.e. videos as slow internet overseas is an issue)][4]	4
That's really interesting! I would imagine that it [might make things more difficult][4] to support each other with in that you don't know how the disciplinary/ legal services function in different countries but it could be [a brilliant platform for learning and reaching out.][1]	4,1
[UK based midwives have different issues to MWs in France, Africa, USA] [2]	2
By providing a tool for all [we can support one another][1]	1
[We can learn a lot from each other.][1]	1
Think it [should be UK only][2] as practice varies so much	2
[I wouldn't necessarily rate this either way][3], hence 'Neutral'. it [should be available for all to use][1] if the want it but I wouldn't say it is essential.	3,1
[We can learn from each other][1] - and [all midwives need to be able to access this][1] as it is a worldwide issue. We are an international midwifery family - and facing similar issues no matter where we work.	1,1
[Lovely idea][1] but the role, systems and culture of midwives work is [not the same in every country]2 and for this reason it would be [more appropriate for different countries to adopt their own systems.][2]	1,2,2
[Global insights very valuable][1]	1
While the role of a midwife may be similar around the world, there are many cultural differences that are [better addressed by cultural specific programs][2]	2
It is [more important to assist health professionals in this country in the first instance.][2]	2
[Users from different countries may have specific requirements of the platform][2]. Plus, [legal issues will vary from country to country and will become a minefield.][4]	2,4
On reflection, this is [probably a good idea][1] but it [generates additional complexities e.g. over safeguarding][4]	1,4
once you open these things it will be targeting globally	0
this could be [difficult to achieve][4]	4
[depends on local context and culture][3], [cost, resources, facilitation etc.][4]	3,4
[Not sure][3] if this would be preferable to a format that is appropriate to a specific culture/country	3
Most midwives feel they are the only one and very isolated, [to know it is a global phenomenon would be reassuring][1]	1

	**Theme**	**Number of times categorised**
1.	The inclusion of midwives from around the world - Helpful	18
2.	The inclusion of midwives from around the world - unhelpful	14
3.	The inclusion of midwives from around the world - Undecided	6
4.	The inclusion of midwives from around the world - Challenging to facilitate	10

Do you have any additional comments you would like to share?

Comment	Themes Assigned to
Such a resource is [valuable to all][1] - [other countries may not have the infrastructure/resources to develop such a resource][3]; would be a [good global village health promoting action][1]	1,3,1
[Global perspectives are always valuable][1] and [can offer valuable learning or support opportunities.][1]	1,1
[Happy to affiliate][1] our GlobalVillageMidwives to this online support modem	1
Maybe not so much support as [learning and networking?][1]	1
In developing this platform for a specific group of midwives, a [future goal may be to adapt it][2] for other specific groups once this project is functioning and any difficulties have been eliminated	2
I would suggest developing the Platform for one country and then [enabling it to be adapted if necessary][2] to roll out to other countries. Maybe countries with similar requirements and regulations could be grouped to enable an international interaction.	2

	Theme	Number of times categorised
1.	The inclusion of midwives from around the world - Helpful	6
2.	The inclusion of midwives from around the world - Could be made fit for purpose	2
3.	The inclusion of midwives from around the world - Challenging to facilitate	1

13

An online intervention designed to support midwives in work-related psychological distress should prioritise proactive moderation (i.e, users are able to block unwanted content and online postings are 'pre-approved')

13.1 Proactive moderation (i.e, users are able to block unwanted content and online postings are 'pre approved')

Rank value	Option	Count
1	Not a priority	1
2	Low priority	2
3	Somewhat a priority	2
4	Neutral	3
5	Moderate priority	9
6	High priority	15
7	Essential priority	12

Mean rank	5.5
Variance	2.2
Standard Deviation	1.48
Lower Quartile	5.0
Upper Quartile	7.0

Consensus Achieved = Yes (High/Essential priority) 61.4%

Minimum score = Not a Priority 1 (2.3%)

Maximum score = High priority 15 (34.1%)

Proactive moderation (i.e, users are able to block unwanted content and online postings are 'pre approved')

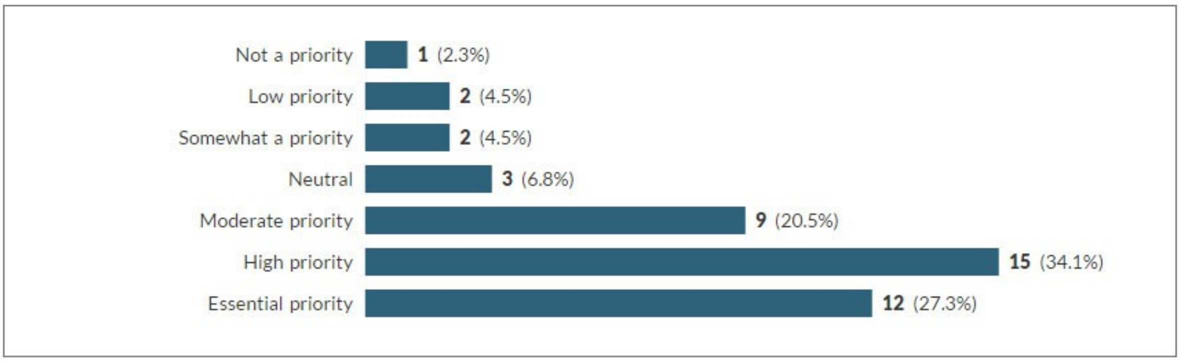

Thematic analysis of open text responses

Why did you choose this rating of priority?

Comment	Themes Assigned to
Careful moderation appears to [increase the positive identity impacts][1] from belonging to a network, particularly when online-but must be noted that careful moderation involves mostly support and responding to what people post-not blocking things-which is likely to be an NHS style- [so being clear about the nature of moderation is important][4]	1,4
[Saves unwanted and inappropriate content being shared][1]; [ensures integrity in the system][1]	1,1
[needs moderation][1]	1
[Probably necessary][1] but a massive job for someone.	1
[within reason][1]… [depends on what is envisaged would be blockable][4]	1,4

Midwives already have a code of practice so this [may not this necessary][2]	2
the [user shouldn't have to be exposed to offensive [5]	5
[pre approval is always a worry][2]. This means the person posting has to wait until the moderator approves the post before she gets help and also [unwanted content is in the eye of the beholder!][4]	4
Platform users are likely to be highly sensitive and [do not want to be accessing material that will not be helpful][4].	4
Ensure the [site is not misused or deviates from purpose][5]	5
[Needs a control centre][5]	5
[I don't really understand what's meant by 'pre approved posting'?][3] But used [should have the ability to moderate somewhat themselves][4], otherwise [trolling and spam may be missed][5].	3,4,5
[This is important][1] to prevent abuse of the intervention and abuse within it	1
[Yes][1], and this would personalise the platform.	1
[Pre approval would slow things down a lot][2] and might make things [feel a little patronising or stressful][2] when you want to reach out to someone immediately who you feel may need that. However, there should be [no space for abusive, cruel or bullying content][5]. That would not make it a safe space. Proactive moderation, [yes][1], [pre approval, no][2, 4].	2,2,5,1,2,4
[No advertising from formula companies for a start][4]...nor Bounty. Ethical considerations v important.	4
[Not necessary][2]	2
[To reduce spam or solicitation from unwanted parties][1].	1
[This is essential][1] so that any illegal activities are identified before being made public and the user incriminates themselves. [A private discussion could then be had between the moderators and the user if necessary][4]. It will [also prevent any unwanted and inappropriate content being posted][1].	1,4,1
[Think it is important][1]	1
This is [common sense to me][1]. It [must be moderated][5] by a person/people [who understand the issues][4]. There is potential for massive breeches in confidentiality etc. so it [has to be moderated][5].	1,5,4,5
[on line forums could be harmful if not "policed"][1, 5]	1,5
I think [good moderation is important for all][1, 5]	1,5
This would [provide a level of control][1] to the user and [allow them to determine some of the content][1].	1,1
[Need more detail as to what is meant by pre-approved][3] to answer question fully.	3
Users [should be able to 'block' other users if they are feeling exposed, i.e. to their supervisors at work][4]. However, I [do not like the idea of moderator approval being required][2] before posts can be made as this [delays the interaction and places a further level of burden on the moderators][2].	4,2,2
Surely that U.S. [A form of censorship?][2]	2
[Important measures in protecting participants][1]. Other online fora have these in place. Lessons to learn from those.	1
it is difficult to answer questions as specific as this when the [proposed intervention and context is undecided][4]	4
[For safety][1]	1
[Need to feel they retain some control][1]	1

	Theme	Number of times categorised
1.	Proactive moderation - Helpful	21
2.	Proactive moderation - Unhelpful	8
3.	Proactive moderation - Meaning unclear	2
4.	Proactive moderation - Must be tailored to suit context	11
5.	Moderation - essential	9

Do you have any additional comments you would like to share?

Comment	Themes Assigned to
Sometimes trolling behaviour or mentally unstable users may cause additional distress [if there was no block button][1] or [previewing of postings][1]	1,1
Moderation of postings [would be recommended][1] to eliminate the occasional 'rant' inappropriate statement that may be posted	1
We must respect that people accessing the Platform will be professionally trained and registered and therefore [should be less likely to post anything inappropriate][3].	3
Might [not need to pre-approve postings][2] - ability for users to flag content to moderators [might be enough][2].	2,2
moderated discussion could be highly resource intensive - it would be easier therefore to answer questions like this in context- I think people may appreciate an on line chat facility which needs to be in real time [rather than pre approved posting][2] with responses as the moment could often be lost by the time the person comes back to the site	2

	Theme	Number of times categorised
1.	Proactive moderation - Helpful	3
2.	Proactive moderation - Unhelpful	3
3.	Midwives - Able to self-moderate	1

14

An online intervention designed to support midwives in work-related psychological distress should prioritise reactive moderation (i.e., users are able to report inappropriate content to a system moderator for removal)

14.1 Prioritise reactive moderation (i.e, users are able to report inappropriate content to a system moderator for removal)

Rank value	Option	Count
1	Not a priority	1
2	Low priority	2
3	Somewhat a priority	2
4	Neutral	2
5	Moderate priority	6
6	High priority	16
7	Essential priority	15

Mean rank	5.68
Variance	2.26
Standard Deviation	1.5
Lower Quartile	5.0
Upper Quartile	7.0

Consensus Achieved = Yes (High/Essential priority) 70.5%

Minimum score = Not a Priority 1 (2.3%)

Maximum score = High priority 16 (36.4%)

Prioritise reactive moderation (i.e, users are able to report inappropriate content to a system moderator for removal)

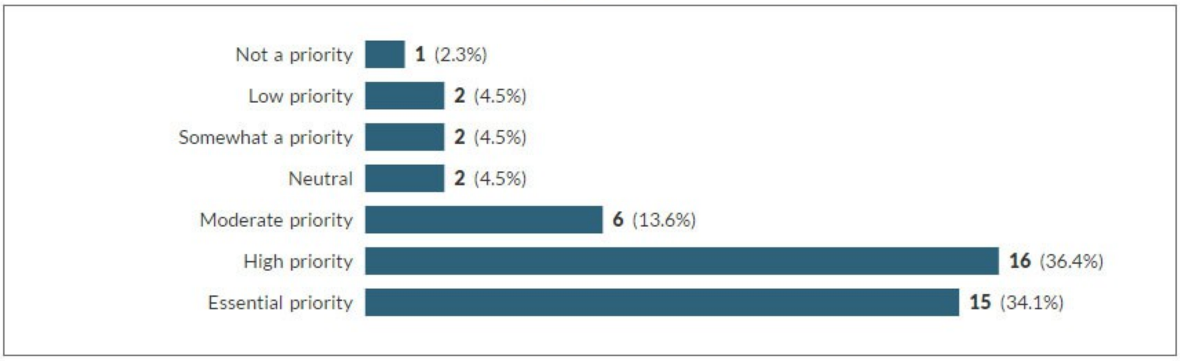

Thematic analysis of open text responses

Why did you choose this rating of priority?

Comment	Themes Assigned to
The issue of blocking is an important concern-symbolic impact-and issues around authority and also online hazing. These question for me are not so much about priority but [represent serious design challenges][3] for the provision of psychologically savvy online support-worth checking out the recent research about online CBT and its limitations	3
[Essential][1] to eliminate trolling or inappropriate use of system	1
an [important aspect][1]	1
its hard to imagine why users would do this [but][1]….	1

if breech of confidentiality [yes][1]	1
as above (the [user shouldn't have to be exposed to offensive postings][1])	1
if you had details rules of engagement then I suppose this [might work][1] but again inappropriate is [in the eye of the beholder][3] so this would need to be done carefully	1,3
[Feeling unable to report inappropriate material adds to a low sense of self esteem][1].	1
Yes, I think there [needs to be some recourse for reporting inappropriate content][1]	1
[Some are unable to distinguish between what is appropriate and what is not][3]	3
This [ensures professionalism][1] and [quality control /standards][1]	1,1
I think having a few moderators with increased power to remove posts etc. is a [good idea][1] as in my experience running a group of about 7000 users, it [works well in keeping posts supportive][1].	1,1
[Moderators may not see everything][3] and there [needs to be a way to remove posts if they are inappropriate][1]	3,1
This is an [essential feature][1]. Users will be in an emotionally vulnerable state and [need a robust form of protection from trolls and bullies][1].	1,1
[I don't feel that it is necessary][2] [but Maybe][1] users would post inappropriately in the heat of the moment when looking for advice	2,1
This [works well][1] on other forums.	1
I think proactive (Q13) is [better than reactive moderation][2]. Sometimes things are posted and it could be hours/days before it is removed and potential [damage is already done][2].	2,2
Would [prefer to have a proactive approach][2] rather than reactive.	2
[Yes this is needed][1] in addition to proactive moderation	1
Some [comments may be inappropriate and cause distress][1], making the user feel powerless and perhaps unwilling to continue.	1
[Extremely important][1] to reduce additional stress.	1
This [would provide a safety net][1] which protects against not having pre-moderation for posts, as per question 14.	1
[Definitely][1]. [Important protective feature]1. [Needs clear and transparent criteria][3]	1,1,3
[If the site doesn't work for the individual it will not be used][3]	3

	**Theme**	**Number of times categorised**
1.	Reactive moderation - Helpful	23
2.	Reactive moderation - Unhelpful	4
3.	Reactive moderation - Design challenges	6

Do you have any additional comments you would like to share?

Comment	Themes Assigned to
same as previous question (moderated discussion could be [highly resource intensive][3] - it would be easier therefore to answer questions like this in context- I think people may appreciate an on line chat facility which [needs to be in real time][1] rather than pre approved posting with responses as the [moment could often be lost by the time the person comes back to the site][2])	3,1,2

	Theme	Number of times categorised
1.	Reactive moderation - Helpful	1
2.	Reactive moderation - Unhelpful	1
3.	Reactive moderation - Design challenges	1

15

An online intervention designed to support midwives in work-related psychological distress should prioritise 24/7 availability of the platform

15.1 24/7 availability of the platform

Rank value	Option	Count
1	Not a priority	0
2	Low priority	0
3	Somewhat a priority	2
4	Neutral	1
5	Moderate priority	4
6	High priority	12
7	Essential priority	25

Mean rank	6.3
Variance	1.07
Standard Deviation	1.04
Lower Quartile	6.0
Upper Quartile	7.0

Consensus Achieved = Yes (High/Essential priority) 84.1%

Minimum score = Not a Priority/Low priority 0 (0%)

Maximum score = Essential priority 25 (56.8%)

24/7 availability of the platform

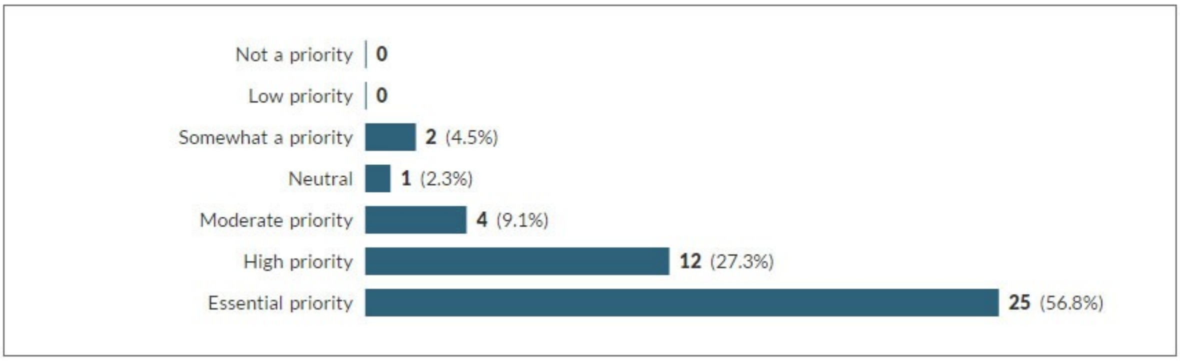

Thematic analysis of open text responses

Why did you choose this rating of priority?

**Comment**	**Themes Assigned to**
[I don't know enough][2] about the working patterns of midwives to comment	2
Many midwives work night duty; distress often keeps people awake at night; having a resource to communicate with 24/7 [may be life saving][1]	1
24-hour access is [essential][1]	1
[well if its global?][1]	1
they [may seize the moment if 24/7][1] and [otherwise not bother once crisis has past][1]	1,1
[so that there is support whatever the time of day or night][1]	1
Why would it close??!! Especially if you are planning to make it open internationally it [needs to be available 24/7][1]	1
Insomnia and night time worry are common and the platform needs to reflect awareness of this and [be available][1] to support.	1
We work across the 24hr period, [would be good][1] to access service when needed and not have to wait until shift pattern allows convenient action	1
with midwives working 24/7 this is [essential][1]	1
[many problems are worse in the hours of darkness][1]	1
Our work involves 24/7 care [We need to self care 24/7][1]	1
[Care given by midwives is 24/7 so a support tool would have to be also.][1]	1
Time zones obviously vary and if it is a global intervention it [needs to be available 24/7][1]	1
[Shift work!][1]	1
Because when you are psychologically distressed, sleep may not come easily and [the time when you need support the most may be at a particularly unsociable hour][1].	1
It is [imperative][1] to be accessible	1

It is [essential that it is 24/7!][1] Slightly [useless otherwise][1] as midwives like the general public access online mediums all times of the day, plus by the inclusion of international midwives it would [need to be to allow for time differences][1.]	1,1,1
[People expect 24/7 availability][1] of any websites	1
This is [essential][1] for users who work in healthcare due to the 24hr service. 3am in the middle of a night shift can be a lonely place if you are needing support!	1
If it was available internationally to all midwives who live in different time zones it would be more [valuable to have it available 24/7][1]	1
This would be [ideal.][1]	1
this is [crucial][1] if staff can access support when they need it	1
[We work all hours, 24/7][1], public holidays too	1
[Midwives provide 24-hour service; they need the same][1]. Also, distress needs to be addressed as it arises	1
[Essential][1] due to differing shift patterns and the individuality/unpredictability of when someone may require/seek help	1
[Stress and emotional responses happen 24/7, and waiting for the support Platform to open during certain hours may add to the stress being felt][1].	1
[Needs to be available whenever][1] people want to participate and fit all shift patterns	1
[midwives work 24/7][1] plus [if its going to be global][1]…	1
people need help when they log in and that [can be any time][1] - [particularly important if making worldwide][1]	1
[Needs to be available at all times][1] as the lows come at all times	1

	**Theme**	**Number of times categorised**
1.	24/7 availability - Helpful	35
2.	24/7 availability - Undecided	1

Do you have any additional comments you would like to share?

**Comment**	**Themes Assigned to**
if global [this will be essential][1]	1
the biggest thing [midwives lack is time][3] - over worked stressed midwives feel over whelmed - they often know about the availability of counselling. relaxation strategies, can seek current web based support interventions like those offered on mindfulness web sites - they can have wellness apps on their phones, daily inspirational messages etc. - what they consistently say is there is [no time][3] - there is also the trust issues and [need for confidentiality][2]- so if they have that window of opportunity when they are looking for support [24-hour access should be available][1]	3,3,2,1

	**Theme**	**Number of times**

		categorised
1.	24/7 availability - Helpful	2
2.	Midwives - Need confidentiality	1
3.	Midwives - Have no time	2

16

An online intervention designed to support midwives in work-related psychological distress should prioritise the implementation of an initial simple user assessment using a psychological distress scale to prompt the user to access the most suitable support available

16.1 The implementation of an initial simple user assessment using a psychological distress scale to prompt the user to access the most suitable support available

Rank value	Option	Count
1	Not a priority	1
2	Low priority	2
3	Somewhat a priority	1
4	Neutral	2
5	Moderate priority	14
6	High priority	17
7	Essential priority	7

**Mean rank**	5.39
**Variance**	1.78
**Standard Deviation**	1.34
**Lower Quartile**	5.0
**Upper Quartile**	6.0

Consensus Achieved = Yes (Moderate/High priority) 70.4%

Minimum score = Not a Priority/Somewhat priority 1/1 (2.3%/2.3%)

Maximum score = High priority 25 (38.6%)

The implementation of an initial simple user assessment using a psychological distress scale to prompt the user to access the most suitable support available

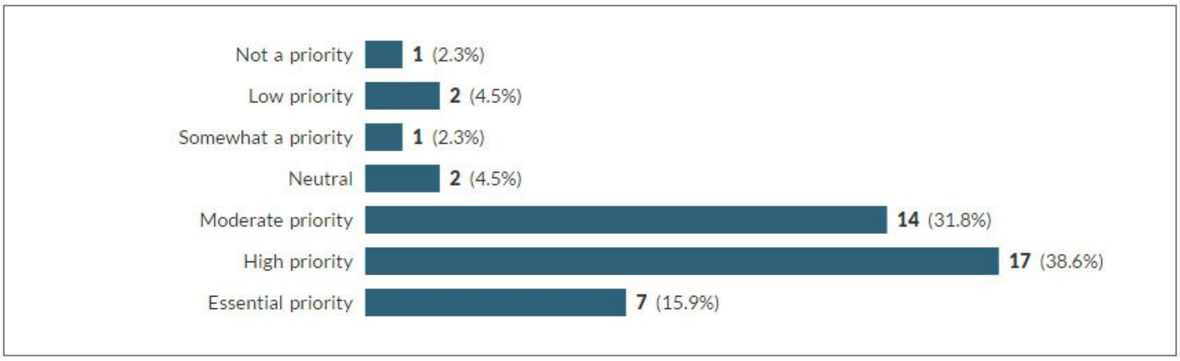

Thematic analysis of open text responses

Why did you choose this rating of priority?

Comment	Themes Assigned to
This is [likely to be helpful][1] -and they are freely available online anyway-but [generally they don't say what help you need but just indicate that help is needed?][3]	1,3
[Enables triage][1]	1
definitely formalizing the input.	
[not sure who benefits by this...who is monitoring?][3] [what is trigger for contact...what if 24/7 use then who man's][3] (e.g. sever distress leaves monitor distressed when later reads post) ... [what is done with individual or accumulated information?][3]	3,3,3
[to enable the user to obtain the most appropriate source of support][1]	1
[Yes this is a good idea][1], but [how often would you administer it though??][3] [Once, on enrolment would be too little, every day on log in too much![3]	1,3,3
Again this [could be impersonal][2], [I am not sure][4]	2,4
It's [good to recognise and advise the appropriate pathway of continued care as quickly as possible][1]	1
If one exists that [would be excellent,][1]	1
I think it's [best to have this as an option][5]	5
[Accessibility is important] [3]	3
[Sounds like strong support.][1]	1
This [could alert moderators to high risk people who need support immediately][1]	1
[Yes - quick and simple][1]	1
A tool as this [can help the user to address their own state properly][1] and [may prompt them to seek help][1] which they hadn't realised that they were a candidate for before.	1,1
seems like [a sensible idea][1]	1
All [directed help is useful][1] as initially you don't know where to start looking for help	1
[High priority][1] as very distressed may need urgent face to face support	1

Tools such as PHQ9 and GAD7 for self assessment [would be a beneficial tool][1] to have online with clear links to external support such as IAPT etc.	1
This [may be able to guide the midwife on who/what may fulfil her needs at the time.][1]	1
This is a [simple safety feature][1] which [could really help to increase midwives' self-awareness][1] and [signpost them to appropriate support.][1]	1,1,1
Seems a [good idea][1]	1
This [provides the user with an insight into the level of distress they are managing - a reality check][1]. Some may be resistant to other supports if they do not have this awareness	1
[Although reliability and validity of answers cannot always be assured.][3]	3
[Important][1], as some people will not realise the severity of the stress and anxiety they may be feeling. This [may encourage users to seek help from a GP where required.][1]	1,1
[Might be helpful][1] if scale is appropriate. [May need to be tested][3]	1,3
that appears to be [a good idea][1]	1
[not enough context really][3] to [make an informed decision on this one][4]	3,4
[Depends on the goals of the platform][3, 4] - not all users will be enthusiastic about filling out a form	3,4
I found most midwives underplay what is happening to them and [would probably not self evaluate well therefore][2]	2

	Theme	Number of times categorised
1.	Simple user assessment - Helpful	25
2.	Simple user assessment - unhelpful	2
3.	Simple user assessment - Context for use required	11
4.	Simple user assessment - Undecided	3
5.	Simple user assessment - Should be optional	1

Do you have any additional comments you would like to share?

Comment	Themes Assigned to
[which scale - which aspect of distress] [1]<br>[prompt to what] [1]<br>[at this point no decision made as to whether there is a chat facility, real time support peer support, legal advice etc][1]	1,1,1

	Theme	Number of times categorised
1.	Simple user assessment - Context for use required	3

An online intervention designed to support midwives in work-related psychological distress should prioritise the gathering of anonymised data and concerns from users, only with explicit permission, so that trends and concerns may be highlighted at a national level.

17.1 The gathering of anonymised data and concerns from users, only with explicit permission, so that trends and concerns may be highlighted at a national level.

Rank value	Option	Count
1	Not a priority	2
2	Low priority	2
3	Somewhat a priority	2
4	Neutral	5
5	Moderate priority	8
6	High priority	10
7	Essential priority	15

Mean rank	5.39
Variance	2.87
Standard Deviation	1.7
Lower Quartile	4.75
Upper Quartile	7.0

Consensus Achieved = No

Minimum score = Not/Low/Somewhat a priority 2/2/2 (4.5%/4.5%/4.5%)

Maximum score = Essential priority 15 (34.1%)

The implementation of an initial simple user assessment using a psychological distress scale to prompt the user to access the most suitable support available

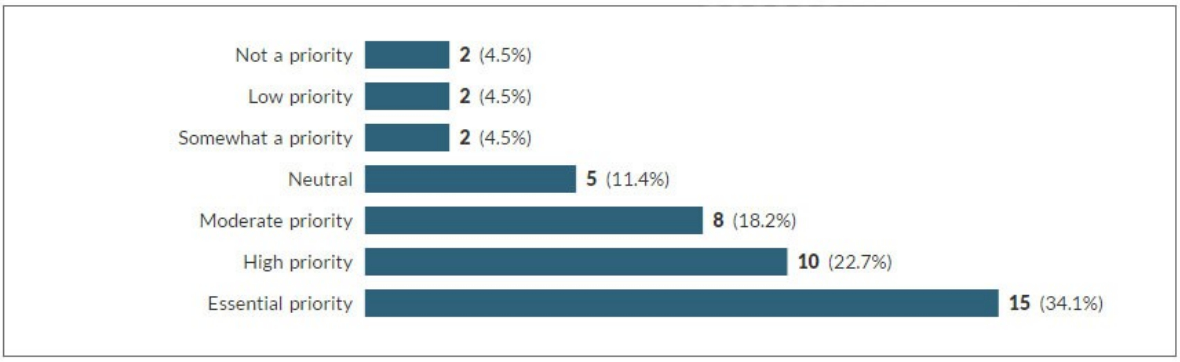

Thematic analysis of open text responses

Why did you choose this rating of priority?

Comment	Themes Assigned to
[Uncomfortable about mixing research with intervention][1]. Hard to know what sample is so [likely to be written off as unrepresentative][2]. [Service use figures make sense][1] but anything that treats the visits as a sample [I would be concerned about][2].	1,2,1,2
[Critical that trends are identified][1] and strategies developed to address those trends at a national level	1
this [might be useful][1, 4]	1
[Not sure there's a problem][1, 4] if its "anonymised" unless it been in the press in an identifying way.	1,4
if anonymised there is [potential for mischief][2] so [can't always be sure each post is a bonafide case][2]	2,2
this [needs to be clarified][1] when first logging in	1
[to raise awareness][1] of the present lack of support for midwives and how this may impact on their health	1
Hmm well if you collect data by convenience (only those who provide permission) rather than everyone then [you will not be able to determine trends!!!][2]	2
[Providing that it is anonymous][3], [data should be available at every level to highlight trends and concerns.][1]	3,1
To highlight the extent of the problems [would be excellent][1]	1
[Yes, this would be beneficial][1] to generate evidence to inform service provision, organisational change, supportive structures etc.	1
[Vital][1] for mapping future change, education research	1
[Good idea][1] to collect evidence for pushing for better support for midwives. But would have to be [guaranteed anonymously gathered][3], as otherwise midwives may not want to open up.	1,3
[Essential to highlight the potential degree of distress in our industry][1]	1
This [could put some people off][2] if they knew their information, issues and comments are used for research	2
[Please- anything that helps][1] the state we're in to be widely recognised [would be so welcome.][1]	1,1
[we need more data][1]. [absolutely fundamental][1].	1,1
[Useful to monitor the trends][1] and looking into ways to reduce	1
I [don't think this should be necessarily a goal][2], but through a democratic decision the forum users could decide this at a later date depending on the issues raised.	2
[May be biased][2] - [would be good to compare answers with those given by non-distressed midwives][1]	2,1
[Statistical data to facilitate change in staff support systems in the NHS is essential][1]	1
Again - this [should be international][1] so that ICM could see what was affecting midwives and where, and are they similar issues and how could they support/deal with it.	1
[This would be very useful][1]	1
[Very important][1], [this will offer valuable information][1] and [can be shared in a bid to make improvements][1]	1,1,1

This management of the data will be providing a basis for the most targeted measures [to minimise future distress][1]	1
[Extremely vital][1] given the increased pressures on the NHS as a whole.	1
[It's a nice idea]1, but I think in practice, the permission may be varied, hence the results of [any national trend finding will be skewed.][2]	1,2
[Anonymised stories themselves can inform policy making][1]. [Data gathering likely to put off participants.][2]	1,2
[any data collection should be part of an ethics supported study][5] and [separate informed consent should be obtained on each occasion][5]	5,5
This is a wide spread problem and there is a lack of openness about it that [such information would dispel][1]	1

	**Theme**	**Number of times categorised**
1.	The gathering of anonymised data and concerns - Helpful	30
2.	The gathering of anonymised data and concerns - Unhelpful	10
3.	Midwives - Require anonymity	2
4.	The gathering of anonymised data and concerns - Undecided	1
5.	The gathering of anonymised data and concerns - Requires ethical consideration	2

Do you have any additional comments you would like to share?

**Comment**	**Themes Assigned to**
[this may then drive services forward to implement change][1]	1
[if anonymised do you need consent?][4]	4
[confidentiality is vital][3] - [anonymised data is important][1] but [reporting would be biased as we would only be reporting distress data with no denominator][2]	3,1,2

	**Theme**	**Number of times categorised**
1.	The gathering of anonymised data and concerns - Helpful	2
2.	The gathering of anonymised data and concerns - Unhelpful	1
3.	Midwives - Require confidentiality	1
4.	The gathering of anonymised data and concerns - Requires ethical consideration	1

18

An online intervention designed to support midwives in work-related psychological distress should prioritise access for a midwife's friends and family members

18.1 Access for a midwife's friends and family members

Rank value	Option	Count
1	Not a priority	17
2	Low priority	8
3	Somewhat a priority	1
4	Neutral	9
5	Moderate priority	8
6	High priority	1
7	Essential priority	0

Mean rank	2.68
Variance	2.76
Standard Deviation	1.66
Lower Quartile	1.0
Upper Quartile	4.0

Consensus Achieved = No

Minimum score = Essential priority 0 (0%)

Maximum score = Not a priority 17 (38.6%)

Access for a midwife's friends and family members

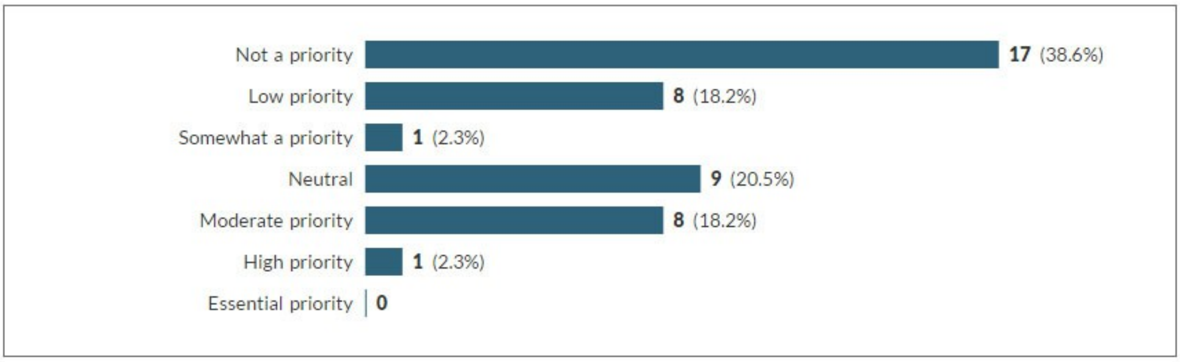

Thematic analysis of open text responses

Why did you choose this rating of priority?

Comment	Themes Assigned to
Link to purpose...and [if it's online it would be likely that anyone could access] [4] - and [if identifiers are required it won't get used anyway][4]	4,4
[just if she gives her consent][4]	4
[Not sure about this one][3] as [anonymity would be compromised][4]	4,3
[It is for the midwife][2]	2
[that's not anonymous!][4]	4
[to what end?][6]	6
[not sure how this would be beneficial][2, 3]	2,3

[could be useful][1] but I would [need to know more about how this would work][6] before being able to prioritise it. e.g. [family members would not be bound by the same code of ethics and professional conduct so this would need to be thought through][4].	1,6,4
This [might be useful][1] for some midwives but it also [could be overrun by too many extra people.][2]	1,2
[Unsure about this][3] [don't have enough information.][6]	3,6
[I can't see how this would work exactly][3] [in terms of keeping things anonymous][4], [but perhaps][1].	3,4,1
Friends and family need to be able to support a midwife but this [could be limited access][5]	5
[Maybe good][1] if a serious issue comes up.	1
There [could be a friends and family section][5] where they could ask for support with coping with their midwife and how best to help/ raise concerns. But this should be an add on rather than a main feature and separate from Midwife- only areas.	5
[nope...only for midwives please.][2]	2
[Should be only for Midwives][2] and private to avoid fears of incrimination as well as being a forum to speak honestly	2
[Absolutely not!][2] Would reduce the level of openness and honesty as some distress maybe attributed to friends and family.	2
[Not sure how helpful this would be][3]	3
[I'm not sure if I would class this as a priority or not][3]. The home page could include good help and support links and contact numbers of agencies that could offer help. I would [worry about the confidentiality of the midwife][4].	3,4
[I see this as being midwife specific.][2]	2
[I don't think it can be all things to all people][2] - it may be [better concentrate on being a good resource for midwives][2]. [Maybe just some signposting for friends and family?][5]	2,2,5
[I'm not sure of the benefit of this][3]	3
While family and friends provide important support, [the needs of the midwife should remain paramount.][2]	2
Maybe an option overtime, but [feel priority should be given to health professionals in the first instance][2] and following audit and evaluation of platform.	2
This [would be a support tool for the midwife][2], and being amongst other midwives will be key to its success. [Friends and family would need to seek support from other sources.][2]	2,2
[Likely to be conflicts here if stress at home is part of the distress being experienced][2, 4]	2,4
[There could be a separate area for friends?][5]	5
[should be just about midwives][2]	2
I think this [should be for reporting midwives only][2]	2
[This would need to be done very carefully if put in place][4]	4

	**Theme**	**Number of times categorised**
1.	Access for a midwife's friends and family - Helpful	4
2.	Access for a midwife's friends and family - Unhelpful	16
3.	Access for a midwife's friends and family - Undecided	7

4.	Access for a midwife's friends and family - Ethical considerations must be recognised	10
5.	Access for a midwife's friends and family - Could require a separate, designated area	4
6.	Access for a midwife's friends and family - Need more information	3

Do you have any additional comments you would like to share?

**Comment**	**Themes Assigned to**
[Maybe this would work if there was a separate forum for family and friends of distressed midwives?][1]	1
[This needs to be a space for midwives, with midwives][2].	2
[Advice on the services etc available for access by family and friends could be provided][1]	1

	**Theme**	**Number of times categorised**
1.	Access for a midwife's friends and family - Could require a separate, designated area	2
2.	Access for a midwife's friends and family - Unhelpful	1

19

An online intervention designed to support midwives in work-related psychological distress should prioritise the following up and identification of those at risk

19.1 Following up and identification of those at risk

Rank value	Option	Count
1	Not a priority	2
2	Low priority	1
3	Somewhat a priority	1
4	Neutral	5
5	Moderate priority	7
6	High priority	12
7	Essential priority	16

Mean rank	5.59
Variance	2.51
Standard Deviation	1.59
Lower Quartile	5.0
Upper Quartile	7.0

Consensus Achieved = Yes (High/Essential priority) 63.7%

Minimum score = Low/Somewhat a priority 1/1 (2.3%/2.3%)

Maximum score = Essential priority 16 (36.4%)

Following up and identification of those at risk

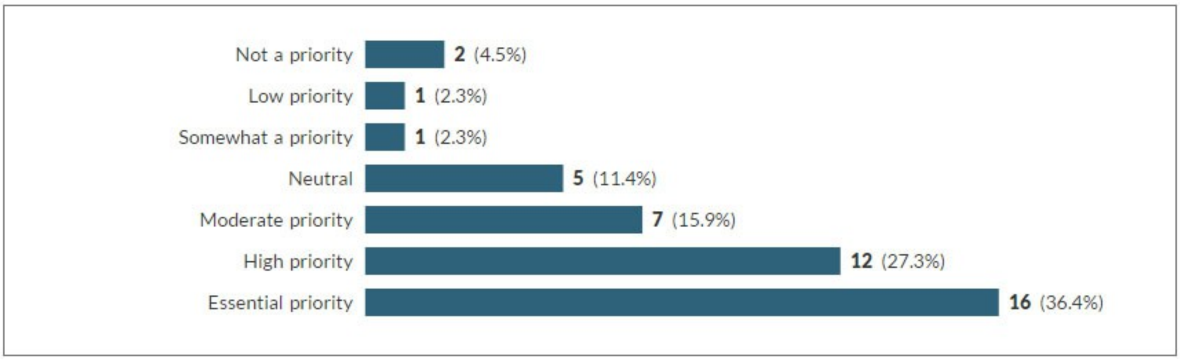

Thematic analysis of open text responses

Why did you choose this rating of priority?

Comment	Themes Assigned to
[It is a failing in duty of care to encourage someone to open up and access distress and not have adequate support in place to manage the fall out from this.][1]	1
[just if she is willing][1] and [gives her consent][3]	1,3
If suicidal behaviour is conveyed through the postings, [that person needs following up][1]	1
identification of [high risk issues needs attention][1]	1
[Obviously.][1]	1
I think [this is difficult to do][3]...and with [anonymised system what would you do][3] (unless only anonymised posts but system logs accurate user information). I [don’t think we can take responsibility for all posts][2] but can [display general warning to contact GP or A&E if sever distress and worrying about self harm][1]	2,3,3,1
[not sure][4] - there would be signposts to sources of support	4
it would be [difficult to assess][3] who would require follow up	3
Yes, but [depends what you mean by "risk"][4] and [this would need to be explicitly stated at the start.][4]	4,4
[A serious psychological problem cannot be ignored.][1]	1
Encouraging follow up [can prevent slipping through][1] as too busy to continue to engage. Easy to prioritise work before own well being	1
[I'm undecided about this][4]	4
[ethical][1]	1
Suicide and self-destructive behaviours have been identified in healthcare professionals, [this seems a very sensible idea.][1]	1
Safety is imperative and if there were major concerns [it is important this is addressed][1]	1
[How are you going to do this if it's anonymous?][3] It's a [nice idea...][1]	3,1
think that is [too much to ask from an online forum][2], [but signposting mws in the right direction is a must.][1]	2,1

If it will help to direct you to getting help then this [would not be required][2]	2
The [forum would have to be completely transparent that user may be followed and identified as 'at risk'][3], this could [only occur with explicit consent prior to their signing up][3]. This [feels like potential 'policing]'[2], also [clarity of what 'at risk' means is required][3]. [Very ambiguous][4].	3,3,2,3,4
[As long as][1] [the user has given their consent to be followed up when signing in to the platform][3]... [some users may not want any contact][2] and may be using the platform to off load and debrief in a safe, [one way environment.][2]	1,3,2,2
Feel it [would be of benefit][1] to midwives using the program	1
Like Samaritans it [should be available for and led by the person in distress.][3]	3
[otherwise it is just a collection of concerns][1]	1
The [system would be incomplete][1] if there were midwives accessing the service and then not having the support if they are at risk	1
[Essential][1], especially if serious risk of harm is identified	1
As per previous answer, any strong and serious risk to the user or another person, such as suicide ideation or talk of harming someone, [would need to be followed up.][1]	1
[Essential priority][1] where there are safeguarding concerns. Otherwise, [need sensible position on duty of care.][3]	1,3
I feel [torn on this question][4] as i think providing a platform for [anonymity is important][3] but I also think we have a [moral obligation][3] to [provide real time support to those identified to be at risk][1]	4,3,3,1
Self evaluation of the seriousness of the situation is [not always reliable][2, 3]	2,3

	**Theme**	**Number of times categorised**
1.	The following up and identification of those at risk - Helpful	21
2.	The following up and identification of those at risk - Unhelpful	7
3.	The following up and identification of those at risk - Challenging	14
4.	The following up and identification of those at risk - Undecided	6

Do you have any additional comments you would like to share?

**Comment**	**Themes Assigned to**
I [worry about the limits of what an it interface can offer][3] [combined with the impact of anything that encourages self examination][2] -it's bad enough when checking online for eg mole patterns on skin.	3,2
Following up those at risk [may help them to feel cared about][1] - [an essential ingredient][1] for mental and emotional health and wellbeing	1,1
I must say that if I was wanting to have a general debrief about a horrid shift, or situation that was distressing me I think [I would not participate or withdraw][2] if I was constantly been told I was "at risk" and asked if I wanted to be referred! Whilst there is no doubt that being in distress makes you vulnerable I also think [we can go too far][3] when [most of the responsibility for this identification and referral lies with the person themselves][3]	2,3,3

	Theme	Number of times categorised
1.	The following up and identification of those at risk - Helpful	2
2.	The following up and identification of those at risk - Unhelpful	2
3.	The following up and identification of those at risk - Beyond the scope of this project	3

20

An online intervention designed to support midwives in work-related psychological distress should prioritise the provision of a general statement about professional codes of conduct and the need for users to keep in mind their responsibilities in relation to them

20.1 The provision of a general statement about professional codes of conduct and the need for users to keep in mind their responsibilities in relation to them.

Rank value	Option	Count
1	Not a priority	1
2	Low priority	4
3	Somewhat a priority	3
4	Neutral	7
5	Moderate priority	9
6	High priority	8
7	Essential priority	12

Mean rank	5.07
Variance	2.88
Standard Deviation	1.7
Lower Quartile	4.0
Upper Quartile	7.0

Consensus Achieved = No

Minimum score = Not a priority 1 (2.3%)

Maximum score = Essential priority 12 (27.3%)

The provision of a general statement about professional codes of conduct and the need for users to keep in mind their responsibilities in relation to them

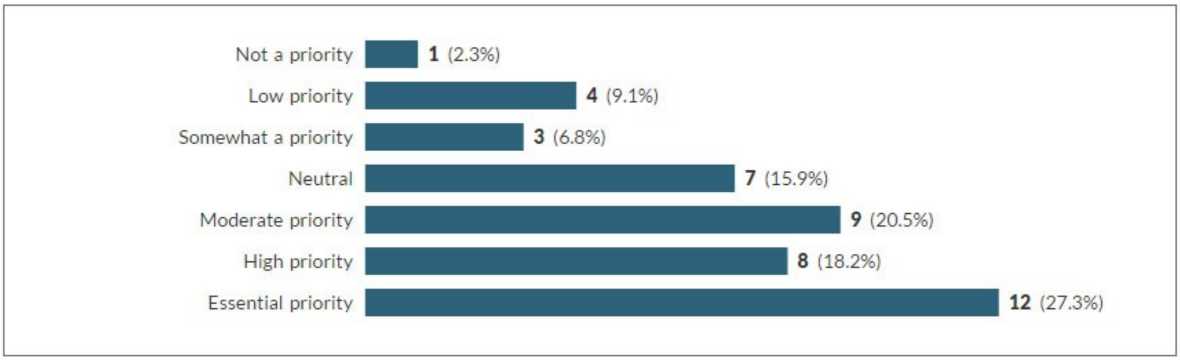

Thematic analysis of open text responses

Why did you choose this rating of priority?

Comment	Themes Assigned to
Already answered this one? [Self care not well developed in professional codes][3]	2
as per previous answer re [stating professional conduct and accountabilities][1]	1
[important to remind midwives][1]	1
[Yes][1], well we are discussing situations where something has patently gone awry?	1
is this not asked previously?	0
[if it is intended just for midwives][1]	1
[Well yes][1] but as I said above if you are planning to include family and friends then you wont be able to get them to keep these "in mind" if they are not [applicable] [4]	1,4
A similar question was asked earlier, [beware of sounding too much like the NMC.][2]	2
[We are aware of the code][5] but reminder in a time of distress [can only be beneficial][1]	1,5
I would [rather see a platform user responsibility and code of conduct][6] which may include a professional responsibilities statement	6
[Need to create awareness of our code][1]	1
[Important for baseline of use][1]	1
Midwifery is not always black & white. [Professional conduct reminder yes][1]. [We are always mindful of them][5] - this [could be considered patronising.][2]	1,5,2
I think that this is important, but also hypocritical if you are stating that midwives have professional responsibilities but then potentially have a space where we can read about severe misconduct/ worrying attitudes/ illegal practices but not do a thing about it if we felt it was necessary.	5
[All should be aware] [5] of the [responsibilities of the code][1]	5,1
[Just include link to Code on NMC website][2]	2
I think this was covered in an earlier question. I answered that [there should be a reminder of the Code]1 within a [set of clear Terms and Conditions when signing up to use the platform][6].	1,6
If this is international there would be [many codes of conduct depending on where you live][4]. ICM has general statements then there could be links to individual country licensing bodies.	4

[Yes][1] I don't have a problem with this. There is still a need to be mindful of these.	1
[This is fundamental][1] to our work and practice	1
[This statement is required][1] not just for the benefit of midwives, but also for those in their care	1
The weight of these responsibilities [may be partially responsible for the workplace stress][2]. Therefore, a reminder of such heavy responsibility [may be counter-productive][2], if not phrased and used very carefully.	2,2
[Unlikely to be very effective][2]	2
See previous comments (prompts should be to seek help - where to get help and then perhaps say why [but prompts to regulation could make people feel worse][2])	2
Needs to be supportive of the individual midwife but [keeping her professionally safe is also important][1]	1

	**Theme**	**Number of times categorised**
1.	A general statement about professional codes of conduct - Helpful	15
2.	A general statement about professional codes of conduct - Unhelpful	8
3.	A general statement about professional codes of conduct -Better to focus on self-care (Theme removed following a process of secondary analysis).	0
4.	A general statement about professional codes of conduct - Must be applicable	2
5.	Midwives - Already aware of codes	4
6.	Online community - Should develop its own codes of conduct	2

Do you have any additional comments you would like to share?

**Comment**	**Themes Assigned to**
[I wonder at why you are trying to force consensus by repeating the questions until we all agree][1]. I am also not necessarily sure that you can say you haven't gotten consensus on the repeat questions just because there is a broad response such as thing as agreeing to disagree and also [some of the questions you have repeated to try to get "consensus" I really don't see the point of][1]. EG how the platform looks is completely irrelevant to me and always will be [I am much more interested in what it will do and how it will support our distressed colleagues than how it looks][1]. As technology changes it will inevitably change too so how it looks today will not be how it looks in 5 years [surely you don't plan to go back to the panel in 5 years and say please can we all agree to change the way it looks !!][1]	1,1,1,1
Comment removed for confidentiality reasons.	0
Aspects like this make the project seem weak- [it can't be completely anonymous and free of consequence, yet responsible and accountable at the same time][1]. Those things don't marry up.	1
if used [should be used in the positive][2] - [reminding midwives of the obligations of other too re treating people with respect][2] - [to empower the midwives to know she can speak out against work based injustice, lack of support etc.][2]	2,2,2

	Theme	Number of times categorised
1.	Achieving consensus - Frustrated by survey questions	5

2.	A general statement about professional codes of conduct - Requires sensitivity	3
